# Unhooking the Hook: Optimization of the Aurora A Targeting PROTAC **JB170** to **CCT400028**, an *In Vitro* Degrader Chemical Probe

**DOI:** 10.1021/acs.jmedchem.5c03024

**Published:** 2026-01-12

**Authors:** Jack A. O’Hanlon, Katrin Gutsche, Juliane Elisabeth Müller, Nihar Ranjan Prusty, Amin Mirza, Theodoros I. Roumeliotis, Hengzhang Yang, Mark Stubbs, Stephen T. Hallett, Lorenz Eing, Peter Craig McAndrew, Jyoti Sharma Choudhary, Yann-Vaï Le Bihan, John Caldwell, Rob L. M. van Montfort, Paul Workman, Elmar Wolf, Gary K. Newton, Lindsay E. Evans

**Affiliations:** a Centre for Cancer Drug Discovery, 5053 The Institute of Cancer Research, London SM2 5NG, U.K.; b Cancer Research UK Children’s Brain Tumour Centre of Excellence, 5053 The Institute of Cancer Research, London SM2 5NG, U.K.; c Institute of Biochemistry, University of Kiel, Rudolf-Höber-Straße 1, Kiel 24118, Germany; d Functional Proteomics Group, Institute of Cancer Research, Chester Beatty Laboratories, 237 Fulham Road, SW3 6JB London, U.K.; e Chair of Biochemistry and Molecular Biology, University of Würzburg, Am Hubland, Würzburg 97074, Germany; f Division of Structural Biology, 5053 The Institute of Cancer Research, London SM2 5NG, U.K.

## Abstract

Proteolysis TArgeting Chimeras (PROTACs) can be used to target both the catalytic and noncatalytic functions of a protein, which can be particularly beneficial for proteins with important scaffolding functions like Aurora A. However, instability, poor selectivity profiles, and the hook effect often limit the applicability of PROTACs as chemical probes. In this study, we report the development of **CCT400028**, a second-generation alisertib-derived Aurora A PROTAC. The hook effect was removed through rational optimization of the CRBN-targeting warhead to decrease affinity for cereblon, which, combined with improved stability to hydrolysis, expands the range of concentrations and duration at which maximal degradation can be achieved. Potent Aurora A degradation was shown in three pediatric tumor cell lines, as well as excellent selectivity and on-target mechanism of action. **CCT400028** and a matched inactive control analogue fulfill the criteria for a degrader chemical probe for studying Aurora A degradation *in vitro*.

## Graphical abstract

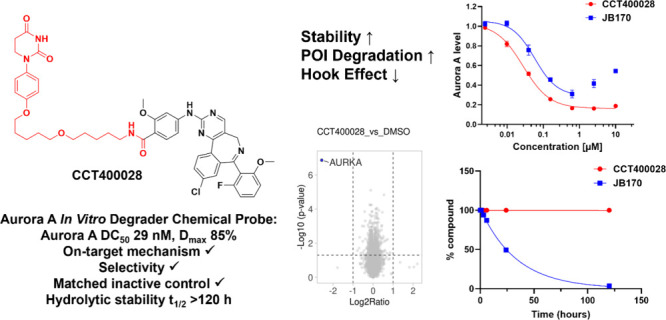

## Introduction

The Aurora family of serine/threonine kinases (Aurora A, B, and C) play important roles in cell cycle progression and the regulation of mitosis through their catalytic activity. Aurora A and B are ubiquitously expressed and have distinct roles during different phases of mitosis, whereas Aurora C is primarily expressed in the testes.^1–3^ Aurora A regulates centrosome maturation before mitotic entry and mitotic spindle assembly. Aurora B is a key component of the chromosomal passenger complex and plays a role in chromosomal alignment, passing the spindle assembly checkpoint, separation of sister chromatids, and formation of the cleavage furrow. During mitotic exit, Aurora A and B are ubiquitinated by the anaphase-promoting complex (APC/C) to enable their proteasomal degradation.^1–3^

Aurora A overexpression is observed in many tumor types and often correlates with poor overall survival.^4^ Aurora A has been extensively investigated as a target for kinase inhibition in a variety of cancer settings. However, while therapeutic activity has been observed with Aurora A kinase inhibitors, they also cause dose-limiting neutropenia and no drug candidates have achieved clinical approval.^4–9^ One clinical candidate, alisertib, progressed into a phase III study in patients with relapsed or refractory peripheral T-cell lymphoma.^10^ Although well tolerated, alisertib failed to show greater efficacy than the comparator arm.^11^

In recent years, there has been increasing focus on converting kinase inhibitors into Proteolysis TArgeting Chimeras (PROTACs) that can degrade a protein of interest (POI). PROTACs have the advantage of targeting both the catalytic and scaffolding functions of the target protein and could therefore provide a more effective treatment approach.^12^ Aurora A has been reported to have important scaffolding functions, including the stabilization of N-myc,^13,14^ a key oncogenic driver in several pediatric cancers including neuroblastoma, medulloblastoma, and glioma.^15–17^ Furthermore, PROTACs have been shown to selectively degrade a specific protein among closely related proteins where it has proven challenging to achieve selectivity with inhibitors.^18,19^ A PROTAC strategy could therefore be beneficial in terms of targeting Aurora A while sparing Aurora B. A variety of Aurora A degraders have been developed from both selective and promiscuous kinase ligands (Figure 1).^20–30^ However, comprehensive selectivity profiling is not always reported and most of the published PROTACs have moderate levels of maximal Aurora A degradation and/or severe hook effects. Most reported examples are based on a thalidomide cereblon (CRBN) binding warhead that is known to undergo hydrolysis at physiological pH;^31^ it is therefore unclear how suitable these PROTACs are for longer-term biological studies.

**Figure 1:**
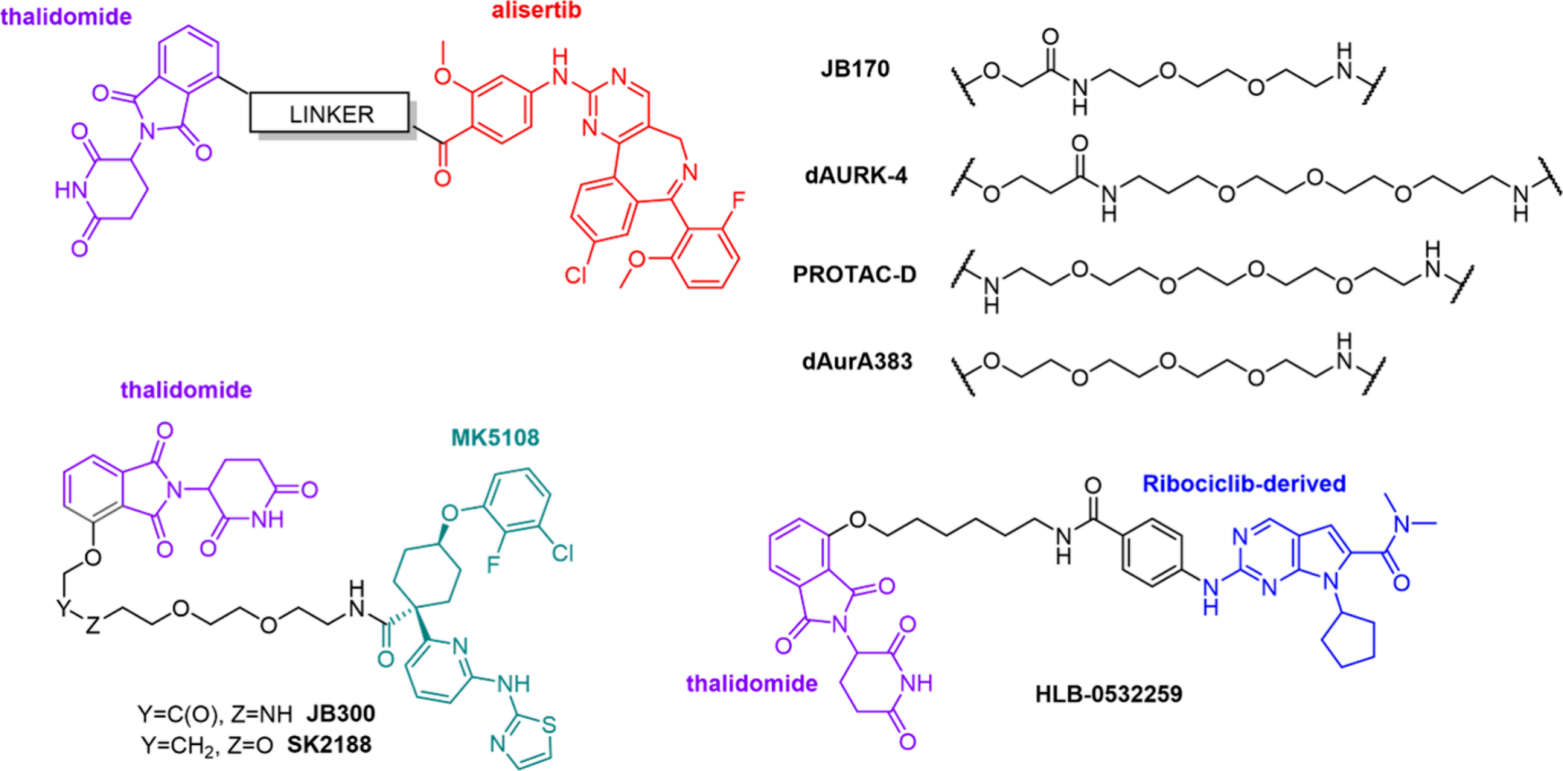
Chemical structures of selected previously reported Aurora A PROTACs derived from alisertib, shown in red (**JB170**,^27^ **dAURK-4**,^20^ **PROTAC-D**,^23^ and **dAurA383** ^24^), **MK5108**, shown in green (**JB300** ^21^ and **SK2188** ^22^), and ribociclib, shown in blue (**HLB-0532259** ^25^).

One of the most comprehensively profiled examples is **JB170**, an alisertib-derived PROTAC that displayed rapid degradation of Aurora A with a reported *D*_max_ of 69% and a DC_50_ of 28 nM, as measured by a HiBiT assay in MV4-11 pediatric leukemia cells.^27,32^ **JB170** showed high levels of degradation selectivity against Aurora B and versus the wider kinome. However, **JB170** suffers from a severe hook effect and a moderate half-life relative to the duration of typical cell-based viability assays (*t*_1/2_ = 25 h in PBS buffer at pH 7.4).^27^

Therefore, the aim of this present work was to develop **JB170** into a more versatile PROTAC chemical probe with increased stability and reduced hook effect, allowing use of a wider concentration range and length of exposure. PROTACs with this profile would be better suited to examining the effects of Aurora A degradation in cancer cell lines *in vitro*.

## Results and Discussion

### Aurora A PROTAC Design and Synthesis

The commercially available Aurora A PROTAC **JB170** was taken as the starting point for our optimization. Adhikari et al.^27^ had previously explored the effects on Aurora A degradation of altering linker length and/or switching from a CRBN to a VHL-based system. During our work, Liu et al.^24^ reported **dAurA383** (Figure 1). Like **JB170**, **dAurA383** is an Aurora A PROTAC derived from thalidomide and alisertib but contains a purely PEG- based linker.^33^ However, non-PEG linkers and changes to the CRBN-binding warhead had not been reported for this PROTAC series. We therefore initially explored changes to the linker to reduce the number of hydrogen-bond donors and increase rigidity (Figure 2). With the selected linker in place, CRBN warheads were screened with the aim of minimizing the hook effect. The PROTACs were prepared according to the synthetic strategies outlined in Scheme 1.

**Figure 2:**
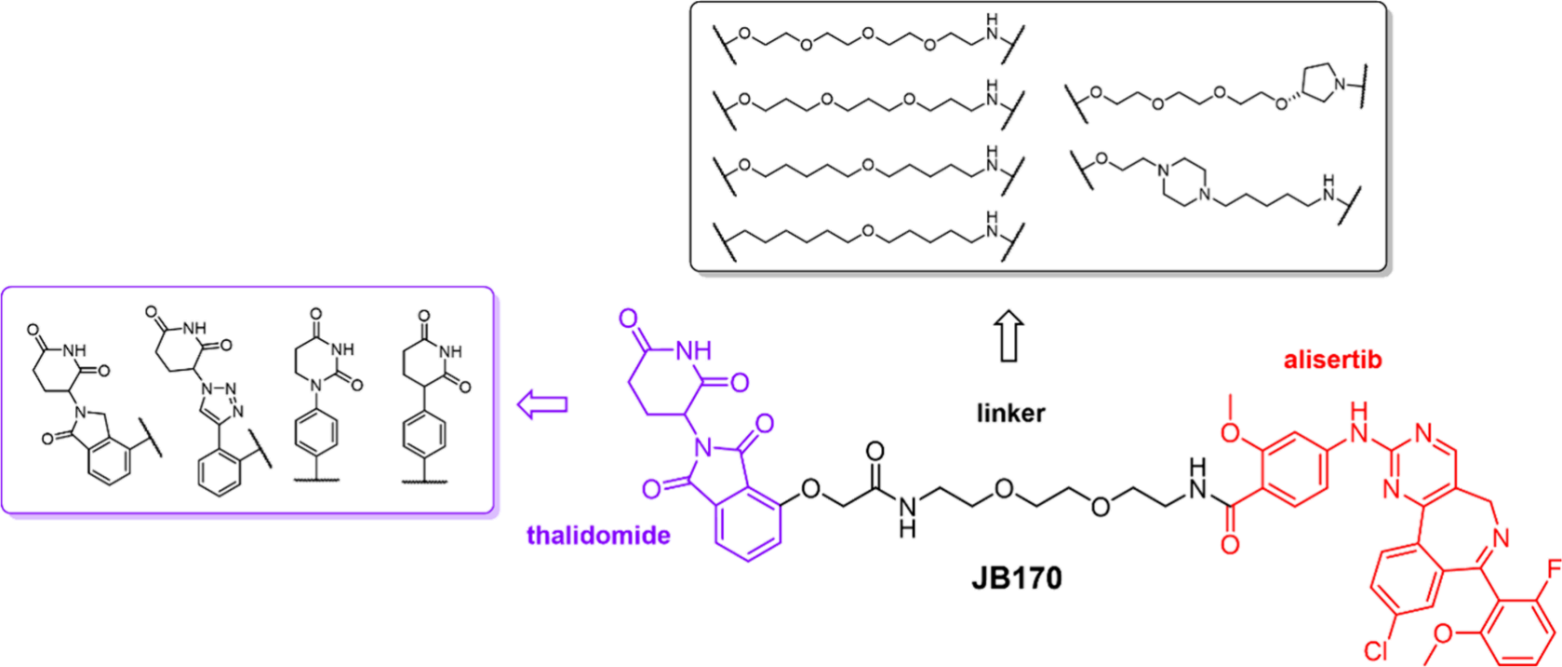
Summary of structural changes to **JB170** made during this work.

**Scheme 1:**
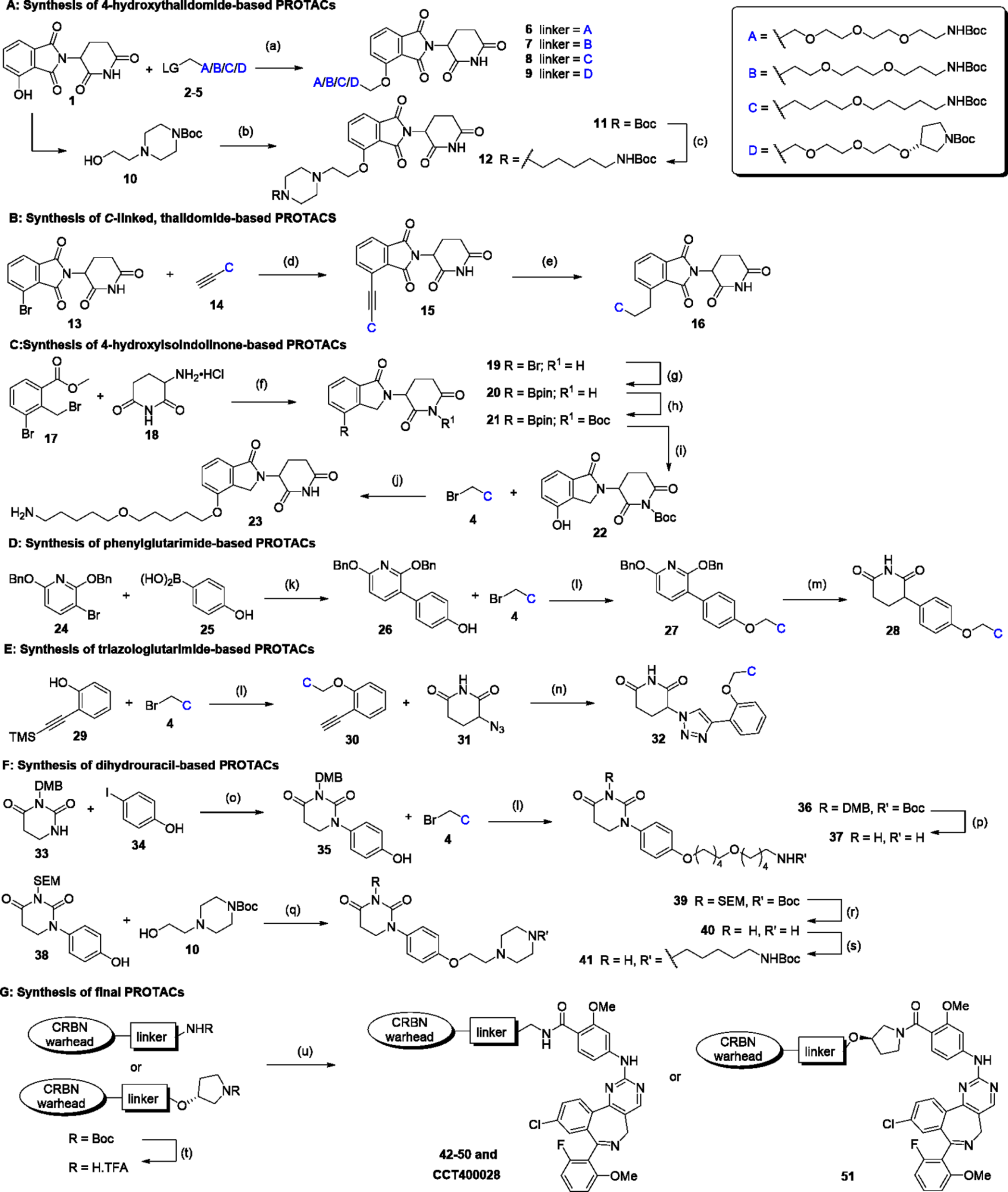
Synthetic Routes Used to Prepare PROTACs 42–51 and CCT400028^a^

a Reagents and conditions: (a) NaHCO_3_, DMF, 60–80 °C; (b) DTBAD, PPh_3_, THF, 0 °C–rt; (c) 1. TFA, CH_2_Cl_2_, 2. *tert*-Butyl *N*-[5-(tosyloxy)­pentyl]­carbamate, NaHCO_3_, DMF, 80 °C; (d) Pd­(PPh_3_)_2_Cl_2_, CuI, Et_3_N, 1,4-dioxane, 80 °C, μW; (e) H_2_ (g), Pd/C, MeOH; (f) DIPEA, MeCN, 82 °C; (g) Pd­(dppf)­Cl_2_, KOAc, B_2_pin_2_, 1,4-dioxane, 90 °C; (h) Boc_2_O, DMAP, 1,4-dioxane; (i) H_2_O_2_ (aq), DMF; (j) 1. NaHCO_3_, DMF, 85 °C, 2. TFA, CH_2_Cl_2_; (k) Pd­(dppf)­Cl_2_, Na_2_CO_3_, 1,4-dioxane/H_2_O, 100 °C; (l) K_2_CO_3_, MeCN, 82 °C; (m) H_2_ (g), Pd/C, EtOH; (n) CuSO_4_ (aq), sodium ascorbate, THF; (o) CuI, DMEDA, K_3_PO_4_, 1,4-dioxane, 60 °C; (p) TfOH, TFA; (q) DTBAD, PPh_3_, THF, 0 °C–rt; (r) TFA/H_2_O, 120 °C, μW; (s) *tert*-butyl *N*-[5-(tosyloxy)­pentyl]­carbamate, K_2_CO_3_, DMF, 35 °C; (t) TFA, CH_2_Cl_2_; (u) HBTU or HATU, DIPEA or NMM, DMF.

Typically, after preparation of the CRBN warhead, the Boc-protected linker was installed via alkylation of the phenol. For the carbon-linked thalidomide PROTAC (**45**), the linker was introduced via Sonogashira coupling and hydrogenation of the alkyne moiety. Deprotection of the CRBN warhead was followed by Boc deprotection of the linker and amide coupling with alisertib to afford the final PROTACs.

### Linker Optimization to Improve Aurora A Degradation Activity

We initially sought to improve physicochemical properties and gain additional degradation efficacy by reducing the number of hydrogen-bond donors in the linker.^34–36^ The oxyacetamide-containing linker of **JB170** was replaced with a PEG-4 chain to give **42** (**dAURA383**,^24^ Figure 1). In an Aurora A HiBiT assay in MV4-11 human pediatric leukemia cells, **42** showed an improvement in DC_50_ compared to **JB170** (DC_50_ = 6.9 and 39 nM, respectively) (Table 1 and Table S1).^27^ However, the *D*_max_ for **42** was reduced compared to **JB170** (56 and 69%, respectively) and a severe hook effect was present (Figure S1). The number of alkylether oxygens in the linker was then reduced from three in **42** to two in **43** or one in **44**. **43** and **44** had comparable activity to **JB170** in the Aurora A-HiBiT assay with similar DC_50_ values and *D*_max_ limited to <70% (Table 1 and Figure S1). Compared to JB170, **44** had an approximately 2-fold increase in half-life in a hydrolytic stability assay (Table 1 and Figure S2). Replacement of the aryl ether connection to the CRBN ligand with a direct alkyl attachment as in **45** was associated with a worsening of DC_50_ and *D*_max_ (Table 1 and Figure S1).

**Table 1:**
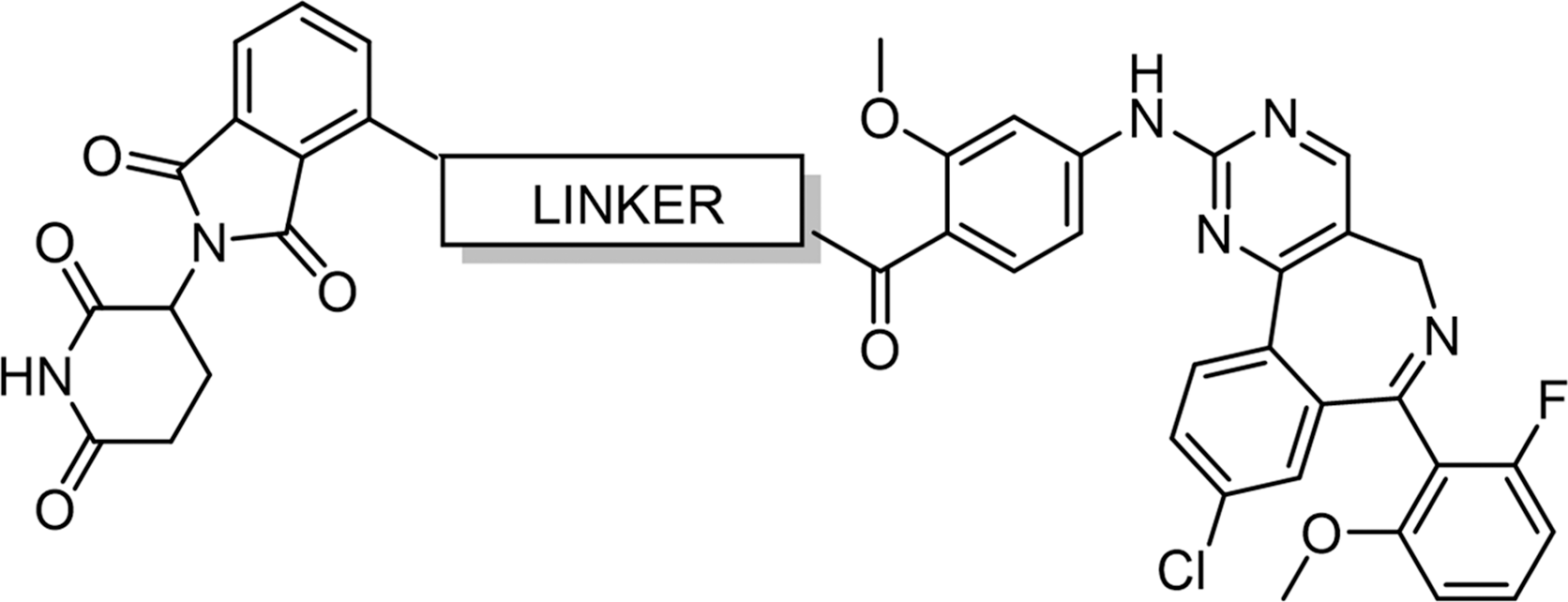

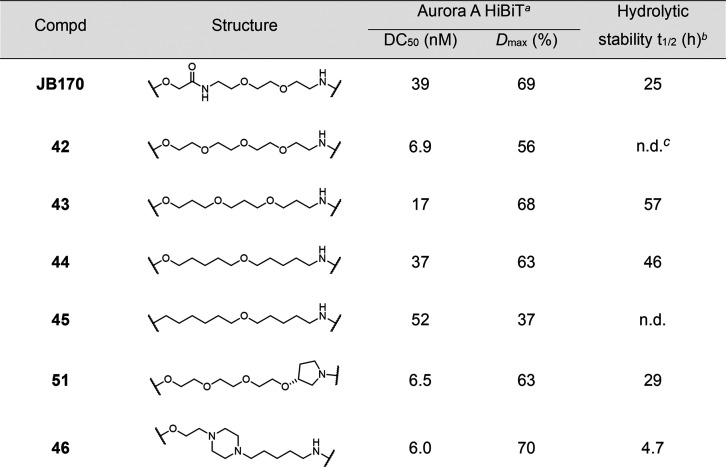
Structures of Aurora A PROTACs with Varying Linker Components, along with Data for Aurora A HiBiT and Hydrolytic Stability Assays

a Aurora A HiBiT in MV4-11 human pediatric leukemia cells with 6 h PROTAC treatment. DC_50_ data represent the geometric mean of at least three biological repeats. *D*_max_ data represent the mean of at least three biological repeats. See Supporting Information Table S1 for full statistics.

b Hydrolytic stability measured in PBS buffer (containing 10% DMSO) at pH 7.4.

c Not determined.

To reduce the linker hydrogen-bond donor count, we explored replacing the NH of the amide attachment to alisertib with a pyrrolidine ring to give **51**. In the molecular dynamics (MD) simulation model of the Aurora A/**JB170**/CRBN ternary complex reported by Adhikari et al.^27^ (Figure 3A), the section of the linker proximal to the amide attachment sits at the interface between Aurora A and CRBN. In this region of the linker, unfavorable gauche interactions are shown (dihedral angle of 46.6° between C–N and C–O bonds; Figure 3B). We hypothesized that introduction of the pyrrolidine ring would alleviate this energetically unfavorable conformation and lead to improved affinity for the ternary complex and increased cooperativity. In the Aurora A HiBiT assay, **51** showed an improvement in DC_50_ to 6.5 nM with a comparable *D*_max_ to the other compounds shown (Table 1, Figure 3C, and Table S1).

**Figure 3:**
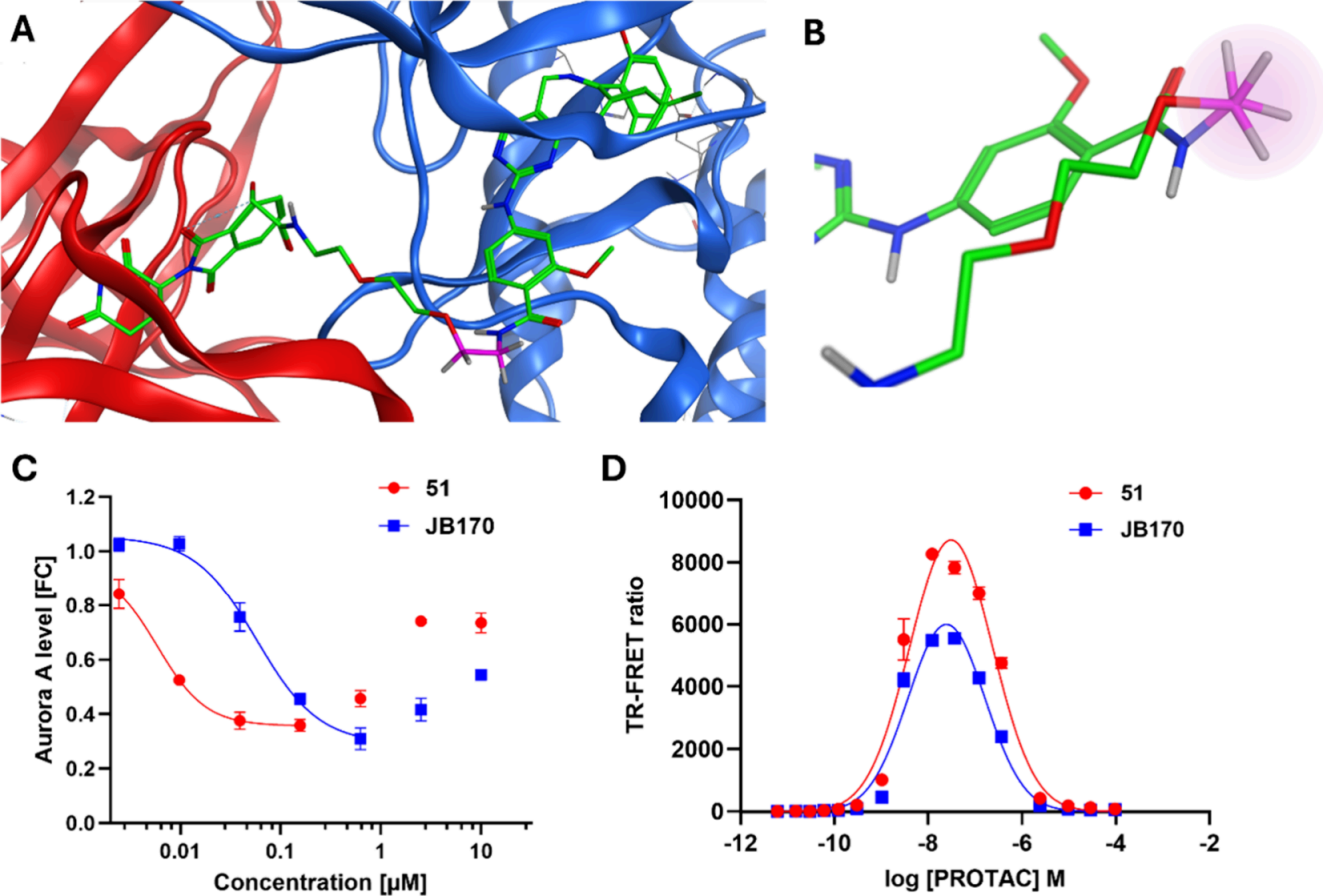
Design and activity profiling of **51**. (A) MD simulation model^26^ of the ternary complex of **JB170** (green) bound to CRBN (red) and Aurora A (blue); the σ bond with gauche interactions is shown in pink. (B) Close-up of the Newman projection along the σ bond (pink) exhibiting gauche interactions. (C) Representative Aurora A-HiBiT assay curves comparing the effect of **JB170** (blue) and **51** (red) on Aurora A protein levels in MV4-11 human pediatric leukemia cells (6 h PROTAC treatment). Each point represents the mean ± SEM, *n* = 3. (D) Representative TR-FRET assay curves comparing ternary complex formation for **JB170** (blue) and **51** (red) at varying PROTAC concentrations. Each point represents the mean ± SEM, *n* = 3.

### Characterization of the Ternary Complex Formation and Dissociation for **JB170** and **51**

To aid in the analysis and optimization of our PROTACs, a TR-FRET (time-resolved fluorescence resonance energy transfer) assay was set up to enable measurement of Aurora A/PROTAC/CRBN ternary complex formation and dissociation.^37,38^ Comparing the TR-FRET profile of **51** to that of **JB170**, increases in amplitude, area under the curve (AUC), and full-width at half-maximal points (FWHM) values were observed for **51**, while the concentration at which maximal ternary complex formation was achieved (E*C*_max_) remained constant (Figure 3D and Table S2; see Figure S3 for parameter descriptions). These changes are consistent with an increase in cooperativity (α; Figure 4),^38^ indicating that more efficient binding to the Aurora A/CRBN complex was achieved by rigidifying the linker and removal of the amide moiety.

**Figure 4:**
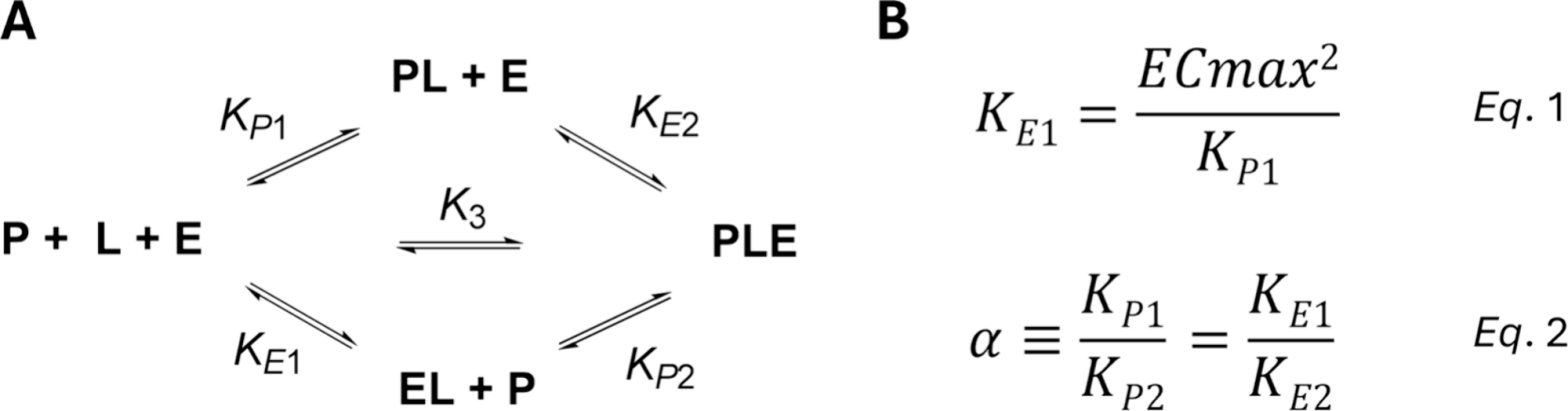
Mathematical description of ternary complex equilibrium, adapted from Han.^38^ (A) Binding interaction between protein (P), ligand (L), and E3 ligase (E) to form a ternary complex (PLE). In our system, *P* = Aurora A, L = PROTAC, and E = cereblon (CRBN). (B) Equations describing the relationship between EC_max_ and binary equilibrium dissociation constants *K*_*E*1_ and *K*_*P*1_ (eq 1), and betweenα and equilibrium dissociation constants (eq 2).

Using the extended LeastSumSquare (extLSS) method provided by Han,^38^ estimates of α can be determined from the TR-FRET profiles and the total concentrations of enzymes used in the assay. To obtain the best possible estimates of α, Han recommends calibrating the system using ligands with known equilibrium constants. The binary equilibrium dissociation constants for Aurora A (*K*_*P*1_, Figure 4A) were determined by Eurofins (Aurora A KdELECT binding assay; Table 2 and Figure S4). However, as a result of solubility issues at the concentration range required to resolve the weak binding interactions between the PROTACs and CRBN,^39^ we could not directly determine the binary equilibrium dissociation constant for CRBN (*K*_*E*1_, Figure 4A). *K*_*E*1_ values were calculated by applying eq 1 (Figure 4B) to the measured *K*_*P*1_ values, along with the EC_max_ values determined in the TR-FRET assay (Table S2). The extLSS method was applied to the equilibrium dissociation constants (*K*_*P*1_ and *K*_*E*1_) and TR-FRET data (Table S2) for **JB170** and **51**. This resulted in an estimated TR-FRET signal to [PLE] conversion factor (β) of 3700 and estimated α values of 24 and 110 for **JB170** and **51**, respectively (Table 2). TR-FRET profiles were simulated using Han’s Excel-based program for kinetic simulation of ternary complexes and the estimated values for α and β.^38^ The simulated profiles showed excellent agreement with the measured profiles (Figure S5A,C), thus increasing confidence in the validity of applying this approach to our PROTACs.

**Table 2:** Key Parameters Describing Ternary Complex Equilibria for **JB170** and **51**

**Compd**	**E**C_**max**_ **(nM)** ^a^	*K*_*P* **1**_ **(nM)** ^b^	*K*_*E* **1**_ **(nM)** ^c^	**α** ^d^	*K*_*P* **2**_ **(nM)** ^e^	*K*_*E* **2**_ **(nM)** ^e^
**JB170**	25	6.9	91	24	0.29	3.8
**51**	31	3.3	290	110	0.03	2.7

a EC_max_ was determined from the TR-FRET profile (Figure 3D and Table S2).

b *K*_*P*1_ was determined by Eurofins (Aurora A KdELECT binding assay, Figure S4) and are mean of *n* = 2.

c *K*_*E*1_ was calculated using eq 1.

d Cooperativity (α) was estimated using the extLSS method.

e *K*_*P*2_ and *K*_*E*2_ were calculated using eq 2.^38^

Using eq 2 (Figure 4B), values for *K*_*E*2_ and *K*_*P*2_ could be calculated from the estimated α value and the corresponding *K*_*E*1_ and *K*_*P*1_ values (Table 2). Switching from an acetamide containing PEG-based linker in **JB170** to a rigidified pyrrolidine-containing linker in **51** resulted in a >4-fold increase in α. This increase in α revealed a 10-fold reduction in *K*_*P*2_, supporting the hypothesis that rigidifying the linker would result in more efficient ternary-complex formation.

### Linker Rigidification by Introducing a Piperazine Ring

To explore rigidification of the linker further and attempt to reduce lipophilicity, a piperazine group was introduced, yielding **46** (Table 1).^40^ The piperazine ring was positioned toward the thalidomide warhead but away from either end of the linker to avoid disrupting warhead binding interactions and prevent contact with the two positively charged arginine residues at the exit of the alisertib-binding pocket.^27^ **46** was the most potent degrader of Aurora A in the HiBiT assay of the compounds tested so far (DC_50_ = 6.0 nM and *D*_max_ = 70% (Table 1 and Figure S1)). However, compared to **44** (*t*_1/2_ of 46 h), **46** showed a 10-fold reduction in hydrolytic stability (*t*_1/2_ of 4.7 h; Table 1 and Figure S2). We hypothesized that the poor hydrolytic stability could be due to the basic nitrogen atoms in the piperazine ring acting as intramolecular general base catalysts for the hydrolysis of the imide groups in the CRBN warhead (Figure S6).^41–43^

### Optimization of the CRBN Warhead to Reduce the Hook Effect and Increase *D*_max_

Through the linker modifications presented in Table 1, we achieved improvements in DC_50_ of up to 6.5-fold, but *D*_max_ remained ≤70%. Numerous factors have been reported to contribute to a partial degradation profile including slow degradation kinetics, selective degradation of a target subpopulation, competing deubiquitination, and impact of the hook effect.^44,45^ A significant hook effect could be observed for our PROTACs (Figure S1), which we hypothesized could be contributing to a limited *D*_max_. The hook effect occurs when binary-binding interactions between PROTAC and target or E3 ligase compete with the ternary-binding interaction between PROTAC–target–E3 ligase. It is well established that the hook effect can be reduced by increasing cooperativity (α) of the ternary complex formation.^44,46,47^ Considering eq 2 (Figure 4B), there are two options for increasing α:

1. Increase affinity of ternary-binding interactions (decrease *K*_*E*2_ or *K*_*P*2_) relative to the corresponding binary-binding interactions (*K*_*E*1_ or *K*_*P*1_)
2. Decrease affinity for binary-binding interactions (increase *K*_*E*1_ or *K*_*P*1_) relative to the corresponding ternary-binding interactions (*K*_*E*2_ or *K*_*P*2_)

Increasing α is commonly achieved by increasing the affinity of the ternary-binding interactions, without significantly altering binary-binding interactions. This is usually done empirically through the “linkerology” strategy for PROTAC optimization, where linker attachment vectors and linker components are varied, and typically requires a high number of analogues.^48,49^ An alternative approach to increasing α is to decrease the affinity of one of the binary-binding interactions (i.e., *K*_*E*1_), without significantly reducing the affinity of the ternary-binding interaction (i.e., *K*_*E*2_). An extreme example of this scenario is seen with molecular glues, which possess little or no affinity for the POI alone but have high ternary-complex formation affinity and correspondingly exhibit high levels of cooperativity and no hook effect. For our PROTAC series, we hypothesized that a reduction in the hook effect could be achieved by increasing α through decreasing the affinity for the least potent binary-binding interaction (*K*_*E*1_), provided the affinity for the ternary complex was not significantly impacted. Increasing *K*_*E*1_ would offer the additional benefit of raising the PROTAC concentration at which the binary-binding interaction with CRBN begins to compete with ternary-complex formation. We had identified several PROTACs with improved hydrolytic stability and improved or similar Aurora A degradation efficacy compared to **JB170** (Table 1). Of these, **44** was selected for further optimization of the CRBN warhead due to ease of synthesis. Ligands with a range of affinities for CRBN (**52**-**56**; CRBN FP assay IC_50_ values of 200–4800 nM, Table 3 and Table S3) and linker attachment vectors were selected,^50–54^ and a matched series of degraders was prepared. Thalidomide in **44** was replaced by an isoindolinone-glutarimide (IG),^55^ a triazologlutarimide (TG),^52,56^ a phenylglutarimide (PG),^50^ or a phenyldihydrouracil (PD)^57^ to give **47**, **48**, **49**, and **CCT400028**, respectively. The PROTACs were tested in the Aurora A HiBiT and hydrolytic stability assays (Table 3 and Figure 5A).

**Table 3:**
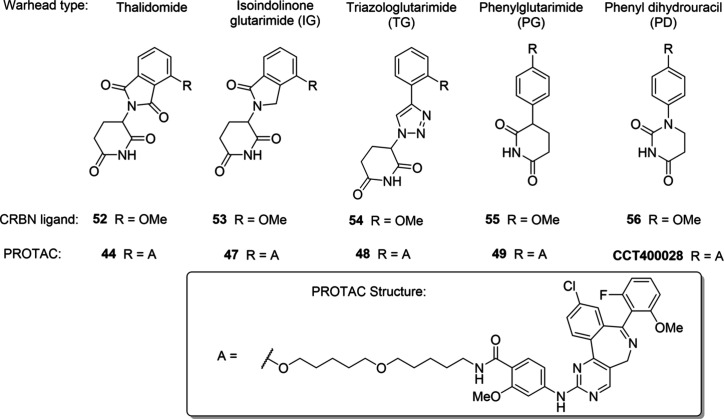
Structures of CRBN Ligands and Aurora A PROTACs, along with Data for CRBN FP, Aurora A HiBiT, and Hydrolytic Stability Assays

**CRBN ligand**		**PROTAC**			
**Compd**	**CRBN FP IC**_**50**_ **(nM)** ^a^	**Cmpd**	**Aurora A HiBiT** ^b^		**Hydrolytic stability** *t*_**1/2**_ **(h)** ^c^
			**DC**_**50**_ **(nM)**	*D*_**max**_ **(%)**	
**52**	710	**44**	37	63	46
**53**	200	**47**	18	55	57
**54**	230	**48**	48	79	29
**55**	1100	**49**	48	75	>120
**56**	6300	**CCT400028**	29	85	>120

a IC_50_ values for parent CRBN ligands **52**–**56** measured in the CRBN FP assay. Data represent the geometric mean of at least three biological repeats. See Supporting Information Table S3 for full statistics.

b Aurora A HiBiT in MV4-11 human pediatric leukemia cells with 6 h PROTAC treatment. DC_50_ data represents the geometric mean of at least three biological repeats. *D*_max_ data represents the mean of at least three biological repeats. See Supporting Information Table S1 for full statistics.

c Hydrolytic stability measured in PBS buffer (containing 10% DMSO) at pH 7.4.

**Figure 5:**
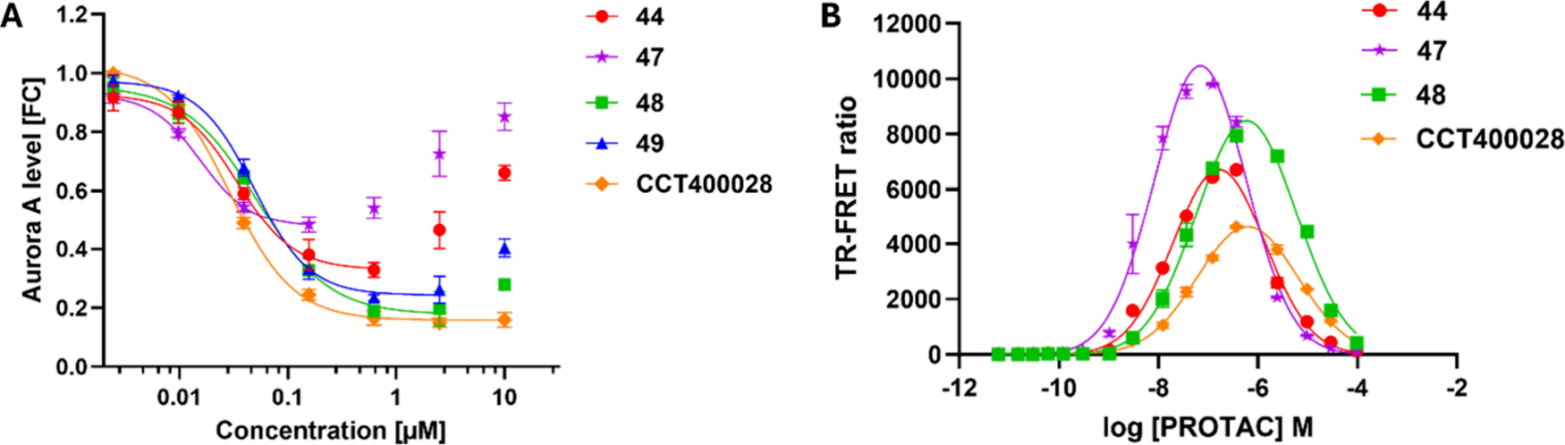
(A) Representative Aurora A-HiBiT assay curves comparing effect on Aurora A protein levels for compounds from Table 3 in MV4-11 human pediatric leukemia cells (6 h PROTAC treatment). Each point represents the mean ± SEM, *n* = 3. (B) Representative Aurora A/CRBN TR-FRET assay curves comparing ternary complex formation for compounds from Table 4 at varying PROTAC concentrations. Each point represents the mean ± SEM, *n* = 3. **44** (red), **47** (purple), **48** (green), **49** (blue), and **CCT400028** (orange).

### Effect of Varying the CRBN Warhead on Hydrolytic Stability and Aurora A Degradation Activity

In the hydrolytic stability assay, TG-containing **48** had a shorter half-life (*t*_1/2_ 29 h) than the phthalimide-containing parent compound **44** (Table 3 and Figure S2). It has been reported in the literature that PG warheads can offer higher hydrolytic stability compared to traditional CRBN ligands and this was borne out with **49** (*t*_1/2_ > 120 h).^50^ PD-containing **CCT400028** was also completely stable in the hydrolytic stability assay (*t*_1/2_ > 120 h). In the Aurora A HiBiT assay, PROTACs **44**–**49** all induced potent degradation of Aurora A (DC_50_ 18–48 nM; Table 3 and Table S1). A varying degree of hook effect was observed (Figure 5A), resulting in a range of *D*_max_ values (from 55% for **47** to 85% for **CCT400028**, Table 3). Interestingly, an increase in hook effect was observed for **47**, which was derived from the most potent CRBN ligand (**53**, CRBN FP IC_50_ = 200 nM), leading to the smallest measured *D*_max_ value (Table 3). In contrast, the hook effect was no longer observed for **CCT400028**, which was derived from the least potent CRBN ligand (**56**, CRBN FP IC_50_ = 6300 nM), leading to the largest measured *D*_max_. These data suggest that even with the small number of analogues tested, complete removal of the hook effect and increase in *D*_max_ could be achieved by decreasing affinity for CRBN (increasing *K*_*E1*_) as hypothesized. A reduction in hook effect was also observed for **48** and **49**.

### Increasing *K*_*E*1_ Increases Cooperativity (α) and Reduces the Hook Effect

The ability of PROTACs **44**, **47**, **48**, and **CCT400028** to form Aurora A/PROTAC/CRBN ternary complexes was assessed in the TR- FRET assay (Figure 5B and Table S2). Han^38^ describes the effects on ternary complex profile upon changing *K*_*P*1_ affinity while α remains constant and upon changing α while *K*_*E*1_ and *K*_*P*1_ remain constant. However, the effects on ternary complex profile when α varies as a result of *K*_*E*1_ or *K*_*P*1_ changing are not described and are less straightforward to interpret. This is because decreasing *K*_*E*1_ would result in a right shift of the Gaussian curve and a reduction in amplitude, which would be partially offset by the increase in α. As described in the previous section, *K*_*P*1_ values were determined by Eurofins (Aurora A KdELECT assay; Table 4 and Figure S4) and used to calculate corresponding *K*_*E*1_ values (Table 4 and Figure 4B eq 1). The extLSS method was applied to the equilibrium dissociation constants (*K*_*E*1_ and *K*_*P*1_) and TR-FRET data for **44**, **47**, **48**, and **CCT400028** to determine estimated values for α (Table 4).

**Table 4:** Key Parameters Describing Ternary Complex Equilibria for PROTACs **44**, **47**, **48**, and **CCT400028**

**Compd**	**E**C_**max**_ **(nM)** ^a^	*K*_*P* **1**_ **(nM)** ^b^	*K*_*E* **1**_ **(nM)** ^c^	**α** ^d^	*K*_*P* **2**_ **(nM)** ^e^	*K*_*E* **2**_ **(nM)** ^e^
**44**	170	50	550	190	0.27	3.0
**47**	70	48	100	170	0.28	0.60
**48**	590	140	2500	1200	0.12	2.1
**CCT400028**	620	71	5400	650	0.11	8.3

a EC_max_ was determined from the TR-FRET profile (Figure 3D and Table S2).

b *K*_*P*1_ was determined by Eurofins (Aurora A KdELECT binding assay, Figure S4) and are mean of *n* = 2.

c *K*_*E*1_ was calculated using eq 1.

d Cooperativity (α) was estimated using the extLSS method.

e *K*_*P*2_ and *K*_*E*2_ were calculated using eq 2.^38^

For all PROTACs, the measured and simulated TR-FRET profiles again showed excellent agreement (Figure S5B,D). Using eq 2, values for *K*_*E*2_ and *K*_*P*2_ were calculated from the estimated α values and the corresponding *K*_*E*1_ and *K*_*P*1_ values (Table 4). Changing from the thalidomide warhead in **44** (CRBN FP IC_50_ = 710 nM) to an isoindoline warhead in **47** (CRBN FP IC_50_ = 200 nM) resulted in a 5.5-fold decrease in *K*_*E*1_ to 100 nM (Table 4). This decrease in *K*_*E*1_ was matched by an equivalent decrease in *K*_*E*2_ to 0.6 nM, resulting in no overall change in α; *K*_*P*1_ and *K*_*P*2_ remained constant. In the Aurora A HiBiT assay, **47** exhibited a strong hook effect (Figure 5A). The increase in hook effect is likely a result of decreased *K*_*E1*_ (more potent binary binding interaction with CRBN), meaning that binary binding to CRBN outcompetes formation of the ternary complex at lower concentrations than for **44**. The opposite effect was observed for **CCT400028**, which contains a PD warhead (CRBN FP IC_50_ = 6300 nM) and had no measurable hook effect (Figure 5A). In comparison to **44**, **CCT400028** has a 10-fold higher *K*_*E*1_ value and 3.5-fold higher α, indicating an increase in cooperativity as a result of reduced affinity for binary-binding interaction with CRBN (Table 4). Our dual design hypothesis of increasing α and *K*_*E*1_ to reduce the hook effect is supported by these data. Interestingly, **48**, which contained a more potent TG CRBN warhead than **44** (CRBN FP IC_50_ = 230 and 710 nM, respectively), had a higher *K*_*E*1_ value (*K*_*E*1_ = 2500 nM; Table 4). The decrease in affinity of **48** for CRBN is possibly a result of a suboptimal linker structure or attachment vector for binary complex formation. Compared to **44**, **48** had a 5-fold increase in *K*_*E*1_ while *K*_*E*2_ remained constant (2.1 nM), resulting in a 6-fold increase in α (1200). The increase in α and *K*_*E*1_ was reflected in the HiBiT assay data, where **48** showed a significant reduction in hook effect compared to **44** (Figure 5A).

### Combining Piperazine Linker and Dihydrouracil Warhead Gives a Potent and Hydrolytically Stable PROTAC

In Table 1, we showed that switching from an alkyl ether linker (**44**) to a piperazine linker (**46**) resulted in a 6-fold improvement in DC_50_. To further increase the potency of **CCT400028**, we replaced the alkyl ether linker with a piperazine linker to give **50**. We expected that due to its hydrolytically stable dihydrouracil CRBN warhead **50** would not be vulnerable to the intramolecularly catalyzed hydrolysis observed for **46**. As predicted, the stability to hydrolysis observed for **CCT400028** was unaffected by the introduction of the piperazine group with a half-life >120 h for **50** (Table 5 and Figure S2). In the Aurora A-HiBiT assay, **50** had DC_50_ and *D*_max_ values of 8 nM and 74%, respectively (comparable to **46**), and with no apparent hook effect at concentrations up to 10 μM (Figure 6, Table 5, and Table S1). Based on their potency in the HiBiT assay and their stability to hydrolysis, **CCT400028** and **50** were selected for further testing including: Aurora A degradation in additional cell lines, effect on cell viability, and proteomics and kinase selectivity profiling.

**Table 5:**
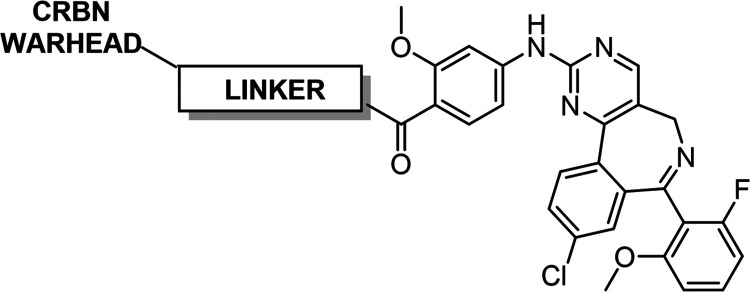

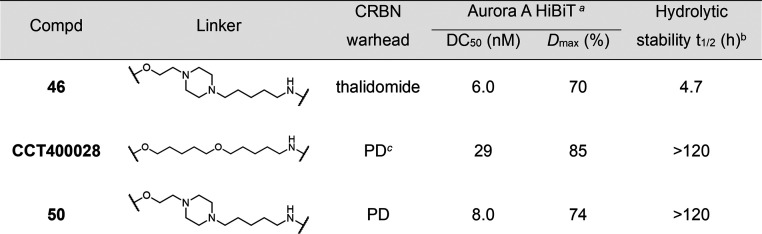
Structures of Aurora A PROTACs **46**, **50**, and **CCT400028**, along with Data for Aurora A HiBiT and Hydrolytic Stability Assays

a Aurora A HiBiT in human pediatric leukemia cells MV4-11 with 6 h of PROTAC treatment. DC_50_ data represent the geometric mean of at least three biological repeats. *D*_max_ data represent the mean of at least three biological repeats. See Supporting Information Table S1 for full statistics.

b Hydrolytic stability measured in PBS buffer (containing 10% DMSO) at pH 7.4.

c PD is phenyl dihydrouracil.

**Figure 6:**
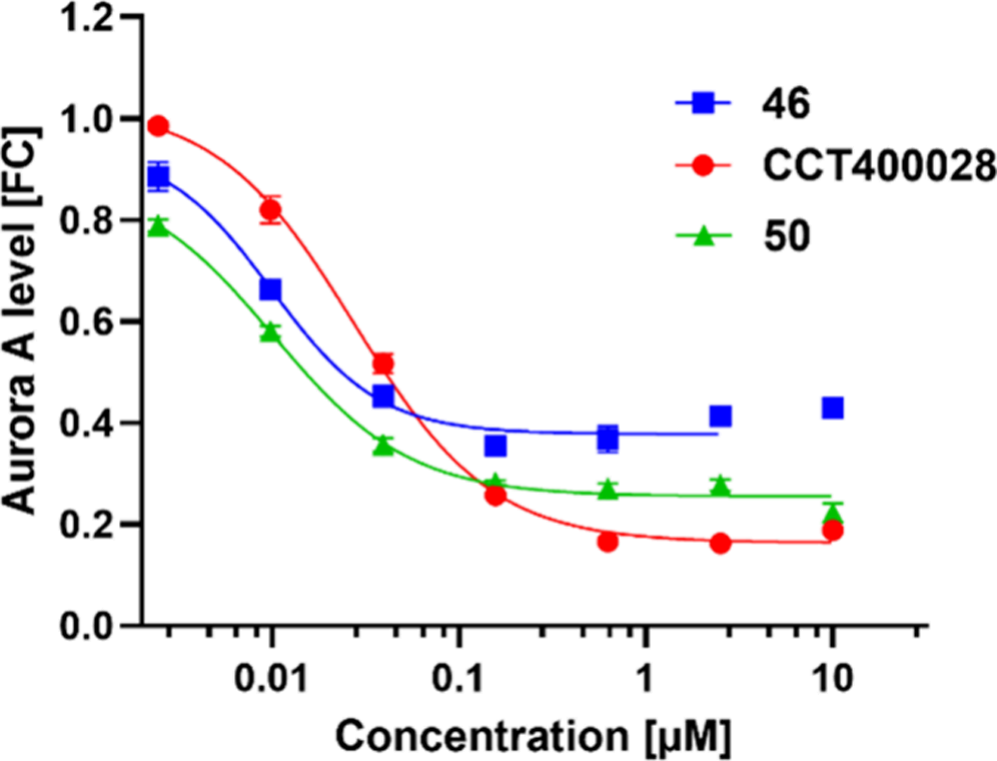
Representative Aurora A-HiBiT assay curves comparing effect on Aurora A protein levels for **46** (blue), **CCT400028** (red), and **50** (green) in MV4-11 human pediatric leukemia cells (6 h PROTAC treatment). Each point represents the mean ± SEM, *n* = 3.

### Effect on Cell Viability

Cell viability was assessed using an alamarBlue assay in MV4-11 leukemia cells after treatment with **JB170**, **CCT400028**, and **50** for 72 h (Figure S7A,B). All three PROTACs caused significant reduction in viability, consistent with previous reports that treatment with Aurora A PROTACs leads to S phase arrest and subsequent apoptosis.^27^ **JB170** was the least active of the three PROTACs tested (GI_50_ = 1100 nM). **CCT400028** and **50** were >3.5-fold and >8-fold more potent than **JB170**, respectively (**CCT400028** GI_50_ = 310 nM, **50** GI_50_ = 130 nM).

### Aurora A Degradation Activity in Neuroblastoma Cells

We next looked to evaluate the Aurora A degradation activity of our PROTACs in a neuroblastoma cell line as it has previously been reported that Aurora A can stabilize N-Myc in neuroblastoma.^14,15^ In Kelly human pediatric neuroblastoma cells, both **CCT400028** and **50** depleted Aurora A to lower levels than **JB170** by Western blot analysis (Figure 7 and Figure S8). Likely due to the observed warhead instability for **JB170**, Aurora A depletion was less efficient at 24 h than at 4 h. The same effect did not occur when cells were treated with our more stable, phenyl dihydrouracil-containing **CCT400028** and **50**. A hook effect was observed for **50** at 10 μM but was not observed for **CCT400028**.

**Figure 7:**
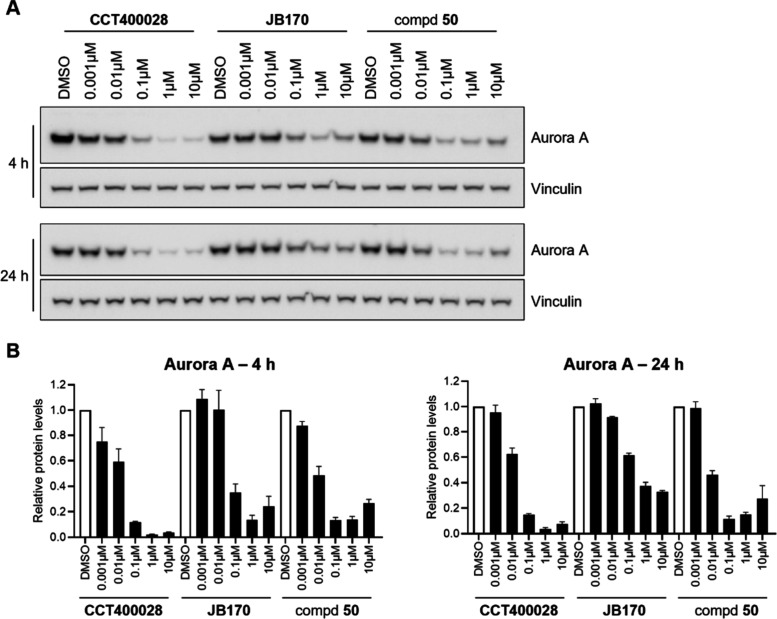
Aurora A depletion in Kelly human pediatric neuroblastoma cells. (A) Kelly cells were treated with different concentrations of **JB170**, **CCT400028**, and **50** for 4 and 24 h. Whole cell lysates were analyzed by immunoblotting for Aurora A and vinculin as the loading control (representative images of three independent experiments). (B) Quantification of (A) (*n* = 3 for all, except *n* = 2 for 0.001 and 0.01 μM **JB170**).

Proteomics profiling of **CCT400028** and **50** in Kelly neuroblastoma cells (Figure 8) showed excellent selectivity for depletion of Aurora A, with the extent of Aurora A depletion (Log2Ratio) greater than that of **JB170** for **CCT400028** and slightly lower than that of **JB170** for **50**. For all three PROTACs, we did not observe statistically significant depletion of N-Myc, Aurora B, or transcriptional and translational regulators, including CDK9 and GSPT1 (Figure 8 and Figure S8B).

**Figure 8:**
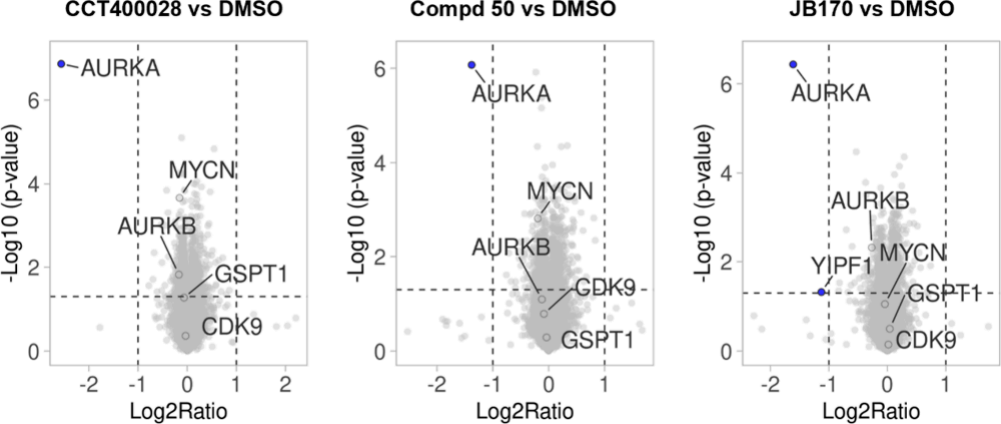
Proteomics profiling of **CCT400028**, **50**, and **JB170** in Kelly human pediatric neuroblastoma cells. Volcano plots showing changes in total protein abundance in whole cell lysates of Kelly cells treated with 1 μM of compound for 4 h. The *x*-axis displays the relative abundance of proteins in compound versus DMSO treated cells (log2 fold change). The *y*-axis displays the *p*-value (−log10) from three independent experiments (paired *t* test). Dashed lines indicate cutoffs (*p*-value <0.05, absolute log2FC >1). Protein identifiers are shown using UniProt gene names (MYCN = N-Myc, AURKA = Aurora A, AURKB = Aurora B).

### Kinase Selectivity Profiling for **50** and **CCT400028**

To evaluate kinase binding selectivity, **CCT400028** and **50** were profiled in the Eurofins *scan*EDGE kinase binding assay, which contains 97 kinases distributed across the kinome (Figure 9A and Table S4).^58^ **CCT400028** showed excellent binding selectivity against the 97 kinase panel with Aurora A the only kinase that **CCT400028** bound to potently (<35% control binding remaining) at 1 μM. Under the same conditions, **50** showed poorer selectivity as it bound to 10/97 of the tested kinases with <35% control binding remaining, including Aurora A and B. For both compounds, the binding affinity (*K*_D_) for Aurora A, B, and C was also determined at Eurofins (Figure 9B and Figure S9). Within the Aurora kinase family, **CCT400028** showed high selectivity for Aurora A with no measurable affinity for Aurora C and a 28-fold selectivity over Aurora B. **CCT400028** would therefore not be expected to affect Aurora B activity in cells due to its weak affinity for Aurora B (2100 nM) and inability to induce Aurora B degradation, as shown by both proteomics (Figure 8) and Western blot analysis (Figure S8A). **50** bound more potently to all Aurora family members compared to **CCT400028** but retained 15-fold selectivity for Aurora A over Aurora B and >1000-fold selectivity over Aurora C.

**Figure 9:**
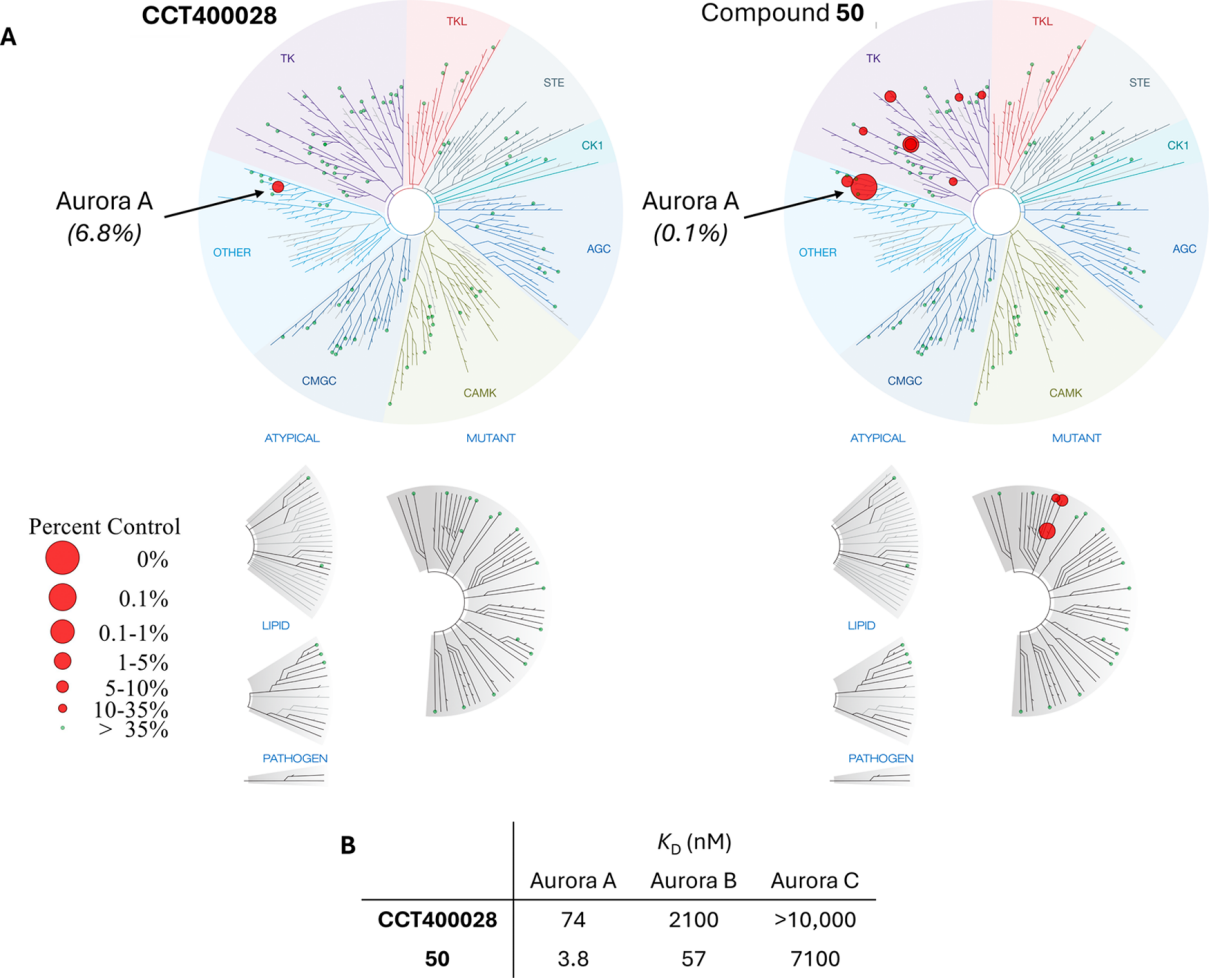
Kinase selectivity profiling for **CCT400028** and **50** tested at 1 μM. (A) Kinome profiling in Eurofins *scan*EDGE 97 Kinase Panel binding assay (Eurofins DiscoverX, LLC (San Diego)). (B) Binding affinity (*K*_D_) determination against Aurora A/B/C in Eurofins KdELECT binding assay (Eurofins DiscoverX, LLC (San Diego)). *K*_D_ values are mean of two biological repeats.

### Degradation of Aurora A by **CCT400028** is CRBN Mediated

In Kelly neuroblastoma cells, **CCT400028** gave superior depletion of Aurora A at 4 and 24 h compared to **50** (Figure 7) and exhibited greater selectivity against a panel of 97 kinases (Figure 9A and Table S4). **CCT400028** was therefore selected for further profiling to ensure the degradation activity was mediated through an on-target mechanism of action. Rescue experiments were carried out with **MG132** (proteosome inhibitor), **MLN4924** (neddylation inhibitor), **CC220** (competitive CRBN ligand), and **alisertib** (competitive Aurora A ligand) (Figure 10).^59^ **MG132** and **MLN4924** both prevented Aurora A depletion, confirming that the depletion was proteasome and Cullin ring ligase dependent, respectively. Prevention of Aurora A depletion by competition with **CC220** and **alisertib** confirmed that the depletion was dependent on binding to CRBN and Aurora A, respectively. In addition, a CRBN-inactive *N*-methylated version of **CCT400028** (**57**) was unable to reduce Aurora A protein levels at concentrations up to 10 μM in Kelly neuroblastoma cells (Figure S10). The above data validated that the degradation of Aurora A by **CCT400028** was on-target and on-mechanism. CRBN-mediated Aurora A degradation was also confirmed in KNS-42 human pediatric glioma cells. **CCT400028** showed improved Aurora A depletion compared to **JB170** and activity consistent with that seen in MV4-11 leukemia cells and Kelly neuroblastoma cells (Figure S11A,B). Rescue experiments confirmed that the degradation of Aurora A is also on-target and on-mechanism in this cell line (Figure S11C,D). Altogether, our data confirm effective CRBN-mediated Aurora A degradation by **CCT400028** in three different human pediatric cancer cell lines from distinct disease types.

**Figure 10:**
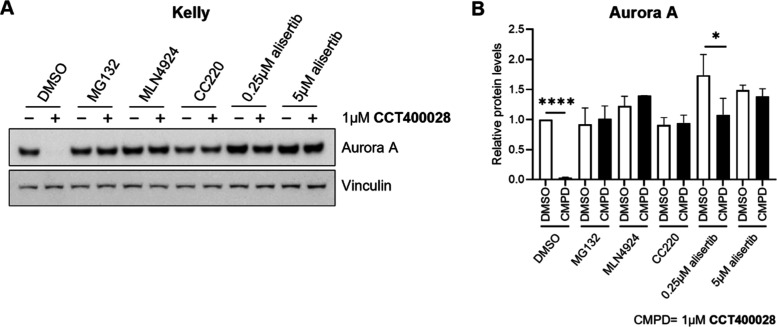
**CCT400028** induces proteasomal degradation of Aurora A via CRBN-mediated ubiquitination in Kelly human pediatric neuroblastoma cells. (A) Kelly cells were pretreated with **MG132** (10 μM), **MLN4924** (1 μM), **CC220** (10 μM,) and 0.25 or 5 μM **alisertib** for 2 h. This was followed by a combined treatment ± 1 μM **CCT400028** for 4 h. Whole-cell lysates were analyzed by immunoblotting for Aurora A and vinculin as loading control (representative images of three independent experiments). (B) Quantification of (A) (*n* = 3, *p < 0.05, ****p < 0.0001, paired *t* test).

### **CCT400028** Fulfills Criteria for an *In Vitro* Aurora A Degrader Chemical Probe

The aim of this work was to develop a versatile PROTAC chemical probe suitable for studying Aurora A degradation in an *in vitro* cellular context. Compared to the commercially available start-point **JB170**, **CCT400028** exhibited superior activity as an Aurora A degrader by HiBiT (MV4-11 leukemia cells; Figure 11A), Western blot (Kelly neuroblastoma cells and KNS-42 glioma cells; Figure 7 and Figure S11, respectively), and proteomics profiling (Kelly neuroblastoma cells; Figure 8). For **CCT400028**, *D*_max_ is not limited by a hook effect and is maintained over >1.6 log units of concentration, expanding the range of concentrations over which maximal degradation can be achieved (Figure 11A). In addition, stability in aqueous buffer was greatly improved for **CCT400028** (*t*_1/2_ > 120 h) compared to **JB170** (*t*_1/2_ 25 h; Figure 11B).

**Figure 11:**
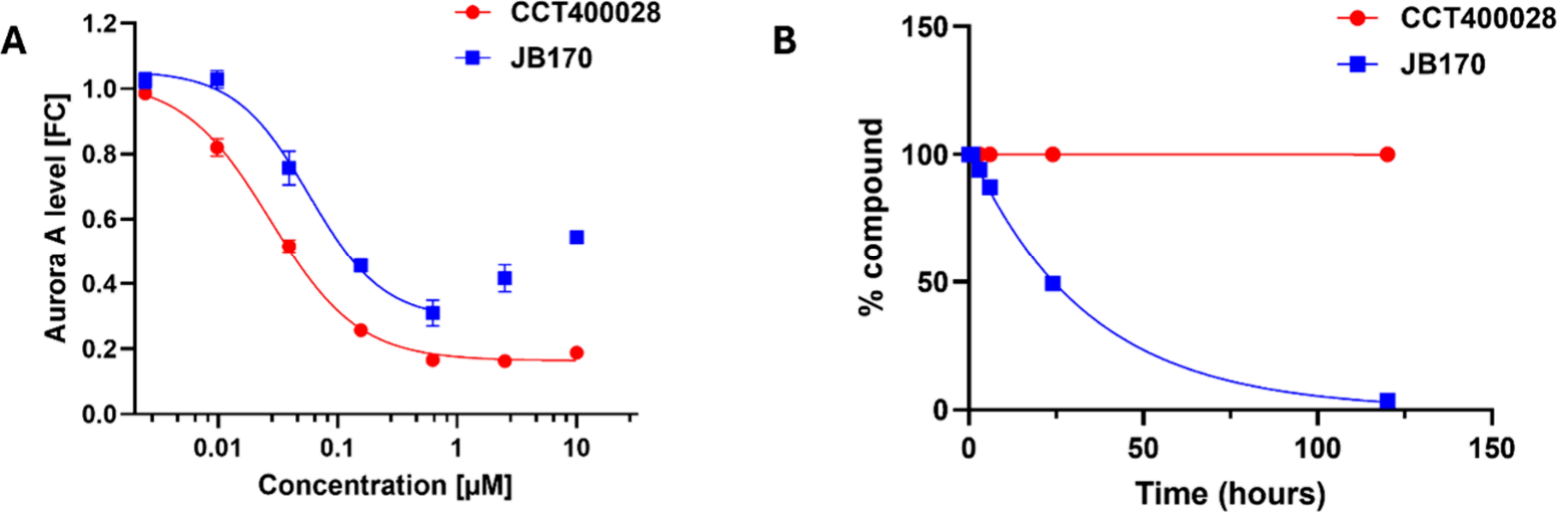
**CCT400028** shows improved Aurora A degradation activity and stability to hydrolysis compared to **JB170**. (A) Representative Aurora A-HiBiT assay curves comparing effect on Aurora A protein levels for **JB170** (blue) and **CCT400028** (red) in MV4-11 human pediatric leukemia cells (6 h PROTAC treatment). Each point represents the mean ± SEM, *n* = 3. (B) Analysis of stability in PBS buffer (37 °C) for 1 μM **JB170** (blue) and **CCT400028** (red).

In 2023, Hartung et al.^59^ set out quality criteria for degrader chemical probes: (1) DC_50_ < 1 μM; (2) *D*_max_ > 80%; (3) > 30-fold selectivity to neighbors and unbiased proteomics profiling; (4) proof of on-target and on-E3 activity; (5) >100-fold reduction in potency against POI for matched inactive; (6) sufficient stability. **CCT400028** demonstrates good Aurora A degradation (DC_50_ 29 nM, *D*_max_ 85%) and excellent selectivity in proteomic and kinase selectivity profiling (Figures 8 and 9, respectively).^58^ Proof of on-target and on-E3 ligase activity was demonstrated in Kelly neuroblastoma and KNS-42 glioma cells (Figure 10 and Figure S11, respectively), and a matched inactive control analogue (**57**) was unable to reduce Aurora A protein levels at concentrations up to 10 μM (>300-fold above **CCT400028** DC_50_; Figure S10). In addition, **CCT400028** exhibited excellent hydrolytic stability (half-life >120 h). **CCT400028** therefore fulfills all of the Hartung et al.^59^ best-practice criteria for a degrader chemical probe (Table 6).

**Table 6:** CCT400028 Fulfills Hartung *et al.* ^59^ Degrader Chemical Probe Criteria

Quality criteria	Degrader chemical probe	CCT400028
Potency	DC_50_ < 1 μM, *D*_max_ > 80%	DC_50_ 29 nM, *D*_max_ 85%
Selectivity	>30-fold selectivity to neighbors and unbiased proteomics profiling	selective for Aurora A in proteomics and kinase panel profiling, 28-fold selective over Aurora B, > 135-fold selective over Aurora C
Target engagement	proof of on-target and on-E3 activity	CRBN-mediated Aurora A degradation confirmed
Chemical matter	derisk potentially promiscuous or PAINs motifs	none present in molecule
Control compounds	>100-fold reduction in potency against POI for matched inactive	inactive control compound **57** does not degrade Aurora A at concentrations ≤10 μM
Stability and reactivity	avoid fast deterioration of structural integrity	hydrolytic *t*_1/2_ > 120 h

## Conclusions

In this study, we report the optimization of **JB170**, a commercially available Aurora A PROTAC, to **CCT400028**, a second-generation alisertib-derived degrader for use as an *in vitro* chemical probe. By removing the amide moiety and oxygen atoms from the linker of **JB170**, an increase in hydrolytic stability was observed without compromising the Aurora A degradation potency and efficiency, as measured by an Aurora A HiBiT assay. An Aurora A/CRBN TR-FRET assay was used to measure ternary complex formation and dissociation, which informed the rational optimization of the CRBN-targeting warhead to remove the hook effect. This was achieved by decreasing affinity of the binary-binding interaction with CRBN (increasing *K*_*E*1_) without impacting the ternary-binding interaction (*K*_*E*2_) and represents a novel approach to optimizing α (cooperativity). It remains to be seen if this approach is widely applicable to other PROTAC series, protein targets, and E3 ligases.

**CCT400028** exhibits superior activity as a degrader in the HiBiT assay, Western blot analysis, and proteomics profiling compared to **JB170**. Potent Aurora A degradation was shown for **CCT400028** in three different human pediatric tumor cell lines, namely, leukemia (MV4-11), neuroblastoma (Kelly), and glioma (KNS-42). In addition, **CCT400028** has improved stability to hydrolysis, which combined with the absence of hook effect, expands the range of concentrations and duration at which maximal degradation can be achieved. In line with the probe quality criteria set out by Hartung et al., **CCT400028** exhibited sufficient potency (DC_50_ 29 nM, *D*_max_ 85%) and excellent selectivity in proteomic and kinase selectivity profiling.^59^ In addition, proof of on-target and on-E3 ligase activity was demonstrated and a matched inactive control analogue was devoid of Aurora A degradation activity. Based on this profile, we recommend **CCT400028** for use as a degrader chemical probe for studying Aurora A degradation *in vitro* over a concentration range of 30 nM to 1 μM; caution should be used at concentrations between 1 and 10 μM as selectivity profiling was not carried out above 1 μM. **57**, which contains an *N*-methylated CRBN warhead, representing a suitable matched inactive control. Further work is being undertaken to develop an Aurora A PROTAC chemical probe suitable for *in vivo* use.

## Experimental Section

### Materials and Methods (Chemistry)

Reactions were carried out under N_2_ at room temperature unless otherwise stated. All anhydrous solvents and reagents were obtained from commercial suppliers and used without further purification. Where available, compounds were purchased or made according to literature procedure as cited. Compounds **52**, **53**, and **54** were sourced from commercial suppliers. The synthesis and characterization of **JB170** was described in our previously published work.^27^ 3-Aminoglutarimide hydrochloride, 3-bromoglutarimide, and 4-hydroxythalidomide were supplied as racemic mixtures. Evaporation of solvents was carried out using a rotary evaporator under reduced pressure at a bath temperature of up to 50 °C, or a Biotage V-10 Touch Evaporation System using preset methods. Flash column chromatography was carried out using a Biotage purification system using prepacked SNAP KP-Sil or Sfär Silica HC D cartridges or on an Isco purification system using prepacked RediSep Rf cartridges or on the reverse-phase mode using SNAP Ultra C18 or Sfär C18D cartridges. Microwave-assisted reactions were carried out using a Biotage initiator microwave system. Final compounds were purified to ≥95% purity by HPLC analysis. NMR data were collected on a Bruker AVANCE 500 spectrometer equipped with a 5 mm BBO/QNP probe or on a Bruker AVANCE Neo 600 spectrometer equipped with a 5 mm TCI Cryo-Probe. NMR data are presented in the form of chemical shift δ (multiplicity, coupling constants, integration) for major diagnostic protons, given in parts per million relative to tetramethylsilane, referenced to the internal deuterated solvent. High-resolution mass spectrometry (HRMS) was assessed using an Agilent 1200 series HPLC and a diode array detector coupled to a 6120 time of a flight mass spectrometer with a dual multimode APCI/ESI source or on a Waters Acquity UHPLC and a diode array detector coupled to a Waters G2 QToF mass spectrometer fitted with a multimode ESI/APCI source.

### Synthesis of PROTAC Compounds

PROTACs were synthesized according to the synthetic routes shown in Scheme 1. Full synthetic routes, procedures, and characterization for synthetic intermediates and final products can be found in Supporting Information Section 2.2.

## Supplementary Material







## Notes

Additional figures, tables, and scheme; additional experimental details; synthetic procedures; and ^1^H and C NMR spectra and HPLC traces for final compounds (PDF)
Proteomics profiling data for **CCT400028**, **50**, and **JB170** (XLSX)
Molecular string files for all final compounds (CSV)

## Abbreviations

AURKA: aurora kinase A
AURKB: aurora kinase B
Bn: benzyl
Boc: *tert*-butyloxy carbonyl
CDK9: cyclin-dependent kinase 9
cmpd: compound
CRBN: Cereblon E3 ligase
DC_50_: half-maximal degradation concentration
DIPEA: *N*,*N*-diisopropylethylamine
*D*_max_: maximal amount of degradation
DMB: 2,4-dimethoxybenzyl
DMEDA: *N*,*N*′-dimethylethylenediamine
DMSO: dimethyl sulfoxide
dppf: 1,1′-bis­(diphenylphosphino)­ferrocene
Et_3_N: triethylamine
EtOH: ethanol
FP: fluorescence polarization
GSPT1: G1 to S phase transition 1
HBTU: hexafluorophosphate benzotriazole tetramethyl uranium
MD: molecular dynamics
MeCN: acetonitrile
MeOH: methanol
NMM: *N*-methylmorpholine
PBS: phosphate-buffered saline
PEG: polyethylene glycol
Ph: phenyl
pin: pinacol
POI: protein of interest
PROTAC: proteolysis-targeting chimera
SEM: standard error of the mean
*t*_1/2_: half-life
TfOH: trifluoromethanesulfonic acid
THF: tetrahydrofuran
TMS: trimethylsilyl
TR-FRET: time-resolved fluorescence resonance energy transfer
VHL: Von Hippel-Lindau tumor suppressor
YIPF-1: Yip1 domain family member 1
