# Supplementary material for: Unhooking the Hook: Optimization of the Aurora A Targeting PROTAC JB170 to CCT400028, an In Vitro Degrader Chemical Probe
Source: J Med Chem. 2026 Jan 12;69(2):1552–67. doi: 10.1021/acs.jmedchem.5c03024 (PMC12833858; doi:10.1021/acs.jmedchem.5c03024)
Supplement: Supplementary file 1 [file jm5c03024_si_001.pdf]

Supporting Information

Unhooking the Hook: Optimisation of the Aurora A Targeting PROTAC JB170 to CCT400028, an *In Vitro* Degrader Chemical Probe

Jack A. O'Hanlon,[a,b] Katrin Gutsche,[a,$] Juliane Elisabeth Müller,[c,$] Nihar Ranjan Prusty,[a] Amin Mirza,[a] Theodoros I. Roumeliotis,[d] Hengzhang Yang,[a] Mark Stubbs,[a] Stephen T. Hallett,[a] Lorenz Eing,[e] Peter Craig McAndrew,[a] Jyoti Sharma Choudhary,[c] Yann-Vaï Le Bihan,[a,f] John Caldwell,[a] Rob L. M. van Montfort,[a,f] Paul Workman,[a,b] Elmar Wolf,[c,*], Gary K. Newton [a,b,*] and Lindsay E. Evans [a,b,*]

[a] *Centre for* Cancer *Drug Discovery, The Institute of Cancer Research, London SM2 5NG, U.K*

[b] *Cancer Research UK Children's Brain Tumour Centre of Excellence, The Institute of Cancer Research, London SM2 5NG, U.K*

[c] *Institute of Biochemistry, University of Kiel, Rudolf-Höber-Straße 1, Kiel 24118, Germany*

[d] *Functional Proteomics Group, Institute of Cancer Research, Chester Beatty Labs, 237 Fulham Road, SW3 6JB London, UK*

[e] *Chair of Biochemistry and Molecular Biology, University of Würzburg, Am Hubland, 97074 Würzburg, Germany*

[f] *Division of Structural Biology, The Institute of Cancer Research, London SM2 5NG, U.K.*

[$] these authors contributed equally

* Joint corresponding authors: Lindsay E. Evans (lindsay.evans@icr.ac.uk), Gary K. Newton (gary.newton@icr.ac.uk), and Elmar Wolf (elmar.wolf@biochem.uni-kiel.de)

Table of Contents

1. Supplementary Figures and Tables S2
2. Experimental methods S17
    2.1 Biological methods S17
    2.2 Synthetic methods S25
        2.2.1 General Details S25
        2.2.2 Experimental Procedures S26
3. NMR Spectra of synthesized compounds S86
4. HPLC Traces for synthesized compounds S101
5. References S108

Supplementary Figures, Tables and Scheme

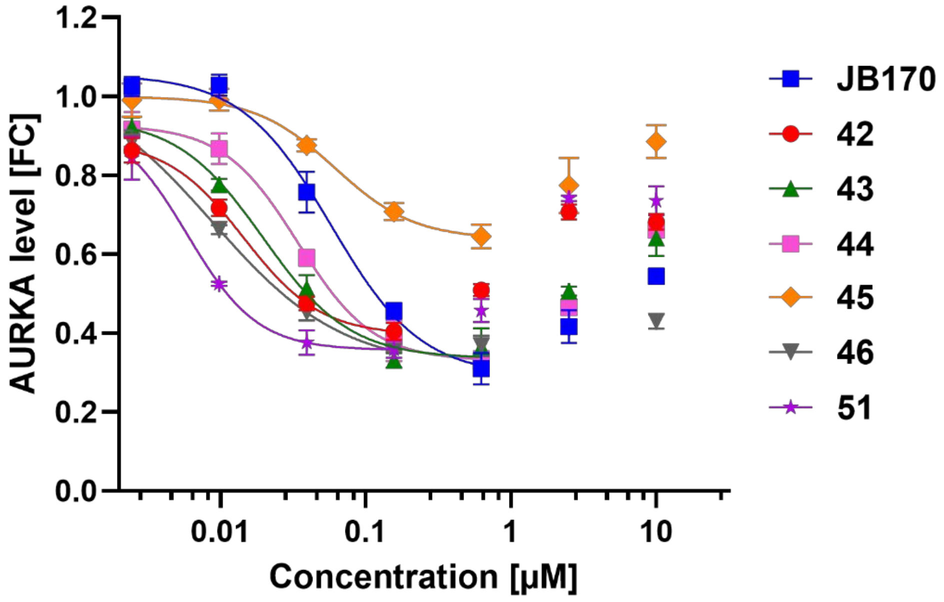

Figure S1. Degradation of Aurora A in MV4-11 human paediatric leukaemia cells after 6 h PROTAC treatment. Representative Aurora A-HiBiT assay curves comparing compounds from Table 1 (main text). **JB170** (blue), **42** (red), **43** (green), **44** (pink), **45** (orange), **46** (grey) and **51** (purple). Each point represents the mean ± SEM, *n* = 3.

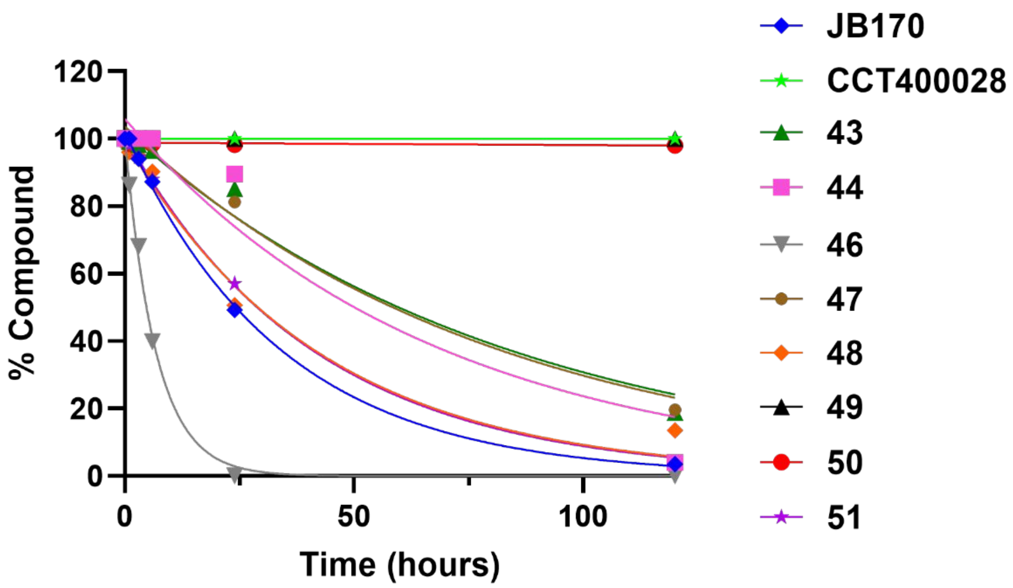

Figure S2. Analysis of hydrolytic stability in PBS buffer at pH 7.4 (37 °C) for 1 µM **JB170** (blue diamond), **CCT400028** (green star), **43** (dark green triangle), **44** (pink square), **46** (grey inverted triangle), **47** (brown circle), **48** (orange diamond), **49** (black triangle), **50** (red circle), **51** (purple star).

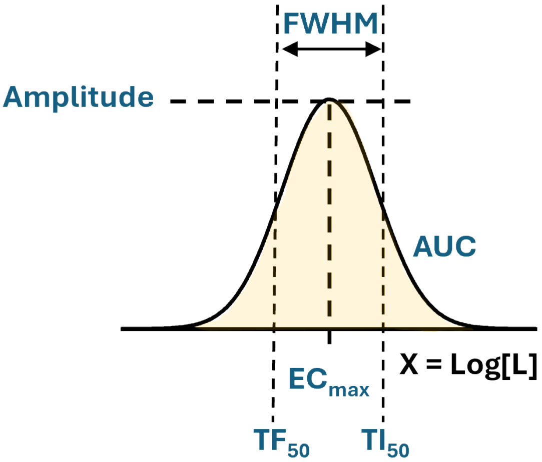

Figure S3. Schematic representation of key ternary complex dose-response curve fitting parameters used in TF-FRET data analysis. Image is adapted from Han.[1] AUC is area under the curve; FWHM is the full width at half-maximal points; ECmax is the maximally effective concentration; TF50 is the ternary complex–forming EC_{50}; TI_{50} is the ternary complex–inhibiting EC50, where EC50 is defined as the half maximal effective concentration.[2]

JB170 replicate 1, K_D = 5.9 nM

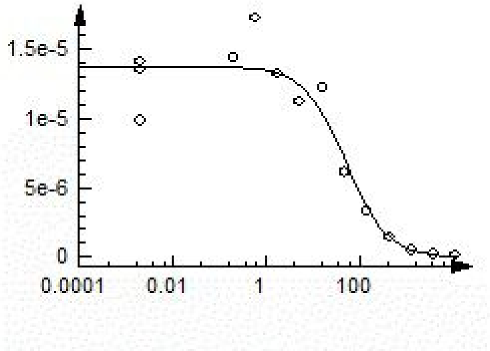

JB170 replicate 2, K_D = 7.9 nM

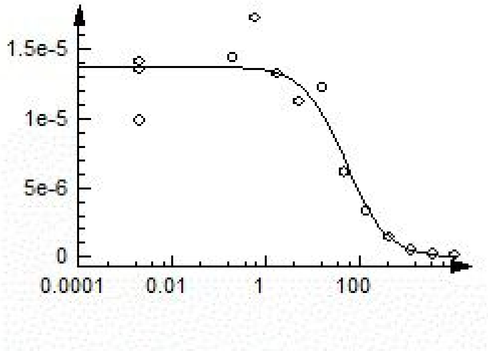

Compd **51** replicate 1, K_D = 3.7 nM

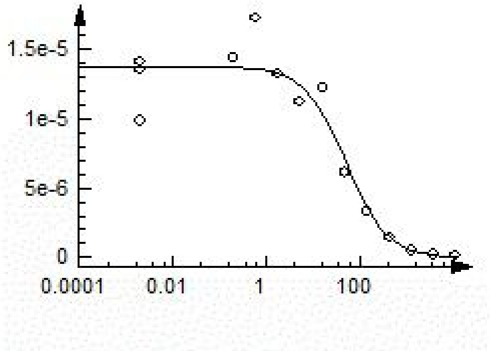

Compd **51** replicate 2, K_D = 2.9 nM

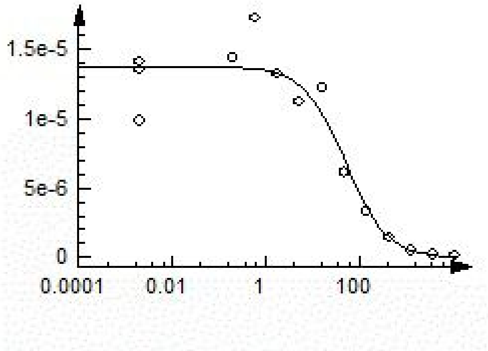

Compd **44** replicate 1, K_D = 49 nM

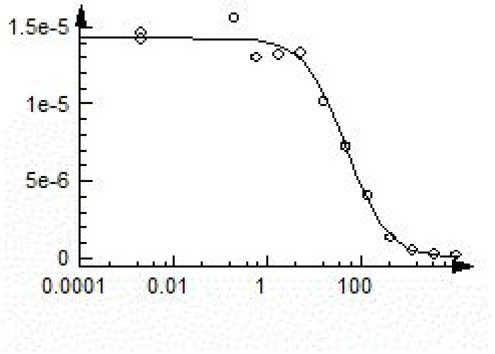

Compd **44** replicate 2, K_D = 51 nM

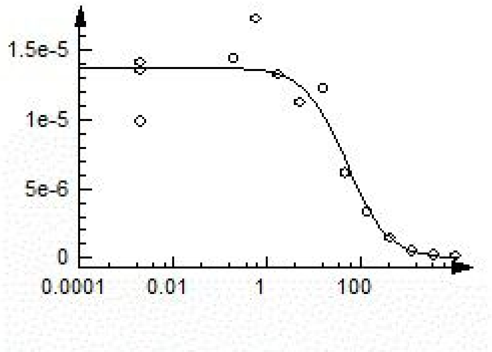

Compd **47** replicate 1, K_D = 48 nM

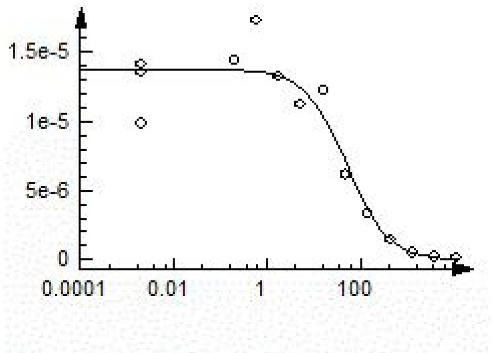

Compd **47** replicate 2, K_D = 49 nM

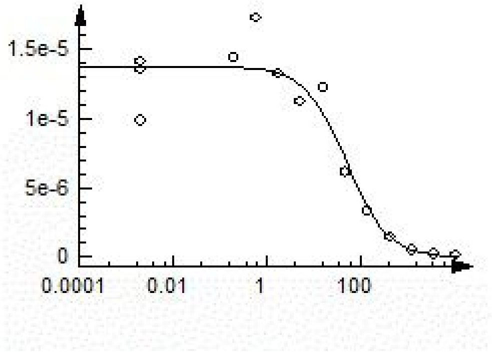

Compd **48** replicate 1, K_D = 160 nM

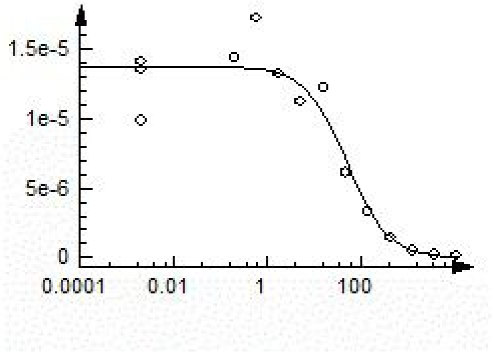

Compd **48** replicate 2, K_D = 120 nM

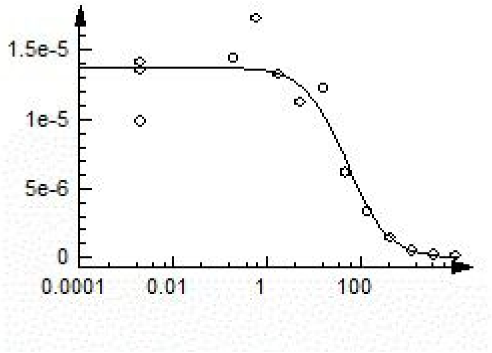

CCT400028, replicate 1, K_D = 55 nM

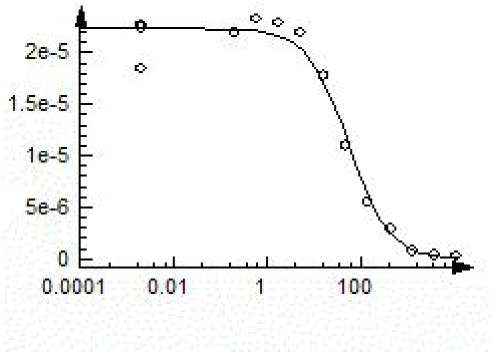

CCT400028, replicate 2, K_D = 92 nM

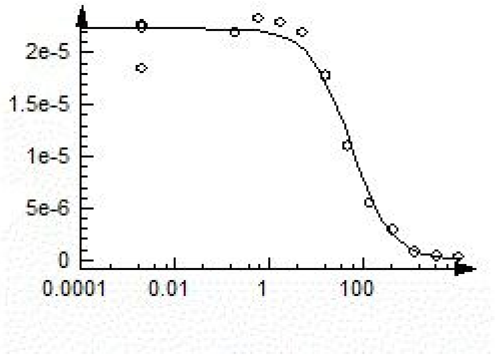

Figure S4. Binding curves and K_D values determined for PROTACs against Aurora-A in Eurofins KdELECT Binding LeadHunter Assay (Eurofins DiscoverX, LLC, San Diego). The amount of kinase measured by qPCR (Signal; y-axis) is plotted against the corresponding compound concentration in nM in log10 scale (x-axis).

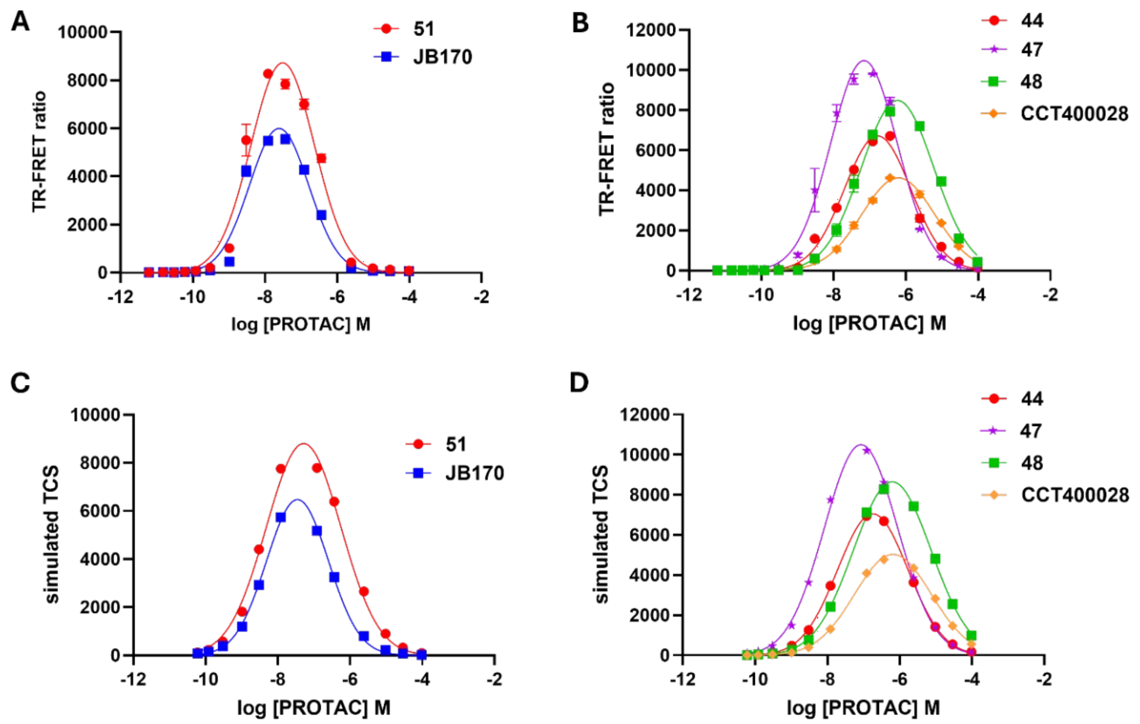

Figure S5. Experimental Aurora-A/CRBN TR-FRET assay curves (A and B) and simulated ternary complex profiles (C and D) comparing ternary-complex formation/dissociation for **51** and **JB170** (A and C), and **44**, **47**, **48** and **CCT400028** (B and D) at varying PROTAC concentrations. For experimental Aurora-A/CRBN TR-FRET profiles (A and B) each point represents the mean ± SEM, n = 3. Simulated ternary complex profiles (C and D) were generated using Han's excel-based program for kinetic simulation of ternary complexes and values of α and β estimated using the extLSS method also reported by Han.[1]

A

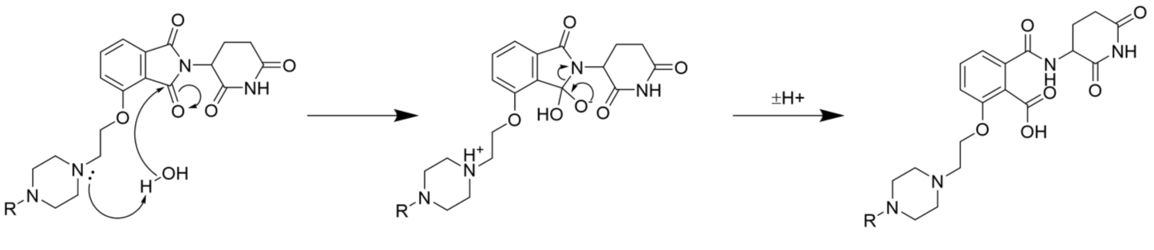

58 59 60

B

Time (hr)	% Cmpd 46[b]	% Single hydrolysis product[c]	% Double hydrolysis product[d]
0	100	0	0
1	86	14	0
3	68	32	0
6	40	54	6
24	0	70	30
120	0	29	71

Figure S6. (A) Proposed mechanism for intramolecular general base catalysed phthalimide hydrolysis; (B) Percentages of compound **46** and the corresponding single and double hydrolysis products detected by LCMS analysis over 120 hr in the hydrolytic stability assay.[a]

[a]Hydrolysis of compound **46** was determined by LC-MS at 0, 1, 3, 6, 24, 120 hr. By 3 hrs 32% of the detected species resulted from a single hydrolysis event with m/z 495.7 ($[M+2H]^{2+}$). Two different products, each representing products of a single hydrolysis event (m/z 495.7 ($[M+2H]^{2+}$)) could be observed: however, the sites of hydrolysis could not be identified from analysis of the fragmentation pattern.

[b]$[M+2H]^{2+}$ = m/z 486.7; [c]$[M+2H]^{2+}$ = m/z 495.7; [d]$[M+2H]^{2+}$ = m/z 504.7.

A

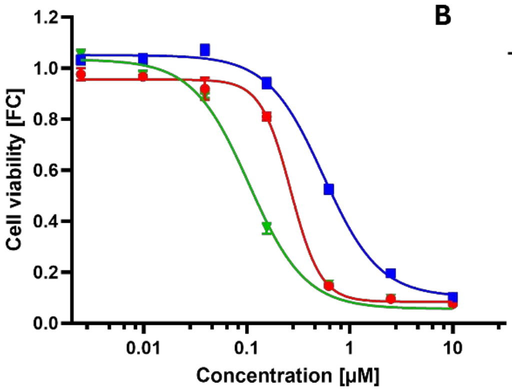

B

	GI_{50} (nM)	pGI_{50} ± SEM
JB170	1100	5.8 ± 0.13
CCT400028	310	6.5 ± 0.05
50	130	6.8 ± 0.03

Figure S7. (A) Representative cell viability dose-response curves comparing **JB170** (blue), **CCT400028** (red) and **50** (green) for MV4-11 paediatric leukaemia cells in an alamarBlue assay (72 h PROTAC treatment). Each point represents the mean ± SEM, *n* = 3 technical repeats. (B) Corresponding GI_{50} values (geometric mean) and pGI50 values (mean ± SEM) for 3 biological repeats.

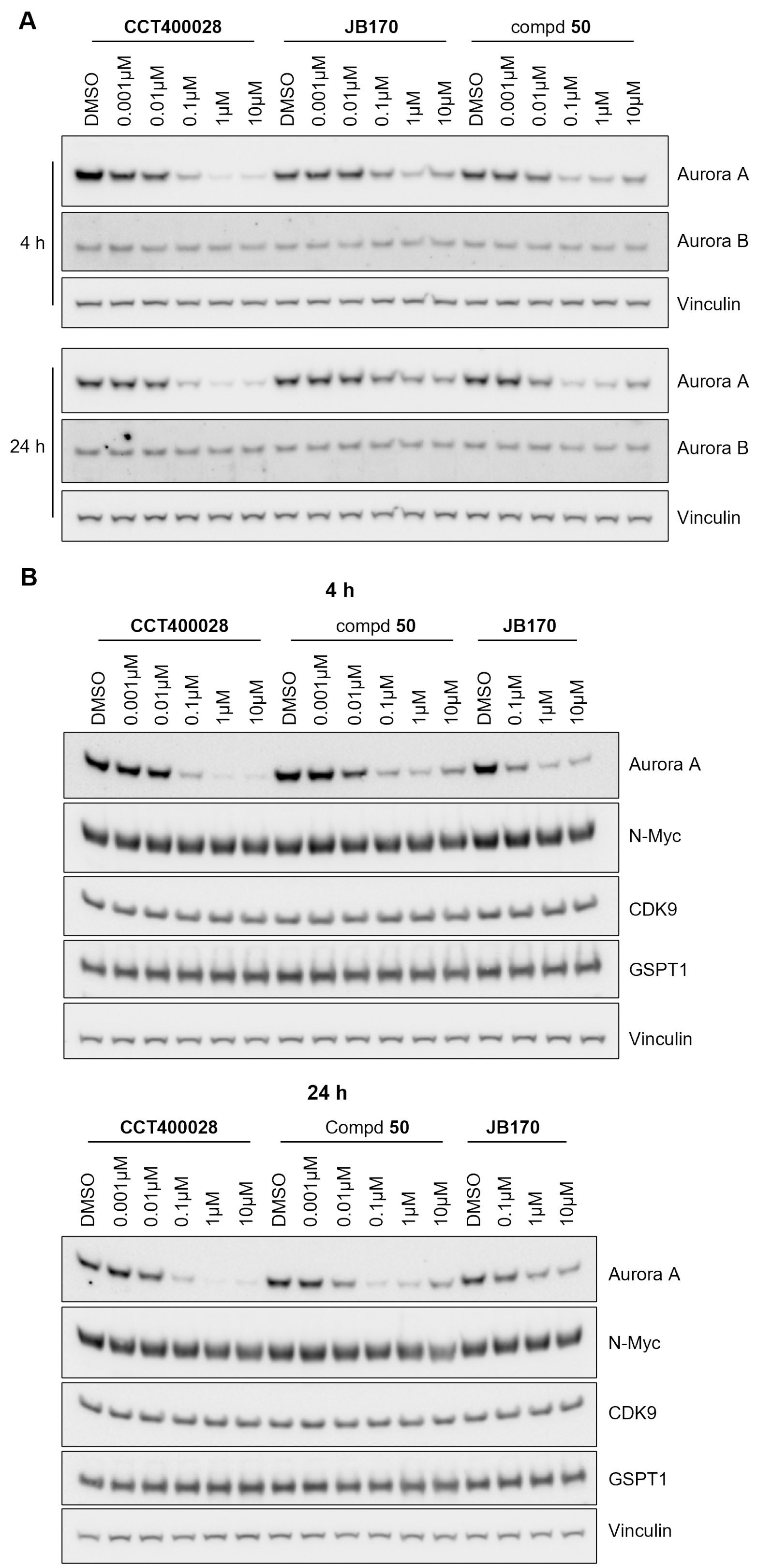

Figure S8. Effect of **JB170**, **CCT400028**, and **50** on Kelly human paediatric neuroblastoma cells. (A) Whole cell lysates were analysed by immunoblotting for Aurora A, Aurora B and vinculin (n = 1, part of the dataset shown in Figure 7). (B) Whole cell lysates were analysed by immunoblotting for Aurora A, N-Myc, CDK9, GSPT1 and vinculin (n=1, part of the dataset shown in Figure 7).

Aurora A

CCT400028, replicate 1 K_D = 55 nM | **CCT400028**, replicate 2 K_D = 92 nM | Compd **50**, replicate 1 K_D = 4.5 nM | Compd **50**, replicate 2 K_D = 3.2 nM

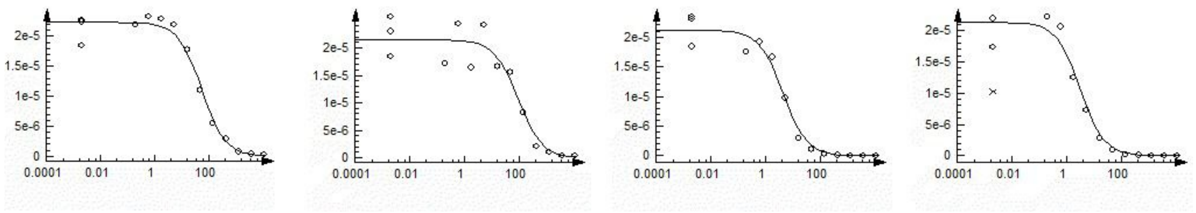

Aurora B

CCT400028, replicate 1 K_D = 1500 nM | **CCT400028**, replicate 2 K_D = 2700 nM | Compd **50**, replicate 1 K_D = 52 nM | Compd **50**, replicate 2 K_D = 62 nM

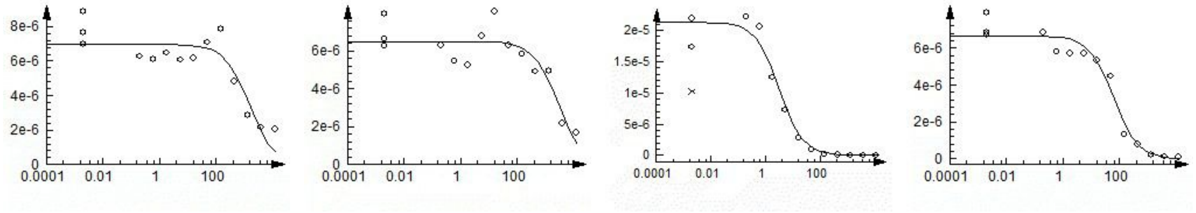

Aurora C

CCT400028, replicate 1 K_D >12000 nM | **CCT400028**, replicate 2 K_D >12000 nM | Compd **50**, replicate 1 K_D = 6800 nM | Compd **50**, replicate 2 K_D = 7400 nM

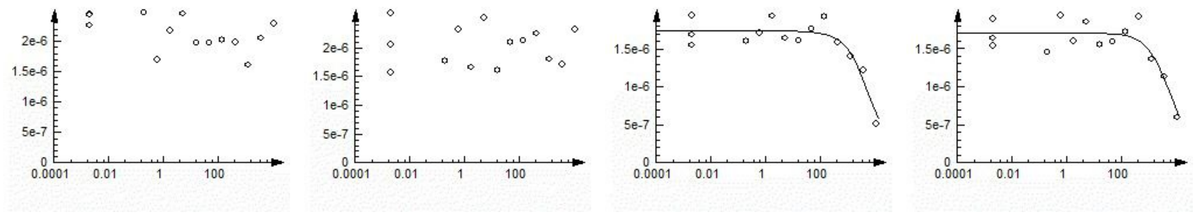

Figure S9. Binding curves and K_D values determined for **CCT400028** and **50** against Aurora-A, B and C in Eurofins KdELECT Binding LeadHunter Assay (Eurofins DiscoverX, LLC (San Diego)). The amount of kinase measured by qPCR (Signal; y-axis) is plotted against the corresponding compound concentration in nM in log10 scale (x-axis). Data points marked with an "x" were not used for K_D determination.

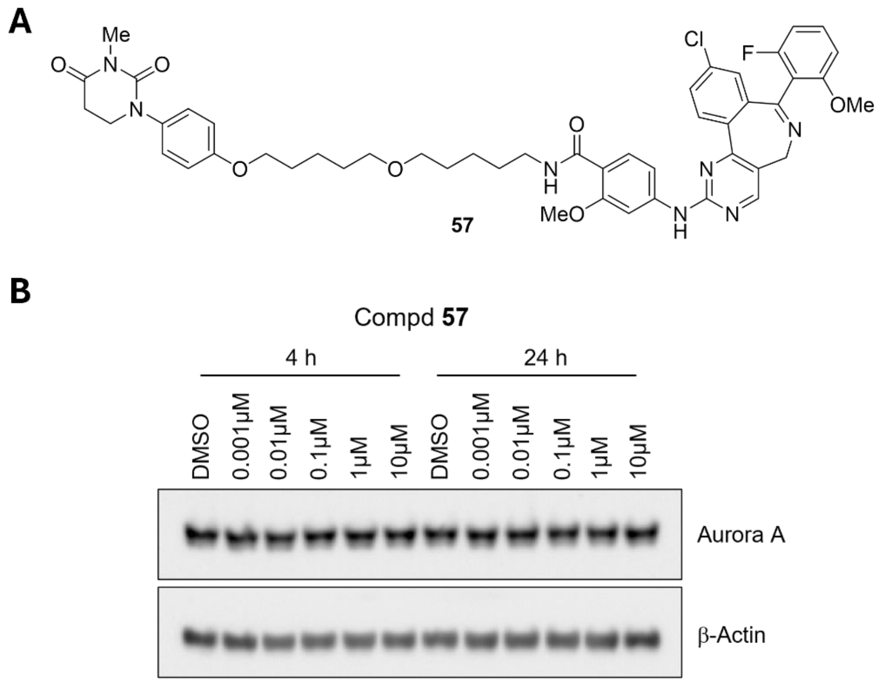

Figure S10. Effect of inactive control **57** on Aurora A protein levels in Kelly human paediatric neuroblastoma cells. (A) Chemical structure of **57**; (B) Kelly human neuroblastoma cells were treated with different concentrations of **57** for 4 and 24 h. Whole cell lysates were analysed by immunoblotting for Aurora A and β-Actin as the loading control (representative images of 2 independent experiments).

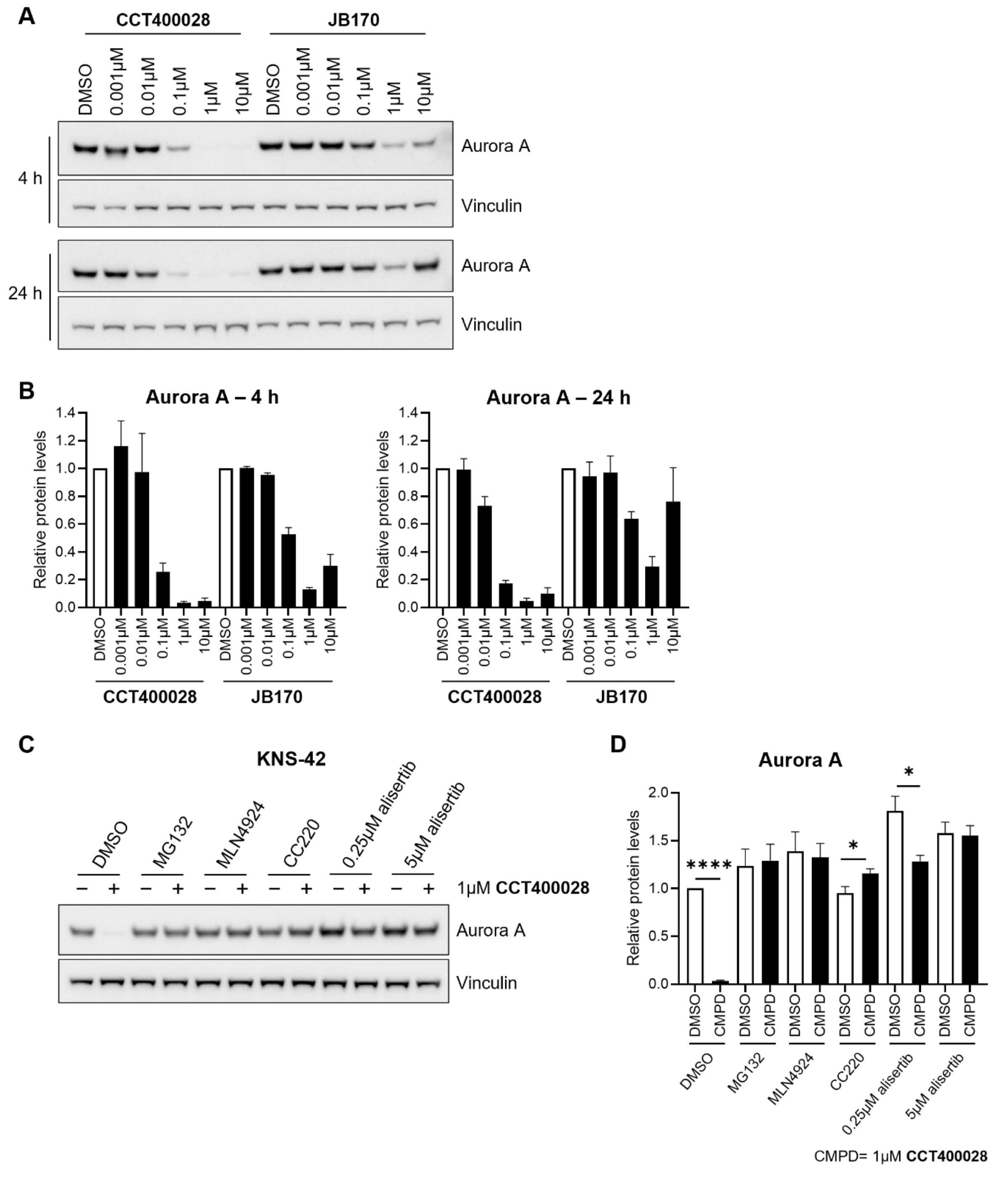

Figure S11. CCT400028 degrades Aurora-A in KNS-42 human paediatric glioma cells. (A) KNS-42 cells were treated with different concentrations of **CCT400028** and **JB170** for 4 and 24 h. Whole cell lysates were analysed by immunoblotting for Aurora A and vinculin (representative images of 3 independent experiments). (B) Quantification of (A) with n=3. (C) KNS-42 cells were pre-treated with **MG132** (10 µM), **MLN4924** (1 µM), **CC220** (10 µM) and 0.25 or 5 µM **Alisertib** for 2 h. This was followed by a combined treatment +/- 1 µM **CCT400028** for 4 h. Whole cell lysates were analysed by immunoblotting for Aurora A and Vinculin (representative images of 3 independent experiments). (D) quantification of (C) (n=3, $*p < 0.05$, $****p < 0.0001$, paired t-test).

Table S1. Summary statistics for Aurora A HiBiT data presented in **Table 1**, **3** and **5** (main text).

Cmpd	DC_{50} (nM)[a]	pDC_{50} ± SEM[b]	D_{max} ± SEM (%)[c]	n
**JB170**	39	7.4 ± 0.2	69 ± 2.3	5
**CCT400028**	29	7.5 ± 0.0	85 ± 0.4	3
**42**	6.9	8.1 ± 0.1	56 ± 2.0	6
**43**	17	7.8 ± 0.1	68 ± 2.3	4
**44**	37	7.5 ± 0.1	63 ± 1.4	6
**45**	52	7.3 ± 0.1	37 ± 2.7	3
**46**	6.0	8.2 ± 0.2	70 ± 4.7	3
**47**	18	7.8 ± 0.1	55 ± 1.5	4
**48**	48	7.3 ± 0.1	79 ± 1.2	11
**49**	48	7.3 ± 0.1	75 ± 0.4	3
**50**	8.0	8.1 ± 0.0	74 ± 0.7	3
**51**	6.5	8.2 ± 0.1	63 ± 2.4	3

[a]Data represent the geometric mean of at least three biological repeats. [b]$pDC_{50} = -\log DC_{50}$ (M). Data represent the mean ± SEM of at least three biological repeats. [c]Data represents the mean ± SEM of at least three biological repeats.

Table S2. Characterisation of selected PROTACs in the Aurora-A/CRBN TR-FRET assay.[a]

Cmpd	Amp. (% ctrl)	AUC (% ctrl)	EC_{max} (nM)	FWHM	TF_{50} (nM)	TI_{50} (nM)
**JB170**	100	100	25	1.9	2.7	230
**CCT400028**	77	95	620	2.4	40	9500
**44**	110	130	170	2.2	1.4	2000
**47**	170	200	70	2.2	6.0	840
**48**	140	170	590	2.4	39	8900
**51**	130	150	31	2.2	2.6	380

[a]TR-FRET assay data was collected in triplicate and analysed using Graphpad Prism (10.4.1; GAUSSIAN model). Parameters were determined according to Han.[1] AUC is area under the curve; FWHM is the full width at half-maximal points; EC_{max} is the maximally effective concentration; TF_{50} is the ternary complex–forming EC_{50}; TI_{50} is the ternary complex–inhibiting EC_{50}, where EC_{50} is defined as the half maximal effective concentration. % Amplitude (Amp.) and AUC were calculated with respect to values measured for JB170.

Table S3. Summary statistics for relative affinities of CRBN ligands measured in CRBN fluorescence polarisation (FP) assays using displacement of a fluorescently-labelled thalidomide-based probe.[3]

Cmpd	Structure	IC_{50} (nM)[a]	pIC_{50} ± SEM[b]	n
**52**	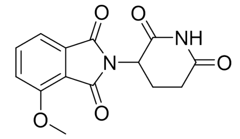	710	6.2 ± 0.09	3
**53**	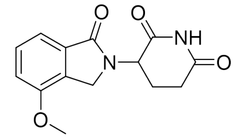	200	6.7 ± 0.14	3
**54**	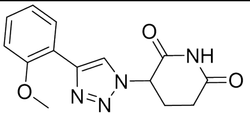	230	6.6 ± 0.15	3
**55**	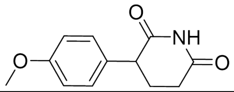	1100	6.0 ± 0.08	3
**56**	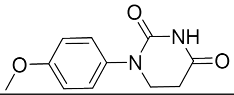	6300	5.2 ± 0.06	3

[a]Data represent the geometric mean of at least three biological repeats. [b]$pDC_{50} = -\log IC_{50}$ (M). Data represent the mean ± SEM of at least three biological repeats.

Table S4. Kinome profiling in Eurofins *scan*EDGE 97 Kinase Panel binding assay (Eurofins DiscoverX, LLC (San Diego)) for **CCT400028** and **50** at 1 μM in duplicate. Average remaining activity at % of control is presented for each kinase. Activities <35% of control are in bold italics.

	% control @ 1 μM	
**DiscoverX Gene Symbol**	**CCT400028**	**Compd 50**
ABL1(E255K)-phosphorylated	78	***21***
ABL1(T315I)-phosphorylated	96	37
ABL1-nonphosphorylated	46	***1.3***
ABL1-phosphorylated	69	***9.9***
ACVR1B	83	61
ADCK3	78	98
AKT1	79	88
AKT2	94	88
ALK	96	98
AURKA	***6.8***	***0.1***
AURKB	47	***7.9***
AXL	92	***10***
BMPR2	100	100
BRAF	100	100
BRAF(V600E)	100	100
BTK	100	79
CDK11	72	91
CDK2	100	100
CDK3	100	92
CDK7	100	100
CDK9	100	87
CHEK1	96	100
CSF1R	100	50
CSNK1D	100	93
CSNK1G2	99	99
DCAMKL1	98	100
DYRK1B	81	100
EGFR	95	51
EGFR(L858R)	94	45
EPHA2	81	64
ERBB2	82	91
ERBB4	86	72
ERK1	100	100

FAK	100	36
FGFR2	88	***34***
FGFR3	84	71
FLT3	76	66
GSK3B	78	93
IGF1R	100	85
IKK-alpha	100	100
IKK-beta	97	100
INSR	96	100
JAK2(JH1domain-catalytic)	100	100
JAK3(JH1domain-catalytic)	97	99
JNK1	93	93
JNK2	94	92
JNK3	100	89
KIT	100	91
KIT(D816V)	92	92
KIT(V559D, T670I)	100	98
LKB1	100	100
MAP3K4	94	100
MAPKAPK2	72	87
MARK3	79	100
MEK1	88	98
MEK2	99	100
MET	100	67
MKNK1	100	100
MKNK2	100	100
MLK1	100	100
p38-alpha	98	100
p38-beta	82	72
PAK1	87	98
PAK2	100	100
PAK4	87	100
PCTK1	100	100
PDGFRA	97	93
PDGFRB	84	45
PDPK1	80	88
PIK3C2B	100	100
PIK3CA	100	100
PIK3CG	99	97

PIM1	91	100
PIM2	90	100
PIM3	94	100
PKAC-alpha	93	60
PLK1	100	100
PLK3	94	100
PLK4	100	86
PRKCE	74	75
RAF1	69	74
RET	95	66
RIOK2	100	100
ROCK2	100	100
RSK2(Kin.Dom.1-N-terminal)	92	50
SNARK	100	64
SRC	74	***5.1***
SRPK3	93	100
TGFBR1	84	92
TIE2	72	***20***
TRKA	100	***24***
TSSK1B	100	93
TYK2(JH1domain-catalytic)	92	88
ULK2	100	100
VEGFR2	100	98
YANK3	97	61
ZAP70	100	100

Experimental Methods

Biological Methods

Recombinant protein production and purification

Human CRBN/DDB1 complex: The expression and purification of the His tagged WT CRBN/DDB1 complex was mentioned in our previously published work.[3]

Human NAvi-Aurora $A_{122-403}$: Residues E122 to S403 of Aurora A, together with an N-terminal His_{10}-HRV3C-Avitag, were cloned into a pCDFDUET-1 vector containing BirA. Expression was performed using BL21-AI cells grown in Terrific Broth. At an $OD_{600\ nm}$ > 1.0, 50 µM D-(+) biotin was added to the cultures and protein production was induced with 0.2 % L-Arabinose and 0.2 mM IPTG, followed by overnight incubation at 18 °C. The following day cultures were incubated at 37 °C for 1 hour to promote efficient biotinylation of the Avitag, then cells were harvested by centrifugation (6200 x g, 20 minutes, 4 °C). Cell pellets were re-suspended with a 5-fold volume of IMAC buffer A (50 mM HEPES pH 7.5, 500 mM NaCl, 5 mM $MgCl_2$, 0.5 mM TCEP, 10% Glycerol and 5 mM Imidazole) complemented with 1 mM $MgCl_2$, 1x cOmplete ULTRA protease inhibitors and 12.5 U/mL Benzonase. Cells were lysed by sonication followed by centrifugation (55,900 g, 45 minutes, 4 °C) and filtration through a 1.2 µM syringe filter. Clarified lysate was applied to a 5 mL HiTrap Talon crude column, washed with IMAC buffer A, and eluted with a gradient of IMAC Buffer B (IMAC Buffer A + 150 mM Imidazole) over 10 column volumes. Pooled fractions were then incubated with His-HRV-3C protease (~23 U/mg) overnight at 4 °C. After cleavage, the sample was concentrated and buffer exchanged into IMAC buffer A using a PD-10 desalting column. The sample was re-applied to a 5 mL HiTrap Talon crude column, the flow through fraction collected and loaded onto a HiLoad Superdex 200 16/600 pg column pre-equilibrated in 20 mM HEPES pH 7.0, 200 mM NaCl, 5 mM $MgCl_2$, 1 mM TCEP and 10 % Glycerol. Peak fractions were pooled, concentrated to 4.15 mg/mL flash frozen on dry ice and stored at −80 °C.

Aurora A HiBiT assay

Details of the assay were described in our previously published work.[4] In short, MV4–11 human pediatric leukemia cells stably expressing nHiBiT AURORA-A were seeded

in 96-well plates (20 000 cells/well) and treated with serial dilutions of AURORA-A PROTACs or DMSO for 6 h. The HiBiT assay (Promega) was using the Nano-Glo HiBiT Lytic Detection System. Luminescence was measured using the TECAN Spark Multiplate reader. DC_{50} values were calculated with the sigmoidal dose-response (four parameters) equation in GraphPad Prism. The hook region is defined as data points at concentrations above D_{max}, where Aurora A levels are $\geq D_{max} + 0.1D_{max}$.

Hydrolytic stability

MS grade solvents, formic acid, or alternative eluent modifiers were purchased from Fisher (Loughborough, UK) unless otherwise stated. PBS (Sigma 79382) was prepared using 1 tab per 200 mL of 18.2 MΩ-cm deionised water at pH 7.4.

10 µL of compound (10mM in DMSO) was diluted in PBS with 10% DMSO (100 mL) and vortexed thoroughly for 30 seconds. Samples were stored at 37 °C in a G1367B auto-sampler with G1330B thermostat module prior to injection. MS analysis was carried out at 0, 1, 3, 6, 24, 120 h time points. Nominal 3 µL injections (with needle wash) of samples were made onto a Kinetix C18 column (2.6 µM, 100A, 30 x 2.1 mm, 00A-4462-AN, Phenomenex, Torrence, USA). Sample was loaded onto the column using a G1312B SL binary pump dispensing a gradient from 95:5 to 5:95 water and methanol (both modified with 0.1% formic acid) at a flow rate of 0.5 mL/min. Chromatographic separation was carried out at 25 C using a 1200 Series HPLC (Agilent, Santa Clara, USA) over a 10 minute gradient elution. Between 2 and 10 minutes, the eluent flow was infused into a 6520 Series qToF mass spectrometer (G6520A) fitted with a dual ESI ionisation source (Agilent, Santa Clara, USA). LC eluent and nebulising gas was introduced into the grounded nebuliser with spray direction orthogonal to the capillary axis. The aerosol was dried by heated gas (12 L/min of nitrogen at 350 °C, 60 psig), producing ions by ESI. Ions entered the transfer capillary along which a potential difference of 4 kV was applied. The fragmentor voltage was set at 180 V and skimmer at 65 V. Signal was optimised by AutoTune.m. Profile mass spectrometry data was acquired in positive ionisation mode over a scan range of m/z 200–1200 (scan rate 1.0). Raw data was processed using Agilent MassHunter Qualitative Analysis B.07.00 sp2.

EIC (extracted ion chromatograms) for single or double charged ions for each compound and its sodium adduct, along with 1, 2, and 3 additions of water, were summed and integrated. Summed areas for the EICs of the compounds were taken as a percentage compared to the corresponding areas for the hydrolysed versions of each compound. Half-life values were calculated with the exponential one phase decay equation in GraphPad Prism 10.4.1.

Time-Resolved Fluorescence Resonance Energy Transfer (TR-FRET) assay

All TR-FRET assays were run in Black 384-well ProxiPlate Plus (Perkin-Elmer), in a buffer containing 20 mM HEPES pH 8.0, 150 mM NaCl, 0.5 mM TCEP, 0.05 % Tween 20 and 0.05 % BSA with a final assay volume of 10.3 µL. An Labcyte Echo 650 acoustic liquid dispenser was used to create final 16-point concentration ranges of compound from 97.1 µM to 0.006 nM, with a final DMSO concentration of 2.9 %. Acoustic dispensing was also used to add NAvi-AuroraA$_{122-403}$, anti-His-Tb and Streptavidin d2 to all wells to final concentrations of 5 nM, 0.5 nM and 1.25 nM, respectively. His tagged WT CRBN/DDB1 complex was added to the plate with a Thermo Scientific™ Multidrop™ Combi Reagent Dispenser to a final concentration of 5 nM. Plate was sealed, centrifuged at 200 x g for 1 minute and incubated at room temperature. The TR-FRET signal was measured on a PHERAstar FSX plate reader (BMG Labtech) after 4 hours of incubation at room temperature. Raw data from TR-FRET was plotted in Graphpad Prism 10.4.1 with GAUSSIAN fitting to extract amplitude, standard deviation and mean values and the fit quality was monitored via R^2. The parameters to evaluate the ternary complex system such as amplitude, full width half maximum (FWHM), area under the curve (AUC), EC_{max}, EC_{50} #1 (TF_{50}, ternary complex–forming EC_{50}) and EC_{50} #2 (TI_{50}, ternary complex–inhibitory EC50) were calculated and analysed based on a published work.[1]

TR-FRET Modelling and simulations

TR-FRET modelling to estimate cooperativity (α) and system calibration factor (β) was performed using the Extended LeastSumSquare method (extLSS) based Microsoft Excel program provided as a supporting document to work published by Han.[1] Prior to running the simulation, the equilibrium constant, K_{E1} was calculated using the

following formula, where K_{P1} was determined by Eurofins (Aurora A KdELECT) and EC_{max} calculated from GAUSSIAN curve fitting for the TR-FRET signals.

$$K_{E1} = ECmax^2/K_{P1}$$

The extLSS program was run according to published instructions with values provided for the raw TR-FRET signals over a 12-point concentration range (97 μM to 0.06 nM), the concentrations of Aurora A (5 nM) and CRBN (5 nM) used in TR-FRET experiment, and equilibrium constants K_{E1} and K_{P1}.

Next, simulated [PLE] values were obtained using the published simulator programmed in Microsoft Excel run according to the instructions provided.[1] The simulator was run using values for the concentrations of Aurora A (5 nM) and CRBN (5 nM) used in TR-FRET experiment, equilibrium constants K_{E1} and K_{P1}, the concentration range of compounds used for dose-response experiment (97 μM to 0.06 nM) and α values estimated using the extLSS program. The simulated [PLE] values for each compound were converted to ternary complex signal (TCS) by multiplying β according to the following published relationship: TCS = β x [PLE]. The TCS data was plotted in GraphPad Prism 10.4.1 with GAUSSIAN fitting against log of compound concentration (M).

CRBN FP assay

Details of the assay were described in our previously published work.[3] FP assays were run in Black 384-well ProxiPlate Plus (Perkin-Elmer, USA), in a buffer containing 20 mM HEPES pH 8.0, 150 mM NaCl, 0.5 mM TCEP and 0.05 % Tween 20, with a final assay volume of 10 μL. An Echo E5XX (Beckman Coulter, USA) acoustic liquid dispenser was used to create final concentration ranges of compound from 300 μM to 0.94 nM, with a final DMSO concentration of 3 %. WT full length CRBN/DDB1 complex was added with a Tempest liquid handler (Formulatrix, USA) to all wells to a final concentration of 80 nM, except for low controls where WT CRBN was substituted with mutant Y384A−W386 A CRBN (mutant unable to bind the probe). The Echo was also used to add 5 nM final concentration of Sulfo-Cy5 fluorescent thalidomide-based probe to each well. Plates were sealed, centrifuged at 200 g for 1 minute and stored

overnight at 4 °C. Fluorescence Polarization and total fluorescence were read using a PHERAstar FSX plate reader (BMG Labtech, Germany).

Kinase selectivity profiling

Kinome profiling was performed in Eurofins *scan*EDGE 97 Kinase Assay Panel (Eurofins DiscoverX, LLC (San Diego) https://www.eurofinsdiscoveryservices.com/) for **CCT400028** and **50** at 1 μM in duplicate. Average remaining activity as % of control is reported for each kinase, with lower numbers indicating stronger inhibition.

Kinase K_D determination

K_D values were determined for **CCT400028** and **50** against Aurora A, B and C in Eurofins KdELECT Binding LeadHunter Assay (Eurofins DiscoverX, LLC (San Diego) https://www.eurofinsdiscoveryservices.com/). Binding constants (K_ds) were calculated with a standard dose-response curve using the Hill equation: Response = Background + Signal - Background 1 + (Kd / Dose). The Hill Slope was set to -1. Curves were fitted using a non-linear least square fit with the Levenberg-Marquardt algorithm.

AlamarBlue cell viability assay

Details of the assay were described in our previously published work.[4] In short, human paediatric leukaemia MV4-11 cells were seeded in 96 well plates (9000 cells/well) and treated with serial dilutions of compounds for 72 h. The alamarBlue was performed by adding alamarBlue™ HS Cell Viability Reagent (Thermo Fisher Scientific). After an incubation time of 90 min, fluorescence was measured on a Spark Multimode Microplate Reader (Promega) using an excitation wavelength of 560 nm and an emission wavelength of 590 nm. GI_{50} values were calculated with the sigmoidal dose-response (four parameters) equation in GraphPad Prism.

KNS-42 and Kelly cell culture and treatments

KNS-42 human paediatric glioma cells were obtained from the JCRB Cell Bank (Japanese Collection of Research Bioresources Cell Bank). Kelly human paediatric neuroblastoma cells were obtained from the DSMZ (German Collection of Microorganisms and Cell Cultures). Cell lines were grown in their recommended

culture medium, supplemented with 10% FBS, at 37 °C in 5% CO_2. In-house authentication of cell lines by SNP profiling was carried out and cells were regularly screened for Mycoplasma. The following commercially available compounds were used at various concentrations as indicated: **MG132** (Sigma-Aldrich), **MLN4924** (Cayman Chemical), **CC220** (Selleck Chemicals), **alisertib** (**MLN8237**; Selleck Chemicals). For single compound treatments, cells were seeded, the next day they were treated with compounds at various concentrations and harvested after 4 h and 24 h. For rescue experiments, cells were pre-treated for 2 h, followed by a combined treatment for 4 h.

Immunoblotting

Proteins were extracted from cells using 2x LDS sample buffer (Invitrogen). Equal amounts of protein were separated by SDS-PAGE using 4-12% Bis-Tris NuPAGE gels (Invitrogen), transferred to nitrocellulose membrane (Amersham), and detected with antibodies against Aurora A (CST4718, Cell Signaling Technology), β-Actin (A1978, clone AC-15, Sigma-Aldrich), Vinculin (V9264, Sigma-Aldrich), GSPT1 (HPA052488, Sigma-Aldrich) and CDK9 (CST2316, Cell Signaling Technology). Secondary HRP-conjugated antibodies Swine Anti-Rabbit (P0217, Dako) and Rabbit Anti-Mouse (P0260, Dako) were used. Protein bands were visualised using Pierce ECL Western Blotting Substrate (Thermo Scientific) and ChemiDoc Imaging System (Bio-Rad). Protein bands were quantified using the Image Lab Software (Bio-Rad). Values were normalised to their respective loading control. If not otherwise indicated, results are presented as mean values ± standard error of the mean (SEM) of at least three independent experiments. Statistical analyses were performed in Prism (GraphPad software) using Student`s t-test. P-values <0.05 were considered statistically significant.

Proteomics profiling

Kelly human paediatric neuroblastoma cells were plated on 10 cm dishes at $6x10^6$ cells total. The next day cells were treated with 1 µM of compounds. After 4 h cells were washed three times with cold PBS and cell pellets were collected and stored at -80 °C until further analysis. The samples of three biological replicates were prepared and analysed simultaneously by 12-plex TMT-Mass spectrometry.

Cell pellets were lysed in a buffer containing 1% sodium deoxycholate (SDC), 100 mM triethylammonium bicarbonate (TEAB), 10% isopropanol, and 50 mM NaCl, freshly supplemented with 5 mM TCEP (Thermo, Bond-breaker), 10 mM iodoacetamide (IAA), universal nuclease 1:2000 vol/vol (Pierce, #88700) and Halt protease and phosphatase inhibitor cocktail (Thermo, #78442, 100X) with 5 min of bath sonication. Protein concentration was measured with the Quick Start Bradford protein assay (Bio-Rad). Aliquots of 30 μg of total protein were digested overnight with trypsin (Pierce, 1:20) at room temperature. Peptides were labelled with the TMTpro reagents (Thermo) by adding 5 μL of the reagent (25 μg/μL) into 12.5 μL of sample volume. The TMTpro mixture was acidified with formic acid at 2% and the precipitated SDC was removed by centrifugation. The peptide pool was fractionated with high pH Reversed-Phase chromatography using the XBridge C18 column (2.1 × 150 mm, 3.5 μm, Waters) on an UltiMate 3000 HPLC system over a 1% gradient in 35 min. Mobile phase A was 0.1% (v/v) ammonium hydroxide and mobile phase B was 0.1% ammonium hydroxide (v/v) in acetonitrile.

LC-MS analysis was performed on an UltiMate 3000 system coupled with the Orbitrap Ascend mass spectrometer (Thermo) using a 25 cm capillary column (Waters, nanoE MZ PST BEH130 C18, 1.7 μm, 75 μm × 250 mm) over a 100 min gradient 5%-27% of mobile phase B composed of 80% acetonitrile, 0.1% formic acid. Peptides were preconcentrated onto an Acclaim PepMap 100, 100 μm × 2 cm C18, 5 μm trapping column at 10 μL/min of 0.1% TFA and the analytical column was connected to an EASY-Spray emitter (Thermo ES991). MS spectra were collected at Orbitrap mass resolution of 120k and precursors were targeted for HCD fragmentation in the top speed mode (3 sec) with collision energy 32% and iontrap detection in turbo scan rate. MS3 scans were triggered by Real Time Search (RTS) against a fasta file containing UniProt Homo sapiens reviewed canonical and isoform sequences with multi-notch isolation (10 notches) and HCD fragmentation with collision energy 55% at 45k Orbitrap resolution. Targeted precursors were dynamically excluded from further activation for 45 seconds with 10 ppm mass tolerance and RTS close-out was enabled with max 4 peptides per protein. Static modifications for RTS were TMTpro16plex at K/n-term (+304.2071), Carbamidomethyl at C (+57.0215) and variable modifications were Deamidated NQ (+0.984) and Oxidation of M (+15.9949) with maximum 1 missed-cleavage and 2 variable modifications per peptide.

The Sequest HT and Comet nodes in Proteome Discoverer 3.0 (Thermo) were used to search the raw mass spectra against a fasta file containing reviewed UniProt Homo sapiens entries. The precursor mass tolerance was set at 20 ppm and the fragment ion mass tolerance at 0.5 Da (or 1 Da for Comet) with up to 2 trypsin missed-cleavages allowed. TMTpro at N-terminus/K and Carbamidomethyl at C were defined as static modifications. Dynamic modifications were oxidation of M and deamidation of N/Q. Peptide confidence was estimated with the Percolator node and peptide FDR was set at 0.01 based on target-decoy search. Only unique peptides were used for quantification, considering protein groups for peptide uniqueness. Peptides with average reporter signal-to-noise greater than 3 were used for protein quantification. Data normalization by sample median, log2 scaling per experiment and differential analysis were performed with an in-house pipeline in RStudio using the HybridMTest package from Bioconductor; volcano plots were made using the VolcaNoseR web app.[5]

The mass spectrometry proteomics data have been deposited to the ProteomeXchange Consortium via the PRIDE partner repository with the dataset identifier PXD070555.[6]

Notes

This research did not involve human or animal participants

Synthetic Methods

General details

Reactions were carried out under N_2 at room temperature unless otherwise stated. All anhydrous solvents and reagents were obtained from commercial suppliers and used without further purification. Where available, compounds were purchased or made according to literature procedure as cited. Compounds **52**, **53** and **54** were sourced from commercial suppliers. The synthesis and characterization of **JB170** was described in our previously published work.[4] 3-Aminoglutarimide hydrochloride, 3-bromoglutarimide, and 4-hydroxythalidomide were supplied as racemic mixtures. Evaporation of solvents was carried out using a rotary evaporator under reduced pressure at a bath temperature of up to 50 °C, or a Biotage® V-10 Touch Evaporation System using pre-set methods. Flash column chromatography was carried out using a Biotage purification system using prepacked SNAP KP-Sil or Sfär Silica HC D cartridges or on an Isco purification system using prepacked RediSep Rf cartridges or on the reverse-phase mode using SNAP Ultra C18 or Sfär C18D cartridges. Microwave-assisted reactions were carried out using a Biotage initiator microwave system. All final compounds are >95% pure by HPLC. NMR data were collected on a Bruker AVANCE 500 spectrometer equipped with a 5 mm BBO/QNP probe or on a Bruker AVANCE Neo 600 spectrometer equipped with a 5 mm TCI Cryo-Probe. NMR data are presented in the form of chemical shift δ (multiplicity, coupling constants, integration) for major diagnostic protons, given in parts per million relative to tetramethylsilane, referenced to the internal deuterated solvent. High-resolution mass spectrometry (HRMS) was assessed using an Agilent 1200 series HPLC and a diode array detector coupled to a 6120 time of a flight mass spectrometer with a dual multimode APCI/ESI source or on a Waters Acquity UHPLC and a diode array detector coupled to a Waters G2 QToF mass spectrometer fitted with a multimode ESI/APCI source.

Abbreviations used: aq for aqueous, 9-BBN for 9-borabicyclo[3.3.1]nonane, Bn for benzyl, Boc for *tert*-butyloxy carbonyl, Bu for butyl, DIPEA for *N*,*N*-diisopropylethylamine, DMAP for 4-dimethylaminopyridine, DMEDA for *N*,*N*'-dimethylethylenediamine, DMF for dimethylformamide, DMSO for dimethylsulfoxide,

dppf for 1,1-bis(diphenylphosphino)ferrocene, DTBAD for di-*tert*-butyl azodicarboxylate, HBTU for hexafluorophosphate benzotriazole tetramethyl uronium, HRMS for high-resolution mass spectrometry, HSQC for heteronuclear single quantum coherence, NBS for *N*-bromo succinimide, NMM for *N*-methyl morpholine, NMR for nuclear magnetic resonance, pin for pinacol, SEM for 2-(trimethylsilyl)ethoxymethyl, TFA for trifluoroacetic acid, THF for tetrahydrofuran, TMS for trimethylsilyl.

Experimental procedures

Synthesis of Common Intermediate A - *tert*-butyl *N*-[5-(5-bromopentoxy)pentyl] carbamate (4)

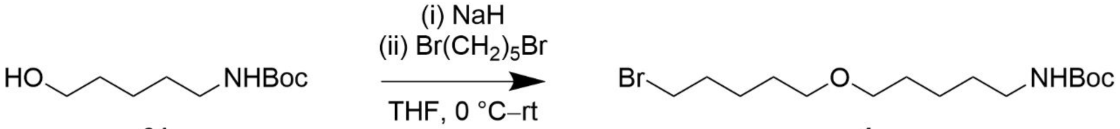

tert-Butyl *N*-[5-(5-bromopentoxy)pentyl] carbamate (4)

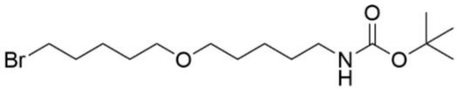

NaH (60% w/w in mineral oil, 91 mg, 2.3 mmol, 1.0 eq) was added to a solution of *tert*-butyl *N*-(5-hydroxypentyl)carbamate (**61**) (460 mg, 2.3 mmol, 1.0 eq) in THF (0.92 mL) under an atmosphere of N_2 at 0 °C. The solution was stirred at 0 °C for 30 min and a solution of 1,5-dibromopentane (0.46 mL, 3.4 mmol, 1.5 eq) in THF (4.0 mL) was added. The solution was stirred at room temperature for 23 h and poured into ice-water (25 mL). The solution was extracted with EtOAc (3 × 30 mL). The combined organic layers were washed with H_2O (10 mL) and brine (10 mL), dried over Na_2SO_4, filtered, adsorbed onto silica, and purified by normal-phase flash chromatography (0–25% EtOAc/cyclohexane) to afford the title compound (226 mg, 28%) as a colourless oil. ^{1}H NMR (500 MHz, chloroform-*d*) δ 4.54 (s, 1H), 3.43 – 3.36 (m, 6H), 3.11 (t, *J* = 8.2 Hz, 2H), 1.88 (dt, *J* = 14.8, 6.9 Hz, 2H), 1.62 – 1.53 (m, 4H), 1.53 – 1.45 (m, 4H), 1.43 (s, 9H), 1.41 – 1.32 (m, 2H); ^{13}C NMR (126 MHz, chloroform-*d*) δ 156.1, 79.2,

70.9, 70.7, 40.7, 33.9, 32.8, 30.0, 29.5, 29.0, 28.6, 25.1, 23.7; HRMS calc. for $C_{15}H_{31}{}^{79}BrNO_3$ [M+H]+ 352.1482, found 352.1477.

Synthesis of 4-[[9-chloro-7-(2-fluoro-6-methoxy-phenyl)-5*H*-pyrimido[5,4-*d*][2]benzazepin-2-yl]amino]-*N*-[5-[5-[4-(2,4-dioxohexahydropyrimidin-1-yl)phenoxy]pentoxy] pentyl]-2-methoxy-benzamide (CCT400028)

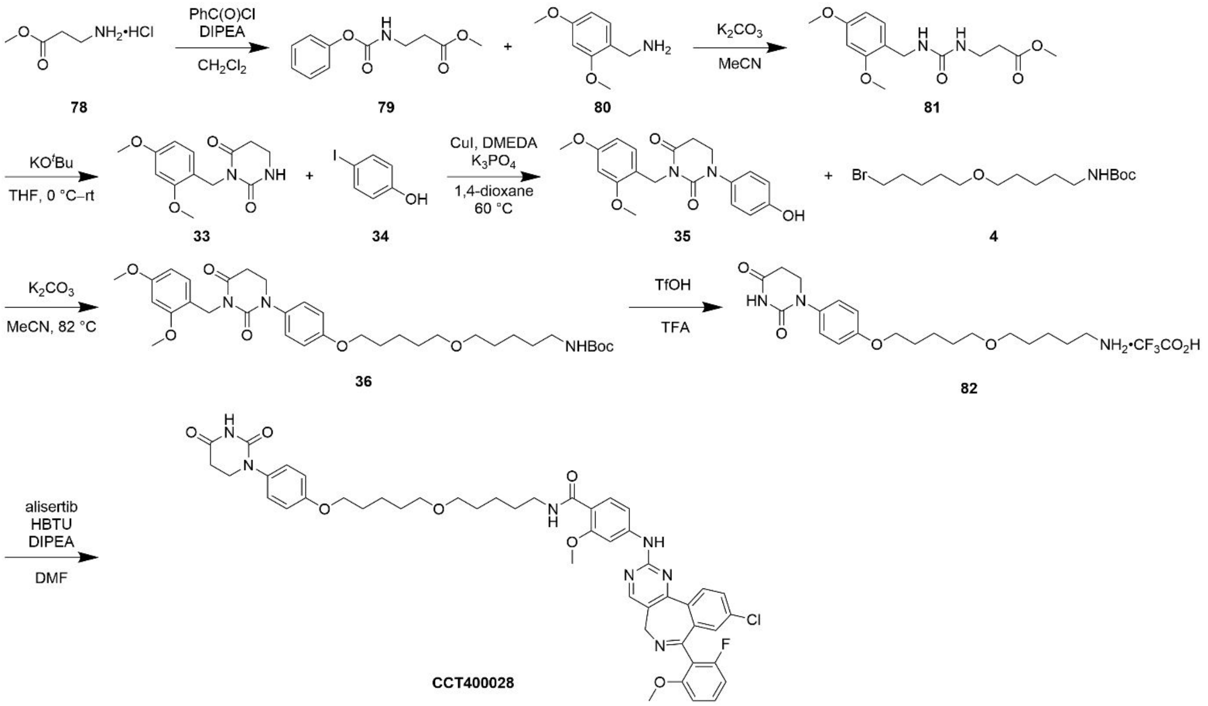

Methyl 3-(phenoxycarbonylamino)propanoate (79)

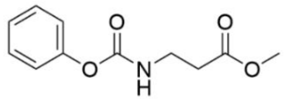

Phenyl chloroformate (1.8 mL, 14.3 mmol, 1.0 eq) was added to a solution of methyl 3-aminopropanoate hydrochloride (**78**) (2.0 g, 14.3 mmol, 1.0 eq) and *N*,*N*-diisopropylethylamine (5.5 mL, 31.5 mmol, 2.2 eq) in CH_2Cl_2 (36 mL) under an atmosphere of N_2. The solution was stirred at room temperature for 19 h then washed with saturated aqueous NH_4Cl (30 mL), saturated aqueous $NaHCO_3$ (30 mL), and brine (20 mL). The organic layer was passed through a phase separator and concentrated to afford the title compound (3.0 g, 94%) as an off-white amorphous solid. ^1H NMR (600 MHz, chloroform-*d*) δ 7.35 (t, *J* = 7.8 Hz, 2H), 7.19 (t, *J* = 7.4 Hz,

1H), 7.12 (d, *J* = 8.0 Hz, 2H), 5.61 (s, 1H), 3.73 (s, 3H), 3.54 (q, *J* = 6.1, 5.6 Hz, 2H), 2.62 (t, *J* = 6.0 Hz, 2H); ^{13}C NMR (151 MHz, chloroform-*d*) δ 172.9, 154.7, 151.1, 129.4, 125.4, 121.7, 52.0, 36.80, 34.2; HRMS calc. for $C_{11}H_{13}NaNO_4$ $[M+Na]^+$ 246.0742, found 246.0743.

Methyl 3-[(2,4-dimethoxyphenyl)methylcarbamoylamino]propanoate (81)

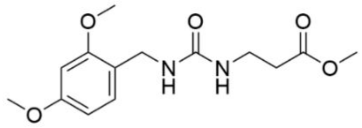

2,4-Dimethoxybenzylamine (**80**) (3.0 mL, 19.8 mmol, 1.5 eq) was added to a mixture of methyl 3-(phenoxycarbonylamino)propanoate (**79**) (2.9 g, 13.2 mmol, 1.0 eq) and K_2CO_3 (4.6 g, 32.9 mmol, 2.5 eq) in MeCN (26 mL) under an atmosphere of N_2. The reaction was stirred at room temperature for 20 h then concentrated. The residue was dissolved in EtOAc (200 mL) and the organic layer was washed with H_2O (2 × 50 mL), saturated aqueous K_2CO_3 (30 mL), saturated aqueous NH_4Cl (50 mL), and brine (50 mL). The organic layer was dried over Na_2SO_4, filtered, and concentrated to afford the title compound (5.4 g, 99%). The product was isolated with phenol (73% purity). ^{1}H NMR (600 MHz, DMSO-*d*$_6$) δ 7.04 (d, *J* = 8.3 Hz, 1H), 6.52 (d, *J* = 2.4 Hz, 1H), 6.45 (dd, *J* = 8.3, 2.4 Hz, 1H), 6.11 (t, *J* = 5.9 Hz, 1H), 6.02 (t, *J* = 5.9 Hz, 1H), 4.05 (d, *J* = 6.0 Hz, 2H), 3.77 (s, 3H), 3.73 (s, 3H), 3.59 (s, 3H), 3.22 (q, *J* = 6.4 Hz, 2H), 2.42 (t, *J* = 6.5 Hz, 2H); ^{13}C NMR (151 MHz, DMSO-*d*$_6$) δ 172.2, 159.6, 157.8, 157.7, 128.7, 120.3, 104.2, 98.2, 55.3, 55.2, 51.3, 37.8, 35.3, 34.8; HRMS calc. for $C_{14}H_{21}N_2O_5$ [M+H]+ 297.1450, found 297.1448.

3-[(2,4-Dimethoxyphenyl)methyl]hexahydropyrimidine-2,4-dione (33)

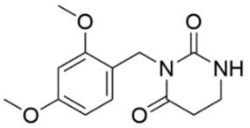

Potassium *tert*-butoxide (1.90 g, 16.9 mmol, 1.3 eq) was added slowly to a solution of methyl 3-[(2,4-dimethoxyphenyl)methylcarbamoylamino]propanoate (**81**) (73% purity, 5.35 g, 13.0 mmol, 1.0 eq) in THF (43 mL) under an atmosphere of N_2. The solution

was stirred at room temperature for 60 minutes then quenched with solid NH_4Cl (1.0 g). The solution was adsorbed onto silica and purified by normal-phase flash chromatography (0–8% $MeOH/CH_2Cl_2$) to afford the title compound (1.71 g, 50%) as an off-white amorphous solid. ^1H NMR (600 MHz, DMSO-d_6) δ 7.84 (t, J = 2.8 Hz, 1H), 6.74 (d, J = 8.4 Hz, 1H), 6.53 (d, J = 2.4 Hz, 1H), 6.42 (dd, J = 8.4, 2.4 Hz, 1H), 4.67 (d, J = 1.0 Hz, 2H), 3.78 (s, 3H), 3.72 (s, 3H), 3.28 (td, J = 6.8, 2.8 Hz, 2H), 2.67 (t, J = 6.8 Hz, 2H); ^{13}C NMR (151 MHz, DMSO-d_6) δ 169.9, 159.2, 157.1, 153.8, 126.1, 117.7, 104.3, 98.1, 55.4, 55.2, 37.3, 34.3, 31.4; HRMS calc. for $C_{13}H_{16}NaN_2O_4$ [M+H]$^+$ 287.1008, found 287.1013.

3-[(2,4-Dimethoxyphenyl)methyl]-1-(4-hydroxyphenyl)hexahydropyrimidine-2,4-dione (35)

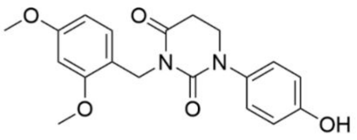

CuI (72.0 mg, 0.38 mmol, 0.4 eq), *N*,*N'*-dimethylethylenediamine (41.0 µL, 0.38 mmol, 0.4 eq) and K_3PO_4 (916 mg, 4.3 mmol, 4.5 eq) were added to a solution of 3-[(2,4-dimethoxyphenyl)methyl]hexahydropyrimidine-2,4-dione (**33**) (250 mg, 0.95 mmol, 1.0 eq) and 4-iodophenol (**34**) (208 mg, 0.95 mmol, 1.0 eq) in 1,4-dioxane (2.7 mL) under an atmosphere of N_2. The solution was stirred at 60 °C for 22 h then cooled to room temperature and diluted with EtOAc (40 mL). The solution was washed with saturated aqueous NH_4Cl (20 mL), water (20 mL), and brine (15 mL), adsorbed onto silica, and purified by normal-phase flash chromatography (0–35% $EtOAc/CH_2Cl_2$) to afford the title compound (186 mg, 55%) as a white amorphous solid. ^1H NMR (600 MHz, DMSO-d_6) δ 9.48 (s, 1H), 7.14 – 7.09 (m, 2H), 6.85 (d, J = 8.5 Hz, 1H), 6.79 – 6.73 (m, 2H), 6.54 (d, J = 2.4 Hz, 1H), 6.44 (dd, J = 8.4, 2.4 Hz, 1H), 4.74 (s, 2H), 3.79 (s, 3H), 3.75 (t, J = 6.7 Hz, 2H), 3.73 (s, 3H), 2.89 (t, J = 6.7 Hz, 2H); ^{13}C NMR (151 MHz, DMSO-d_6) δ 169.5, 159.3, 157.2, 155.7, 152.5, 134.0, 127.0, 126.5, 117.6, 115.2, 104.4, 98.1, 55.4, 55.2, 44.1, 38.2, 31.6; HRMS calc. for $C_{19}H_{21}N_2O_5$ [M+H]$^+$ 357.1450, found 357.1449.

tert-Butyl _N_-[5-[5-[4-[3-[(2,4-dimethoxyphenyl)methyl]-2,4-dioxo-hexahydro pyrimidin-1-yl]phenoxy]pentoxy]pentyl]carbamate (36)

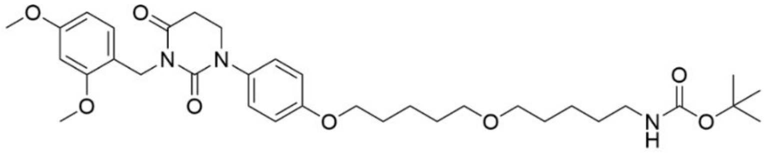

A solution of 3-[(2,4-dimethoxyphenyl)methyl]-1-(4-hydroxyphenyl)hexahydro pyrimidine-2,4-dione (**35**) (147 mg, 0.38 mmol, 1.0 eq), Common Intermediate A (**4**) (135 mg, 0.38 mmol, 1.0 eq), and K_2CO_3 (159 mg, 1.2 mmol, 3.0 eq) in MeCN (1.9 mL) was stirred at 82 °C for 20 h then cooled to room temperature and diluted with EtOAc (30 mL). The solution was washed with H_2O (10 mL) and brine (10 mL), adsorbed onto silica, and purified by normal-phase flash chromatography (0–60% EtOAc/CH_2Cl_2) to afford the title compound (167 mg, 69%) as a pale orange oil. ^{1}H NMR (600 MHz, chloroform-*d*) δ 7.19 – 7.14 (m, 2H), 7.08 (d, *J* = 8.1 Hz, 1H), 6.90 – 6.85 (m, 2H), 6.44 – 6.39 (m, 2H), 4.99 (s, 2H), 4.54 (s, 1H), 3.94 (t, *J* = 6.5 Hz, 2H), 3.81 (s, 3H), 3.77 (s, 3H), 3.75 (t, *J* = 6.6 Hz, 2H), 3.45 – 3.37 (m, 4H), 3.11 (q, *J* = 6.7 Hz, 2H), 2.88 (t, *J* = 6.6 Hz, 2H), 1.79 (p, *J* = 6.7 Hz, 2H), 1.67 – 1.54 (m, 4H), 1.55 – 1.46 (m, 4H), 1.43 (s, 9H), 1.41 – 1.33 (m, 2H);[*] ^{13}C NMR (151 MHz, chloroform-*d*) δ 169.2, 160.1, 158.4, 157.7, 156.1, 153.3, 135.1, 129.2, 126.7, 118.3, 115.1, 104.1, 98.7, 79.1, 70.87, 70.85, 68.3, 55.6, 55.5, 44.6, 40.7, 39.4, 32.6, 30.0, 29.6, 29.5, 29.2, 28.6, 23.7, 22.9; HRMS calc. for $C_{34}H_{50}N_3O_8$ $[M+H]^+$ 628.3598, found 628.3599.

1-[4-[5-(5-Aminopentoxy)pentoxy]phenyl]hexahydropyrimidine-2,4-dione; trifluoroacetic acid salt (37)

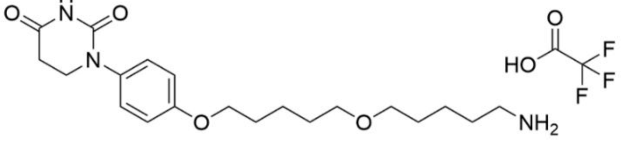

Trifluoroacetic acid (0.190 mL, 2.50 mmol, 10 eq) was added to a solution of *tert*-butyl *N*-[5-[5-[4-[3-[(2,4-dimethoxyphenyl)methyl]-2,4-dioxo-hexahydropyrimidin-1-

[*]CH obscured by chloroform-*d* residual water peak

yl]phenoxy]pentoxy]pentyl]carbamate (**36**) (157 mg, 0.250 mmol, 1.0 eq) in DCM (2.5 mL). The solution was stirred at room temperature for 4.5 hours then concentrated. The residue was dissolved in trifluoroacetic acid (1.3 mL) and trifluoromethanesulfonic acid (0.089 mL, 1.00 mmol, 4.0 eq) was added dropwise. The solution was stirred at room temperature for 19 h then concentrated. The residue was dissolved in EtOAc (20 mL), and the solution was washed with saturated aqueous $NaHCO_3$ (2 × 10 mL), H_2O (10 mL), and brine (10 mL). The product was present in the organic and aqueous layer so both layers were concentrated and combined. The mixture was purified by normal-phase flash chromatography (0–30% MeOH/CH_2Cl_2) then concentrated. The residue was dissolved in MeOH (2.0 mL) and trifluoroacetic acid (0.04 mL) was added. The solution was concentrated to afford the title compound (193 mg, 99%) as a purple solid. The product was isolated as a mixture with *N*-(5-((5-(4-(2,4-dioxotetrahydropyrimidin-1(2H)-yl)phenoxy)pentyl)oxy)pentyl)-2,2,2-trifluoroacetamide (63% purity).

4-[[9-Chloro-7-(2-fluoro-6-methoxy-phenyl)-5*H*-pyrimido[5,4-*d*][2]benzazepin-2-yl]amino]-*N*-[5-[5-[4-(2,4-dioxohexahydropyrimidin-1-yl)phenoxy]pentoxy] pentyl]-2-methoxy-benzamide (CCT400028)

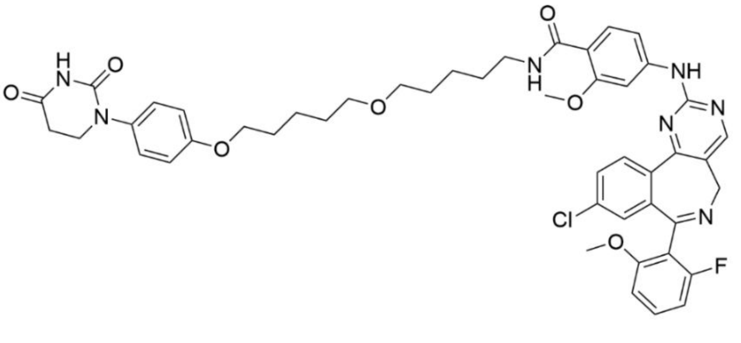

O-(Benzotriazol-1-yl)-*N*,*N*,*N'*,*N'*-tetramethyluronium hexafluorophosphate (91.0 mg, 0.24 mmol, 1.0 eq) was added to a solution of 1-[4-[5-(5-aminopentoxy)pentoxy]phenyl]hexahydropyrimidine-2,4-dione; trifluoroacetic acid salt (**37**) (188 mg, 0.24 mmol, 1.0 eq) in DMF (6.0 mL) and the solution was stirred until everything dissolved. Alisertib (113 mg, 0.22 mmol, 0.90 eq) and *N*,*N*-diisopropylethylamine (0.17 mL, 0.96 mmol, 4.0 eq) were added and the solution was stirred at room temperature for 22 h. The solution was diluted with EtOAc (60 mL) then washed with saturated aqueous NH_4Cl (15 mL), saturated aqueous $NaHCO_3$ (15 mL), H_2O (2 × 30 mL), and brine (10 mL). The solution was adsorbed onto silica and purified

by normal-phase flash chromatography (0–6% MeOH/CH_2Cl_2) then reversed-phase flash chromatography (50–100% MeOH containing 0.1% HCO_2H/H_2O containing 0.1% HCO_2H) to afford the title compound (40 mg, 19%) as a yellow amorphous solid. ^1H NMR (600 MHz, DMSO-d_6) δ 10.29 (s, 1H), 10.17 (s, 1H), 8.72 (s, 1H), 8.30 (d, *J* = 8.5 Hz, 1H), 8.03 (t, *J* = 5.7 Hz, 1H), 7.96 (br s, 1H), 7.85 – 7.78 (m, 2H), 7.46 – 7.39 (m, 2H), 7.26 – 7.17 (m, 3H), 6.92 – 6.89 (m, 2H), 3.96 – 3.91 (m, 5H), 3.69 (t, *J* = 6.7 Hz, 2H), 3.37 (td, *J* = 6.5, 2.4 Hz, 4H), 3.30 – 3.26 (m, 2H), 2.68 (t, *J* = 6.7 Hz, 2H), 1.71 (p, *J* = 6.7 Hz, 2H), 1.59 – 1.49 (m, 6H), 1.48 – 1.40 (m, 2H), 1.39 – 1.31 (m, 2H);[†] ^{13}C NMR (151 MHz, DMSO-d_6) δ 170.6, 164.3, 160.8, 159.6 (d, *J* = 244.4 Hz), 159.4, 157.7, 157.2, 152.3, 144.4, 137.5, 135.2, 134.8, 134.6, 131.4, 131.2 (d, *J* = 8.3 Hz), 130.9, 130.2, 127.5, 126.8, 123.4, 115.1, 114.4, 110.4, 108.0, 101.5, 69.89, 69.85, 67.6, 56.3, 55.7, 49.4, 44.9, 38.9, 31.1, 29.1, 29.0, 28.5, 23.3, 22.4; HRMS calc. for $C_{47}H_{50}{}^{35}ClFN_7O_7$ $[M+H]^+$ 878.3444, found 878.3433.

[†]CH_2, OCH_3 and 2 x CH not observed due to peak broadening

Synthesis of 4-[[9-chloro-7-(2-fluoro-6-methoxy-phenyl)-5H-pyrimido[5,4-*d*][2]benzazepin-2-μyl]amino]-*N*-[2-[2-[2-[2-[2-(2,6-dioxo-3-piperidyl)-1,3-dioxo-isoindolin-4-yl]oxyethoxy]ethoxy]ethoxy]ethyl]-2-methoxy-benzamide (42)

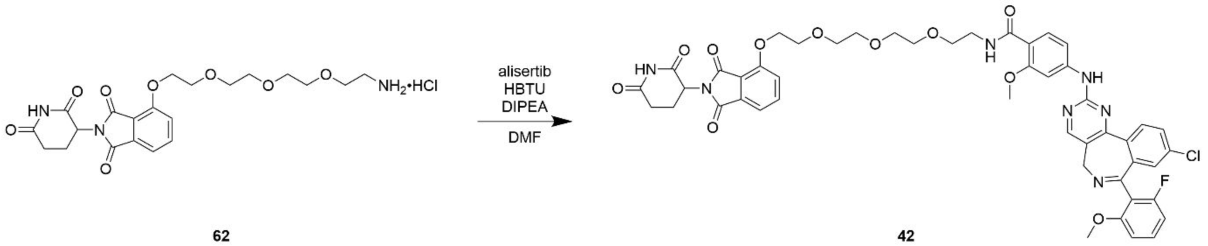

62 42

4-[[9-Chloro-7-(2-fluoro-6-methoxy-phenyl)-5H-pyrimido[5,4-*d*][2]benzazepin-2-μyl]amino]-*N*-[2-[2-[2-[2-[2-(2,6-dioxo-3-piperidyl)-1,3-dioxo-isoindolin-4-yl]oxyethoxy]ethoxy]ethoxy]ethyl]-2-methoxy-benzamide (42)

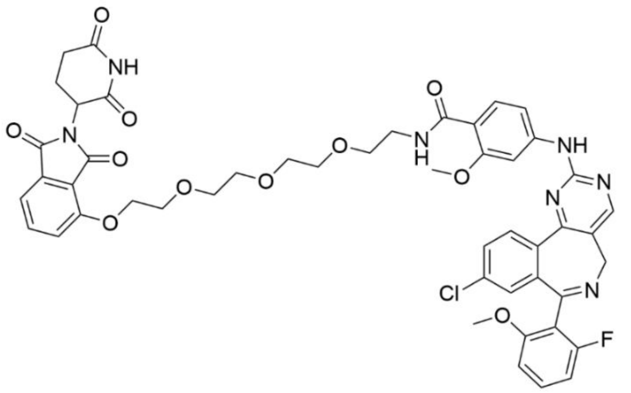

O-(*B*enzotriazol-1-yl)-*N*,*N*,*N*',*N*'-tetramethyluronium hexafluorophosphate (9.4 mg, 0.025 mmol, 1.0 eq) was added to a solution of 4-[2-[2-[2-(2-aminoethoxy) ethoxy]ethoxy]ethoxy]-2-(2,6-dioxo-3-piperidinyl)-1*H*-isoindole-1,3(2*H*)-dione hydrochloride (**62**) (12 mg, 0.025 mmol, 1.0 eq) in DMF (1.2 mL) and the solution was stirred until everything was dissolved. Alisertib (12.8 mg, 0.025 mmol, 1.0 eq) and *N*,*N*-diisopropylethylamine (17 μL, 0.099 mmol, 4.0 eq) were added and the solution stirred at room temperature for 19 h. Solvent was removed on a Biotage V10, the residue was dissolved in MeOH/H_2O/DMSO and purified by reversed-phase flash chromatography (30–100% MeOH containing 0.1% HCO_2H/H_2O containing 0.1% HCO_2H) to afford the title compound (8.6 mg, 36%) as a white amorphous solid. ^{1}H NMR (500 MHz, DMSO-d_6) δ 11.09 (s, 1H), 10.18 (s, 1H), 8.71 (s, 1H), 8.30 (d, *J* = 8.5 Hz, 1H), 8.16 (t, *J* = 5.4 Hz, 1H), 7.97 (s, 1H), 7.85 (d, *J* = 8.6 Hz, 1H), 7.81 (dd, *J*

= 8.5, 2.2 Hz, 1H), 7.76 (dd, *J* = 8.5, 7.3 Hz, 1H), 7.48 (d, *J* = 8.4 Hz, 1H), 7.45 – 7.37 (m, 3H), 7.22 (br s, 1H), 6.99 – 6.73 (br m, 2H), 5.07 (dd, *J* = 12.8, 5.4 Hz, 1H), 4.33 – 4.28 (m, 2H), 3.93 (s, 3H), 3.83 (d, *J* = 12.6 Hz, 1H), 3.80 – 3.76 (m, 2H), 3.64 (dd, *J* = 5.9, 3.6 Hz, 2H), 3.58 – 3.50 (m, 8H), 3.45 (q, *J* = 5.6 Hz, 2H), 2.87 (ddd, *J* = 16.9, 13.8, 5.4 Hz, 1H), 2.60 – 2.54 (m, 1H), 2.04 – 1.96 (m, 1H);‡ ^{13}C NMR (126 MHz, DMSO-d_6) δ 172.7, 169.9, 169.4, 166.8, 166.2, 165.2, 164.2, 161.0, 159.5 (d, *J* = 244.1 Hz), 159.4, 157.9, 157.2, 155.8, 144.6, 141.3, 136.9, 135.1, 134.5, 133.2, 131.6, 131.2, 130.9, 130.2, 127.7, 123.4, 119.9, 116.3, 115.3, 114.3, 110.5, 108.0, 101.4, 70.2, 69.85, 69.83, 69.7, 69.0, 68.8, 68.7, 56.3, 55.7, 49.4, 48.7, 38.9, 30.9, 22.0; HRMS calc. for $C_{48}H_{46}{}^{35}ClFN_7O_{11}$ $[M+H]^+$ 950.2922, found 950.2923.

‡CH obscured by DMSO-d_6 residual solvent peak but observed by HSQC. CH and OCH_3 peaks are not observed due to broadening.

Synthesis of 4-[[9-chloro-7-(2-fluoro-6-methoxy-phenyl)-5*H*-pyrimido[5,4-*d*][2]benzazepin-2-yl]amino]-*N*-[3-[3-[3-[2-(2,6-dioxo-3-piperidyl)-1,3-dioxo-isoindolin-4-yl]oxypropoxy]propoxy]propyl]-2-methoxy-benzamide (43)

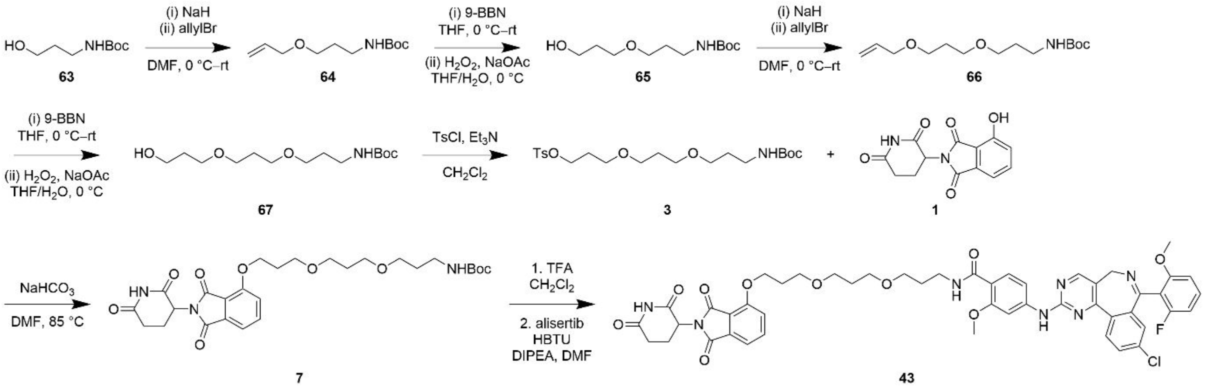

***tert*-Butyl *N*-(3-allyloxypropyl)carbamate (64)**

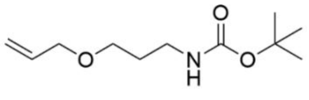

NaH (60% w/w in mineral oil, 97 mg, 2.4 mmol, 1.2 eq) was added to a solution of *tert*-butyl-*N*-(3-hydroxypropyl)carbamate (**63**) (355 mg, 2.0 mmol, 1.0 eq) in DMF (6.8 mL) under an atmosphere of N_2 at 0 °C. The solution was stirred at 0 °C for 15 min then allyl bromide (0.21 mL, 2.4 mmol, 1.2 eq) was added dropwise and the solution was stirred at room temperature for 19 h. H_2O (15 mL) was added, and the solution was extracted with EtOAc (3 × 25 mL). The combined organic layers were washed with H_2O (2 × 20 mL) and brine (10 mL), dried over Na_2SO_4, filtered, and concentrated. The residue was adsorbed onto silica and purified by normal-phase flash chromatography (0–35% EtOAc/cyclohexane) to afford the title compound (186 mg, 43%) as a colourless oil. ^1H NMR (600 MHz, chloroform-d) δ 5.95 – 5.85 (m, 1H), 5.27 (dt, *J* = 17.3, 1.4 Hz, 1H), 5.18 (dt, *J* = 10.5, 1.4 Hz, 1H), 4.82 (s, 1H), 3.96 (dd, *J* = 5.5, 1.4 Hz, 2H), 3.50 (t, *J* = 6.0 Hz, 2H), 3.29 – 3.18 (m, 2H), 1.77 (p, *J* = 6.3 Hz, 2H), 1.44 (s, 9H); ^{13}C NMR (151 MHz, chloroform-d) δ 156.2, 134.9, 117.0, 79.2, 72.0, 68.7, 38.9, 30.0, 28.6.

tert-Butyl N-[3-(3-hydroxypropoxy)propyl]carbamate (65)

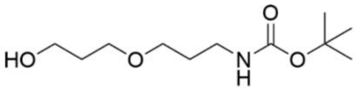

9-Borabicyclo[3.3.1]nonane (0.5 M in THF, 4.1 mL, 2.1 mmol, 2.5 eq) was added to a solution of *tert*-butyl *N*-(3-allyloxypropyl)carbamate (**64**) (178 mg, 0.83 mmol,1.0 eq) in THF (1.5 mL) under an atmosphere of N_2 at 0 °C. The solution was stirred at room temperature for 4.5 h then cooled to 0 °C and H_2O (0.3 mL) and THF (1.5 mL) were added. A mixture of H_2O_2 (30% v/v in H_2O, 3.6 mL) and NaOAc (20% w/v in H_2O, 4.8 mL) was added, and the mixture was extracted with EtOAc (3 × 10 mL). The combined organic layers were washed with brine (10 mL), dried over Na_2SO_4, filtered, and concentrated. The residue was adsorbed onto silica and purified by normal-phase flash chromatography (0–80% EtOAc/cyclohexane) to the title compound (113 mg, 59%) as a colourless oil. ^{1}H NMR (600 MHz, chloroform-*d*) δ 4.97 – 4.51 (m, 1H), 3.78 (t, *J* = 5.7 Hz, 2H), 3.59 (t, J = 5.8 Hz, 2H), 3.49 (t, *J* = 5.9 Hz, 2H), 3.22 (t, *J* = 6.5 Hz, 2H), 2.09 (s, 1H), 1.81 (p, *J* = 5.7 Hz, 2H), 1.74 (p, *J* = 6.1 Hz, 2H), 1.44 (s, 9H); ^{13}C NMR (151 MHz, chloroform-*d*) δ 156.3, 79.5, 69.4, 69.1, 61.2, 38.5, 32.2, 30.0, 28.6.

tert-Butyl N-[3-(3-allyloxypropoxy)propyl]carbamate (66)

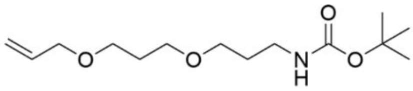

NaH (60% w/w in mineral oil, 15 mg, 0.39 mmol, 1.5 eq) was added to a solution of *tert*-butyl *N*-[3-(3-hydroxypropoxy)propyl]carbamate (**65**) (60 mg, 0.26 mmol, 1.0 eq) in THF (0.64 mL) under an atmosphere of N_2 at 0 °C. The solution was stirred at room temperature for 30 min then cooled to 0 °C and allyl bromide (33 µL, 0.39 mmol, 1.5 eq) was added dropwise. The solution was stirred at room temperature for three days then poured into ice-water (15 mL). The solution was extracted with EtOAc (3 × 20 mL), and the combined organic layers were washed with H_2O (10 mL) and brine (10 mL), dried over Na_2SO_4, filtered, and concentrated. The residue was adsorbed onto silica and purified by normal-phase flash chromatography (0–50% EtOAc/cyclohexane) to afford the title compound (39 mg, 56%) as a colourless oil. ^{1}H

NMR (600 MHz, chloroform-*d*) δ 5.91 (ddt, *J* = 17.3, 10.4, 5.6 Hz, 1H), 5.27 (dq, *J* = 17.2, 1.7 Hz, 1H), 5.19 – 5.15 (m, 1H), 4.89 (s, 1H), 3.97 (dt, *J* = 5.6, 1.5 Hz, 2H), 3.56 – 3.46 (m, 6H), 3.22 (q, *J* = 6.3 Hz, 2H), 1.85 (p, *J* = 6.3 Hz, 2H), 1.74 (p, *J* = 6.2 Hz, 2H), 1.44 (s, 9H); ^{13}C NMR (151 MHz, chloroform-*d*) δ 156.1, 135.1, 117.0, 79.1, 72.0, 69.5, 68.1, 67.4, 38.9, 30.2, 29.8, 28.6.

tert-Butyl N-[3-[3-(3-hydroxypropoxy)propoxy]propyl]carbamate (67)

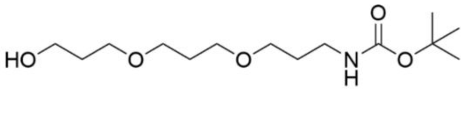

9-Borabicyclo[3.3.1]nonane (0.5 M in THF, 0.37 mL, 0.19 mmol, 1.5 eq) was added to a solution of *tert*-butyl *N*-[3-(3-allyloxypropoxy)propyl]carbamate (**66**) (34 mg, 0.12 mmol, 1.0 eq) in THF (0.22 mL) under an atmosphere of N_2 at 0 °C. The solution was stirred at room temperature for four hours then 9-borabicyclo[3.3.1]nonane (0.5 M in THF, 0.37 mL, 0.19 mmol, 1.5 eq) was added and the solution was stirred at room temperature for a further 90 min. The solution was cooled to 0 °C and H_2O (0.10 mL), THF (0.22 mL), and a mixture of H_2O_2 (30% v/v in H_2O, 0.30 mL) and NaOAc (20% w/v in H_2O, 0.4 mL) were added. The mixture was extracted with EtOAc (3 × 10 mL), and the combined organic layers were washed with brine (10 mL), dried over Na_2SO_4, filtered, and concentrated. The residue was adsorbed onto silica and purified by normal-phase flash chromatography (0–50% EtOAc/cyclohexane) to afford the title compound (25 mg, 69%) as a colourless oil. ^{1}H NMR (600 MHz, chloroform-*d)* δ 4.96 (s, 1H), 3.77 (q, *J* = 5.3 Hz, 2H), 3.63 (t, *J* = 5.7 Hz, 2H), 3.53 (t, *J* = 6.2 Hz, 2H), 3.48 (q, *J* = 5.9 Hz, 4H), 3.22 (q, *J* = 6.3 Hz, 2H), 1.86 – 1.80 (m, 4H), 1.74 (p, *J* = 6.1 Hz, 2H), 1.44 (s, 9H); ^{13}C NMR (151 MHz, chloroform-*d*) δ 156.2, 79.1, 70.6, 69.4, 68.4, 68.0, 62.4, 38.8, 32.1, 30.1, 29.8, 28.6.

3-[3-[3-(*tert*-butoxycarbonylamino)propoxy]propoxy]propyl 4-methyl benzenesulfonate (3)

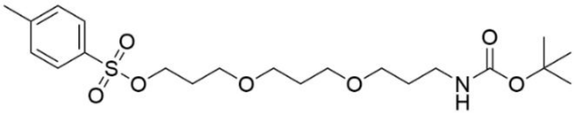

4-Dimethylaminopyridine (0.84 mg, 0.0069 mmol, 0.1 eq), triethylamine (29 µL, 0.210 mmol, 3.0 eq), and 4-toluenesulfonyl chloride (17 mg, 0.089 mmol, 1.3 eq) were added to a solution of *tert*-butyl *N*-[3-[3-(3-hydroxypropoxy)propoxy]propyl]carbamate (**67**) (20 mg, 0.069 mmol, 1.0 eq) in CH_2Cl_2 (0.57 mL) under an atmosphere of Ar. The solution was stirred at room temperature for 22 h then quenched with H_2O (10 mL). The solution was extracted with EtOAc (3 × 20 mL), and the combined organic layers were washed with saturated aqueous $NaHCO_3$ (10 mL), saturated aqueous NH_4Cl (10 mL), and brine (10 mL), dried over Na_2SO_4, filtered, and concentrated. The residue was adsorbed onto silica and purified by normal-phase flash chromatography (0–60% EtOAc/cyclohexane) to afford the title compound (19 mg, 62%) as a colourless oil which was immediately used in the subsequent step.

***tert*-Butyl *N*-[3-[3-[3-[2-(2,6-dioxo-3-piperidyl)-1,3-dioxo-isoindolin-4-yl]oxypropoxy]propoxy]propyl]carbamate (7)**

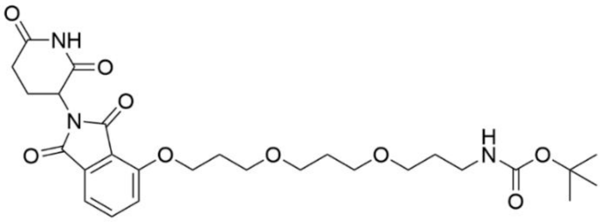

A mixture of 2-(2,6-dioxopiperidin-3-yl)-4-hydroxyisoindoline-1,3-dione (**1**) (12 mg, 0.044 mmol, 1.0 eq), 3-[3-[3-(*tert*-butoxycarbonylamino)propoxy]propoxy]propyl 4-methylbenzenesulfonate (**3**) (19 mg, 0.044 mmol, 1.0 eq), and $NaHCO_3$ (7.4 mg, 0.088 mmol, 2.0 eq) in DMF (0.29 mL) was stirred at 85 °C for 19 h then cooled to room temperature and diluted with H_2O (10 mL). The solution was extracted with EtOAc (3 × 20 mL), and the combined organic layers were washed with saturated aqueous $NaHCO_3$ (10 mL), H_2O (10 mL), and brine (10 mL), dried over Na_2SO_4, filtered, and concentrated. The residue was adsorbed onto silica and purified by normal-phase flash chromatography (0–8% MeOH/CH_2Cl_2) to afford the title compound (19 mg, 79%) as an off-white amorphous solid. The compound was isolated with impurities.

4-[[9-Chloro-7-(2-fluoro-6-methoxy-phenyl)-5*H*-pyrimido[5,4-*d*][2]benzazepin-2-yl]amino]-*N*-[3-[3-[3-[2-(2,6-dioxo-3-piperidyl)-1,3-dioxo-isoindolin-4-yl]oxypropoxy]propoxy]propyl]-2-methoxy-benzamide (43)

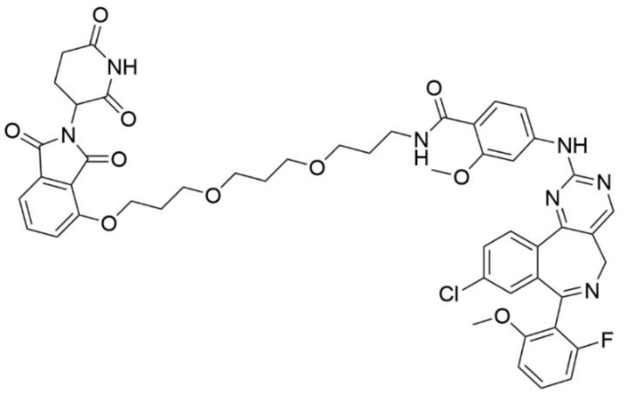

Trifluoroacetic acid (0.05 mL, 0.66 mmol, 20 eq) was added to a solution of *tert*-butyl *N*-[3-[3-[3-[2-(2,6-dioxo-3-piperidyl)-1,3-dioxo-isoindolin-4-yl]oxypropoxy]propoxy]propyl]carbamate (**7**) (18 mg, 0.033 mmol, 1.0 eq) in CH_2Cl_2 (0.66 mL) and the solution was stirred at room temperature for 5.5 h then concentrated. The residue was dissolved in DMF (1.3 mL), *O*-(benzotriazol-1-yl)-*N*,*N*,*N*',*N*'-tetramethyluronium hexafluorophosphate (12 mg, 0.033 mmol, 1.0 eq) was added, and the mixture was stirred until everything was dissolved. Alisertib (16 mg, 0.031 mmol, 0.95 eq) and *N*,*N*-diisopropylethylamine (23 µL, 0.13 mmol, 4.0 eq) were added and the solution was stirred at room temperature for 20 h then diluted with EtOAc (40 mL). The solution was washed with H_2O (20 mL) and brine (10 mL) then adsorbed onto silica and purified by normal-phase flash chromatography (0–10% MeOH/CH_2Cl_2) then reversed-phase flash chromatography (50–100% MeOH containing 0.1% HCO_2H/H_2O containing 0.1% HCO_2H) to afford the title compound (11 mg, 34%) as a white amorphous solid. ^1H NMR (600 MHz, DMSO-d_6) δ 11.09 (s, 1H), 10.16 (s, 1H), 8.71 (s, 1H), 8.29 (d, *J* = 8.5 Hz, 1H), 8.05 (t, *J* = 5.6 Hz, 1H), 7.94 (s, 1H), 7.83 – 7.75 (m, 3H), 7.49 (d, *J* = 8.6 Hz, 1H), 7.45 – 7.38 (m, 3H), 7.21 (br s, 1H), 7.02 – 6.72 (br m, 2H), 5.07 (dd, *J* = 12.9, 5.5 Hz, 1H), 4.23 (t, *J* = 6.2 Hz, 2H), 3.91 (s, 3H), 3.54 (t, *J* = 6.2 Hz, 2H), 3.45 – 3.37 (m, 6H), 2.87 (ddd, *J* = 17.0, 13.9, 5.7 Hz, 1H), 2.57 (ddd, *J* = 17.0, 4.4, 2.4 Hz, 1H), 2.04 – 1.93 (m, 3H), 1.76 – 1.68 (m, 4H);[§] ^{13}C NMR (151 MHz, DMSO-d_6) δ 172.8, 170.0, 166.8, 165.3, 164.3, 160.9, 160.5 – 158.3 (m), 159.4, 157.7, 157.2, 156.4,

[§]CH obscured by DMSO-d_6 residual solvent peak and CH_2 obscured by water peak but observed by HSQC spectra. CH_2 and OCH_3 not observed due to broadening.

155.9, 144.4, 137.7, 137.0, 135.4, 134.6, 133.2, 131.4, 131.1, 130.9, 130.3, 127.7, 123.4, 119.7, 116.3, 115.2, 115.0, 110.4, 108.0, 101.4, 68.3, 67.2, 67.1, 66.2, 65.9, 56.3, 55.7, 49.5, 48.7, 36.7, 30.9, 29.6, 29.4, 28.8, 22.0; HRMS calc. for $C_{49}H_{48}{}^{35}ClFN_7O_{10}$ $[M+H]^+$ 948.3135, found 948.3113.

Synthesis of 4-[[9-chloro-7-(2-fluoro-6-methoxy-phenyl)-5*H*-pyrimido[5,4-*d*][2]benzazepin-2-yl]amino]-*N*-[5-[5-[2-(2,6-dioxo-3-piperidyl)-1,3-dioxo-isoindolin-4-yl]oxypentoxy]pentyl]-2-methoxy-benzamide (44)

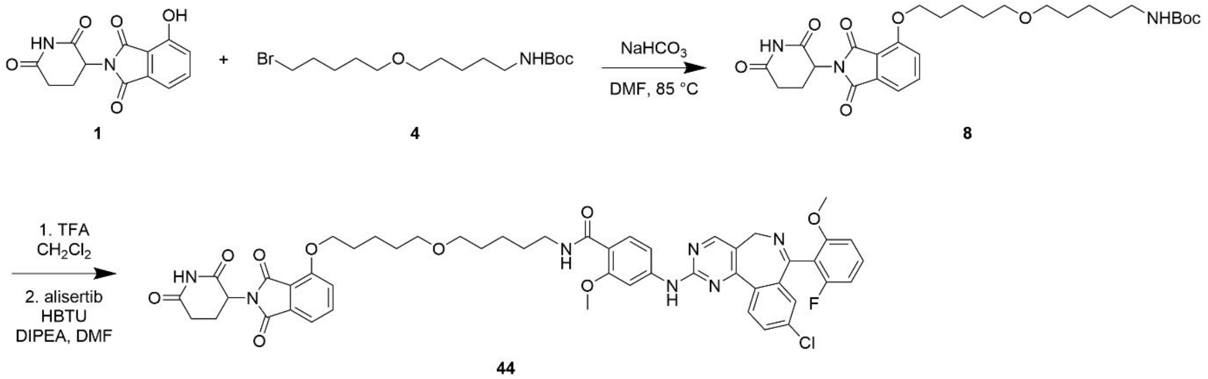

***tert*-Butyl *N*-[5-[5-[2-(2,6-dioxo-3-piperidyl)-1,3-dioxo-isoindolin-4-yl]oxy pentoxy]pentyl]carbamate (8)**

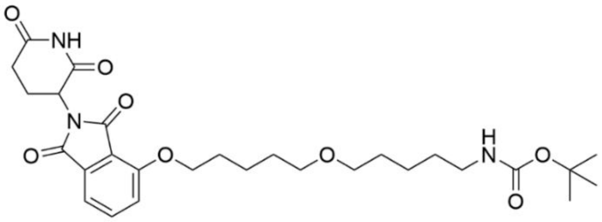

A mixture of 2-(2,6-dioxopiperidin-3-yl)-4-hydroxyisoindoline-1,3-dione (**1**) (30 mg, 0.11 mmol, 1.0 eq), Common Intermediate A (**4**) (39 mg, 0.11 mmol, 1.0 eq), and $NaHCO_3$ (18 mg, 0.22 mmol, 2.0 eq) in DMF (0.73 mL) was stirred at 60 °C for 20 h. The solution was diluted with EtOAc (30 mL) and washed with water (10 mL), saturated aqueous NaHCO3 (10 mL), and brine (10 mL), then adsorbed onto silica and purified by normal-phase flash chromatography (0–5% MeOH/CH_2Cl_2) to afford the title compound (39 mg, 65%) as a yellow oil. ^{1}H NMR (500 MHz, chloroform-*d*) δ 8.12 (s, 1H), 7.67 (dd, *J* = 8.5, 7.3 Hz, 1H), 7.45 (d, *J* = 7.3 Hz, 1H), 7.21 (d, *J* = 8.4 Hz, 1H), 4.95 (dd, *J* = 12.3, 5.4 Hz, 1H), 4.56 (s, 1H), 4.18 (t, *J* = 6.5 Hz, 2H), 3.48 – 3.34 (m, 4H), 3.19 – 3.04 (m, 2H), 2.94 – 2.67 (m, 3H), 2.17 – 2.06 (m, 1H), 1.91 (p, *J* = 6.8 Hz, 2H), 1.70 – 1.62 (m, 2H), 1.62 – 1.53 (m, 4H), 1.52 – 1.40 (m, 11H), 1.40 – 1.32 (m, 2H); ^{13}C NMR (126 MHz, chloroform-*d*) δ 171.0, 168.2, 167.2, 165.8, 156.9, 156.2, 136.6, 134.0, 119.1, 117.3, 115.9, 79.2, 70.9, 70.7, 69.5, 49.2, 40.7, 31.6, 30.0, 29.5,

28.9, 28.6, 23.6, 22.8; HRMS calc. for $C_{28}H_{39}NaN_3O_8$ $[M+Na]^+$ 568.2635, found 568.2639.

4-[[9-Chloro-7-(2-fluoro-6-methoxy-phenyl)-5*H*-pyrimido[5,4-*d*][2]benzazepin-2-yl]amino]-*N*-[5-[5-[2-(2,6-dioxo-3-piperidyl)-1,3-dioxo-isoindolin-4-yl]oxypentoxy]pentyl]-2-methoxy-benzamide (44)

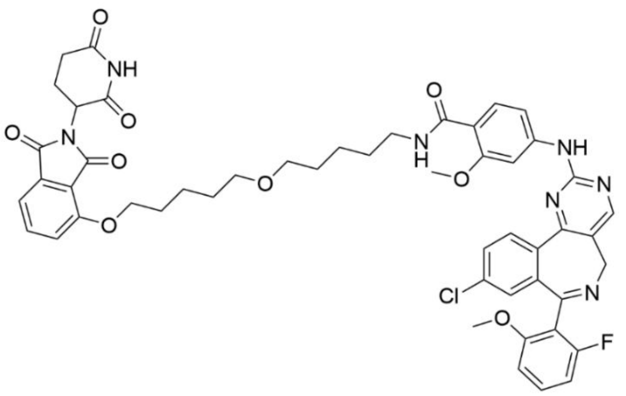

tert-Butyl *N*-[5-[5-[2-(2,6-dioxo-3-piperidyl)-1,3-dioxo-isoindolin-4-yl]oxypentoxy] pentyl]carbamate (**8**) (30 mg, 0.05 mmol, 1.0 eq) was dissolved in a mixture of CH_2Cl_2 (0.8 mL) and trifluoroacetic acid (0.20 mL) and stirred at room temperature for two hours then concentrated. The residue was dissolved in DMF (2.5 mL), *O*-(benzotriazol-1-yl)-*N*,*N*,*N'*,*N'*-tetramethyluronium hexafluorophosphate (21 mg, 0.05 mmol, 1.0 eq) was added, and the solution was stirred until everything was dissolved. Alisertib (28 mg, 0.05 mmol, 1.0 eq) and *N*,*N*-diisopropylethylamine (38 µL, 0.22 mmol, 4.0 eq) were added and the solution was stirred at room temperature for 18 h. The solvent was removed on a Biotage V10 and the residue was adsorbed onto silica and purified by normal-phase flash chromatography (0–8% MeOH/CH_2Cl_2) then reversed-phase flash chromatography (30–100% MeOH containing 0.1% HCO_2H/H_2O containing 0.1% HCO_2H) to afford the title compound (28 mg, 53%) as an off-white amorphous solid. ^1H NMR (500 MHz, DMSO-d_6) δ 11.09 (s, 1H), 10.15 (s, 1H), 8.71 (s, 1H), 8.29 (d, *J* = 8.4 Hz, 1H), 8.01 (t, *J* = 5.7 Hz, 1H), 7.95 (s, 1H), 7.84 – 7.74 (m, 3H), 7.48 (d, *J* = 8.6 Hz, 1H), 7.45 – 7.37 (m, 3H), 7.22 (s, 1H), 6.88 (br s, 2H), 5.07 (dd, *J* = 12.8, 5.5 Hz, 1H), 4.18 (t, *J* = 6.5 Hz, 2H), 3.91 (s, 3H), 3.36 (td, *J* = 6.4, 4.5 Hz, 4H), 3.27 (q, *J* = 6.9 Hz, 2H), 2.87 (ddd, *J* = 16.8, 13.8, 5.5 Hz, 1H), 2.61 – 2.54 (m, 1H), 2.05 – 1.98

(m, 1H), 1.76 (p, J = 6.7 Hz, 2H), 1.61 – 1.44 (m, 8H), 1.39 – 1.30 (m, 2H);** ^{13}C NMR (126 MHz, DMSO-d_6) δ 172.8, 169.9, 166.8, 165.3, 164.3, 160.5, 159.4, 158.6, 157.7, 157.1, 156.0, 144.3, 137.0, 134.6, 133.2, 131.4, 130.8, 127.4, 123.4, 119.7, 116.2, 115.1, 110.4, 108.0, 101.4, 79.2, 69.9, 69.84, 68.76, 56.3, 55.7, 49.4, 48.7, 31.0, 29.1, 29.0, 28.9, 28.2, 23.3, 22.2, 22.0; HRMS calc. for $C_{50}H_{50}{}^{35}ClFN_7O_9$ $[M+H]^+$ 946.3337, found 946.3359.

**CH obscured under DMSO-d_6 residual solvent peak. CH_2 and OCH_3 peaks are not observed due to broadening.

Synthesis of 4-[[9-chloro-7-(2-fluoro-6-methoxy-phenyl)-5*H*-pyrimido[5,4-*d*][2]benzazepin-2-yl]amino]-*N*-[5-[6-[2-(2,6-dioxo-3-piperidyl)-1,3-dioxo-isoindolin-4-yl]hexoxy]pentyl]-2-methoxy-benzamide (45)

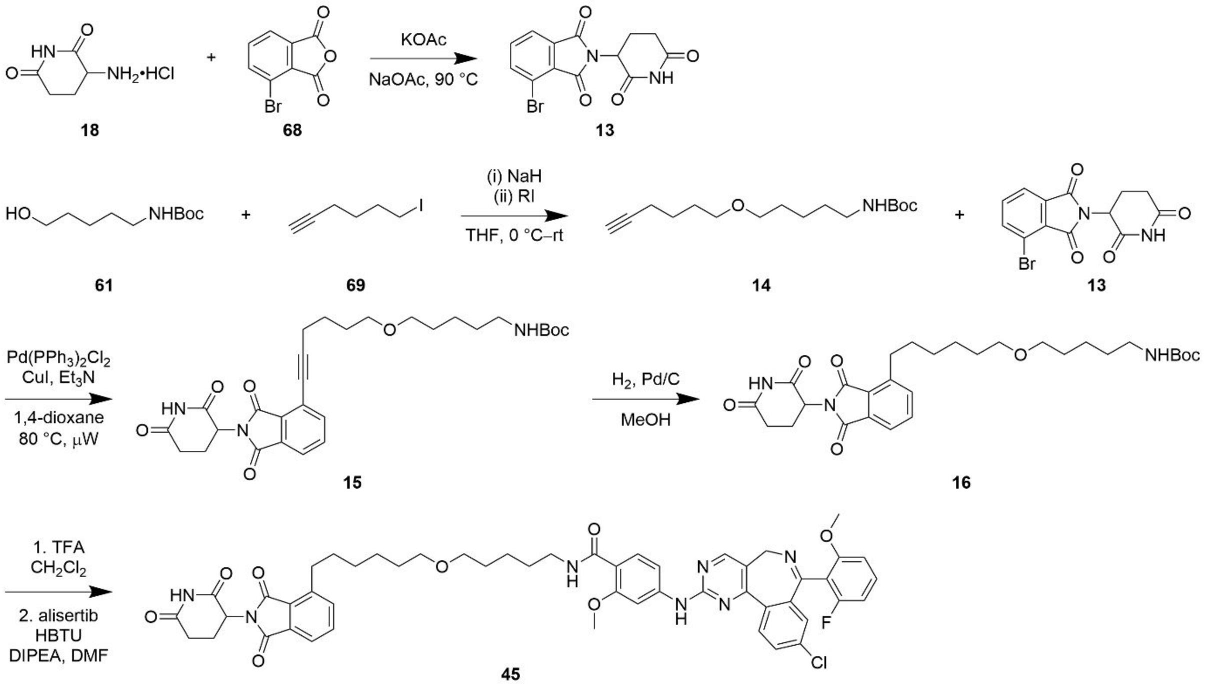

4-Bromo-2-(2,6-dioxo-3-piperidyl)isoindoline-1,3-dione (13)

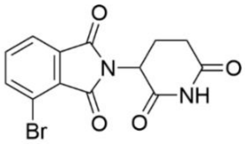

A solution of 3-bromophthalic anhydride (**68**) (300 mg, 1.3 mmol, 1.0 eq), 3-aminopiperidine-2,6-dione hydrochloride (**18**) (218 mg, 1.3 mmol, 1.0 eq), and KOAc (406 mg, 4.1 mmol, 3.1 eq) in AcOH (4.4 mL) was stirred at 90 °C for 19 h then cooled to room temperature and concentrated. H_2O (10 mL) was added to the residue, and the mixture was filtered. The solid was washed with H_2O (5 mL) and MeCN (5 mL) then dried under high-vacuum to afford the title compound (417 mg, 93%) as a grey amorphous solid. ^{1}H NMR (600 MHz, DMSO-d_6) δ 11.15 (s, 1H), 8.07 (dd, J = 8.1, 0.8 Hz, 1H), 7.94 (dd, J = 7.4, 0.8 Hz, 1H), 7.78 (dd, J = 8.1, 7.4 Hz, 1H), 5.17 (dd, J = 13.0, 5.4 Hz, 1H), 2.89 (ddd, J = 17.2, 13.9, 5.4 Hz, 1H), 2.61 (ddd, J = 17.2, 4.5, 2.4

Hz, 1H), 2.57 – 2.47 (m, 1H), 2.07 (dtd, J = 13.0, 5.4, 2.4 Hz, 1H);†† ^{13}C NMR (151 MHz, DMSO-d_6) δ 172.8, 169.7, 165.6, 165.2, 139.2, 136.3, 133.7, 128.7, 122.9, 117.6, 49.2, 30.9, 21.8; HRMS calc. for $C_{13}H_{10}{}^{79}BrN_2O_4$ [M+H]$^+$ 336.9824, found 336.9825.

***tert*-Butyl *N*-(5-hex-5-ynoxypentyl)carbamate (14)**

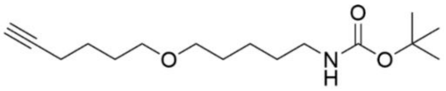

NaH (60% w/w in mineral oil, 70.0 mg, 1.7 mmol, 1.1 eq) was added to a solution of *tert*-butyl *N*-(5-hydroxypentyl)carbamate (**61**) (323 mg, 1.6 mmol, 1.0 eq) in THF (3.5 mL) under an atmosphere of N_2 at 0 °C. The solution was stirred at room temperature for 30 minutes then cooled to 0 °C and 6-iodo-1-hexyne (**69**) (0.23 mL, 1.7 mmol, 1.1 eq) was added dropwise. The solution was stirred at room temperature for 19 h then poured into ice-water (15 mL). The solution was extracted with EtOAc (3 × 20 mL), and the combined organic layers were washed with H_2O (10 mL) and brine (10 mL), adsorbed onto silica and purified by normal-phase flash chromatography (0–50% EtOAc/cyclohexane) to afford the title compound (96 mg, 21%) as a colourless oil. ^{1}H NMR (600 MHz, chloroform-d) δ 4.53 (s, 1H), 3.45 – 3.37 (m, 4H), 3.11 (q, J = 6.7 Hz, 2H), 2.22 (td, J = 7.1, 2.7 Hz, 2H), 1.94 (t, J = 2.7 Hz, 1H), 1.68 (dq, J = 8.6, 6.5 Hz, 2H), 1.59 (dtd, J = 15.0, 8.0, 7.6, 5.1 Hz, 4H), 1.53 – 1.46 (m, 2H), 1.44 (s, 9H), 1.40 – 1.32 (m, 2H); ^{13}C NMR (151 MHz, chloroform-d) δ 156.1, 84.5, 79.2, 70.8, 70.4, 68.5, 40.7, 30.0, 29.5, 28.9, 28.6, 25.4, 23.7, 18.4; HRMS calc. for $C_{16}H_{29}NaNO_3$ [M+Na]$^+$ 306.2046, found 306.2037.

††CH obscured under DMSO-d_6 residual solvent peak.

***tert*-Butyl *N*-[5-[6-[2-(2,6-dioxo-3-piperidyl)-1,3-dioxo-isoindolin-4-yl]hex-5-ynoxy]pentyl]carbamate (15)**

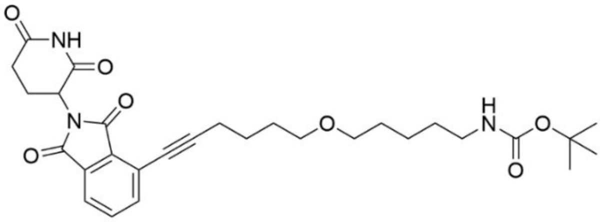

Triethylamine (0.31 mL, 2.2 mol, 18 eq) was added to a mixture of 4-bromo-2-(2,6-dioxo-3-piperidyl)isoindoline-1,3-dione (**13**) (42 mg, 0.12 mmol, 1.0 eq), *tert*-butyl *N*-(5-hex-5-ynoxypentyl)carbamate (**14**) (35 mg, 0.12 mmol, 1.0 eq), bis(triphenylphosphine)palladium (ii) chloride (8.7 mg, 0.012 mmol, 0.1 eq), and CuI (2.4 mg, 0.012 mmol, 0.1 eq) in DMF (0.31 mL) under an atmosphere of N_2. The solution was stirred at 80 °C under microwave irradiation for one hour then filtered through Celite and concentrated. The residue was adsorbed onto silica and purified by normal-phase flash chromatography (0–10% MeOH/CH_2Cl_2) then normal-phase flash chromatography (0–5% MeOH/CH_2Cl_2) to afford the title compound (50 mg, 75%) as a yellow amorphous solid. The compound was isolated with impurities.

***tert*-Butyl *N*-[5-[6-[2-(2,6-dioxo-3-piperidyl)-1,3-dioxo-isoindolin-4-yl]hexoxy] pentyl]carbamate (16)**

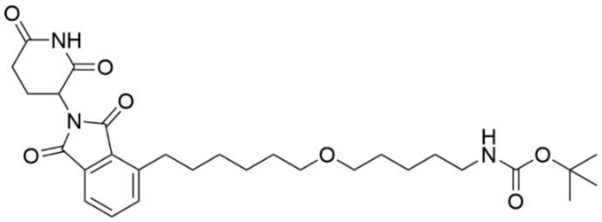

Pd (10% w/w on carbon, 7.6 mg, 0.0072 mmol, 0.3 eq) was added to a solution of *tert*-butyl *N*-[5-[6-[2-(2,6-dioxo-3-piperidyl)-1,3-dioxo-isoindolin-4-yl]hex-5-ynoxy]pentyl] carbamate (**15**) (12.9 mg, 0.024 mmol, 1.0 eq) in MeOH (0.68 mL) under an atmosphere of N_2. The vessel was purged with H_2, and the solution was stirred at room temperature under an atmosphere of H_2 for 22 h. The reaction vessel was purged with N_2, and the solution was filtered through Celite and concentrated to afford the title

compound (13.0 mg, 100%) as a colourless oil. The product was immediately carried on to the next step.

4-[[9-Chloro-7-(2-fluoro-6-methoxy-phenyl)-5*H*-pyrimido[5,4-*d*][2]benzazepin-2-yl]amino]-*N*-[5-[6-[2-(2,6-dioxo-3-piperidyl)-1,3-dioxo-isoindolin-4-yl]hexoxy]pentyl]-2-methoxy-benzamide (45)

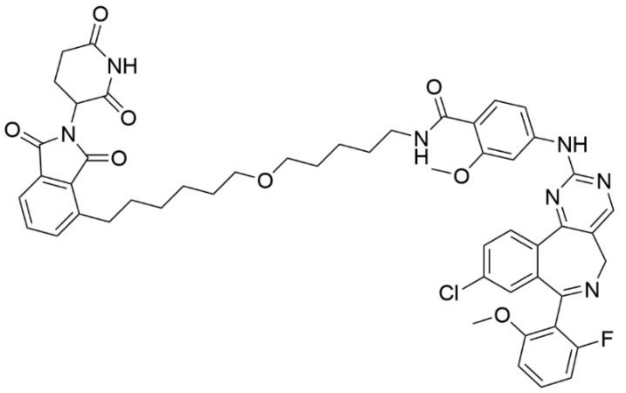

Trifluoroacetic acid (37 µL, 0.48 mmol, 20 eq) was added to a solution of *tert*-butyl *N*-[5-[6-[2-(2,6-dioxo-3-piperidyl)-1,3-dioxo-isoindolin-4-yl]hexoxy]pentyl]carbamate (**16)** (13.0 mg, 0.024 mmol, 1.0 eq) in CH_2Cl_2 (0.48 mL). The solution was stirred at room temperature for two hours then concentrated. The residue was dissolved in DMF (0.96 mL), *O*-(benzotriazol-1-yl)-*N*,*N*,*N'*,*N'*-tetramethyluronium hexafluorophosphate (9.1 mg, 0.024 mmol, 1.0 eq) was added, and the mixture was stirred until everything was dissolved. Alisertib (12 mg, 0.023 mmol, 0.95 eq) and *N*,*N*-diisopropylethylamine (17 µL, 0.096 mmol, 4.0 eq) were added and the solution was stirred at room temperature for 18 h then diluted with EtOAc (30 mL). The solution was washed with saturated aqueous NH_4Cl (15 mL), saturated aqueous $NaHCO_3$ (15 mL), H_2O (15 mL), and brine (15 mL) then adsorbed onto silica and purified by normal-phase flash chromatography (0–9% MeOH/CH_2Cl_2) then reversed-phase flash chromatography (50–100% MeOH containing 0.1% HCO_2H/H_2O containing 0.1% HCO_2H) to afford the title compound (8.0 mg, 34%) as a white amorphous solid. ^{1}H NMR (600 MHz, DMSO-d_6) δ 11.11 (s, 1H), 10.16 (s, 1H), 8.71 (s, 1H), 8.29 (d, *J* = 8.5 Hz, 1H), 8.01 (t, *J* = 5.7 Hz, 1H), 7.95 (s, 1H), 7.85 – 7.76 (m, 2H), 7.77 – 7.69 (m, 2H), 7.66 (dd, *J* = 7.0, 1.7 Hz, 1H), 7.45 – 7.37 (m, 2H), 7.21 (br s, 1H), 7.02 – 6.70 (br m, 2H), 5.12 (dd, *J* = 12.9, 5.5 Hz, 1H), 4.87 (br s, 1H), 3.91 (s, 3H), 3.35 – 3.30 (m, 4H), 3.27 (q, *J* = 6.7

Hz, 2H), 3.03 – 2.97 (m, 2H), 2.88 (ddd, *J* = 17.0, 13.9, 5.4 Hz, 1H), 2.63 – 2.57 (m, 1H), 2.56 – 2.51 (m, 1H), 2.08 – 2.00 (m, 1H), 1.63 – 1.42 (m, 8H), 1.39 – 1.25 (m, 6H);[‡‡] ^{13}C NMR (151 MHz, DMSO-d_6) δ 172.8, 169.9, 167.6, 167.0, 164.3, 160.9, 159.5 (d, *J* = 243.2 Hz), 159.4, 157.7, 157.2, 144.4, 142.6, 137.4, 136.0, 135.0, 134.6, 134.4, 131.8, 131.4, 131.2, 130.9, 130.2, 127.5, 123.4, 121.1, 115.1, 110.4, 108.5 – 107.4 (m), 101.4, 69.9, 69.9, 56.3, 55.7, 49.5, 48.8, 38.9, 30.9, 30.4, 30.3, 29.14, 29.07, 28.97, 28.7, 25.4, 23.3, 22.0; HRMS calc. for $C_{51}H_{52}{}^{35}ClFN_7O_8$ $[M+H]^+$ 944.3550, found 944.3531.

[‡‡]CH and OCH_3 not observed due to peak broadening.

Synthesis of 4-[[9-chloro-7-(2-fluoro-6-methoxy-phenyl)-5*H*-pyrimido[5,4-*d*][2]benzazepin-2-yl]amino]-*N*-[5-[4-[2-[2-(2,6-dioxo-3-piperidyl)-1,3-dioxo-isoindolin-5-yl]oxyethyl]piperazin-1-yl]pentyl]-2-methoxy-benzamide (46)

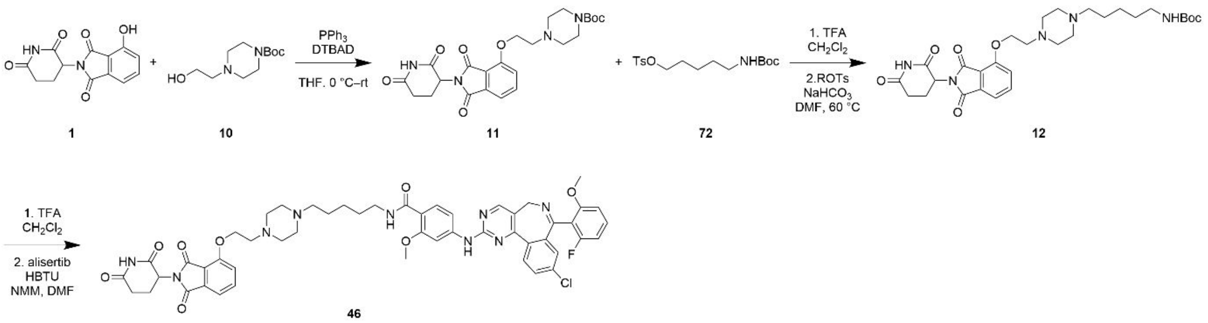

***tert*-Butyl 4-[2-[2-(2,6-dioxo-3-piperidyl)-1,3-dioxo-isoindolin-4-yl]oxyethyl] piperazine-1-carboxylate (11)**

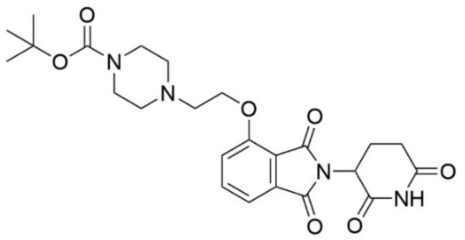

Di-*tert*-butylazodicarboxylate (278 mg, 1.2 mmol, 1.3 eq) was added to a solution of 2-(2,6-dioxopiperidin-3-yl)-4-hydroxyisoindoline-1,3-dione (**1**) (250 mg, 0.91 mmol, 1.0 eq), *tert*-butyl 4-(2-hydroxyethyl)piperazine-1-carboxylate (**10**) (210 mg, 0.91 mmol, 1.0 eq), and triphenylphosphine (310 mg, 1.2 mmol, 1.3 eq) in DMF (9.1 mL) under an atmosphere of N_2 at 0 °C. The solution was stirred at room temperature for 20 h then adsorbed onto silica and purified by normal-phase flash chromatography (0–9% MeOH/CH_2Cl_2) to afford the title compound (412 mg, 93%) as a pale-yellow amorphous solid. ^{1}H NMR (600 MHz, chloroform-*d*) δ 8.21 (s, 1H), 7.68 (dd, *J* = 8.4, 7.3 Hz, 1H), 7.47 (d, *J* = 7.3 Hz, 1H), 7.23 (d, *J* = 8.5 Hz, 1H), 4.95 (dd, *J* = 12.5, 5.4 Hz, 1H), 4.31 (t, *J* = 5.6 Hz, 2H), 3.44 (t, *J* = 5.0 Hz, 4H), 2.91 (t, *J* = 5.6 Hz, 2H), 2.88 – 2.69 (m, 3H), 2.58 (t, *J* = 5.1 Hz, 4H), 2.12 (m, 1H), 1.45 (s, 9H); ^{13}C NMR (151 MHz, $CDCl_3$) δ 171.0, 168.2, 167.1, 165.8, 156.4, 154.9, 136.7, 134.0, 119.3, 117.5, 116.3, 79.9, 68.0, 57.0, 53.7, 49.3, 42.7, 31.5, 28.6, 22.8; calc. for $C_{24}H_{31}N_4O_7$ [M+H]$^+$ 487.2193, found 487.2180.

5-(*tert*-Butoxycarbonylamino)pentyl 4-methylbenzenesulfonate (72)

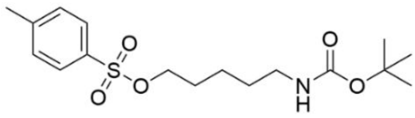

Triethylamine (0.190 mL, 1.4 mmol, 3.0 eq) and *p*-toluenesulfonyl chloride (112 mg, 0.59 mmol, 1.3 eq) were added to a solution of *tert*-butyl *N*-(5-hydroxypentyl)carbamate (**61**) (92.0 mg, 0.45 mmol, 1.0 eq) in CH_2Cl_2 (1.1 mL) under an atmosphere of N_2 at 0 °C. The solution was stirred at room temperature for two days then diluted with CH_2Cl_2 (30 mL). The solution was washed with saturated aqueous NH_4Cl (10 mL), saturated aqueous $NaHCO_3$ (10 mL), and brine (10 mL), then adsorbed onto silica and purified by normal-phase flash chromatography (0–15% EtOAc/CH_2Cl_2) to afford the title compound (114 mg, 70%) as a colourless oil. ^{1}H NMR (600 MHz, chloroform-*d*) δ 7.81 – 7.75 (m, 2H), 7.34 (d, *J* = 8.0 Hz, 2H), 4.48 (s, 1H), 4.01 (t, *J* = 6.4 Hz, 2H), 3.06 (q, *J* = 6.8 Hz, 2H), 2.45 (s, 3H), 1.70 – 1.62 (m, 2H), 1.46 – 1.38 (m, 11H), 1.37 – 1.29 (m, 2H); ^{13}C NMR (151 MHz, chloroform-*d*) δ 156.1, 144.9, 133.3, 130.0, 128.0, 79.3, 70.5, 40.4, 29.6, 28.6, 28.6, 22.8, 21.78; HRMS calc. for $C_{17}H_{27}NaNO_5S$ $[M+Na]^+$ 380.1508, found 380.1507.

***tert*-Butyl *N*-[5-[4-[2-[2-(2,6-dioxo-3-piperidyl)-1,3-dioxo-isoindolin-4-yl]oxyethyl] piperazin-1-yl]pentyl]carbamate (12)**

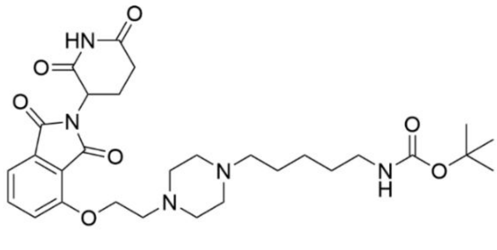

Trifluoroacetic acid (94 μL, 1.2 mmol, 10 eq) was added to a solution of *tert*-butyl 4-[2-[2-(2,6-dioxo-3-piperidyl)-1,3-dioxo-isoindolin-4-yl]oxyethyl]piperazine-1-carboxylate (**11**) (60 mg, 0.12 mmol, 1.0 eq) in CH_2Cl_2 (1.2 mL). The solution was stirred at room temperature for 20 h then concentrated. The residue was dissolved in DMF (1.2 mL) and 5-(*tert*-butoxycarbonylamino)pentyl 4-methylbenzenesulfonate (**72**) (44 mg, 0.12 mmol, 1.0 eq) and $NaHCO_3$ (41 mg, 0.49 mmol, 4.0 eq) were added. The solution was

stirred at 60 °C for 22 h then cooled to room temperature and purified by reversed-phase flash chromatography (60–100% MeOH containing 0.1% HCO_2H/H_2O containing 0.1% HCO_2H) to afford the title compound (18 mg, 22%) as a white amorphous solid. This compound was isolated as the diformate salt and isolated with impurities.

4-[[9-Chloro-7-(2-fluoro-6-methoxy-phenyl)-5*H*-pyrimido[5,4-*d*][2]benzazepin-2-yl]amino]-*N*-[5-[4-[2-[2-(2,6-dioxo-3-piperidyl)-1,3-dioxo-isoindolin-5-yl]oxyethyl]piperazin-1-yl]pentyl]-2-methoxy-benzamide (46)

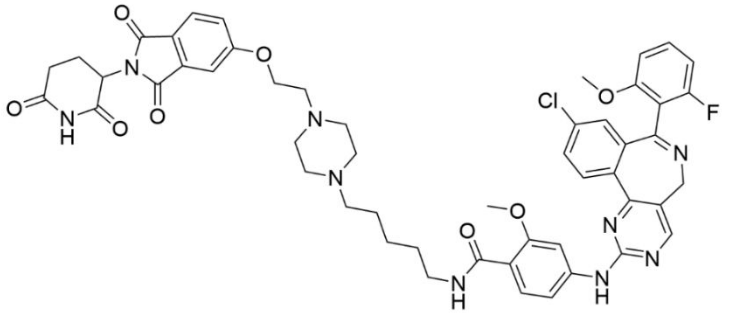

Trifluoroacetic acid (39 µL, 0.51 mmol, 20 eq) was added to a solution of *tert*-butyl *N*-[5-[4-[2-[2-(2,6-dioxo-3-piperidyl)-1,3-dioxo-isoindolin-4-yl]oxyethyl]piperazin-1-yl]pentyl]carbamate diformate (**12**) (17 mg, 0.026 mmol, 1.0 eq) in CH_2Cl_2 (0.5 mL) and the solution was stirred at room temperature for three hours. The solution was concentrated then the residue was dissolved in DMF (1.0 mL). *O*-(Benzotriazol-1-yl)-*N*,*N*,*N*',*N*'-tetramethyluronium hexafluorophosphate (9.7 mg, 0.026 mmol, 1.0 eq) and the solution was stirred until everything was dissolved. Alisertib (13 mg, 0.024 mmol, 0.95 eq) and *N*-methylmorpholine (14 µL, 0.130 mmol, 5.0 eq) were added and the solution was stirred at room temperature for 19 h then the solvent was removed with a flow of air. The residue was dissolved in DMSO and purified by reversed-phase flash chromatography (20–100% MeCN containing 0.1% HCO_2H/H_2O containing 0.1% HCO_2H) to afford the title compound (4.4 mg, 17%) as a yellow amorphous solid. This compound was isolated as the mono-formate salt. ^1H NMR (600 MHz, DMSO-d_6) δ 11.10 (s, 1H), 10.16 (s, 1H), 8.71 (s, 1H), 8.30 (d, J = 8.5 Hz, 1H), 8.16 (s, 1H), 8.02 (t, J = 5.7 Hz, 1H), 7.95 (br s, 1H), 7.84 – 7.77 (m, 3H), 7.52 (d, J = 8.5 Hz, 1H), 7.46 – 7.38 (m, 3H), 7.21 (br s, 1H), 7.02 – 6.73 (br m, 2H), 5.08 (dd, J = 12.9, 5.4 Hz, 1H), 4.88 (br s, 1H), 4.30 (t, J = 5.6 Hz, 2H), 3.92 (s, 3H), 3.29 – 3.25 (m, 2H, overlapping

with H_2O peak), 2.87 (ddd, *J* = 17.1, 13.9, 5.4 Hz, 1H), 2.74 (t, *J* = 5.6 Hz, 2H), 2.61 – 2.36 (m, 10H, overlapping with DMSO peak), 2.31 (t, *J* = 7.5 Hz, 2H), 2.02 (dtd, *J* = 13.0, 5.4, 2.4 Hz, 1H), 1.56 – 1.48 (m, 2H), 1.48 – 1.42 (m, 2H), 1.34 – 1.27 (m, 2H);[§§] ^{13}C NMR (151 MHz, DMSO-d_6) δ 172.8, 170.0, 166.8, 165.3, 164.3, 161.1, 159.6 (d, J = 246.4 Hz), 159.4, 157.7, 157.2, 155.8, 144.4, 137.7, 137.0, 135.2, 135.0, 134.5, 133.2, 131.4, 131.2, 130.9, 130.4, 127.1, 123.4, 120.0, 116.3, 115.3, 115.1, 110.4, 108.0, 101.5, 67.6, 57.7, 56.2, 55.7, 53.0, 52.7, 49.5, 48.7, 38.9, 31.0, 29.2, 25.8, 24.4, 22.0; HRMS calc. for $C_{51}H_{52}{}^{35}ClFN_9O_8$ $[M+H]^+$ 972.3611, found 972.3619.

[§§]CH and OCH_3 not observed due to peak broadening.

Synthesis of 4-[[9-chloro-7-(2-fluoro-6-methoxy-phenyl)-5*H*-pyrimido[5,4-*d*][2]benzazepin-2-yl]amino]-*N*-[5-[5-[2-(2,6-dioxo-3-piperidyl)-1-oxo-isoindolin-4-yl]oxypentoxy] pentyl]-2-methoxy-benzamide (47)

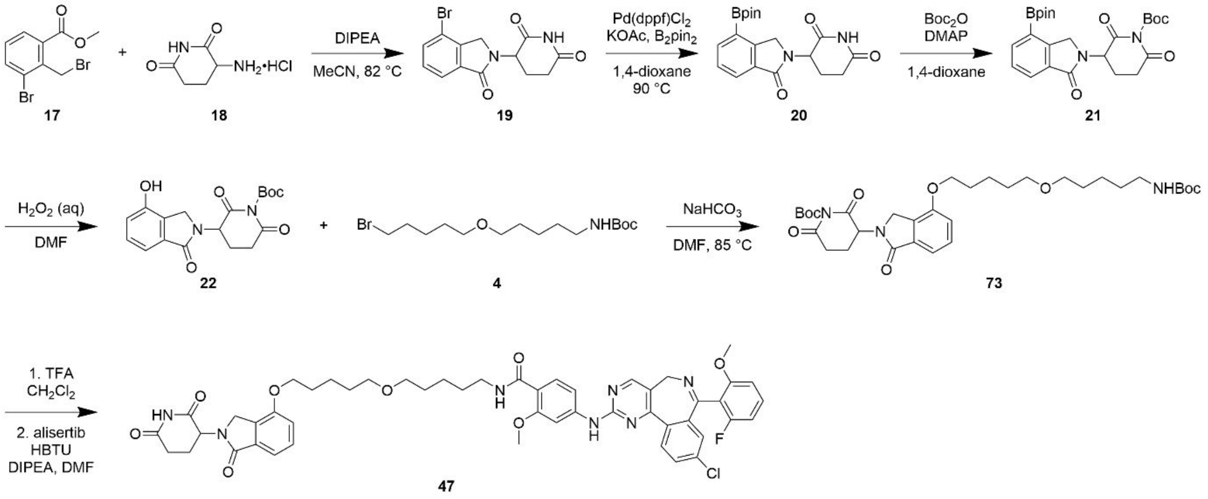

3-(4-Bromo-1-oxo-isoindolin-2-yl)piperidine-2,6-dione (19)

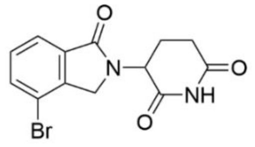

Prepared according to literature procedure.[7] A mixture of 3-aminopiperidine-2,6-dione hydrochloride (**18**) (513 mg, 3.1 mmol, 1.2 eq), methyl-3-bromo-2-(bromomethyl)benzoate (**17**) (0.450 mL, 2.6 mmol, 1.0 eq), and *N*,*N*-diisopropylethylamine (2.30 mL, 13 mmol, 5.0 eq) in MeCN (7.9 mL) was stirred at 82 °C for 16 h then cooled to room temperature. The mixture was filtered and the solid was washed with Et_2O (20 mL) and dried under a high vacuum to afford the title compound (681 mg, 81%) as a blue amorphous solid. ^{1}H NMR (600 MHz, DMSO-d_6) δ 11.01 (s, 1H), 7.87 (dd, *J* = 7.9, 0.9 Hz, 1H), 7.77 (dd, *J* = 7.5, 0.9 Hz, 1H), 7.51 (t, *J* = 7.7 Hz, 1H), 5.15 (dd, *J* = 13.3, 5.1 Hz, 1H), 4.42 (d, *J* = 17.5 Hz, 1H), 4.27 (d, *J* = 17.5 Hz, 1H), 2.91 (ddd, *J* = 17.3, 13.7, 5.4 Hz, 1H), 2.65 – 2.56 (m, 1H), 2.51 – 2.42 (m, 1H), 2.02 (dtd, *J* = 12.7, 5.3, 2.2 Hz, 1H);[***] ^{13}C NMR (151 MHz, DMSO-d_6) δ 172.8,

[***]CH obscured by DMSO-d_6 residual solvent peak

170.9, 167.2, 142.1, 134.6, 133.9, 130.5, 122.5, 117.3, 51.7, 48.0, 31.2, 22.3; HRMS calc. for $C_{13}H_{11}{}^{79}BrNaN_2O_3$ $[M+Na]^+$ 344.9851, found 344.9853.

3-[1-Oxo-4-(4,4,5,5-tetramethyl-1,3,2-dioxaborolan-2-yl)isoindolin-2-yl]piperidine-2,6-dione (20)

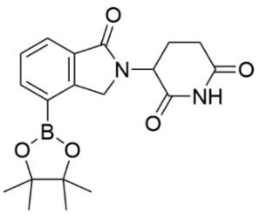

Prepared according to literature procedure.[8] Bis(pinacolato)diboron (979 mg, 3.9 mmol, 2.0 eq), [1,1'-bis(diphenylphosphino)ferrocene]dichloropalladium(II), complex with dichloromethane (157 mg, 0.19 mmol, 0.1 eq), and KOAc (378 mg, 3.9 mmol, 2.0 eq) were added to a solution of 3-(4-bromo-1-oxo-isoindolin-2-yl)piperidine-2,6-dione (**19**) (623 mg, 1.9 mmol, 1.0 eq) in 1,4-dioxane (5.8 mL) under an atmosphere of N_2. The reaction was stirred at 90 °C for 17 h then cooled to room temperature and filtered through Celite. The solution was adsorbed onto silica and purified by normal-phase flash chromatography (0–100% EtOAc/cyclohexane) to afford the title compound (566 mg, 79%) as an off-white amorphous solid. ^1H NMR (600 MHz, DMSO-d_6) δ 10.99 (s, 1H), 7.90 (dd, J = 7.3, 1.2 Hz, 1H), 7.85 (dd, J = 7.6, 1.2 Hz, 1H), 7.55 (t, J = 7.5 Hz, 1H), 5.13 (dd, J = 13.3, 5.2 Hz, 1H), 4.49 (s, 1H), 4.42 (d, J = 17.9 Hz, 1H), 2.90 (ddd, J = 17.3, 13.6, 5.4 Hz, 1H), 2.67 – 2.55 (m, 1H), 2.49 – 2.39 (m, 1H), 2.06 – 1.97 (m, 1H), 1.32 (s, 12H); ^{13}C NMR (151 MHz, DMSO) δ 172.9, 171.1, 167.9, 148.1, 138.0, 131.2, 127.7, 125.9, 123.3, 84.0, 51.7, 48.6, 31.2, 24.7, 24.6, 22.5; HRMS calc. for $C_{19}H_{24}{}^{11}BN_2O_5$ $[M+H]^+$ 371.1782, found 371.1784.

***tert*-Butyl 2,6-dioxo-3-[1-oxo-4-(4,4,5,5-tetramethyl-1,3,2-dioxaborolan-2-yl)isoindolin-2-yl]piperidine-1-carboxylate (21)**

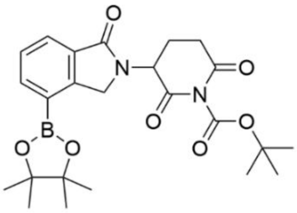

4-(Dimethylamino)pyridine (23.0 mg, 0.19 mmol, 0.15 eq) was added to a solution of 3-[1-oxo-4-(4,4,5,5-tetramethyl-1,3,2-dioxaborolan-2-yl)isoindolin-2-yl]piperidine-2,6-dione (**20**) (470 mg, 1.3 mmol, 1.0 eq) and di-*tert*-butyl-dicarbonate (416 mg, 1.9 mmol, 1.5 eq) in 1,4-dioxane and the solution was stirred at room temperature for three days. The solution was concentrated then re-dissolved in EtOAc (50 mL). The solution was washed with saturated aqueous NH_4Cl (10 mL), saturated aqueous $NaHCO_3$ (10 mL), H_2O (10 mL), and brine (10 mL), dried over Na_2SO_4, filtered, and concentrated to afford the title compound (509 mg, 85%) as an off-white amorphous solid. ^{1}H NMR (600 MHz, DMSO-d_6) δ 7.92 (dd, J = 7.3, 1.2 Hz, 1H), 7.86 (dd, J = 7.6, 1.2 Hz, 1H), 7.56 (t, J = 7.5 Hz, 1H), 5.35 (dd, J = 13.4, 5.2 Hz, 1H), 4.54 (d, J = 17.9 Hz, 1H), 4.43 (d, J = 17.9 Hz, 1H), 3.11 (ddd, J = 17.5, 13.4, 5.5 Hz, 1H), 2.79 (ddd, J = 17.5, 4.5, 2.2 Hz, 1H), 2.57 (qd, J = 13.3, 4.6 Hz, 1H), 2.09 (dtd, J = 12.8, 5.4, 2.2 Hz, 1H), 1.48 (s, 9H), 1.32 (s, 12H); ^{13}C NMR (151 MHz, DMSO-d_6) δ 170.1, 168.8, 168.0, 148.5, 148.1, 138.2, 131.0, 127.7, 126.0, 124.6, 85.9, 84.0, 51.7, 48.8, 30.9, 27.1, 24.7, 24.6, 21.4; HRMS calc. for $C_{24}H_{31}{}^{11}BNaN_2O_7$ $[M+Na]^+$ 493.2117, found 493.2114.

***tert*-Butyl 3-(4-hydroxy-1-oxo-isoindolin-2-yl)-2,6-dioxo-piperidine-1-carboxylate (22)**

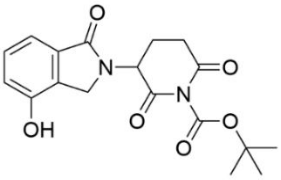

Aqueous H_2O_2 (30% w/w, 0.220 mL, 2.15 mmol, 2.0 eq) was added to a solution of *tert*-butyl 2,6-dioxo-3-[1-oxo-4-(4,4,5,5-tetramethyl-1,3,2-dioxaborolan-2-yl)isoindolin-

2-yl]piperidine-1-carboxylate (**21**) (505 mg, 1.07 mmol, 1.0 eq) in DMF (7.7 mL) and the solution was stirred at room temperature for four hours then the solution was diluted with EtOAc (60 mL). The solution was washed with saturated aqueous $NaHCO_3$ (20 mL), H_2O (2 × 40 mL), and brine (20 mL), then adsorbed onto silica and purified by normal-phase flash chromatography (0–10% MeOH/CH_2Cl_2) to afford the title compound (305 mg, 79%) as a brown amorphous solid. ^{1}H NMR (600 MHz, DMSO-d_6) δ 10.13 (s, 1H), 7.34 (t, *J* = 7.7 Hz, 1H), 7.19 (dd, *J* = 7.4, 0.9 Hz, 1H), 7.03 (dd, *J* = 8.0, 0.9 Hz, 1H), 5.31 (dd, *J* = 13.4, 5.1 Hz, 1H), 4.36 (d, *J* = 17.0 Hz, 1H), 4.20 (d, *J* = 17.0 Hz, 1H), 3.12 (ddd, *J* = 17.6, 13.5, 5.5 Hz, 1H), 2.83 – 2.75 (m, 1H), 2.55 – 2.45 (m, 1H), 2.13 – 2.01 (m, 1H), 1.48 (s, 9H);[†††] ^{13}C NMR (151 MHz, DMSO-d_6) δ 170.1, 168.7, 168.4, 152.6, 148.5, 133.2, 129.6, 128.0, 118.2, 113.8, 85.8, 51.7, 45.3, 30.9, 27.1, 21.5.

***tert*-Butyl 3-[4-[5-[5-(*tert*-butoxycarbonylamino)pentoxy]pentoxy]-1-oxoisoindolin-2-yl]-2,6-dioxo-piperidine-1-carboxylate (73)**

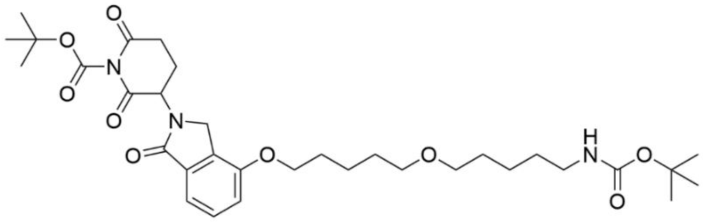

A mixture of *tert*-butyl 3-(4-hydroxy-1-oxo-isoindolin-2-yl)-2,6-dioxo-piperidine-1-carboxylate (**22**) (40 mg, 0.11 mmol, 1.0 eq), Common Intermediate A (**4**) (39 mg, 0.11 mmol, 1.0 eq), and K_2CO_3 (23 mg, 0.17 mmol, 1.5 eq) in DMF (0.56 mL) was stirred at room temperature for 20 h, then stirred at 45 °C for four hours. The solution was diluted with EtOAc (30 mL), washed with saturated aqueous NH_4Cl (10 mL), H_2O (10 mL), and brine (10 mL). The solution was dried over Na_2SO_4, filtered, adsorbed onto silica, and purified by normal-phase flash chromatography (0–8% MeOH/CH_2Cl_2) to afford the title compound (29 mg, 41%) as a colourless oil. ^{1}H NMR (600 MHz, chloroform-*d*) δ 7.50 – 7.44 (m, 1H), 7.40 (t, *J* = 7.8 Hz, 1H), 7.00 (d, *J* = 8.0 Hz, 1H), 5.29 (dd, *J* = 13.5, 5.0 Hz, 1H), 4.42 (d, *J* = 16.4 Hz, 1H), 4.28 (d, *J* = 16.4 Hz, 1H), 4.05 (t, *J* = 6.4 Hz, 2H), 3.45 – 3.36 (m, 6H), 3.09 (t, *J* = 7.1 Hz, 3H), 3.01 – 2.85 (m,

[†††]CH obscured by DMSO-d_6 residual solvent peak

2H), 2.39 (qd, J = 13.3, 4.8 Hz, 1H), 2.23 – 2.15 (m, 1H), 1.84 – 1.78 (m, 2H), 1.64 (p, J = 6.6 Hz, 3H), 1.60 – 1.45 (m, 12H), 1.42 (s, 9H), 1.39 – 1.33 (m, 2H); ^{13}C NMR (151 MHz, chloroform-*d*) δ 169.7, 168.9, 167.7, 156.1, 154.2, 148.1, 132.9, 130.0, 116.1, 114.1, 87.1, 79.2, 70.9, 70.8, 68.2, 51.8, 45.1, 40.7, 31.9, 30.0, 29.6, 29.5, 29.2, 28.6, 28.1 (d, J = 2.0 Hz), 27.5, 23.6, 22.9, 22.9; HRMS calc. for $C_{33}H_{50}N_3O_9$ $[M+H]^+$ 632.3542, found 632.3537.

4-[[9-Chloro-7-(2-fluoro-6-methoxy-phenyl)-5*H*-pyrimido[5,4-*d*][2]benzazepin-2-yl]amino]-*N*-[5-[5-[2-(2,6-dioxo-3-piperidyl)-1-oxo-isoindolin-4-yl]oxypentoxy] pentyl]-2-methoxy-benzamide (47)

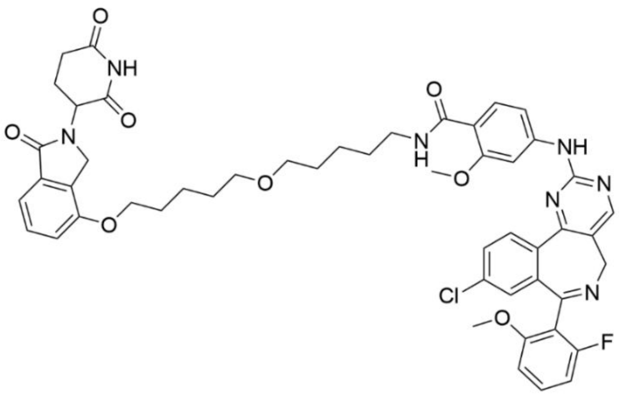

Trifluoroacetic acid (35 µL, 0.460 mmol, 10 eq) was added to a solution of *tert*-butyl 3-[4-[5-[5-(*tert*-butoxycarbonylamino)pentoxy]pentoxy]-1-oxo-isoindolin-2-yl]-2,6-dioxo-piperidine-1-carboxylate (**73**) (29 mg, 0.046 mmol, 1.0 eq) in CH_2Cl_2 (0.46 mL). The solution was stirred at room temperature for 90 min and additional trifluoroacetic acid (35 µL, 0.460 mmol, 10 eq) was added. The solution was stirred for a further two hours, the solution was concentrated, and the residue was redissolved in DMF (2.3 mL). *O*-(benzotriazol-1-yl)-*N*,*N*,*N*',*N*'-tetramethyluronium hexafluorophosphate (17 mg, 0.046 mmol, 1.0 eq) was added and the solution was stirred until everything was dissolved. Alisertib (23 mg, 0.044 mmol, 0.95 eq) and *N*,*N*-diisopropylethylamine (32 µL, 0.180 mmol, 4.0 eq) were added and the solution was stirred at room temperature for 18 h then concentrated on a Biotage V10. The residue was purified by reversed-phase flash chromatography (50–100% MeOH containing 0.1% HCO_2H/H_2O containing 0.1% HCO_2H) to afford the title compound (15 mg, 32%) as an off-white amorphous solid. ^{1}H NMR (600 MHz, DMSO-d_6) δ 10.97 (s, 1H), 10.17 (s, 1H), 8.72 (s, 1H), 8.30 (d, J = 8.5 Hz, 1H), 8.03 (t, J = 5.8 Hz, 1H), 7.96 (s, 1H), 7.85 – 7.78 (m, 2H), 7.49 – 7.39 (m, 3H), 7.29 (d, J = 7.5 Hz, 1H), 7.26 – 7.17 (m, 2H), 7.04 – 6.75 (br m, 2H), 5.11 (dd,

J = 13.3, 5.1 Hz, 1H), 4.89 (br s, 1H), 4.36 (d, J = 17.4 Hz, 1H), 4.22 (d, J = 17.3 Hz, 1H), 4.09 (t, J = 6.4 Hz, 2H), 3.92 (s, 3H), 3.37 (q, 4H, overlapping with H_2O peak), 3.28 (q, J = 6.7 Hz, 2H, overlapping with H_2O peak), 2.91 (ddd, J = 17.3, 13.6, 5.4 Hz, 1H), 2.58 (ddd, J = 17.3, 4.4, 2.3 Hz, 1H), 2.49 – 2.40 (m, 1H), 1.98 (dtd, J = 12.7, 5.3, 2.2 Hz, 1H), 1.78 – 1.71 (m, 2H), 1.59 – 1.50 (m, 6H), 1.49 – 1.42 (m, 2H), 1.38 – 1.32 (m, 2H);[‡‡‡] ^{13}C NMR (151 MHz, DMSO-d_6) δ 172.9, 171.0, 168.1, 164.3, 160.9, 159.4, 157.7, 157.2, 153.8, 144.4, 137.7, 135.2, 134.5, 133.2, 131.4, 131.2, 130.9, 130.4, 129.8, 129.7, 127.4, 123.4, 115.1, 114.9, 114.5, 110.4, 108.0, 101.4, 69.9, 69.8, 67.9, 56.3, 55.7, 51.6, 49.5, 45.0, 38.9, 31.2, 29.1, 29.0, 28.9, 28.4, 23.2, 22.4, 22.3; HRMS calc. for $C_{50}H_{52}{}^{35}ClFN_7O_8$ $[M+H]^+$ 932.3544, found 932.3544.

[‡‡‡]CH and OCH_3 not observed due to peak broadening.

Synthesis of 4-[[9-chloro-7-(2-fluoro-6-methoxy-phenyl)-5*H*-pyrimido[5,4-*d*][2]benzazepin-2-yl]amino]-*N*-[5-[5-[2-[1-(2,6-dioxo-3-piperidyl)triazol-4-yl]phenoxy]pentoxy] pentyl]-2-methoxy-benzamide (48)

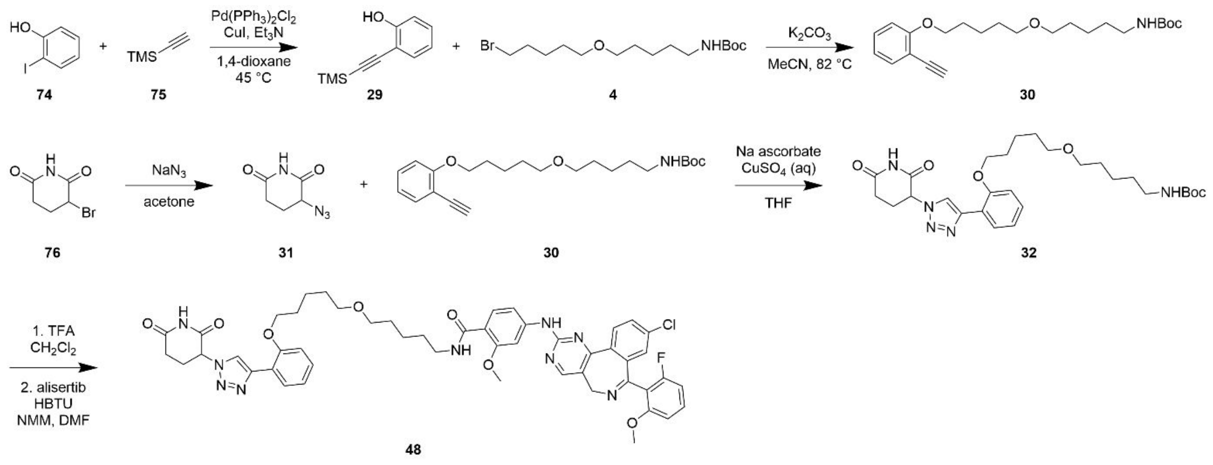

2-(2-Trimethylsilylethynyl)phenol (29)

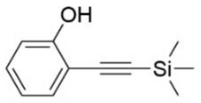

Prepared according to literature procedure.[9] Trimethylsilylacetylene (**75**) (0.33 mL, 2.40 mmol, 1.3 eq), bis(triphenylphosphine)palladium(ii) chloride (13 mg, 0.018 mmol, 0.01 eq), and CuI (7 mg, 0.036 mmol, 0.02 eq) were added to a solution of 2-iodophenol (**74**) (400 mg, 1.80 mmol, 1.0 eq) in 1,4-dioxane (2.0 mL) and triethylamine (1.2 mL) under an atmosphere of N_2. The reaction was stirred at 45 °C for 20 h then cooled to room temperature and filtered through Celite, washing through with Et_2O (50 mL). The solution was washed with 1 M aqueous HCl (10 mL), saturated aqueous $NaHCO_3$ (3 × 10 mL), and brine (10 mL) then adsorbed onto silica and purified by normal-phase flash chromatography (0–4% EtOAc/cyclohexane) to afford the title compound (230 mg, 67%) as a brown oil. ^1H NMR (600 MHz, chloroform-*d*) δ 7.36 (dd, *J* = 7.7, 1.6 Hz, 1H), 7.29 – 7.25 (m, 1H), 6.97 (d, *J* = 8.3 Hz, 1H), 6.88 (td, *J* = 7.5, 1.1 Hz, 1H), 5.86 (d, *J* = 1.9 Hz, 1H), 0.31 (d, *J* = 1.3 Hz, 9H); ^{13}C NMR (151 MHz, chloroform-*d*) δ 157.2, 131.7, 130.8, 120.4, 114.7, 109.6, 102.5, 99.1, 0.1.

***tert*-Butyl *N*-[5-[5-(2-ethynylphenoxy)pentoxy]pentyl]carbamate (30)**

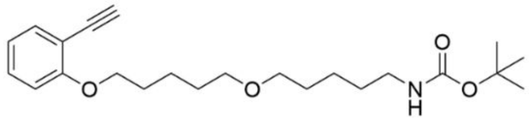

A mixture of 2-(2-trimethylsilylethynyl)phenol (**29**) (86 mg, 0.45 mmol, 1.0 eq), Common Intermediate A (**4**) (175 mg, 0.50 mmol, 1.1 eq), and K_2CO_3 (125 mg, 0.90 mmol, 2.0 eq) in MeCN (1.5 mL) was stirred at 82 °C for 20 h then cooled to room temperature and filtered. The solution was adsorbed onto silica and purified by normal-phase flash chromatography (0–25% EtOAc/cyclohexane) to afford the title compound (102 mg, 58%) as a yellow oil. This compound was isolated as an impure mixture. ^{1}H NMR (600 MHz, chloroform-*d*) δ 7.44 (dd, *J* = 7.6, 1.8 Hz, 1H), 7.28 (ddd, *J* = 8.8, 7.5, 1.8 Hz, 1H), 6.91 – 6.84 (m, 2H), 4.59 – 4.43 (m, 1H), 4.04 (t, *J* = 6.6 Hz, 2H), 3.41 (dt, *J* = 15.6, 6.5 Hz, 4H), 3.25 (s, 1H), 3.11 (q, *J* = 6.7 Hz, 2H), 1.90 – 1.83 (m, 2H), 1.65 (dt, *J* = 15.0, 6.7 Hz, 2H), 1.57 (dq, *J* = 16.0, 9.1, 7.9 Hz, 4H), 1.52 – 1.46 (m, 2H), 1.43 (s, 9H), 1.40 – 1.33 (m, 2H); ^{13}C NMR (151 MHz, chloroform-*d)* δ 160.3, 156.1, 134.2, 130.3, 120.4, 112.1, 111.8, 81.1, 80.3, 79.2, 70.90, 70.85, 68.7, 40.7, 30.0, 29.6, 29.6, 29.0, 28.6, 23.7, 22.8; HRMS calc. for $C_{23}H_{35}NaNO_4$ $[M+Na]^+$ 412.2464, found 412.2466.

3-Azidopiperidine-2,6-dione (31)

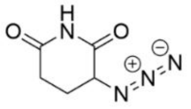

Prepared according to literature procedure.[10] NaN_3 (280 mg, 4.3 mmol, 3.0 eq) was added to a solution of 3-bromopiperidine-2,6-dione (**76**) (276 mg, 1.4 mmol, 1.0 eq) in acetone (0.72 mL) and the solution was stirred at room temperature for three days. The mixture was filtered through Celite, adsorbed onto silica, and purified by normal-phase flash chromatography (0–80% EtOAc/cyclohexane) to afford the title compound (183 mg, 83%) as a white solid. ^{1}H NMR (600 MHz, DMSO-d_6) δ 11.05 (s, 1H), 4.56 (dd, *J* = 11.9, 5.3 Hz, 1H), 2.59 (ddd, *J* = 17.6, 12.2, 5.4 Hz, 1H), 2.55 – 2.39 (m, 1H),

2.04 (dtd, J = 12.9, 5.4, 3.7 Hz, 1H), 1.85 (qd, J = 12.4, 5.0 Hz, 1H);[§§§] ^{13}C NMR (151 MHz, DMSO-d_6) δ 172.4, 171.4, 58.2, 30.0, 23.5.

***tert*-Butyl *N*-[5-[5-[2-[1-(2,6-dioxo-3-piperidyl)triazol-4-yl]phenoxy]pentoxy] pentyl]carbamate (32)**

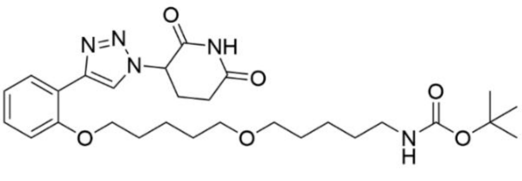

(+)-Sodium L-ascorbate (55 mg, 0.260 mmol, 1.0 eq) and aqueous $CuSO_4$ (0.05 M, 0.51 mL, 0.026 mmol, 0.1 eq) were added to a solution of 3-azidopiperidine-2,6-dione (**31**) (40 mg, 0.260 mmol, 1.0 eq) and *tert*-butyl *N*-[5-[5-(2-ethynylphenoxy)pentoxy]pentyl]carbamate (**30**) (100 mg, 0.260 mmol, 1.0 eq) and THF (5.1 mL) and the solution was stirred at room temperature for 27 h. The solution was diluted with EtOAc (30 mL) and washed with brine (10 mL). The solution was adsorbed onto silica and purified by normal-phase flash chromatography (0–5% MeOH/CH_2Cl_2) to afford the title compound (39 mg, 28%) as a yellow gum-like solid. ^{1}H NMR (600 MHz, chloroform-*d*) δ 8.66 (s, 1H), 8.36 (d, J = 7.7 Hz, 1H), 8.22 (d, J = 2.8 Hz, 1H), 7.32 (t, J = 8.0 Hz, 1H), 7.15 – 7.05 (m, 1H), 6.98 (d, J = 7.8 Hz, 1H), 5.51 – 5.37 (m, 1H), 4.59 (s, 1H), 4.12 (q, J = 5.1, 3.8 Hz, 2H), 3.49 – 3.38 (m, 4H), 3.20 – 3.07 (m, 2H), 3.07 – 2.96 (m, 1H), 2.95 – 2.79 (m, 2H), 2.72 – 2.55 (m, 1H), 1.99 – 1.85 (m, 2H), 1.76 – 1.54 (m, 6H), 1.53 – 1.41 (m, 11H), 1.41 – 1.31 (m, 2H);[****] ^{13}C NMR (151 MHz, chloroform-*d)* δ 170.7, 167.3, 156.0, 155.3, 142.9, 129.3, 127.9, 123.7, 121.1, 119.1, 111.7, 79.3, 70.8, 70.7, 68.3, 59.2, 40.6, 30.4, 30.0, 29.7, 29.4, 29.4, 28.5, 24.9, 23.5, 23.4; HRMS calc. for $C_{28}H_{42}N_5O_6$ [M+H]$^+$ 544.3135, found 544.3134.

[§§§]CH obscured by DMSO-d_6 residual solvent peak

[****]CH obscured by chloroform-*d* residual water peak

4-[[9-Chloro-7-(2-fluoro-6-methoxy-phenyl)-5*H*-pyrimido[5,4-*d*][2]benzazepin-2-yl]amino]-*N*-[5-[5-[2-[1-(2,6-dioxo-3-piperidyl)triazol-4-yl]phenoxy]pentoxy] pentyl]-2-methoxy-benzamide (48)

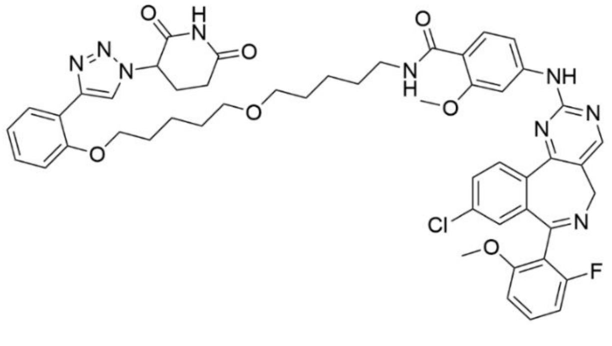

Trifluoroacetic acid (0.11 mL, 1.4 mmol, 20 eq) was added to a solution of *tert*-butyl *N*-[5-[5-[2-[1-(2,6-dioxo-3-piperidyl)triazol-4-yl]phenoxy]pentoxy]pentyl]carbamate (**32**) (38 mg, 0.07 mmol, 1.0 eq) in CH_2Cl_2 (1.4 mL) and the solution was stirred at room temperature for 75 minutes then concentrated. The residue was dissolved in DMF (2.8 mL), *O*-(benzotriazol-1-yl)-*N*,*N*,*N*',*N*'-tetramethyluronium hexafluorophosphate (27 mg, 0.07 mmol, 1.0 eq) was added and the solution was stirred until everything was dissolved. Alisertib (33 mg, 0.06 mmol, 0.9 eq) and *N*-methylmorpholine (31 µL, 0.28 mmol, 4.0 eq) were added and the solution was stirred at room temperature for 22 h. The solution was diluted with EtOAc (30 mL). The solution was washed with saturated aqueous NH_4Cl (10 mL), H_2O (20 mL), and brine (10 mL), then adsorbed onto silica and purified by normal-phase flash chromatography (0–9% MeOH/CH_2Cl_2) then reversed-phase flash chromatography (60–100% MeOH containing 0.1% HCO_2H/H_2O containing 0.1% HCO_2H) to afford the title compound (26 mg, 38%) as a yellow amorphous solid. ^1H NMR (600 MHz, DMSO-*d*$_6$) δ 11.23 (s, 1H), 10.16 (s, 1H), 8.71 (s, 1H), 8.34 (s, 1H), 8.33 – 8.27 (m, 1H), 8.13 (dd, *J* = 7.7, 1.8 Hz, 1H), 8.02 (t, *J* = 5.7 Hz, 1H), 7.95 (br s, 1H), 7.84 – 7.77 (m, 2H), 7.45 – 7.38 (m, 2H), 7.30 (ddd, *J* = 8.7, 7.3, 1.8 Hz, 1H), 7.21 (br s, 1H), 7.10 (dd, *J* = 8.5, 1.0 Hz, 1H), 7.03 (td, *J* = 7.5, 1.0 Hz, 1H), 6.96 – 6.70 (br m, 2H), 5.89 (dd, *J* = 12.6, 5.1 Hz, 1H), 4.14 – 4.04 (m, 2H), 3.91 (s, 3H), 3.35 (q, *J* = 6.4 Hz, 4H), 3.26 (q, *J* = 6.7 Hz, 2H), 2.93 – 2.84 (m, 1H), 2.79 – 2.66 (m, 2H), 2.35 (dtd, *J* = 11.7, 4.8, 4.4, 2.2 Hz, 1H), 1.89 – 1.80 (m, 2H), 1.59 – 1.41 (m, 8H), 1.37 – 1.29 (m, 2H);[††††] ^{13}C NMR (151 MHz, DMSO-*d*$_6$) δ

[††††] CH_2 and OCH_3 not observed due to peak broadening.

172.4, 169.5, 164.3, 160.9, 159.5 (d, J = 248.4 Hz), 159.4, 157.7, 157.2, 154.7, 144.4, 141.8, 138.3, 135.2, 134.7, 131.4, 131.2, 130.9, 130.1, 130.0, 127.2, 126.7, 124.0, 123.4, 120.5, 119.0, 115.1, 112.3, 110.4, 108.0, 101.4, 69.9, 68.0, 59.0, 56.3, 55.7, 49.4, 38.9, 30.8, 29.1, 29.0, 28.9, 28.4, 24.2, 23.2, 22.4; HRMS calc. for $C_{50}H_{52}{}^{35}ClFN_9O_7$ $[M+H]^+$ 944.3662, found, 944.3682.

Synthesis of 4-[[9-chloro-7-(2-fluoro-6-methoxy-phenyl)-5*H*-pyrimido[5,4-*d*][2]benzazepin-2-yl]amino]-*N*-[5-[5-[4-(2,6-dioxo-3-piperidyl)phenoxy]pentoxy]pentyl]-2-methoxy-benzamide (49)

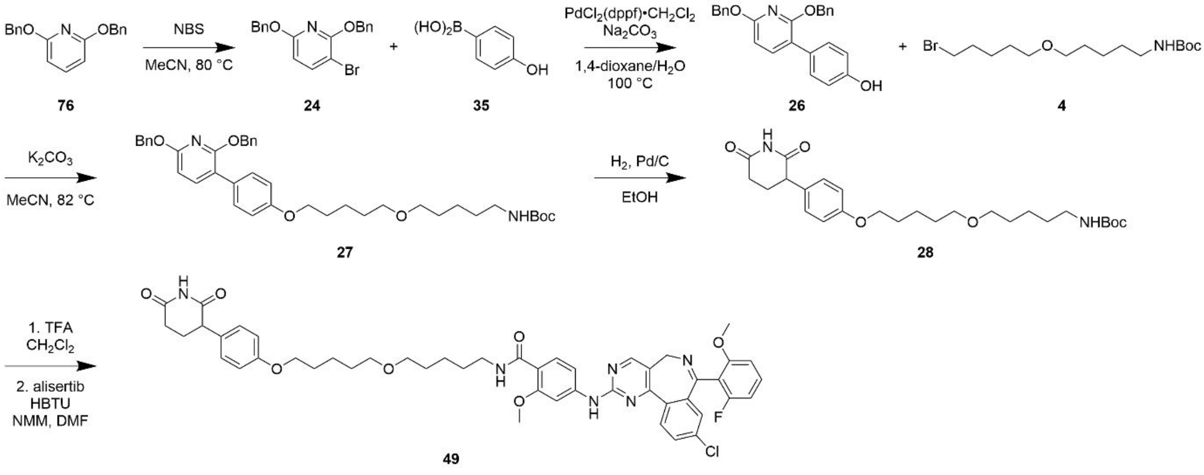

2,6-Dibenzyloxy-3-bromo-pyridine (24)

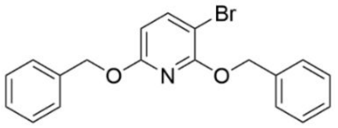

Prepared according to literature procedure.[11] *N*-bromosuccinimide (605 mg, 3.40 mmol, 0.99 eq) was added to a solution of 2,6-bis(benzyloxy)pyridine (**76**) (1.0 g, 3.43 mmol, 1.0 eq) in MeCN (5.5 mL) and the solution was stirred at 80 °C for 75 min. The solution was cooled to room temperature and diluted with EtOAc (60 mL). The solution was washed with H_2O (30 mL) and brine (15 mL), dried over Na_2SO_4, filtered, adsorbed onto silica, and purified by normal-phase flash chromatography (0–2% EtOAc/cyclohexane) to afford the title compound (1.16 g, 92%) as a white solid. ^{1}H NMR (600 MHz, chloroform-*d*) δ 7.67 (dd, *J* = 8.3, 1.2 Hz, 1H), 7.44 (d, *J* = 7.6 Hz, 2H), 7.41 – 7.33 (m, 6H), 7.33 – 7.28 (m, 2H), 6.31 (dd, *J* = 8.2, 1.2 Hz, 1H), 5.42 (s, 2H), 5.29 (s, 2H); ^{13}C NMR (151 MHz, chloroform-*d*) δ 161.4, 157.8, 144.1, 137.33, 137.26, 128.7, 128.6, 128.1, 127.9, 127.9, 127.4, 103.9, 96.1, 68.5, 68.3; HRMS calc. for $C_{19}H_{17}{}^{79}BrNO_2$ [M+H]+ 370.0443, found 370.0439.

4-(2,6-Dibenzyloxy-3-pyridyl)phenol (26)

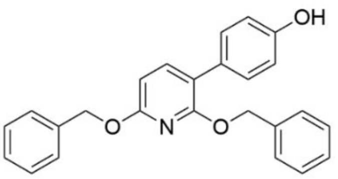

Prepared according to literature procedure.[12] [1,1'-Bis(diphenylphosphino)ferrocene] dichloropalladium(II), complex with CH_2Cl_2 (101 mg, 0.12 mmol, 0.1 eq), 4-hydroxyphenylboronic acid (**35**) (212 mg, 1.5 mmol, 1.3 eq), Na_2CO_3 (250 mg, 2.4 mmol, 2.0 eq), and 1,4-dioxane (4.1 mL) were added to a mixture of 2,6-dibenzyloxy-3-bromo-pyridine (**24**) (520 mg, 1.2 mmol, 1.0 eq) in H_2O (0.56 mL) under an atmosphere of N_2. The solution was stirred at 100 °C for 19 h then cooled to room temperature and filtered through Celite. The solution was adsorbed onto silica and purified by normal-phase flash chromatography (0–35% EtOAc/cyclohexane) to afford the title compound (352 mg, 78%) as an off-white amorphous solid. ^{1}H NMR (600 MHz, chloroform-*d*) δ 7.56 (dd, *J* = 8.0, 0.7 Hz, 1H), 7.46 – 7.40 (m, 4H), 7.39 – 7.35 (m, 4H), 7.35 – 7.31 (m, 3H), 7.30 – 7.27 (m, 1H), 6.88 – 6.83 (m, 2H), 6.46 (dd, *J* = 8.1, 0.7 Hz, 1H), 5.42 (s, 2H), 5.36 (s, 2H); ^{13}C NMR (151 MHz, chloroform-*d*) δ 161.1, 158.4, 154.5, 141.6, 138.1, 137.8, 130.4, 129.6, 128.6, 128.5, 127.9, 127.6, 127.4, 115.8, 115.2, 102.5, 68.0, 67.7; HRMS calc. for $C_{25}H_{21}NaNO_3$ $[M+Na]^+$ 406.1419, found 406.1426.

***tert*-Butyl *N*-[5-[5-[4-(2,6-dibenzyloxy-3-pyridyl)phenoxy]pentoxy] pentyl] carbamate (27)**

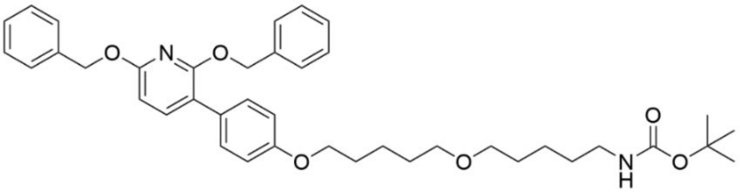

A mixture of 4-(2,6-dibenzyloxy-3-pyridyl)phenol (**26**) (56 mg, 0.15 mmol, 1.0 eq), Common Intermediate A (**4**) (57 mg, 0.16 mmol, 1.1 eq), and K_2CO_3 (40 mg, 0.29 mmol, 2.0 eq) in MeCN (0.73 mL) was stirred at 82 °C for 19 h then cooled to room

temperature and filtered. The solution was adsorbed onto silica and purified by normal-phase flash chromatography (0–15% EtOAc/$CHCl_3$) to afford the title compound (70 mg, 73%) as a yellow oil. ^{1}H NMR (600 MHz, chloroform-*d*) δ 7.57 (d, *J* = 8.0 Hz, 1H), 7.49 – 7.46 (m, 2H), 7.44 – 7.41 (m, 2H), 7.39 – 7.34 (m, 3H), 7.34 – 7.30 (m, 3H), 7.30 – 7.26 (m, 1H), 6.93 – 6.89 (m, 2H), 6.45 (d, *J* = 8.0 Hz, 1H), 5.42 (s, 2H), 5.35 (s, 2H), 4.52 (s, 1H), 3.99 (t, *J* = 6.5 Hz, 2H), 3.47 – 3.36 (m, 4H), 3.11 (t, *J* = 7.1 Hz, 2H), 1.82 (p, *J* = 6.8 Hz, 2H), 1.69 – 1.56 (m, 4H), 1.56 – 1.46 (m, 4H), 1.44 (s, 9H), 1.41 – 1.34 (m, 2H);[‡‡‡‡] ^{13}C NMR (151 MHz, chloroform-*d*) δ 161.1, 158.4, 158.2, 156.0, 141.6, 138.1, 137.8, 130.2, 129.2, 128.6, 128.5, 127.9, 127.5, 127.4, 115.9, 114.3, 102.5, 79.2, 70.92, 70.89, 68.0, 67.7, 40.7, 30.1, 29.7, 29.6, 29.3, 28.6, 23.7, 23.0.

***tert*-Butyl *N*-[5-[5-[4-(2,6-dioxo-3-piperidyl)phenoxy]pentoxy]pentyl]carbamate (28)**

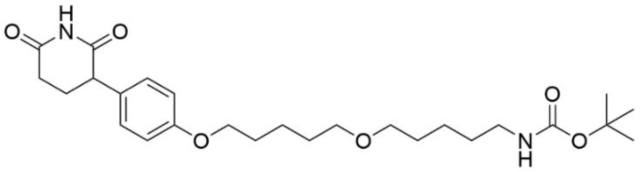

Pd (10% w/w on C, 11 mg, 0.01 mmol, 0.1 eq) was added to a solution of *tert*-butyl *N*-[5-[5-[4-(2,6-dibenzyloxy-3-pyridyl)phenoxy]pentoxy]pentyl]carbamate (**27**) (66 mg, 0.10 mmol, 1.0 eq) in EtOH (2.0 mL) under an atmosphere of N_2. The reaction vessel was purged with H_2, and the reaction was stirred under an atmosphere of H_2 for five days. The reaction vessel was purged with N_2, Pd (10% w/w on C, 20 mg, 0.018 mmol, 0.18 eq) was added, the reaction vessel was purged with H_2, and the reaction was stirred under an atmosphere of H_2 for seven days. The reaction vessel was purged with N_2, the mixture was filtered through Celite, and the solution was concentrated to afford the title compound as a yellow oil. This was immediately used in the subsequent step.

[‡‡‡‡]CH obscured by chloroform-*d* residual water peak

4-[[9-Chloro-7-(2-fluoro-6-methoxy-phenyl)-5*H*-pyrimido[5,4-*d*][2]benzazepin-2-yl]amino]-*N*-[5-[5-[4-(2,6-dioxo-3-piperidyl)phenoxy]pentoxy]pentyl]-2-methoxy-benzamide (49)

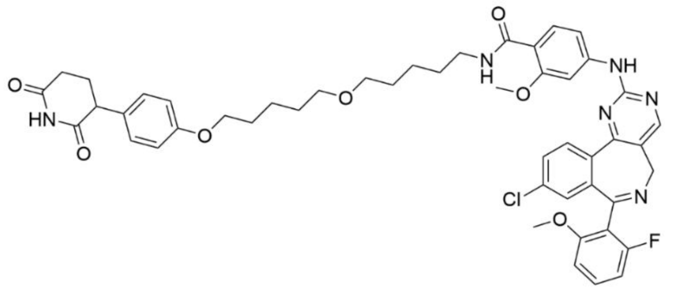

Trifluoroacetic acid (77 µL, 1.01 mmol, 10 eq) was added to a solution of *tert*-butyl *N*-[5-[5-[4-(2,6-dioxo-3-piperidyl)phenoxy]pentoxy]pentyl]carbamate (**28**) (48 mg, 0.100 mmol, 1.0 eq) in CH_2Cl_2 (2.0 mL) and the reaction was stirred at room temperature for 90 min. The solution was concentrated, and the residue was dissolved in DMF (4.0 mL). *O*-(Benzotriazol-1-yl)-*N*,*N*,*N*',*N*'-tetramethyluronium hexafluorophosphate (38 mg, 0.100 mmol, 1.0 eq) was added and the solution was stirred until everything was dissolved. Alisertib (47 mg, 0.09 mmol, 0.9 eq) and *N*-methylmorpholine (44 µL, 0.400 mmol, 4.0 eq) were added and the solution was stirred at room temperature for 21 h then the solvent was removed on a Biotage V10. The residue was purified by reversed-phase flash chromatography (50–100% MeOH containing 0.1% HCO_2H/H_2O containing 0.1% HCO_2H) then normal-phase flash chromatography (0–6% MeOH/CH_2Cl_2) to afford the title compound (24 mg, 27%) as an off-white amorphous solid. ^{1}H NMR (600 MHz, DMSO-*d*$_6$) δ 10.77 (s, 1H), 10.16 (s, 1H), 8.71 (s, 1H), 8.30 (d, *J* = 8.5 Hz, 1H), 8.02 (t, *J* = 5.7 Hz, 1H), 7.96 (br s, 1H), 7.84 – 7.78 (m, 2H), 7.44 – 7.38 (m, 2H), 7.22 (br s, 1H), 7.09 (d, *J* = 8.7 Hz, 2H), 7.03 – 6.72 (m, 4H), 3.94 – 3.89 (m, 5H), 3.75 (dd, *J* = 11.4, 4.9 Hz, 1H), 3.36 (td, *J* = 6.5, 2.0 Hz, 4H, overlapping with H_2O peak), 3.27 (q, *J* = 6.7 Hz, 2H, overlapping with H_2O peak), 2.63 (ddd, *J* = 17.1, 11.7, 5.3 Hz, 1H), 2.46 (dt, *J* = 17.2, 4.3 Hz, 1H), 2.18 – 2.08 (m, 1H), 2.02 – 1.94 (m, 1H), 1.69 (p, *J* = 6.7 Hz, 2H), 1.58 – 1.48 (m, 6H), 1.43 (tt, *J* = 9.6, 5.9 Hz, 2H), 1.39 – 1.31 (m, 2H);[§§§§] ^{13}C NMR (151 MHz, DMSO-*d*$_6$) δ 174.5, 173.4, 164.3, 160.9, 159.5 (d, *J* = 244.6 Hz), 159.4, 157.9 – 157.6 (m), 157.6,

[§§§§] CH_2 and OCH_3 peaks not observed due to peak broadening.

157.2, 144.4, 137.6, 135.2, 134.6, 131.4, 131.3 – 131.1 (m), 130.9, 130.2, 129.5, 127.3, 123.4, 115.1, 114.2, 110.4, 108.3 – 107.7 (m, 2C), 101.5, 69.89, 69.86, 67.3, 56.3, 55.7, 49.5, 46.5, 38.9, 31.3, 29.1, 29.0, 28.5, 26.0, 23.3, 22.4; HRMS calc. for $C_{48}H_{51}{}^{35}ClFN_6O_7$ $[M+H]^+$ 877.3486, found 877.3486.

Synthesis of 4-[[9-chloro-7-(2-fluoro-6-methoxy-phenyl)-5*H*-pyrimido[5,4-*d*][2]benzazepin-2-yl]amino]-*N*-[5-[4-[2-[4-(2,4-dioxohexahydropyrimidin-1-yl)phenoxy]ethyl] piperazin-1-yl]pentyl]-2-methoxy-benzamide (50)

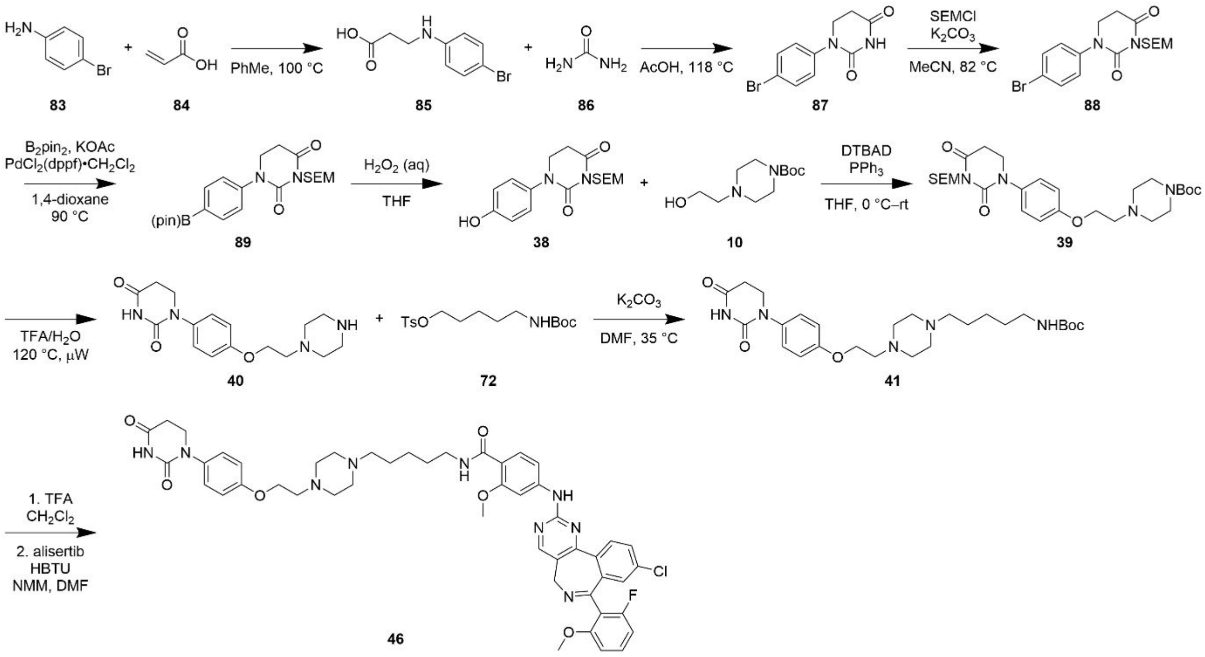

1-(4-Bromophenyl)hexahydropyrimidine-2,4-dione (87)

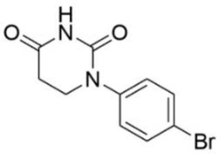

Prepared according to literature procedure.[13] Acrylic acid (**84**) (1.60 mL, 23.3 mmol, 4.0 eq) was added to a solution of 4-bromoaniline (**83**) (1.00 g, 5.80 mmol, 1.0 eq) in PhMe (18 mL) and the solution was stirred at 100 °C for 22 h. The reaction was cooled to room temperature, and the solvent was removed on a rotary evaporator. The residue was dissolved in AcOH (23 mL), urea (**86**) (2.62 g, 43.6 mmol, 7.5 eq) was added, and the solution was stirred at 118 °C for 23 h. The reaction was cooled to room temperature, and the majority of the solvent was removed on a rotary evaporator. H_2O (30 mL) was added, and the formed precipitate was isolated by filtration. The solid was washed with H_2O (20 mL) then dried under high-vacuum at 60 °C to afford the title compound (1.15 g, 74%) as an off-white amorphous solid. ^1H NMR (500 MHz, DMSO-d_6) δ 10.42 (s, 1H), 7.61 – 7.55 (m, 2H), 7.32 – 7.28 (m, 2H), 3.78 (t, J = 6.6

Hz, 2H), 2.70 (t, J = 6.6 Hz, 2H); ^{13}C NMR (126 MHz, DMSO-d_6) δ 170.5, 152.0, 141.3, 131.4, 127.3, 118.1, 44.3, 31.0; HRMS calc. for $C_{10}H_{10}{}^{79}BrN_2O_2$ $[M+H]^+$ 268.9926, found 268.9926.

1-(4-Bromophenyl)-3-(2-trimethylsilylethoxymethyl)hexahydropyrimidine-2,4-dione (88)

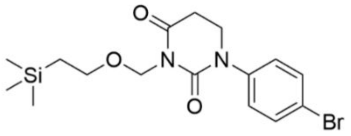

N,*N*-diisopropylethylamine (0.97 mL, 5.6 mmol, 3.0 eq) and 2-(trimethylsilyl)ethoxymethylchloride (0.49 mL, 2.8 mmol, 1.5 eq) were assed to a solution of 1-(4-bromophenyl)hexahydropyrimidine-2,4-dione (**87**) (500 mg, 1.9 mmol, 1.0 eq) in MeCN (4.6 mL) under an atmosphere of N_2. The solution was stirred at 82 °C for 19 h then cooled to room temperature and diluted with EtOAc (50 mL). The mixture was washed with saturated aqueous NH_4Cl (15 mL) and brine (10 mL) then adsorbed onto silica and purified by normal-phase flash chromatography (0–50% EtOAc/cyclohexane) to afford the title compound (720 mg, 97%) as an orange oil. ^{1}H NMR (600 MHz, chloroform-d) δ 7.55 – 7.49 (m, 2H), 7.21 – 7.15 (m, 2H), 5.28 (d, J = 0.9 Hz, 2H), 3.81 (t, J = 6.8 Hz, 2H), 3.70 – 3.62 (m, 2H), 2.91 (t, J = 6.7 Hz, 2H), 0.98 – 0.94 (m, 2H), 0.00 (s, 9H); ^{13}C NMR (151 MHz, chloroform-d) δ 169.0, 152.5, 140.9, 132.3, 126.7, 120.2, 69.8, 67.2, 43.9, 32.1, 18.1, –1.4; HRMS calc. for $C_{16}H_{23}{}^{79}BrNaN_2O_3Si$ $[M+Na]^+$ 421.0559, found 421.0555.

1-(4-Hydroxyphenyl)-3-(2-trimethylsilylethoxymethyl)hexahydropyrimidine-2,4-dione (38)

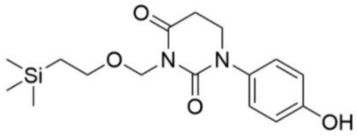

[1,1'-Bis(diphenylphosphino)ferrocene]dichloropalladium(II), complex with dichloromethane (150 mg, 0.18 mmol, 0.10 eq) was added to a mixture of 1-(4-bromophenyl)-3-(2-trimethylsilylethoxymethyl)hexahydropyrimidine-2,4-dione (**88**) (966 mg, 76% purity, 1.8 mmol, 1.0 eq), bis(pinacolato)diboron (934 mg, 3.7 mmol, 2.0 eq), and KOAc (365 mg, 3.7 mmol, 2.0 eq) in 1,4-dioxane (5.6 mL) under an atmosphere of N_2. The mixture was stirred at 90 °C for three hours then cooled to room temperature and diluted with EtOAc (50 mL). The solution was washed with H_2O (30 mL) and brine (20 mL), dried over Na_2SO_4, filtered, and concentrated. The residue was dissolved in THF (9.2 mL) and H_2O_2 (30% w/w aqueous solution, 0.38 mL, 3.7 mmol, 2.0 eq) was added. The solution was stirred at room temperature for 40 minutes after which time a further portion of H_2O_2 (30% w/w aqueous solution, 1.9 mL, 18.4 mmol, 10 eq) was added and the solution was stirred at room temperature for 80 minutes. The solution was diluted with EtOAc (60 mL), and the mixture was washed with saturated aqueous $NaHCO_3$ (20 mL), H_2O (20 mL), and brine (15 mL). The solution was adsorbed onto silica and purified by normal-phase flash chromatography (0–60% EtOAc/cyclohexane) to afford the title compound (88% purity, 703 mg, 100%) as a colourless oil, isolated with residual pinacol. ^{1}H NMR (600 MHz, chloroform-*d*) δ 7.12 – 7.04 (m, 2H), 6.78 – 6.69 (m, 2H), 6.31 (s, 1H), 5.31 (s, 2H), 3.77 (t, J = 6.7 Hz, 2H), 3.73 – 3.66 (m, 2H), 2.91 (t, J = 6.7 Hz, 2H), 1.03 – 0.96 (m, 2H), 0.03 (s, 9H); ^{13}C NMR (151 MHz, chloroform-*d*) δ 169.37, 155.22, 153.42, 134.15, 126.89, 116.32, 69.77, 67.17, 44.56, 32.11, 18.15, –1.39; HRMS calc. for $C_{16}H_{24}NaN_2O_4Si$ $[M+Na]^+$ 359.1404, found 359.1405.

tert-Butyl 4-[2-[4-[2,4-dioxo-3-(2-trimethylsilylethoxymethyl)hexahydro pyrimidin-1-yl]phenoxy]ethyl]piperazine-1-carboxylate (39)

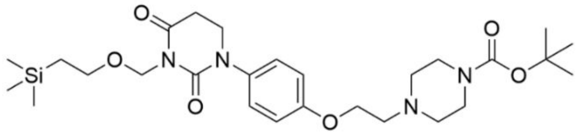

Di-*tert*-butylazodicarboxylate (635 mg, 2.7 mmol, 1.3 eq) was added to a solution of 1-(4-hydroxyphenyl)-3-(2-trimethylsilylethoxymethyl)hexahydropyrimidine-2,4-dione (**38**) (700 mg, 2.1 mmol, 1.0 eq), *tert*-butyl 4-(2-hydroxyethyl)piperazine-1-carboxylate (**10**) (479 mg, 2.1 mmol, 1.0 eq), and triphenylphosphine (709 mg, 2.7 mmol, 1.3 eq) in THF (10.4 mL) under at atmosphere of N_2 at 0 °C. The solution was stirred at room temperature for 24 h then adsorbed onto silica and purified by normal-phase flash chromatography (0–60% MeOH/CH_2Cl_2) to afford the title compound (424 mg, 37%) as a brown oil. This product was isolated as an impure mixture with di-*tert*-butyl hydrazine-1,2-dicarboxylate. ^{1}H NMR (600 MHz, chloroform-*d*) δ 7.22 – 7.16 (m, 2H), 6.97 – 6.89 (m, 2H), 5.30 – 5.24 (m, 2H), 4.10 (t, *J* = 5.7 Hz, 2H), 3.81 – 3.74 (m, 2H), 3.69 – 3.64 (m, 2H), 3.45 (t, J = 5.1 Hz, 4H), 2.93 – 2.86 (m, 2H), 2.85 – 2.78 (m, 2H), 2.51 (t, *J* = 5.0 Hz, 4H), 1.23 (s, 9H), 1.00 – 0.92 (m, 2H), -0.00 (s, 9H); ^{13}C NMR (151 MHz, chloroform-*d*) δ 169.5, 157.5, 154.9, 153.1, 135.2, 126.8, 115.3, 75.2, 69.9, 67.2, 66.3, 57.3, 53.5, 44.5, 43.1, 32.3, 25.0, 18.3, –1.3; HRMS calc. for $C_{27}H_{45}N_4O_6Si$ $[M+H]^+$ 549.3109, found 549.3111.

1-[4-(2-Piperazin-1-ylethoxy)phenyl]hexahydropyrimidine-2,4-dione (40)

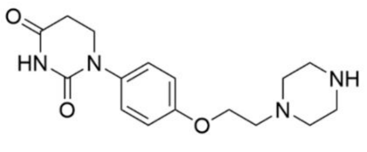

tert-Butyl 4-[2-[4-[2,4-dioxo-3-(2-trimethylsilylethoxymethyl)hexahydropyrimidin-1-yl]phenoxy]ethyl]piperazine-1-carboxylate (**39**) (362 mg, 0.66 mmol, 1.0 eq) was dissolved in a mixture of trifluoroacetic acid (4.4 mL) and H_2O (2.2 mL) and the solution was stirred under microwave irradiation at 120 °C for 90 min. The reaction mixture was diluted with PhMe (20 mL) and the solvent removed on a rotary evaporator. The residue was purified by 5 g SCX-2 cartridge, collecting the fraction that eluted with 7

M NH_3 in MeOH, to afford the title compound (108 mg, 51%) as a brown amorphous solid. This product was isolated with impurities. ^{1}H NMR (600 MHz, DMSO-d_6) δ 10.29 (s, 1H), 7.23 – 7.19 (m, 2H), 6.96 – 6.92 (m, 2H), 4.06 (t, *J* = 5.8 Hz, 2H), 3.71 (t, *J* = 6.7 Hz, 2H), 2.71 (t, *J* = 5.0 Hz, 4H), 2.69 (t, *J* = 6.8 Hz, 2H), 2.65 (t, *J* = 5.9 Hz, 2H), 2.47 – 2.34 (m, 5H); ^{13}C NMR (151 MHz, DMSO-d_6) δ 170.7, 156.5, 152.3, 134.9, 126.9, 114.5, 65.6, 57.3, 54.2, 45.4, 44.9, 31.1; HRMS calc. for $C_{16}H_{23}N_4O_3$ 319.1770, found 319.1761.

***tert*-Butyl *N*-[5-[4-[2-[4-(2,4-dioxohexahydropyrimidin-1-yl)phenoxy]ethyl] piperazin-1-yl]pentyl]carbamate (41)**

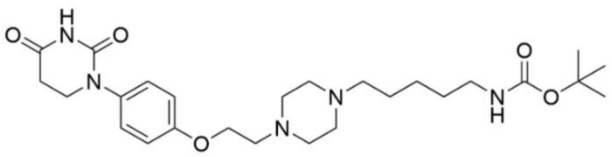

A mixture of 1-[4-(2-piperazin-1-ylethoxy)phenyl]hexahydropyrimidine-2,4-dione (103 mg, 0.32 mmol, 1.0 eq) **(40),** 5-(*tert*-butoxycarbonylamino)pentyl 4-methylbenzenesulfonate (**72**) (116 mg, 0.32 mmol, 1.0 eq), and K_2CO_3 (134 mg, 0.97 mmol, 3.0 eq) in DMF (1.6 mL) was stirred at 35 °C for 19 h then filtered and the DMF was removed under reduced pressure. The residue was dissolved in DMSO and purified by reversed-phase flash chromatography (0–100% MeOH containing 0.1% HCO_2H/H_2O containing 0.1% HCO_2H) to afford the title compound (47 mg, 26%) as a yellow gum. This compound was isolated as the mono-formate salt. ^{1}H NMR (600 MHz, DMSO-d_6) δ 10.30 (s, 1H), 8.24 – 8.04 (m, 1H), 7.26 – 7.19 (m, 2H), 6.99 – 6.91 (m, 2H), 6.76 (t, *J* = 5.8 Hz, 1H), 4.07 (t, J = 5.8 Hz, 2H), 3.71 (t, *J* = 6.7 Hz, 2H), 2.89 (q, *J* = 6.6 Hz, 2H), 2.72 – 2.65 (m, 4H), 2.56 – 2.43 (m, 4H), 2.42 – 2.28 (m, 4H), 2.28 – 2.20 (m, 2H), 1.43 – 1.33 (m, 13H), 1.28 – 1.18 (m, 2H);[*****] ^{13}C NMR (151 MHz, DMSO-d_6) δ 171.1, 163.8, 156.9, 156.0, 152.8, 135.4, 127.3, 115.0, 77.8, 66.2, 58.3, 57.0, 53.5, 53.2, 45.4, 40.2, 31.6, 29.9, 28.8, 26.4, 24.6; HRMS calc. for $C_{26}H_{42}N_5O_7$ $[M+H]^+$ 504.3180, found 504.3182.

[*****]CH obscured by DMSO-d_6 residual solvent peak

4-[[9-Chloro-7-(2-fluoro-6-methoxy-phenyl)-5*H*-pyrimido[5,4-*d*][2]benzazepin-2-yl]amino]-*N*-[5-[4-[2-[4-(2,4-dioxohexahydropyrimidin-1-yl)phenoxy]ethyl] piperazin-1-yl]pentyl]-2-methoxy-benzamide (50)

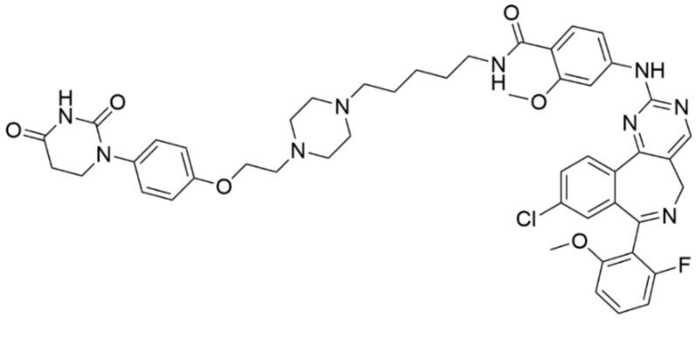

Trifluoroacetic acid (0.14 mL, 1.80 mmol, 20 eq) was added to a solution of *tert*-butyl *N*-[5-[4-[2-[4-(2,4-dioxohexahydropyrimidin-1-yl)phenoxy]ethyl]piperazin-1-yl]pentyl]carbamate (**41**) (45 mg, 0.089 mmol, 1.0 eq) in CH_2Cl_2 (0.89 mL) and the solution was stirred at room temperature for 100 minutes before being concentrated. The residue was dissolved in DMF (2.0 mL), *O*-(Benzotriazol-1-yl)-*N*,*N*,*N'*,*N'*-tetramethyluronium hexafluorophosphate (34 mg, 0.089 mmol, 1.0 eq) was added, and the solution was stirred until everything was dissolved. Alisertib (46 mg, 0.089 mmol, 1.0 eq) and 4-methylmorpholine (39 µL, 0.360 mmol, 4.0 eq) were added and the solution was stirred at room temperature for two hours. The solvent was removed under reduced pressure, and the residue was dissolved in DMSO and purified by reversed-phase flash chromatography (40–100% MeOH containing 0.1% HCO_2H/H_2O containing 0.1% HCO_2H) to afford the title compound (15 mg, 18%) as an off-white amorphous solid. This compound was isolated as the formate salt. ^{1}H NMR (600 MHz, DMSO-*d*$_6$) δ 10.30 (s, 1H), 10.17 (s, 1H), 8.71 (s, 1H), 8.30 (d, *J* = 8.5 Hz, 1H), 8.14 (s, 1H), 8.04 (t, *J* = 5.8 Hz, 1H), 7.96 (br s, 1H), 7.84 – 7.78 (m, 2H), 7.45 – 7.38 (m, 2H), 7.22 (d, *J* = 8.9 Hz, 2H), 6.94 (d, *J* = 9.0 Hz, 2H), 4.09 (t, *J* = 5.6 Hz, 2H), 3.92 (s, 3H), 3.70 (t, *J* = 6.7 Hz, 2H), 3.29 (q, *J* = 6.7 Hz, 2H, overlapping with H_2O peak), 2.78 (t, *J* = 5.5 Hz, 2H), 2.70 – 2.65 (m, 4H), 2.54 (s, 8H, integrates to 5H due to peak broadening), 1.60 – 1.50 (m, 4H), 1.36 – 1.28 (m, 2H);[†††††] ^{13}C NMR (151 MHz, DMSO-*d*$_6$) δ 170.7, 164.4, 164.2, 163.1, 161.0, 159.6 (d, J = 243.0 Hz), 159.4, 157.8, 157.7, 157.2, 156.4, 152.3, 144.4, 135.2, 135.0, 134.6, 131.4, 131.2, 130.9, 130.3, 127.4,

[†††††]CH_2, OCH_3 and 3 x CH missing from spectra due to peak broadening.

126.9, 123.4, 118.0, 115.1, 114.5, 110.4, 108.0, 101.5, 65.5, 56.6, 56.3, 56.0, 55.7, 51.8, 51.4, 49.5, 44.9, 38.7, 31.1, 29.0, 24.5, 23.9; HRMS calc. for $C_{48}H_{52}{}^{35}ClFN_9O_6$ $[M+H]^+$ 904.3713, found 904.3730.

Synthesis of 4-[2-[2-[2-[(3*R*)-1-[4-[[9-chloro-7-(2-fluoro-6-methoxy-phenyl)-5*H*-pyrimido[5,4-*d*][2]benzazepin-2-yl]amino]-2-methoxy-benzoyl]pyrrolidin-3-yl]oxyethoxy] ethoxy]ethoxy]-2-(2,6-dioxo-3-piperidyl)isoindoline-1,3-dione (51)

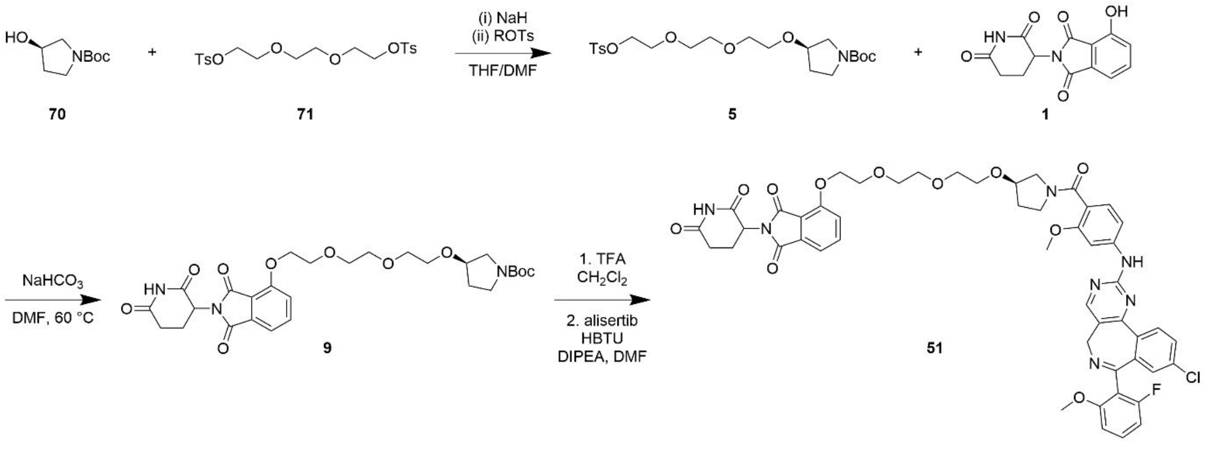

***tert*-Butyl (3*R*)-3-hydroxypyrrolidine-1-carboxylate (70)**

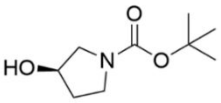

Triethylamine (1.20 mL, 8.3 mmol, 2.0 eq) and di-*tert*-butyl-dicarbonate (1.09 g, 5.0 mmol, 1.2 eq) were added to a solution of (*R*)-3-hydroxypyrrolidine hydrochloride (512 mg, 4.1 mmol, 1.0 eq) in CH_2Cl_2 (4.1 mL) and MeOH (1.0 mL) under at atmosphere of N_2 at 0 °C. The solution was stirred at room temperature for 21 h then the solvent was removed. The residue was suspended in H_2O (10 mL), and the solution was extracted with EtOAc (3 × 20 mL). The combined organic layers were washed with saturated aqueous NH_4Cl (10 mL), saturated aqueous $NaHCO_3$ (10 mL), and brine (10 mL), then dried over Na_2SO_4, filtered, and concentrated to afford the title compound (760 mg, 98%) as a pale-yellow oil. ^{1}H NMR (500 MHz, chloroform-*d*) δ 4.51 – 4.38 (m, 1H), 3.54 – 3.26 (m, 4H), 2.12 – 2.02 (m, 1H), 2.01 – 1.85 (m, 2H), 1.45 (s, 9H); ^{13}C NMR (126 MHz, chloroform-*d*) δ 154.9, 79.5, 72.1 – 69.8 (m), 54.8 – 53.9 (m), 44.5 – 42.9 (m), 34.7 – 33.6 (m), 28.6 (doubling up of peaks in ^{13}C spectrum was observed due to the presence of rotamers, which was assigned via comparison to **51**).

2-[2-[2-(*p*-Tolylsulfonyloxy)ethoxy]ethoxy]ethyl 4-methylbenzenesulfonate (71)

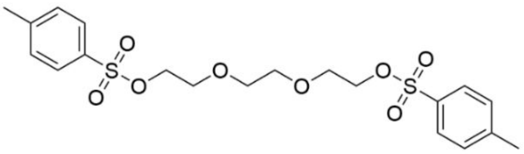

Prepared according to literature procedure.[14] Freshly powdered KOH (1.94 g, 34.6 mmol, 8.0 eq) was added to a solution of triethylene glycol (0.580 mL, 4.30 mmol, 1.0 eq) and *p*-toluenesulfony chloride (1.73 g, 9.1 mmol, 2.1 eq) in CH_2Cl_2 (7.6 mL) under an atmosphere of N_2 at 0 °C. The solution was stirred at room temperature for 3 days and then ice-water (15 mL) was added. The solution was extracted with CH_2Cl_2 (2 × 15 mL), and the combined organic layers were washed with H_2O (10 mL), passed through a phase separator, and concentrated to afford the title compound (1.46 g, 74%) as a yellow amorphous solid. ^{1}H NMR (600 MHz, chloroform-*d*) δ 7.85 – 7.74 (m, 4H), 7.34 (d, *J* = 8.1 Hz, 4H), 4.15 – 4.12 (m, 4H), 3.68 – 3.64 (m, 4H), 3.53 (s, 4H), 2.44 (s, 6H); ^{13}C NMR (151 MHz, chloroform-*d*) δ 145.0, 133.1, 130.0, 128.1, 70.8, 69.3, 68.9, 21.8; HRMS calc. for $C_{20}H_{27}O_8S_2$ [M+H]$^+$ 459.1147, found 459.1147.

***tert*-Butyl (3*R*)-3-[2-[2-[2-(*p*-tolylsulfonyloxy)ethoxy]ethoxy]ethoxy]pyrrolidine-1-carboxylate (5)**

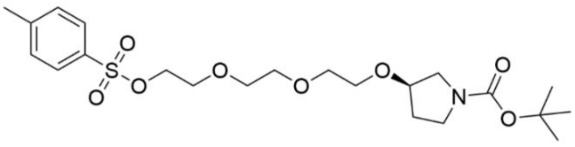

NaH (60% w/w in mineral oil, 56 mg, 1.4 mmol, 1.3 eq) was added to a solution of *tert*-butyl (3*R*)-3-hydroxypyrrolidine-1-carboxylate (**70**) (200 mg, 1.1 mmol, 1.0 eq) in THF (2.1 mL) under an atmosphere of N_2. The solution was stirred at room temperature for 20 minutes then a solution of 2-[2-[2-(*p*-tolylsulfonyloxy)ethoxy]ethoxy]ethyl 4-methylbenzenesulfonate (**71**) (490 mg, 1.1 mmol, 1.0 eq) in THF (0.54 mL) and DMF (0.20 mL) was added. The solution was stirred at room temperature for 23 h then poured into ice-water (15 mL) and extracted with EtOAc (3 × 25 mL). The combined organic layers were washed with brine (10 mL), dried over Na_2SO_4 and filtered, then adsorbed onto silica and purified by normal-phase flash chromatography (0–100%

EtOAc/cyclohexane) to afford the title compound (130 mg, 13%) as a colourless oil. The product was isolated as a ~1:1 mixture with di-*tert*-butyl 3,3'-(((ethane-1,2-diylbis(oxy))bis(ethane-2,1-diyl))bis(oxy))(3*R*,3'*R*)-bis(pyrrolidine-1-carboxylate).

***tert*-Butyl (3*R*)-3-[2-[2-[2-[2-(2,6-dioxo-3-piperidyl)-1,3-dioxo-isoindolin-4-yl]oxy ethoxy]ethoxy]ethoxy]pyrrolidine-1-carboxylate (9)**

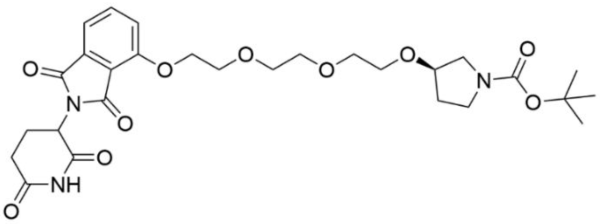

A solution of 2-(2,6-dioxopiperidin-3-yl)-4-hydroxyisoindoline-1,3-dione (**1**) (15 mg, 0.055 mmol, 1.0 eq), *tert*-butyl (3*R*)-3-[2-[2-[2-(*p*-tolylsulfonyloxy)ethoxy]ethoxy] ethoxy]pyrrolidine-1-carboxylate (**5**) (52 mg, 0.055 mmol, 1.0 eq), and $NaHCO_3$ (9.2 mg, 0.11 mmol, 2.0 eq) in DMF (0.36 mL) was stirred at 60 °C for 22 h. The solution was cooled to room temperature and diluted with H_2O (10 mL) then extracted with EtOAc (3 × 20 mL). The combined organic layers were washed with saturated aqueous $NaHCO_3$ (10 mL), H_2O (10 mL), and brine (10 mL). The solution was concentrated then purified by reversed-phase flash chromatography (20–80% MeOH containing 0.1% HCO_2H/H_2O containing 0.1% HCO_2H) to afford the title compound (21 mg, 65%) as a colourless oil. ^{1}H NMR (600 MHz, chloroform-*d*) δ 8.49 – 7.99 (m, 1H), 7.67 (dd, *J* = 8.5, 7.3 Hz, 1H), 7.47 (d, *J* = 7.3 Hz, 1H), 7.27 (dd, *J* = 8.6, 1.8 Hz, 1H), 4.95 (dd, *J* = 12.4, 5.4 Hz, 1H), 4.35 (t, *J* = 4.8 Hz, 2H), 4.11 – 4.03 (m, 1H), 3.95 (t, *J* = 4.8 Hz, 2H), 3.81 – 3.76 (m, 2H), 3.69 – 3.66 (m, 2H), 3.65 – 3.62 (m, 2H), 3.61 – 3.57 (m, 2H), 3.50 – 3.30 (m, 4H), 2.93 – 2.86 (m, 1H), 2.86 – 2.69 (m, 2H), 2.17 – 2.08 (m, 1H), 2.04 – 1.96 (m, 1H), 1.96 – 1.84 (m, 1H), 1.45 (s, 9H); ^{13}C NMR (151 MHz, chloroform-*d*) δ 171.0, 168.1, 167.1, 165.7, 156.6, 136.6, 133.9, 119.7, 117.5, 116.3, 79.4, 79.2 – 77.9 (m), 71.5 – 71.2 (m), 71.1 – 70.6 (m), 69.6 – 69.4 (m), 68.6, 51.9 – 50.6 (m), 49.3, 44.5 – 43.3 (m), 31.6, 30.6, 28.7, 22.8 (doubling up of some peaks in ^{13}C spectrum was observed due to the presence of rotamers, which was assigned via comparison to CCT397510); HRMS calc. for $C_{28}H_{37}NaN_3O_{10}$ $[M+Na]^+$ 598.2377, found 598.2370.

4-[2-[2-[2-[(3*R*)-1-[4-[[9-Chloro-7-(2-fluoro-6-methoxy-phenyl)-5*H*-pyrimido[5,4-*d*][2]benzazepin-2-yl]amino]-2-methoxy-benzoyl]pyrrolidin-3-yl]oxyethoxy]ethoxy]ethoxy]-2-(2,6-dioxo-3-piperidyl)isoindoline-1,3-dione (51)

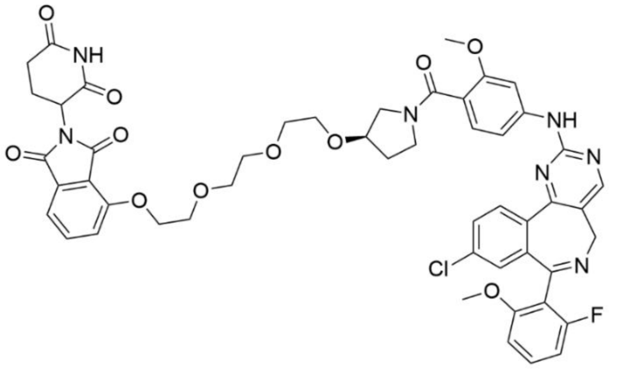

Trifluoroacetic acid (46 µL, 0.600 mmol, 20 eq) was added to a solution of *tert*-butyl (3*R*)-3-[2-[2-[2-[2-(2,6-dioxo-3-piperidyl)-1,3-dioxo-isoindolin-4-yl]oxyethoxy]ethoxy]ethoxy]pyrrolidine-1-carboxylate (**9**) (17 mg, 0.030 mmol, 1.0 eq) in CH_2Cl_2 (0.60 mL) and the solution was stirred at room temperature for 2 h then concentrated. The residue was dissolved in DMF (1.2 mL) and *O*-(benzotriazol-1-yl)-*N*,*N*,*N*',*N*'-tetramethyluronium hexafluorophosphate (11 mg, 0.030 mmol, 1.0 eq). The solution was stirred until everything had dissolved and Alisertib (15 mg, 0.028 mmol, 0.95 eq) and *N*,*N*-diisopropylethylamine (21 µL, 0.120 mmol, 4.0 eq) were added. The solution was stirred at room temperature for 19 h then concentrated on a Biotage V10. The residue was purified by reversed-phase flash chromatography (50–100% MeOH containing 0.1% HCO_2H/H_2O containing 0.1% HCO_2H) then normal-phase flash chromatography (0–9% MeOH/CH_2Cl_2) to afford the title compound (14 mg, 56%) as an off-white amorphous solid. ^{1}H NMR (600 MHz, DMSO-d_6) 11.09 (s, 1H), 10.03 (d, *J* = 3.3 Hz, 1H), 8.68 (d, *J* = 3.2 Hz, 1H), 8.28 (dd, *J* = 8.5, 3.3 Hz, 1H), 7.88 (br d, *J* = 7.0 Hz, 1H), 7.82 – 7.71 (m, 2H), 7.50 (dd, *J* = 26.0, 8.6 Hz, 1H), 7.45 – 7.38 (m, 2H), 7.35 (dd, *J* = 8.3, 1.9 Hz, 1H), 7.21 (br s, 1H), 7.12 (t, *J* = 7.9 Hz, 1H), 7.03 – 6.71 (br m, 2H), 5.12 – 5.03 (m, 1H), 4.86 (br s, 1H), 4.32 (dt, *J* = 28.0, 4.4 Hz, 2H), 4.14 – 4.00 (m, 1H), 3.83 – 3.78 (m, 4H), 3.78 – 3.74 (m, 1H), 3.67 – 3.63 (m, 1H), 3.62 – 3.58 (m, 1H), 3.58 – 3.37 (m, 9H), 3.29 – 3.22 (m, 1H), 3.21 – 3.14 (m, 1H), 2.87 (dddd, *J* = 17.2, 13.8, 5.5, 3.2 Hz, 1H), 2.58 (ddd, *J* = 17.2, 4.5, 2.6 Hz, 1H), 2.01 (ddd,

J = 12.7, 6.7, 3.8 Hz, 1H), 1.94 (td, *J* = 7.7, 3.3 Hz, 1H), 1.91 – 1.85 (m, 1H);‡‡‡‡‡ ^{13}C NMR (151 MHz, DMSO-d_6) δ 172.8, 169.9, 166.8 (d, *J* = 2.6 Hz), 166.7 (d, *J* = 9.2 Hz), 165.3 (d, *J* = 3.2 Hz), 160.9, 159.6 (d, *J* = 245.1 Hz), 159.5, 157.8, 157.1, 155.8 (d, *J* = 4.9 Hz), 155.4, 142.6, 137.7, 137.0 (d, *J* = 5.4 Hz), 135.2, 134.5, 133.2 (d, *J* = 3.2 Hz), 131.2, 130.9, 130.2, 128.1 (d, *J* = 18.1 Hz), 127.5, 123.1, 120.0, 119.9 (d, *J* = 12.3 Hz), 116.3 (d, *J* = 3.3 Hz), 115.4 (d, *J* = 3.8 Hz), 110.4, 108.3 – 107.5 (m), 101.7, 77.4 (d, *J* = 131.8 Hz), 70.2 (d, *J* = 11.7 Hz), 69.9 – 69.8 (m), 68.8 (d, *J* = 6.4 Hz), 68.7 (d, *J* = 5.7 Hz), 67.6 (d, *J* = 3.7 Hz), 56.3, 55.2 (d, *J* = 4.6 Hz), 51.6 (d, *J* = 186.5 Hz), 49.5, 48.7, 44.2 (d, *J* = 249.7 Hz), 30.9, 30.3 (d, *J* = 182.0 Hz), 21.99 (doubling up of some peaks in ^{13}C spectrum was observed due to the presence of rotamers, which was confirmed via EXSY NMR spectroscopy); HRMS calc. for $C_{50}H_{48}{}^{35}ClFN_7O_{11}$ [M+H]+ 976.3084, found 976.3087.

‡‡‡‡‡CH and OCH3 are not observed due to peak broadening. CH obscured under DMSO-d_6 residual solvent peak but observed in HSQC spectra.

Synthesis of 3-(4-methoxyphenyl)piperidine-2,6-dione (55)

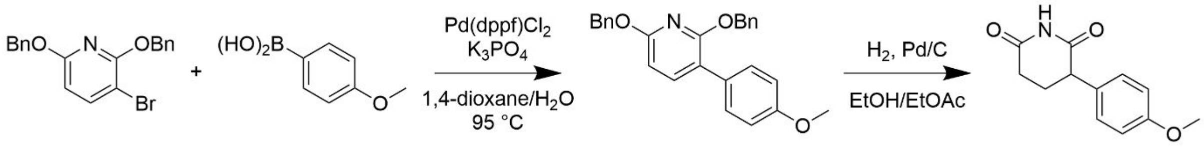

24 90 91 55

2,6-Bis(benzyloxy)-3-(4-methoxyphenyl)pyridine (91)

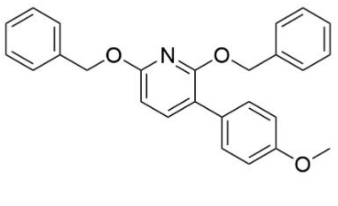

1,4-Dioxane (1.6 mL) and H_2O (0.40 mL) were added to a vial containing 2,6-dibenzyloxy-3-bromo-pyridine (**24**) (200 mg, 0.50 mmol, 1.0 eq), 4-methoxyphenylboronic acid (**90**) (92 mg, 0.60 mmol, 1.2 eq), and K_3PO_4 (130 mg, 0.60 mmol, 1.2 eq) under an atmosphere of N_2. Ar was bubbled through the solution for 5 minutes. [1,1'-Bis(diphenylphosphino)ferrocene]dichloropalladium(II), complex with dichloromethane (43 mg, 0.050 mmol, 0.1 eq) was added and the mixture was stirred at 95 °C for 19 h. The mixture was cooled to room temperature and diluted with EtOAc (40 mL). The mixture was washed with H_2O (20 mL) and brine (20 mL), adsorbed onto silica, and purified by normal-phase flash chromatography (0–45% CH_2Cl_2/cyclohexane) to afford the title compound (101 mg, 51%) as a yellow oil. ^1H NMR (600 MHz, chloroform-*d*) δ 7.58 (d, *J* = 8.0 Hz, 1H), 7.51 (d, *J* = 8.6 Hz, 2H), 7.44 (d, *J* = 7.6 Hz, 2H), 7.41 – 7.36 (m, 4H), 7.33 (dt, *J* = 9.7, 7.2 Hz, 3H), 7.30 – 7.27 (m, 1H), 6.94 (d, *J* = 8.6 Hz, 2H), 6.47 (d, *J* = 8.0 Hz, 1H), 5.43 (s, 2H), 5.37 (s, 2H), 3.84 (s, 3H); ^{13}C NMR (151 MHz, chloroform-*d*) δ 161.1, 158.6, 158.4, 141.6, 138.1, 137.8, 130.2, 129.3, 128.6, 128.5, 127.9, 127.5, 127.4, 115.9, 113.8, 102.5, 68.0, 67.7, 55.4; LCMS calc. for $C_{26}H_{23}NNaO_3$ $[M+Na]^+$ 420.1576, found 420.1580.

3-(4-Methoxyphenyl)piperidine-2,6-dione (55)

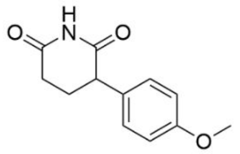

Pd (10% *w*/*w* on C, 26 mg, 0.025 mmol, 0.1 eq) was added to a solution of 2,6-dibenzyloxy-3-(4-methoxyphenyl)pyridine (**91**) (98 mg, 0.25 mmol, 1.0 eq) in EtOH

(1.2 mL) and EtOAc (1.2 mL) under an atmosphere of N_2. The reaction vessel was purged with H_2, and the mixture was stirred under an atmosphere of H_2 for four hours. The reaction vessel was purged with N_2, and the mixture was filtered through Celite. The solution was adsorbed onto silica and purified by normal-phase chromatography (0–6% MeOH/DCM) to afford the title compound (18 mg, 34%) as a white solid. ^{1}H NMR (600 MHz, DMSO-d_6) δ 10.79 (s, 1H), 7.13 (d, *J* = 8.6 Hz, 2H), 6.89 (d, *J* = 8.7 Hz, 2H), 3.78 (dd, *J* = 11.5, 4.9 Hz, 1H), 3.74 (s, 3H), 2.65 (ddd, *J* = 17.2, 11.7, 5.3 Hz, 1H), 2.52 – 2.44 (m, 1H)[§§§§§], 2.21 – 2.10 (m, 1H), 2.01 (dq, *J* = 13.7, 4.8 Hz, 1H). ^{13}C NMR (151 MHz, DMSO-d_6) δ 174.5, 173.4, 158.1, 131.0, 129.6, 113.7, 55.0, 46.5, 31.4, 26.0; LCMS calc. for $C_{12}H_{14}NO_3$ 220.0973, found 220.0969.

Synthesis of 1-(4-methoxyphenyl)dihydropyrimidine-2,4(1*H*,3*H*)-dione (56)

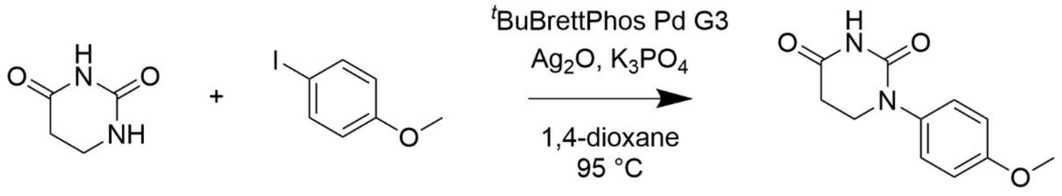

92 **93** **56**

1-(4-Methoxyphenyl)dihydropyrimidine-2,4(1*H*,3*H*)-dione (56)

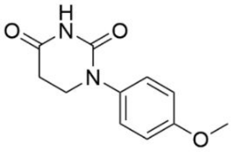

Adapted from a literature procedure.[15] 1,4-Dioxane (3.5 mL) was added to a vial containing 4-iodoanisole (**93**) (180 mg, 0.77 mmol, 1.0 eq), 5,6-dihydrouracil (**92**) (176 mg, 1.54 mmol, 2.0 eq), Ag_2O (196 mg, 0.85 mmol, 1.1 eq), and K_3PO_4 (331 mg, 1.54 mmol, 2.0 eq) under an atmosphere of N_2. Ar was bubbled through the solution for five minutes. *tert*-BuBrettPhos-Pd-G3 (33 mg, 0.039 mmol, 0.05 eq) was added and the mixture was stirred at 95 °C for 20 h. The mixture was cooled to room temperature and diluted with EtOAc (40 mL). The mixture was washed with H_2O (20 mL), saturated aqueous NH_4Cl (20 mL), and brine (20 mL). The organic layer was absorbed onto silica and purified by normal-phase flash chromatography (0–8% MeOH/DCM) and reversed-phase flash chromatography (10–100% MeOH containing 0.1%

§§§§§ CH partially obscured by DMSO-d_6 residual solvent peak.

HCO2H/H2O containing 0.1% HCO2H) to afford the title compound (33 mg, 19%) as a white solid. ^{1}H NMR (600 MHz, DMSO-d_6) δ 10.29 (s, 1H), 7.28 – 7.19 (m, 2H), 6.99 – 6.90 (m, 2H), 3.76 (s, 3H), 3.71 (t, J = 6.7 Hz, 2H), 2.69 (t, J = 6.7 Hz, 2H); ^{13}C NMR (151 MHz, DMSO-d_6) δ 170.7, 157.3, 152.3, 134.9, 126.9, 113. 9, 55.3, 44.9, 31.1; LCMS calc. for $C_{11}H_{13}N_2O_3$ [M+H]$^+$ 221.0926, found 221.0926.

Synthesis of 4-[[9-chloro-7-(2-fluoro-6-methoxy-phenyl)-5H-pyrimido[5,4-*d*][2]benzazepin-2-yl]amino]-2-methoxy-*N*-[5-[5-[4-(3-methyl-2,4-dioxo-hexahydropyrimidin-1-yl)phenoxy]pentoxy]pentyl]benzamide (57)

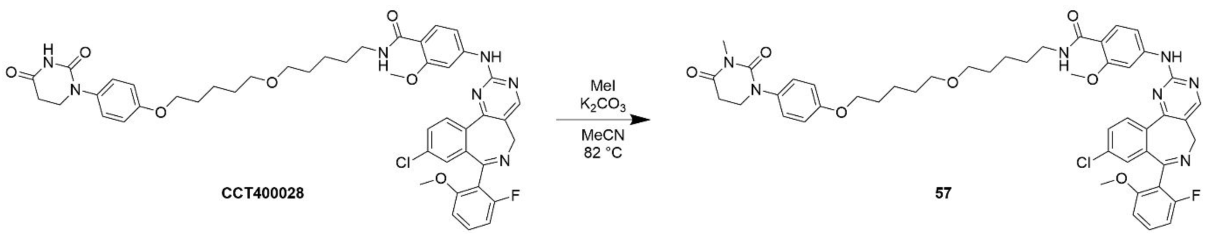

4-[[9-Chloro-7-(2-fluoro-6-methoxy-phenyl)-5H-pyrimido[5,4-*d*][2]benzazepin-2-yl]amino]-2-methoxy-*N*-[5-[5-[4-(3-methyl-2,4-dioxo-hexahydropyrimidin-1-yl)phenoxy]pentoxy]pentyl]benzamide (57)

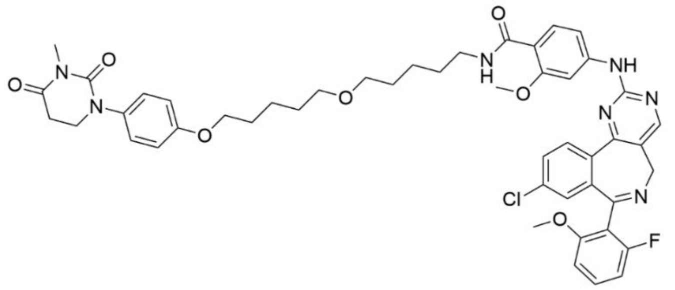

A mixture of 4-[[9-chloro-7-(2-fluoro-6-methoxy-phenyl)-5H-pyrimido[5,4-*d*][2]benzazepin-2-yl]amino]-*N*-[5-[5-[4-(2,4-dioxohexahydropyrimidin-1-yl)phenoxy]pentoxy]pentyl]-2-methoxy-benzamide (**CCT400028**) (15 mg, 0.017 mmol, 1.0 eq), iodomethane (1.3 µL, 0.020 mmol, 1.2 eq), and K_2CO_3 (7.0 mg, 0.051 mmol, 3.0 eq) in MeCN (0.4 mL) was stirred at 82 °C for 3 h. The solution was cooled to room temperature and purified by reversed-phase flash chromatography (50–100% MeOH containing 0.1% HCO_2H/H_2O containing 0.1% HCO_2H) then normal-phase flash chromatography (0–6% MeOH/DCM) to afford the title compound (4.2 mg, 27%) as a white solid. ^{1}H NMR (600 MHz, DMSO-d_6) δ 10.16 (s, 1H), 8.71 (s, 1H), 8.30 (d, *J* = 8.5 Hz, 1H), 8.02 (t, *J* = 5.7 Hz, 1H), 7.96 (s, 1H), 7.83 – 7.78 (m, 2H), 7.44 – 7.39 (m, 2H), 7.27 – 7.17 (m, 3H), 7.07 – 6.73 (m, 4H), 4.87 (s, 1H), 3.94 (t, *J* = 6.5 Hz, 2H), 3.91 (s, 3H), 3.67 (t, *J* = 6.7 Hz, 2H), 3.36 (td, *J* = 6.4, 2.5 Hz, 4H), 3.27 (q, *J* = 6.7 Hz, 2H)*, 3.02 (s, 3H), 2.79 (t, *J* = 6.7 Hz, 2H), 1.70 (p, *J* = 6.7 Hz, 2H), 1.57 – 1.49 (m,

6H), 1.47 – 1.40 (m, 2H), 1.38 – 1.32 (m, 2H);****** ^{13}C NMR (151 MHz, DMSO-d_6) δ 169.72, 164.30, 161.35, 159.55 (d, J = 246.1 Hz), 159.41, 157.71, 157.19, 156.76, 152.84, 144.35, 138.17, 135.31, 135.19, 134.88, 131.36, 131.15, 130.87, 130.32, 126.89, 123.37, 115.10, 114.41, 110.39, 108.03, 101.45, 69.89, 69.85, 67.58, 56.33, 55.66, 49.45, 43.86, 31.36, 29.07, 28.97, 28.49, 27.24, 23.27, 22.38. (5 carbons not visible); HRMS calc. for $C_{48}H_{52}{}^{35}ClFN_7O_7$ $[M+H]^+$ 892.3600, found 892.3627.

******CH partially obscured by DMSO-d6 residual water peak. Four alkyl CH protons missing due to broadening.

NMR Spectra of Synthesized Compounds

CCT400028 - ^{1}H NMR (600 MHz, DMSO-d_6) and ^{13}C NMR (151 MHz, DMSO-d_6)

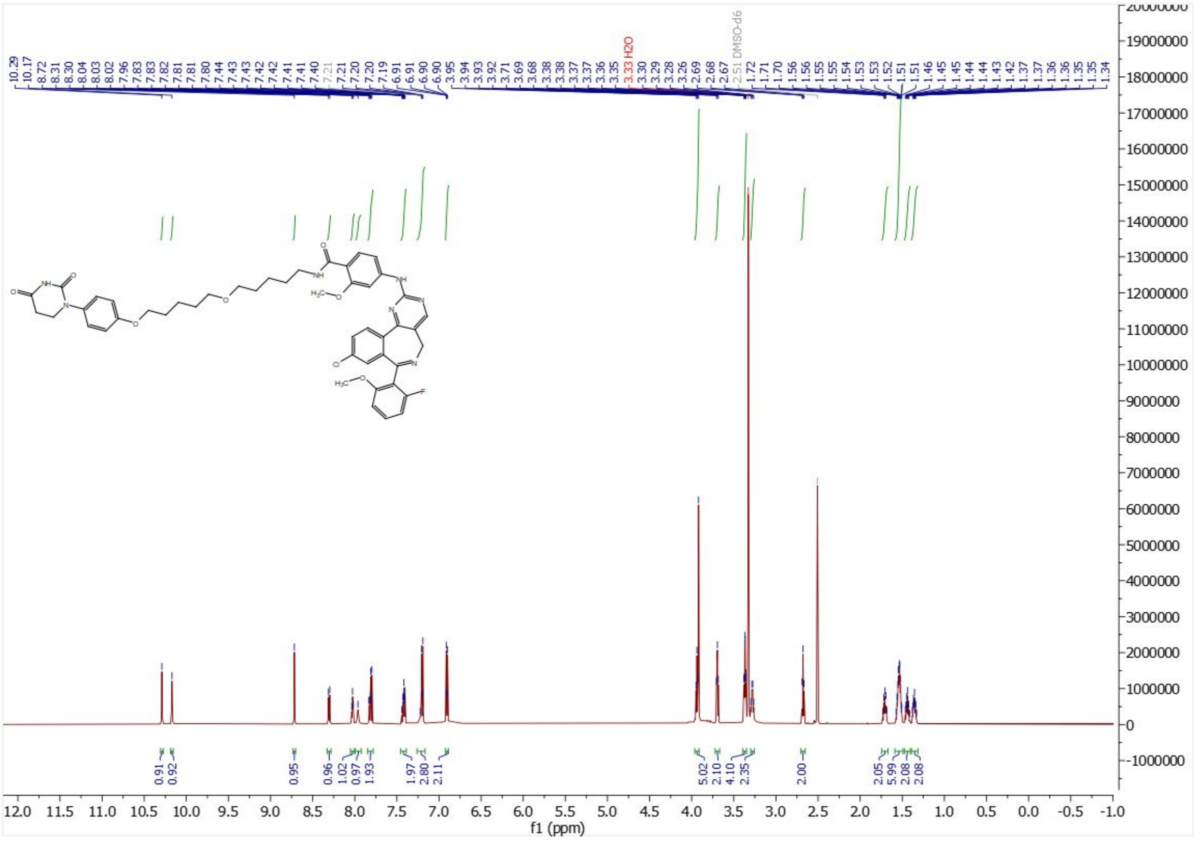

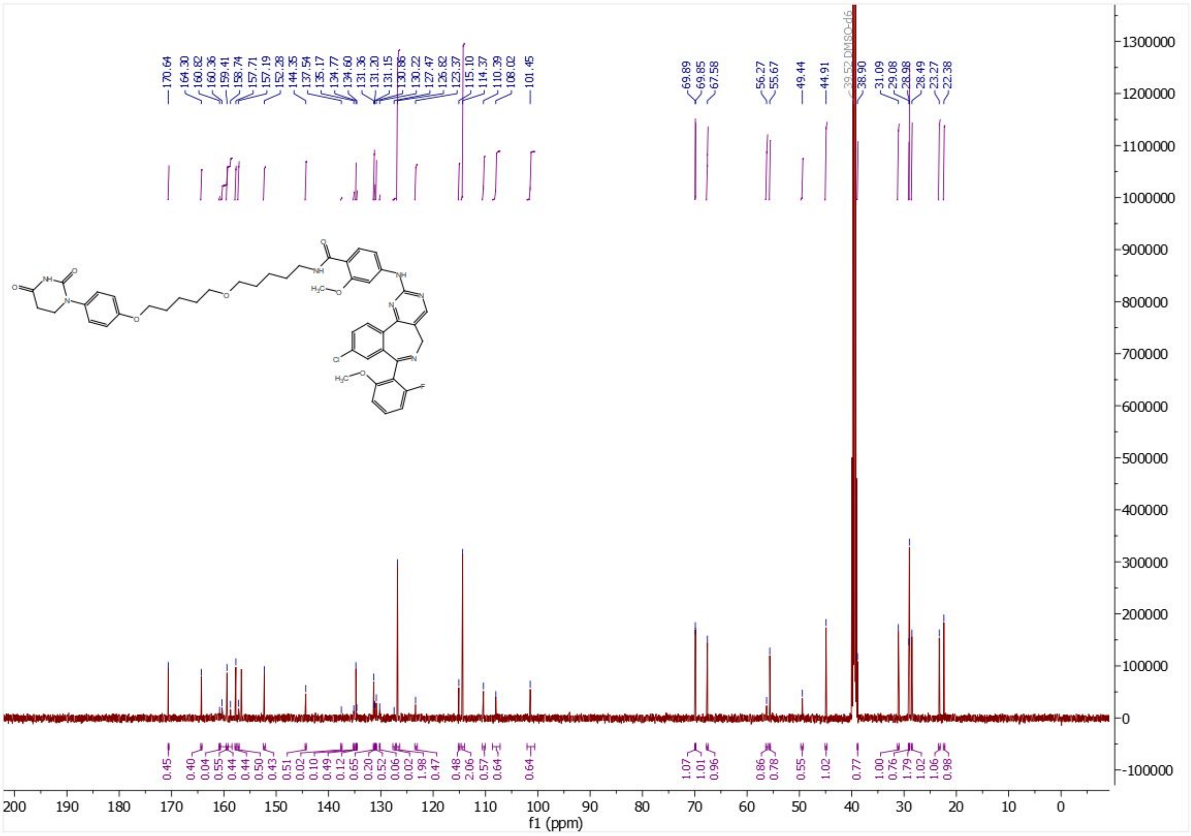

42 - ^{1}H NMR (500 MHz, DMSO-d_6) and ^{13}C NMR (126 MHz, DMSO-d_6)

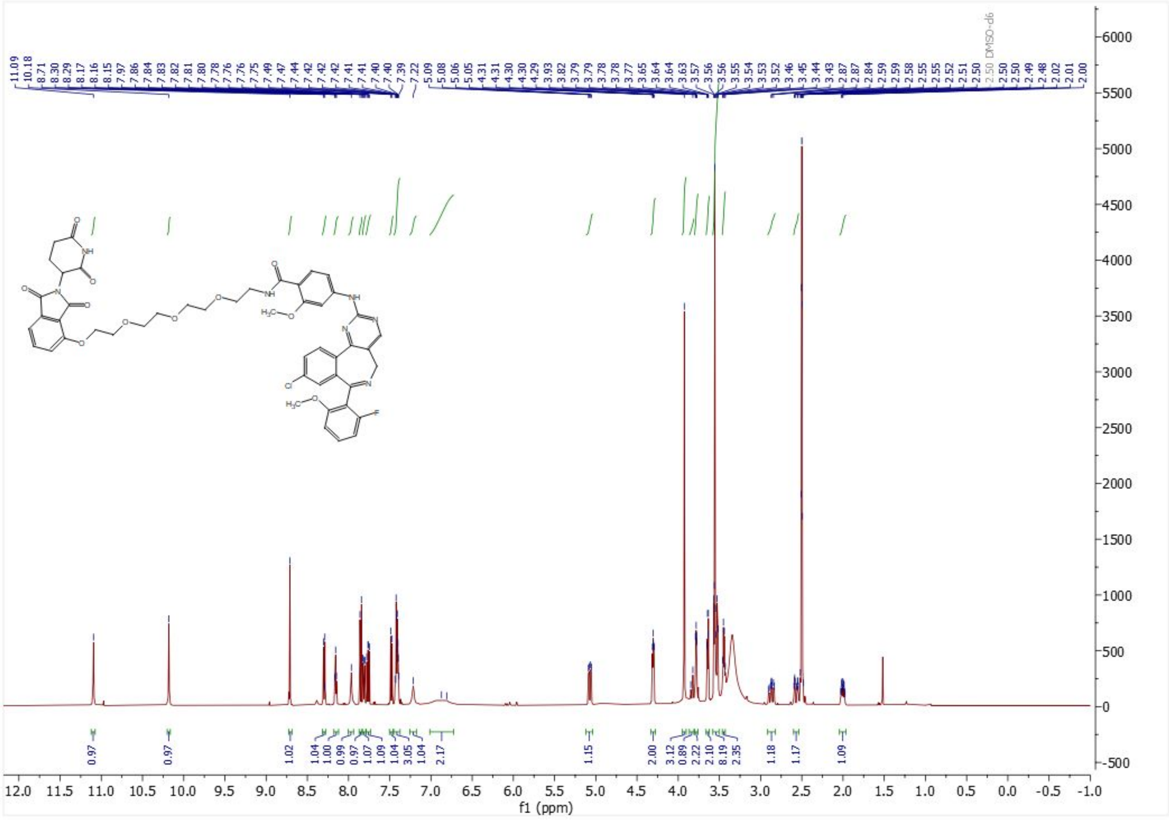

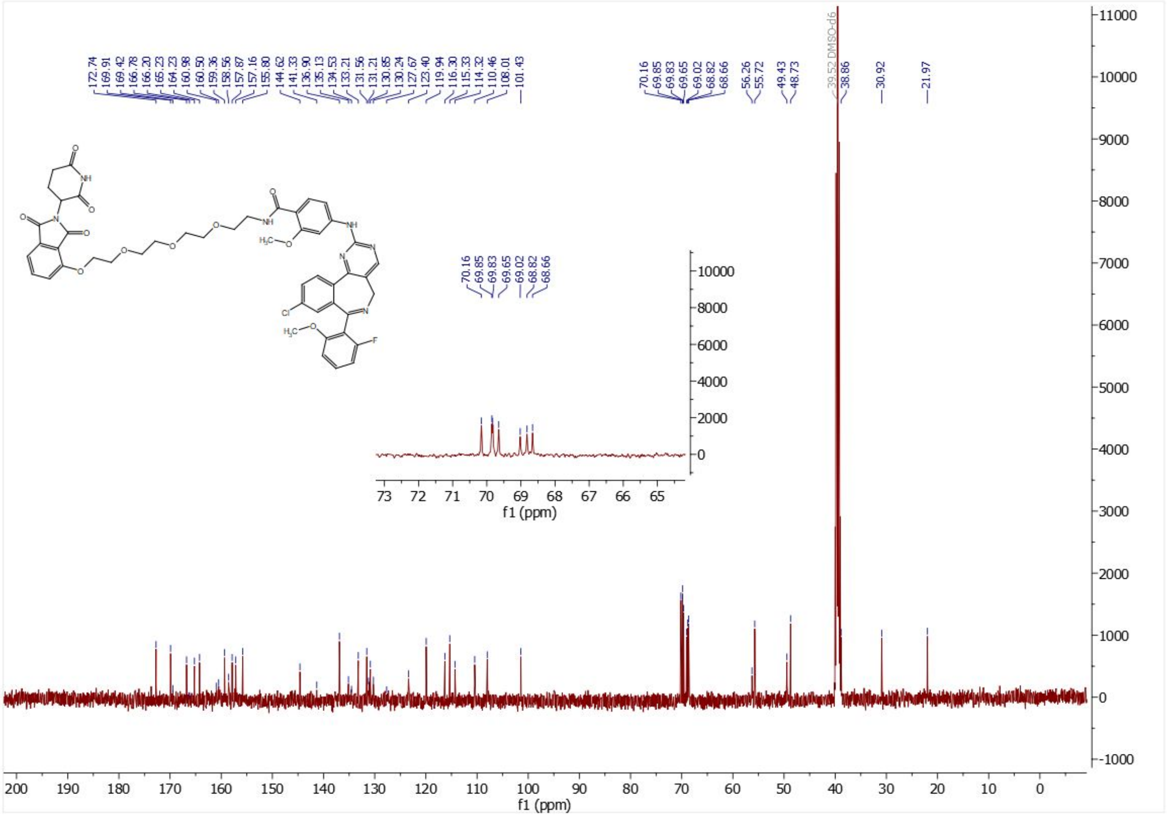

43 - ^{1}H NMR (600 MHz, DMSO-d_6) and ^{13}C NMR (151 MHz, DMSO-d_6)

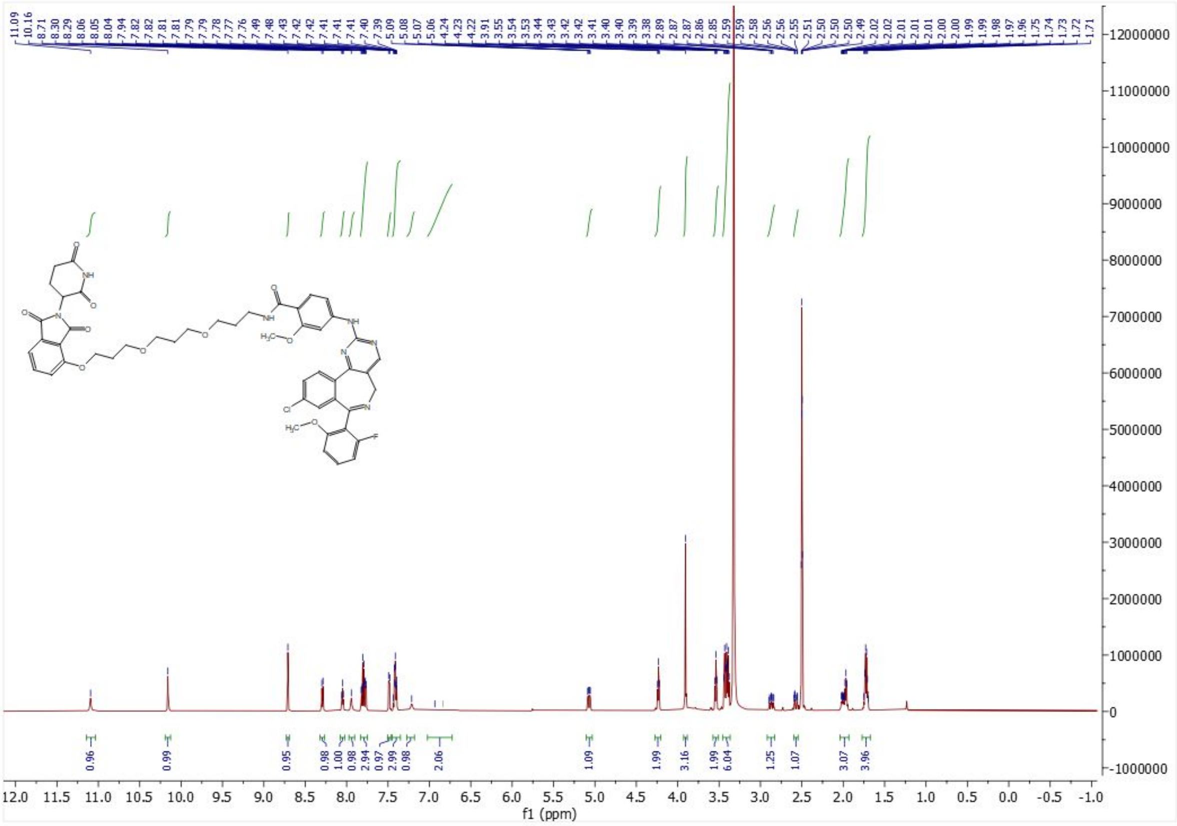

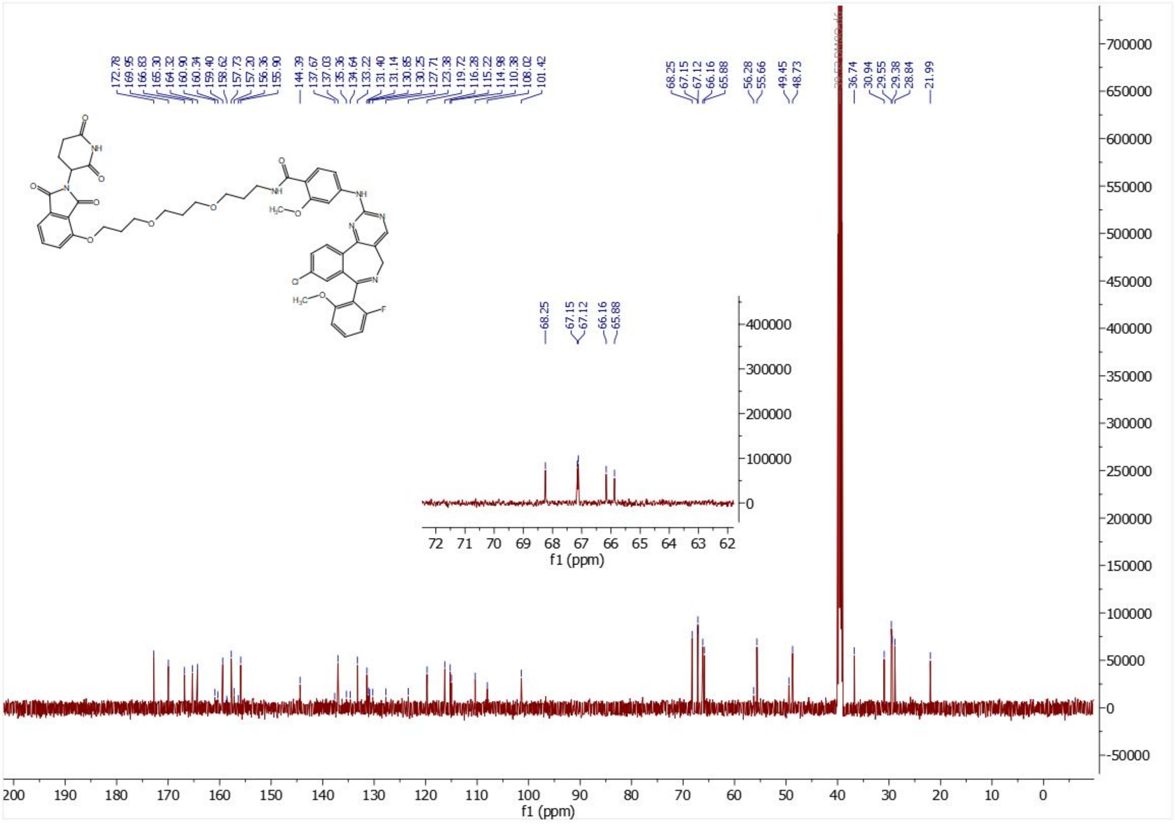

44 - ^{1}H NMR (500 MHz, DMSO-d_6) and ^{13}C NMR (126 MHz, DMSO-d_6)

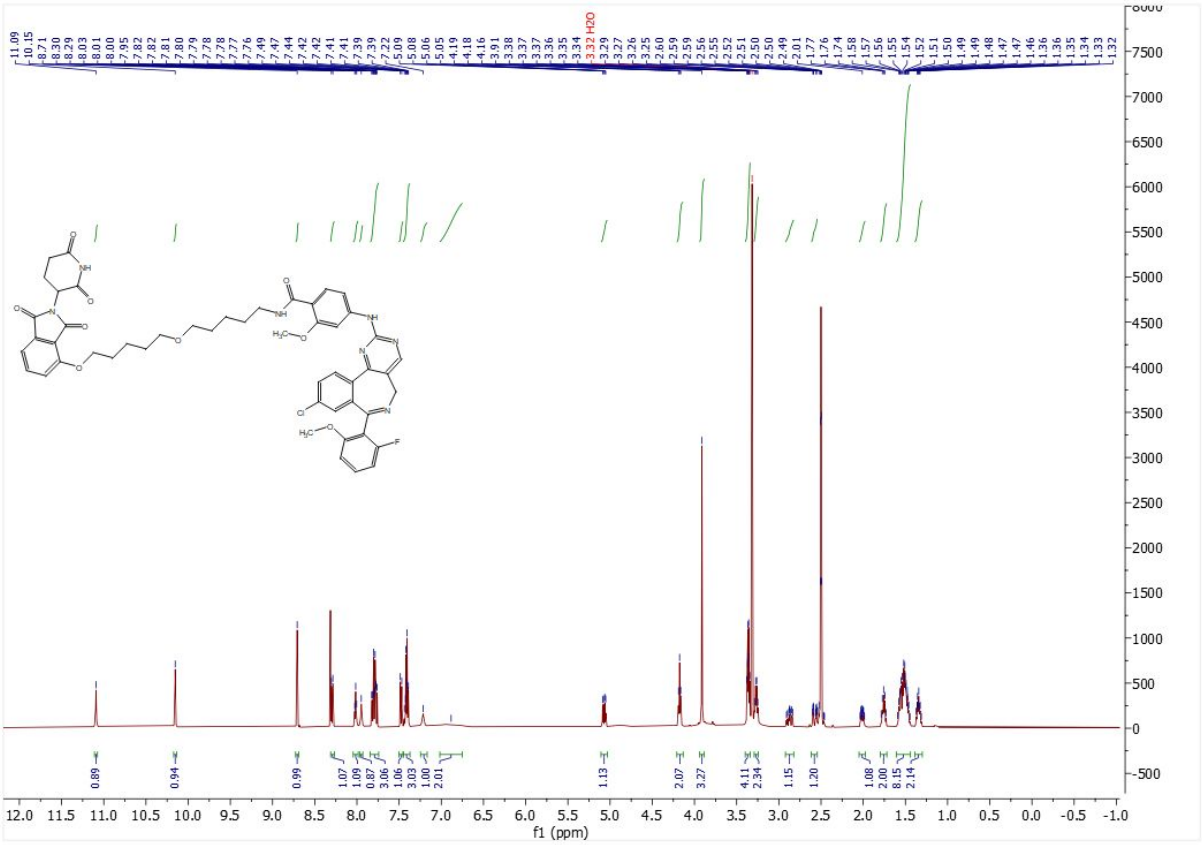

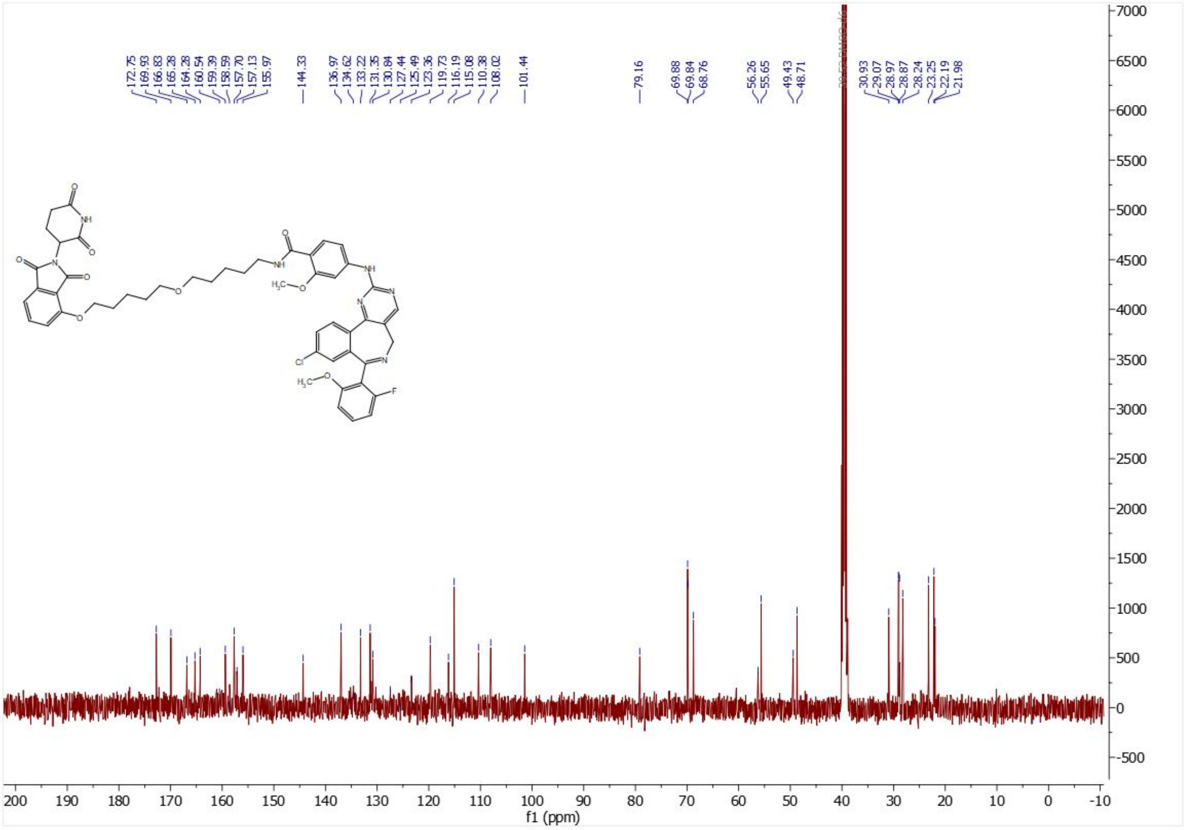

45 - ^{1}H NMR (600 MHz, DMSO-d_6) and ^{13}C NMR (151 MHz, DMSO-d_6)

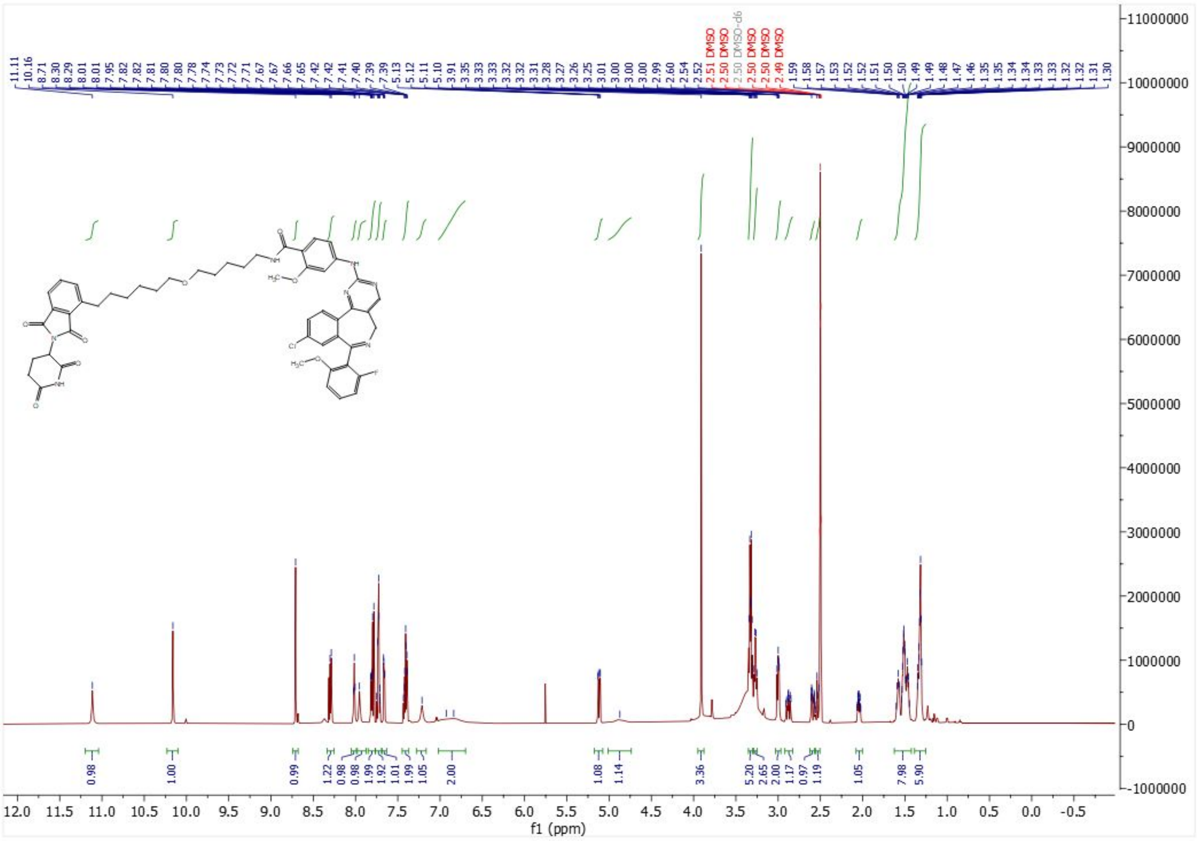

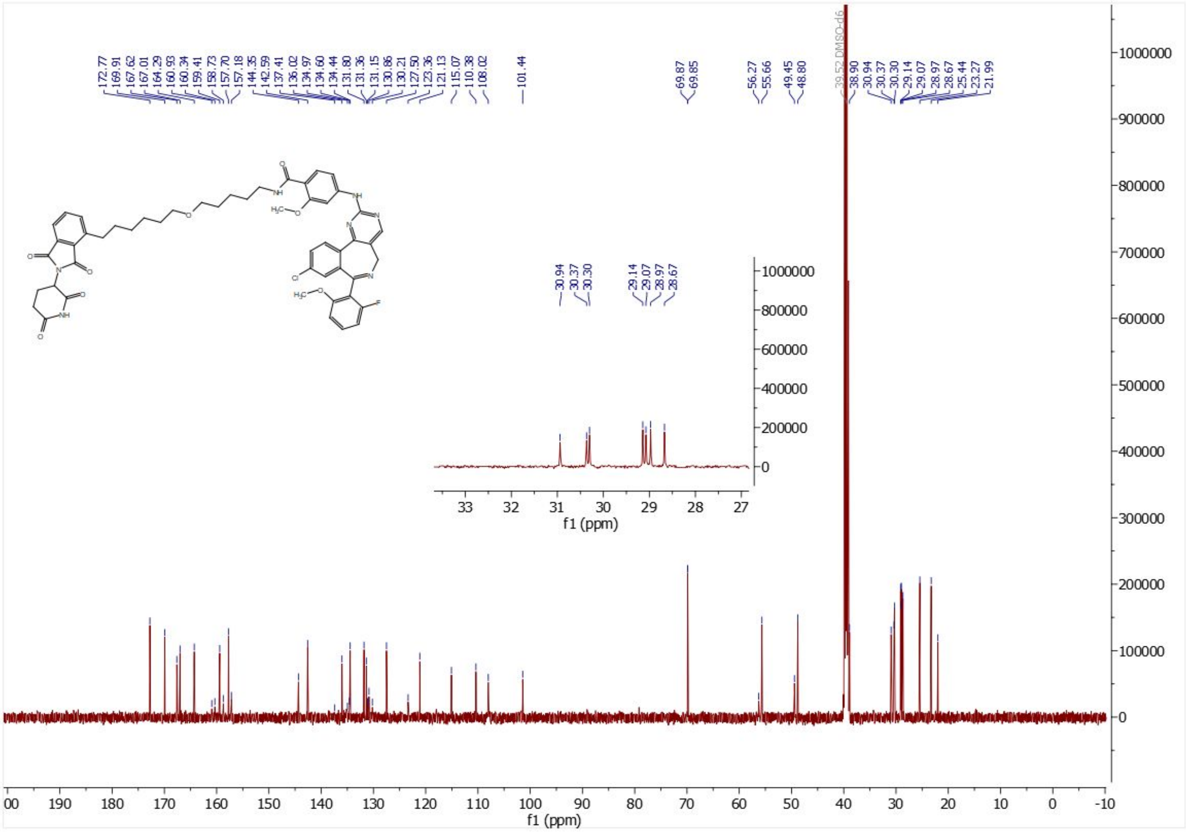

46 - ^{1}H NMR (600 MHz, DMSO-d_6) and ^{13}C NMR (151 MHz, DMSO-d_6)

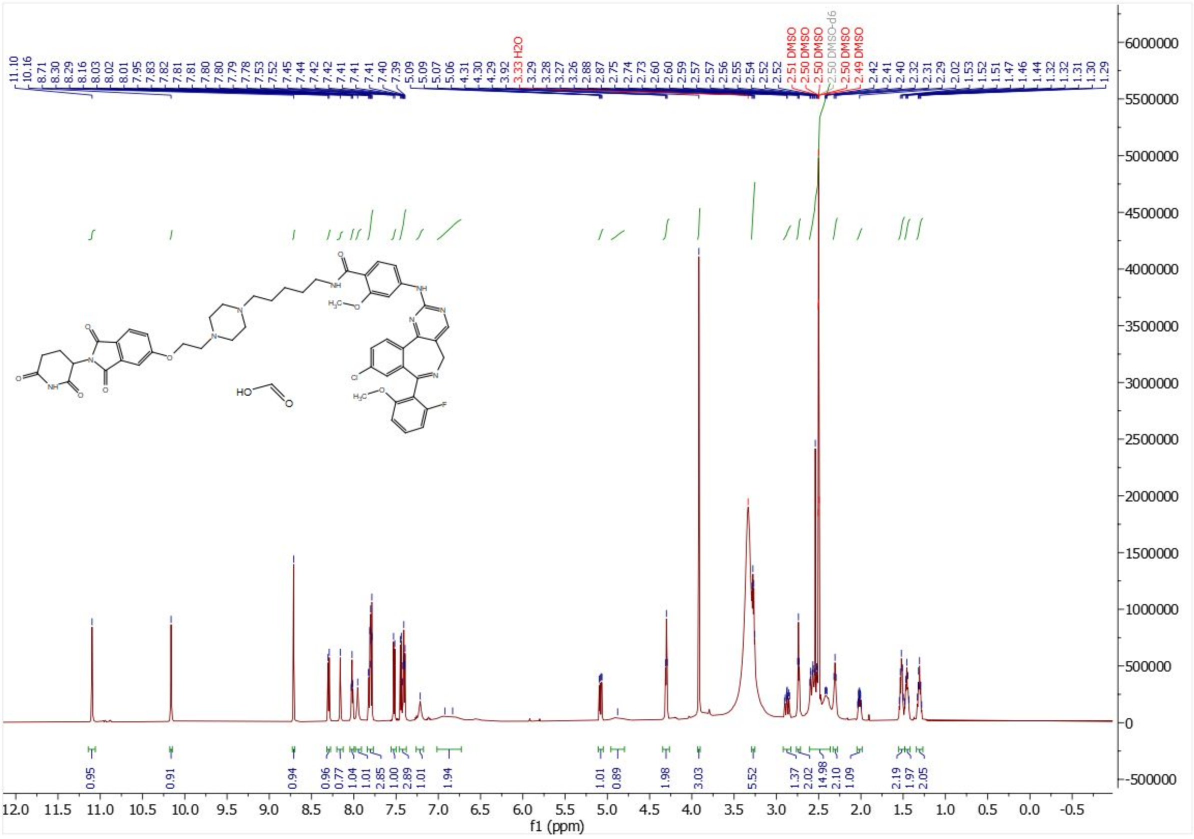

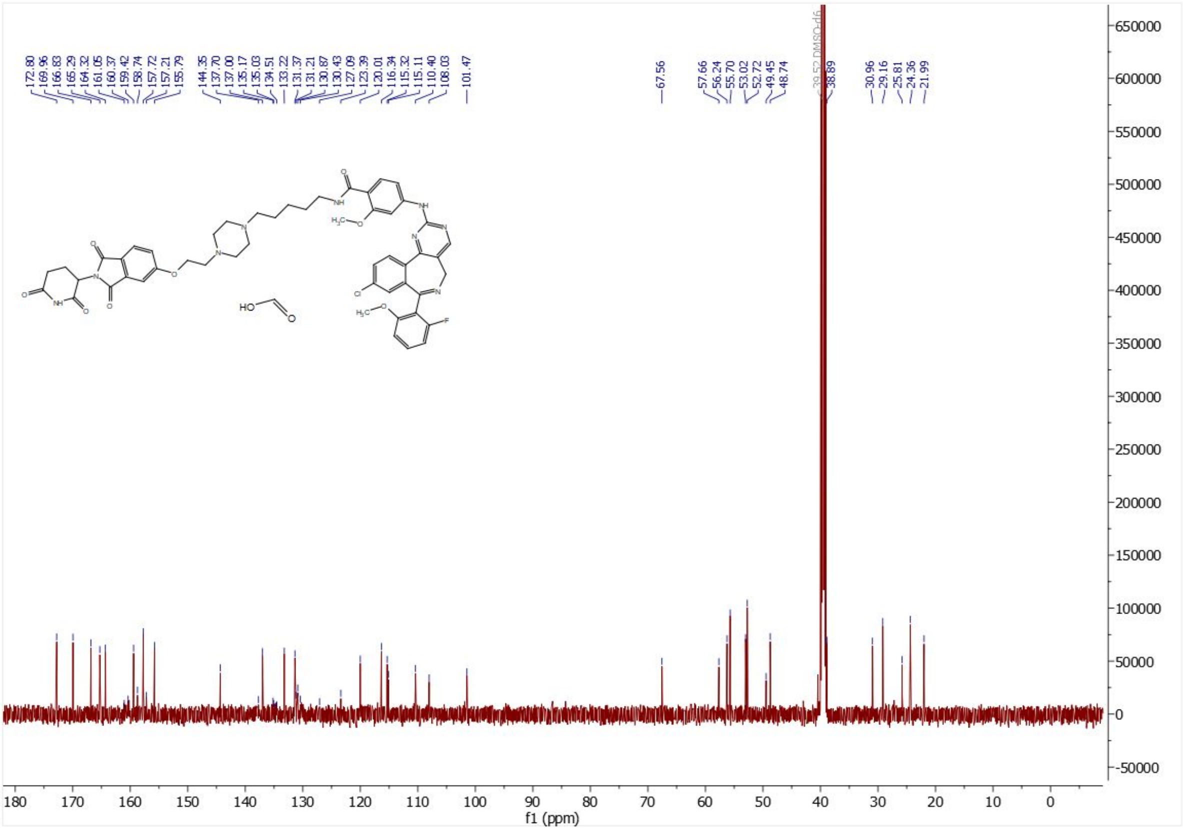

47 - ^{1}H NMR (600 MHz, DMSO-d_6) and ^{13}C NMR (151 MHz, DMSO-d_6)

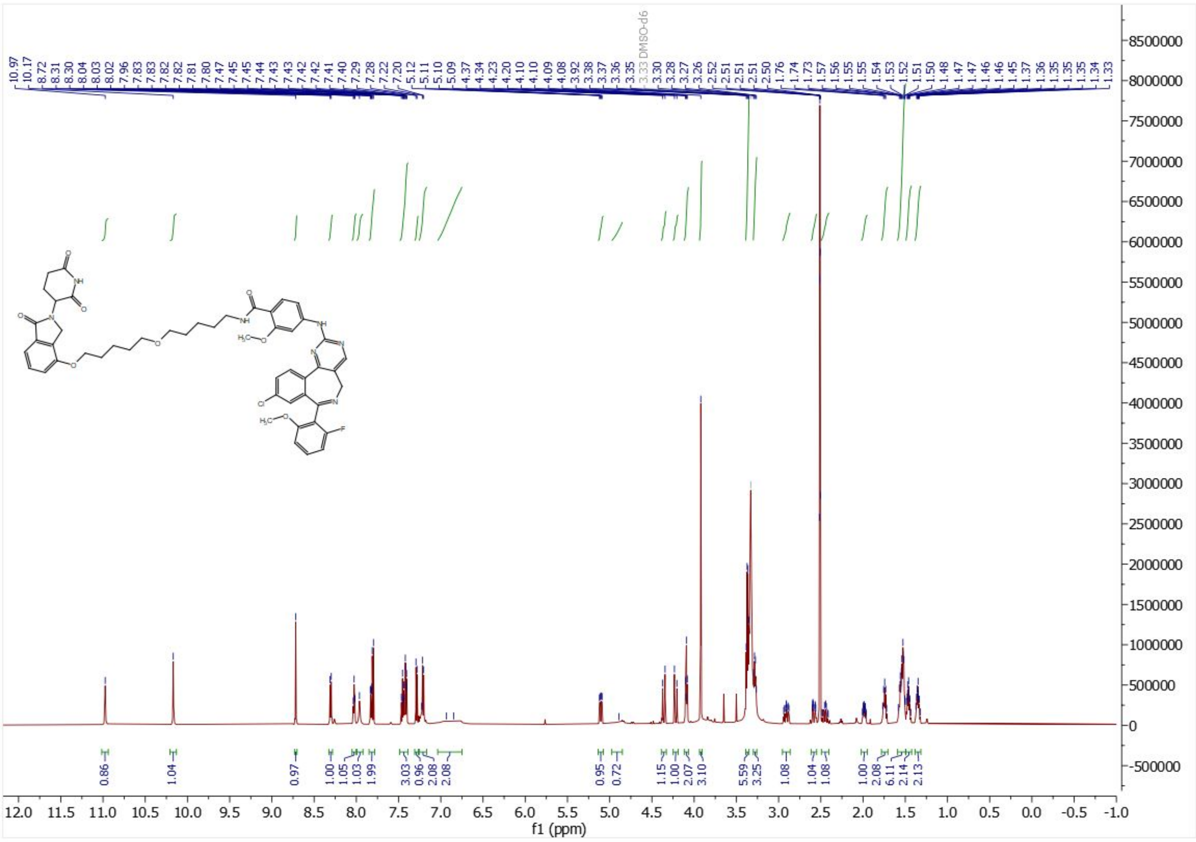

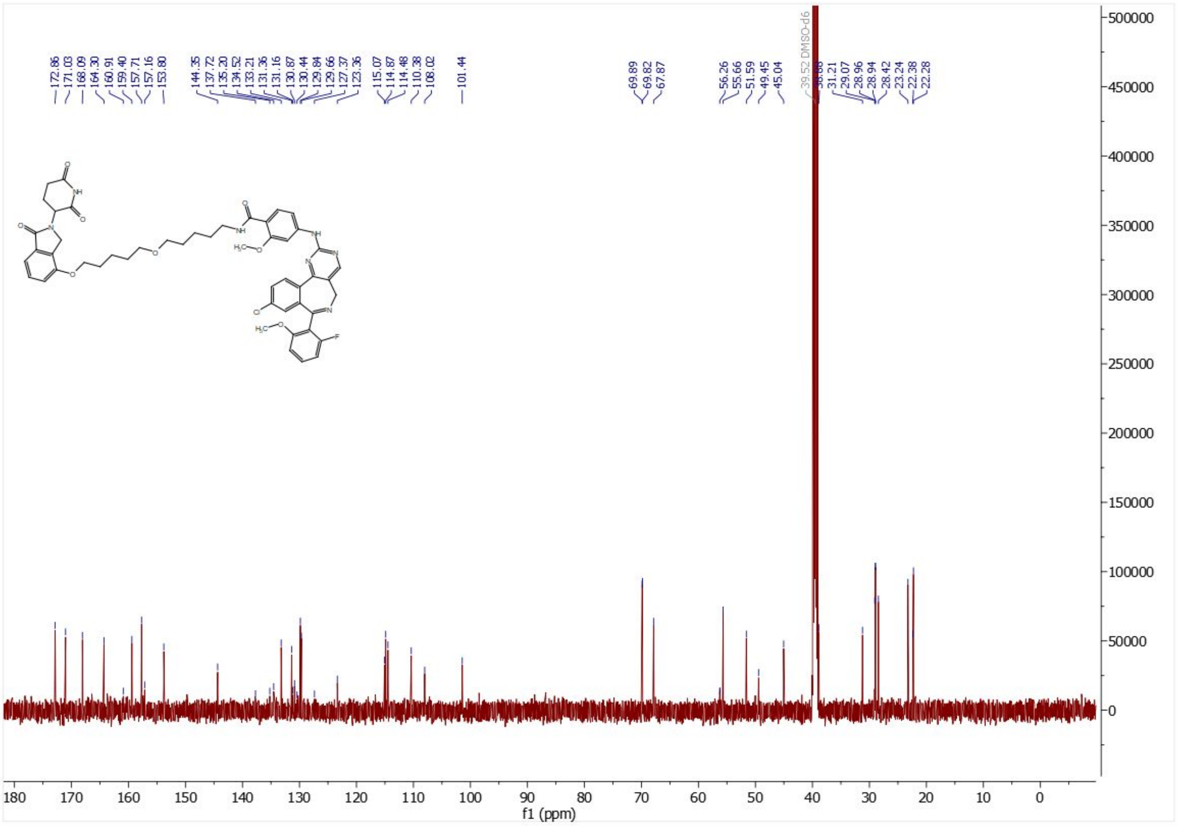

48 - ^{1}H NMR (600 MHz, DMSO-d_6) and ^{13}C NMR (151 MHz, DMSO-d_6)

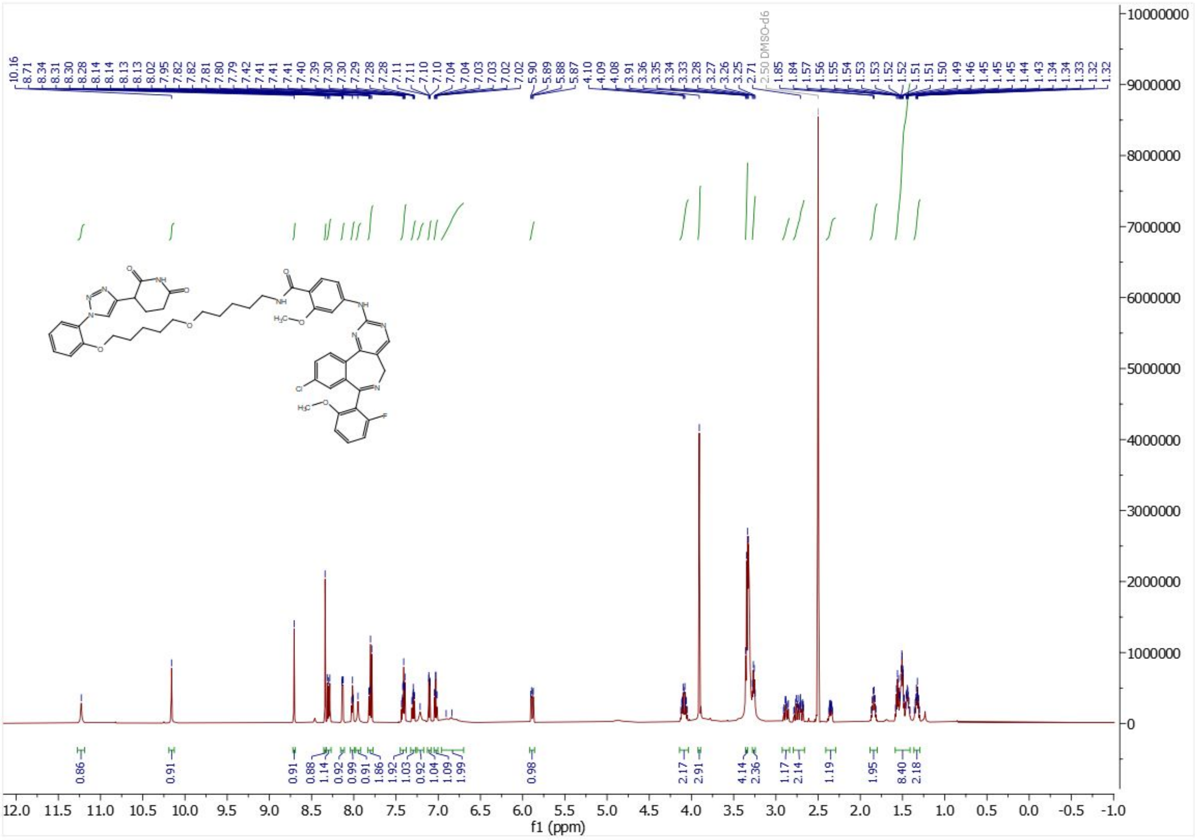

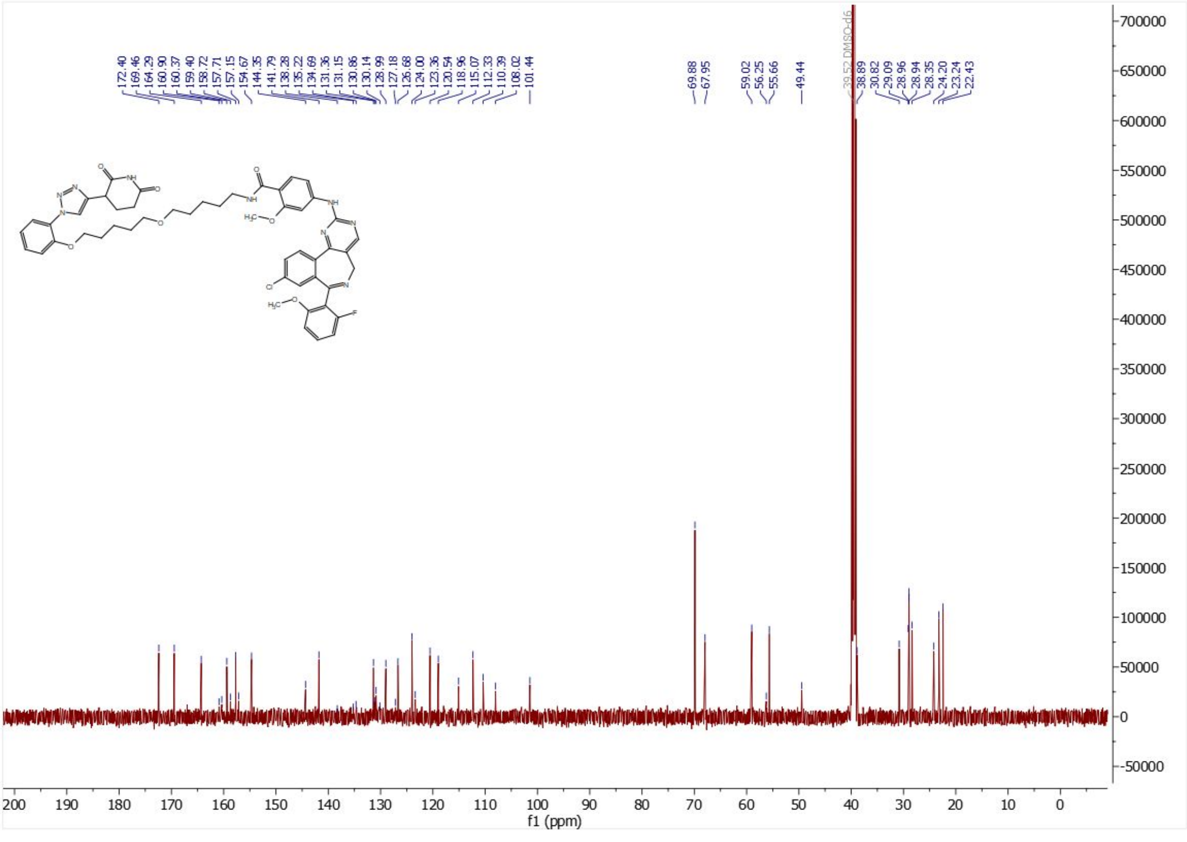

49 - ^{1}H NMR (600 MHz, DMSO-d_6) and ^{13}C NMR (151 MHz, DMSO-d_6)

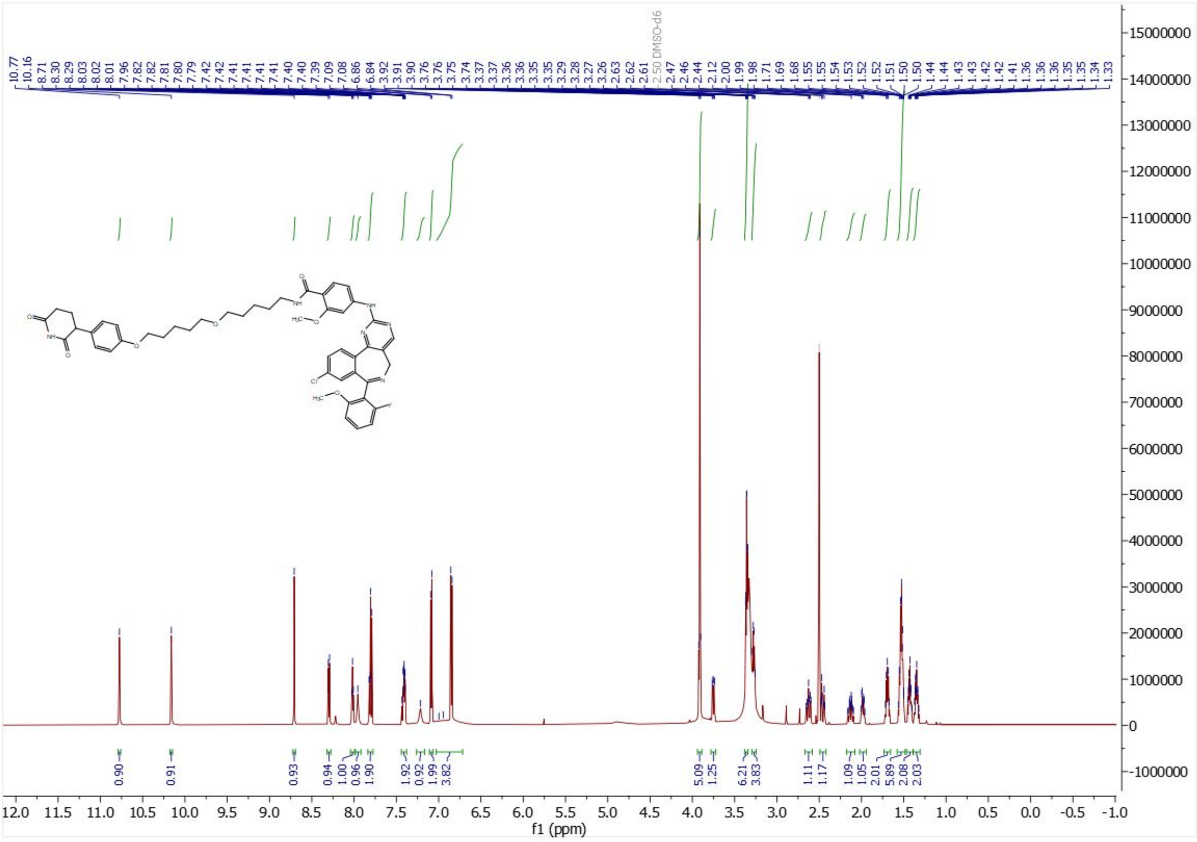

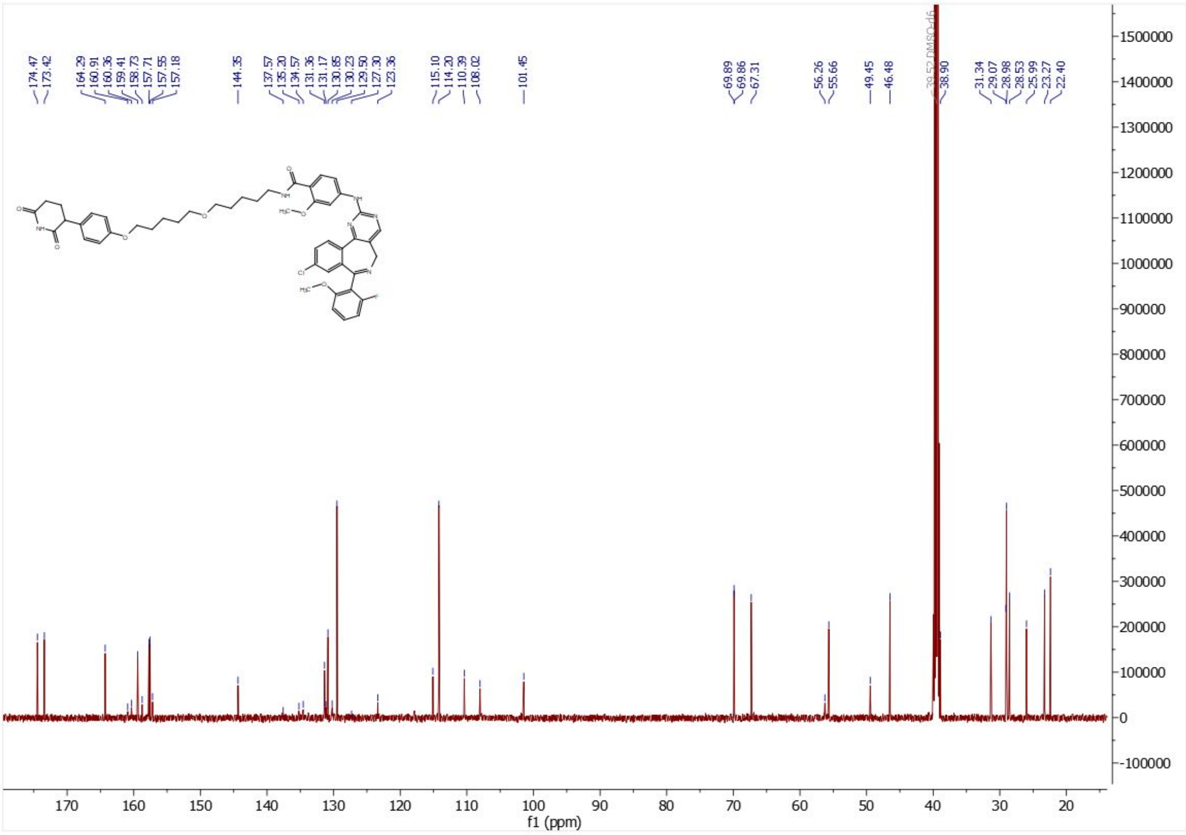

50 - ^{1}H NMR (600 MHz, DMSO-d_6) and ^{13}C NMR (151 MHz, DMSO-d_6)

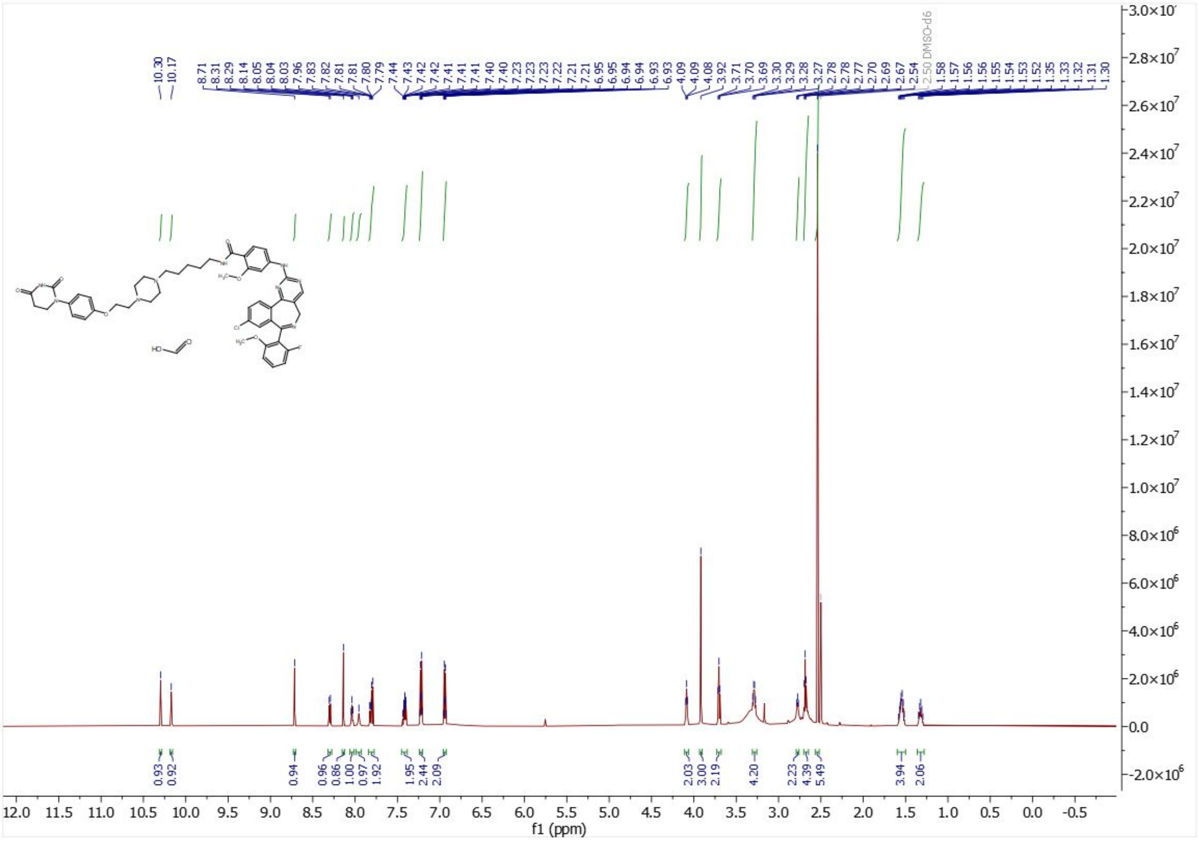

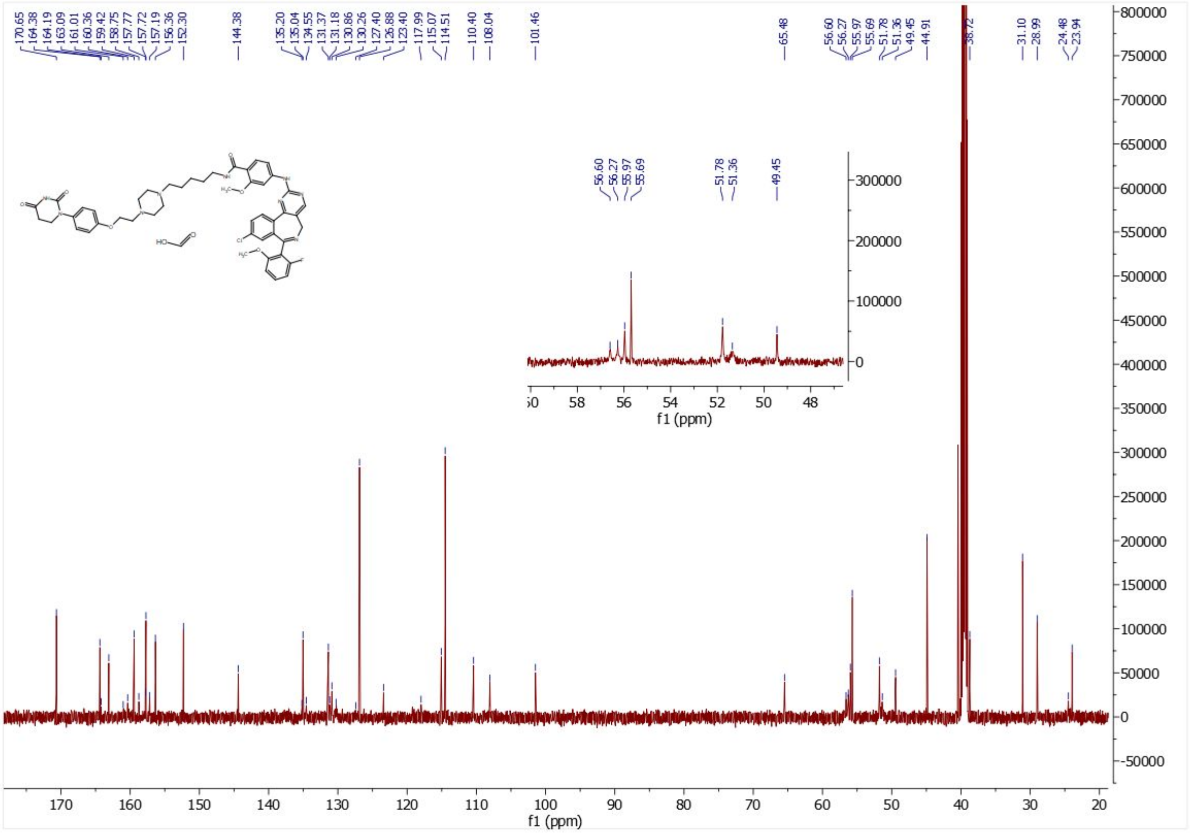

51 - ^{1}H NMR (600 MHz, DMSO-d_6) and ^{13}C NMR (151 MHz, DMSO-d_6)

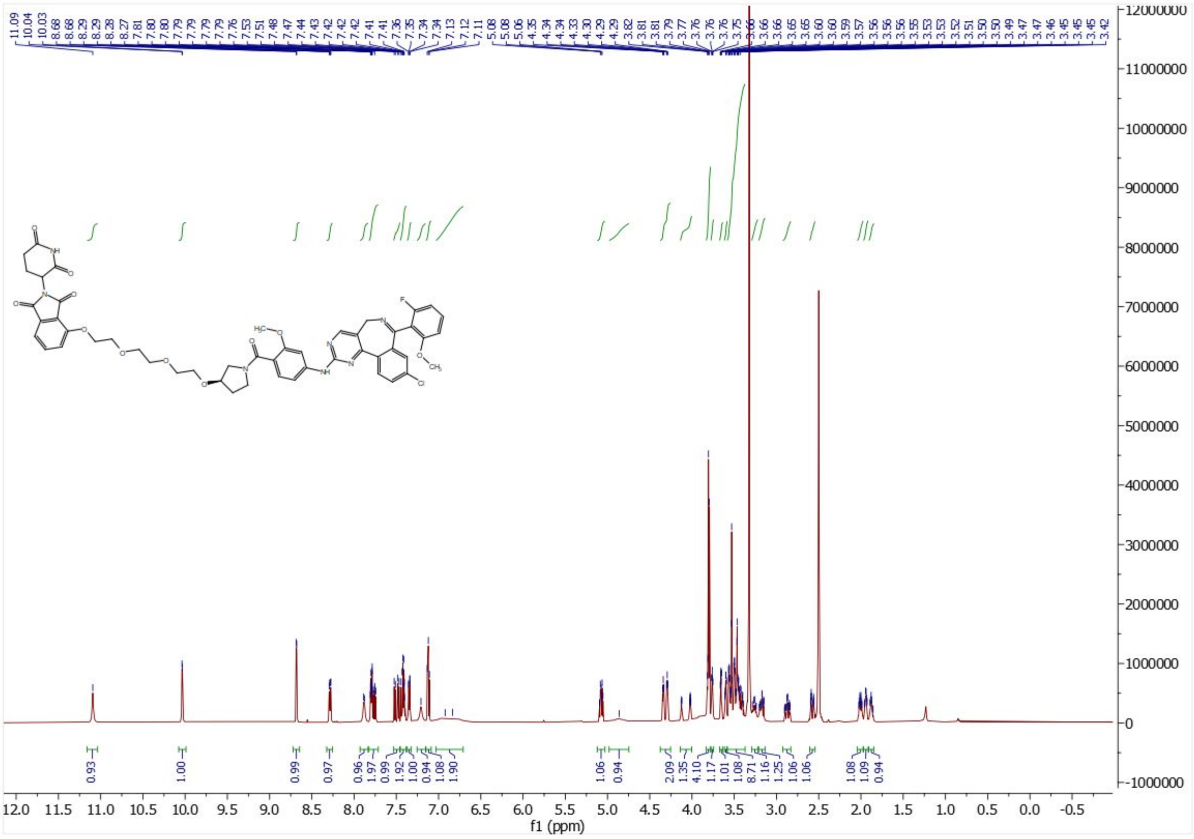

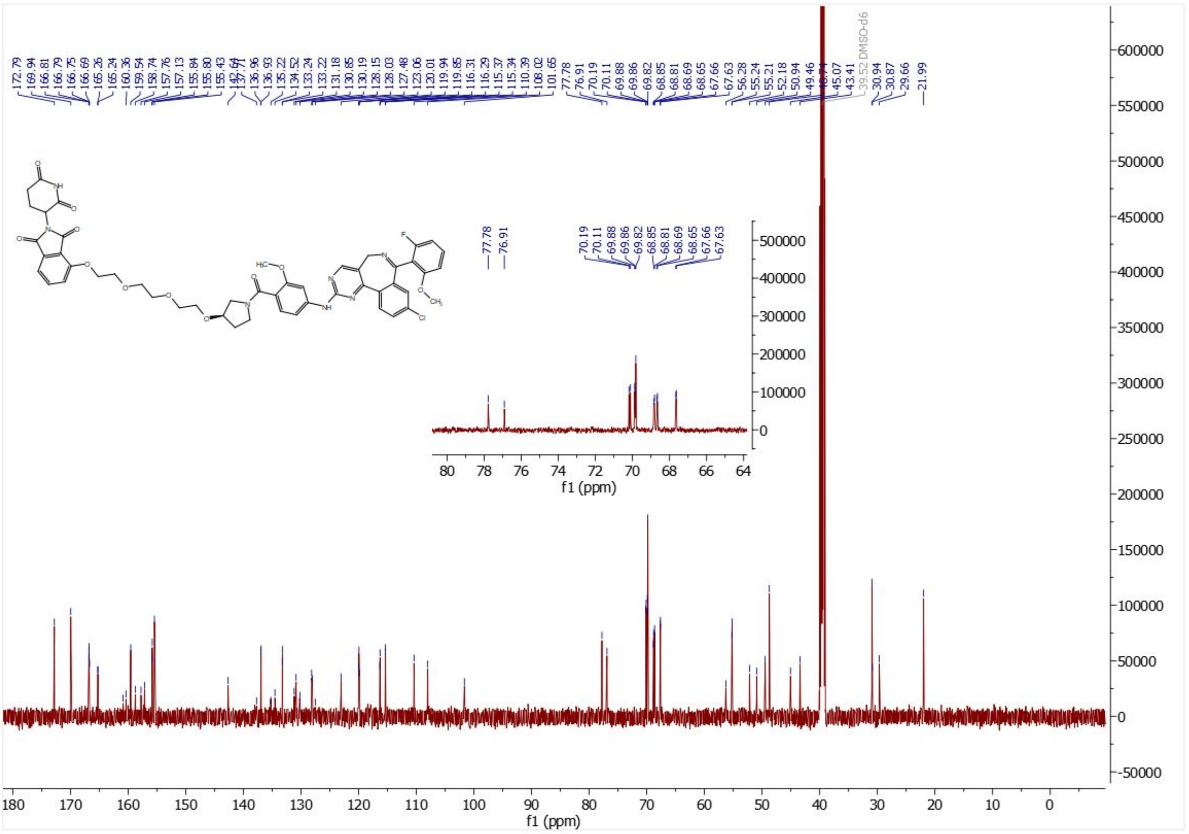

51 – NOESY (600 MHz, DMSO-d_6)

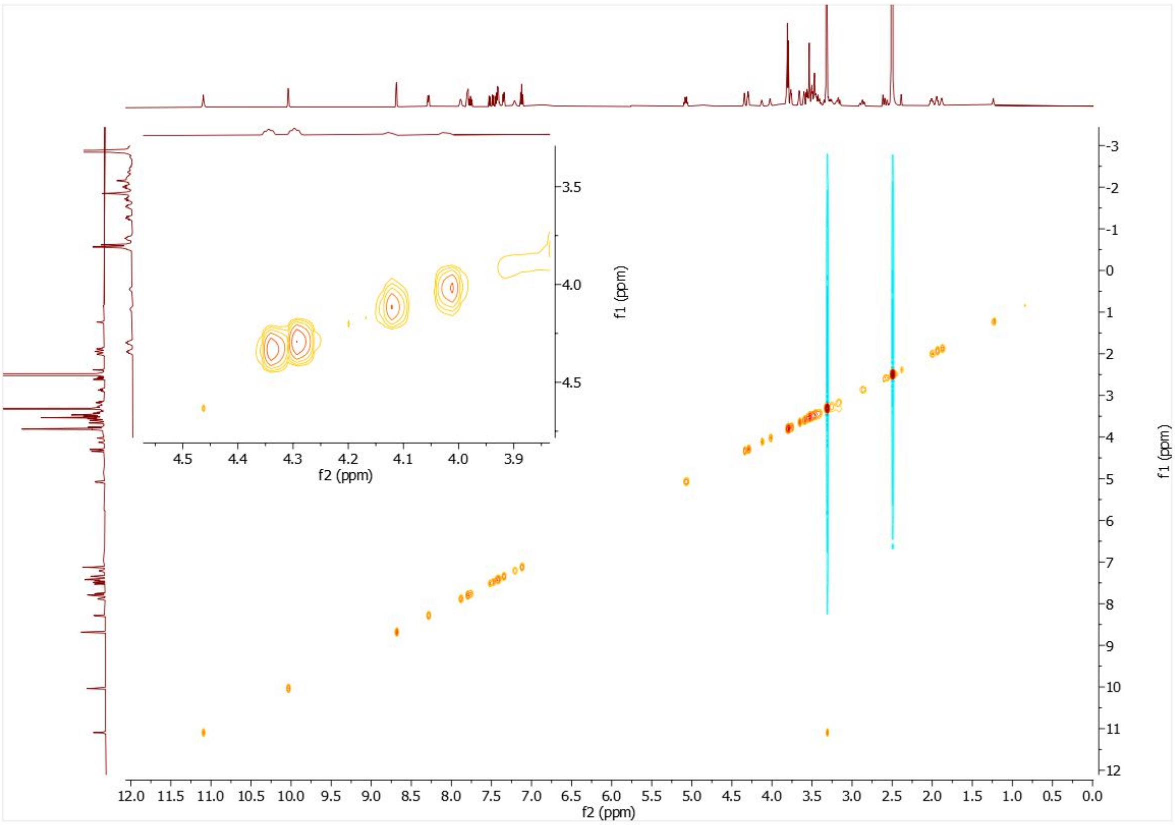

51 – EXSY (600 MHz, DMSO-d_6)

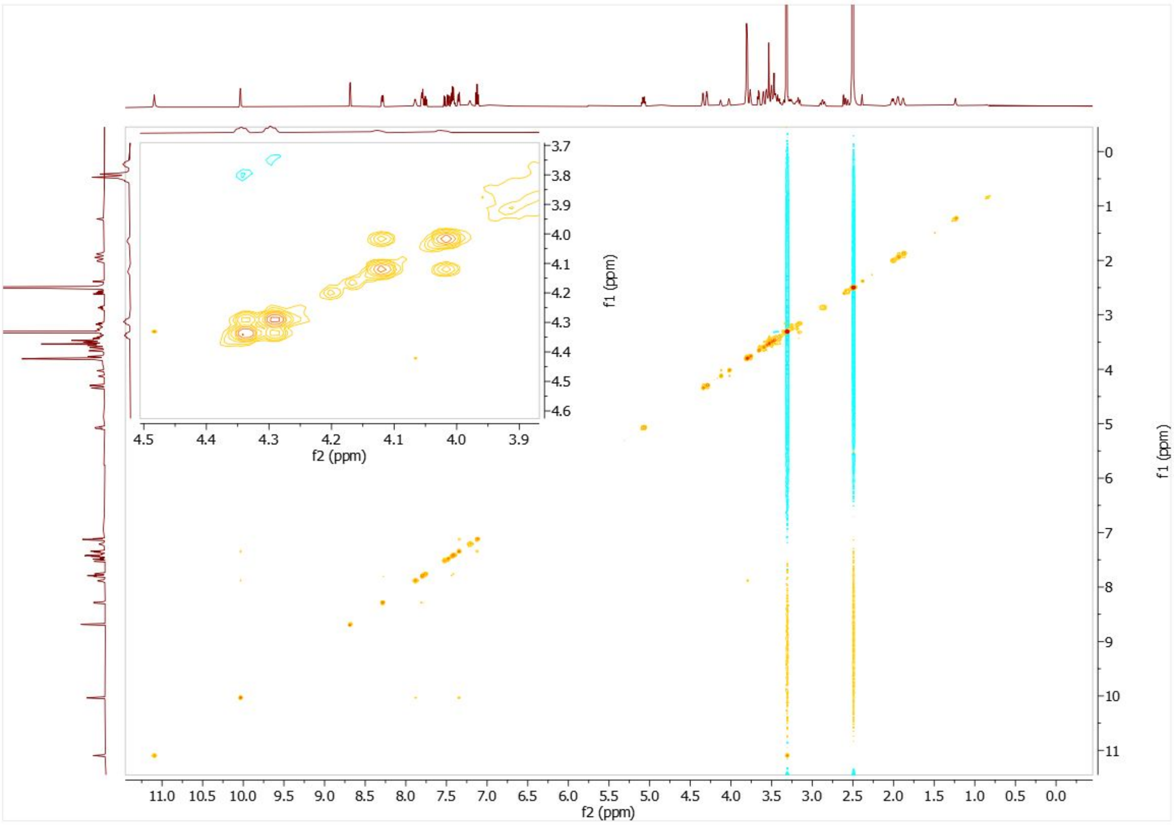

55 – ^{1}H NMR (500 MHz, DMSO-d_6) and ^{13}C NMR (126 MHz, DMSO-d_6)

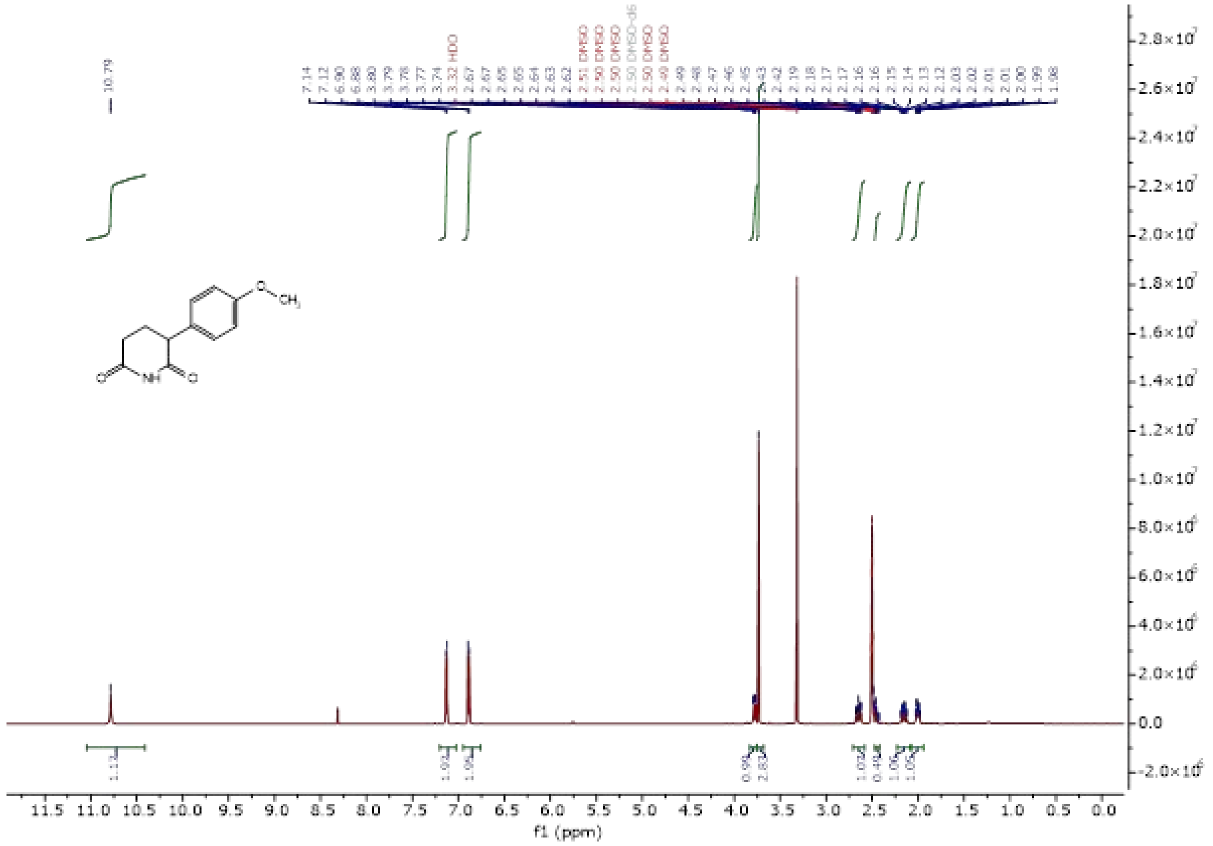

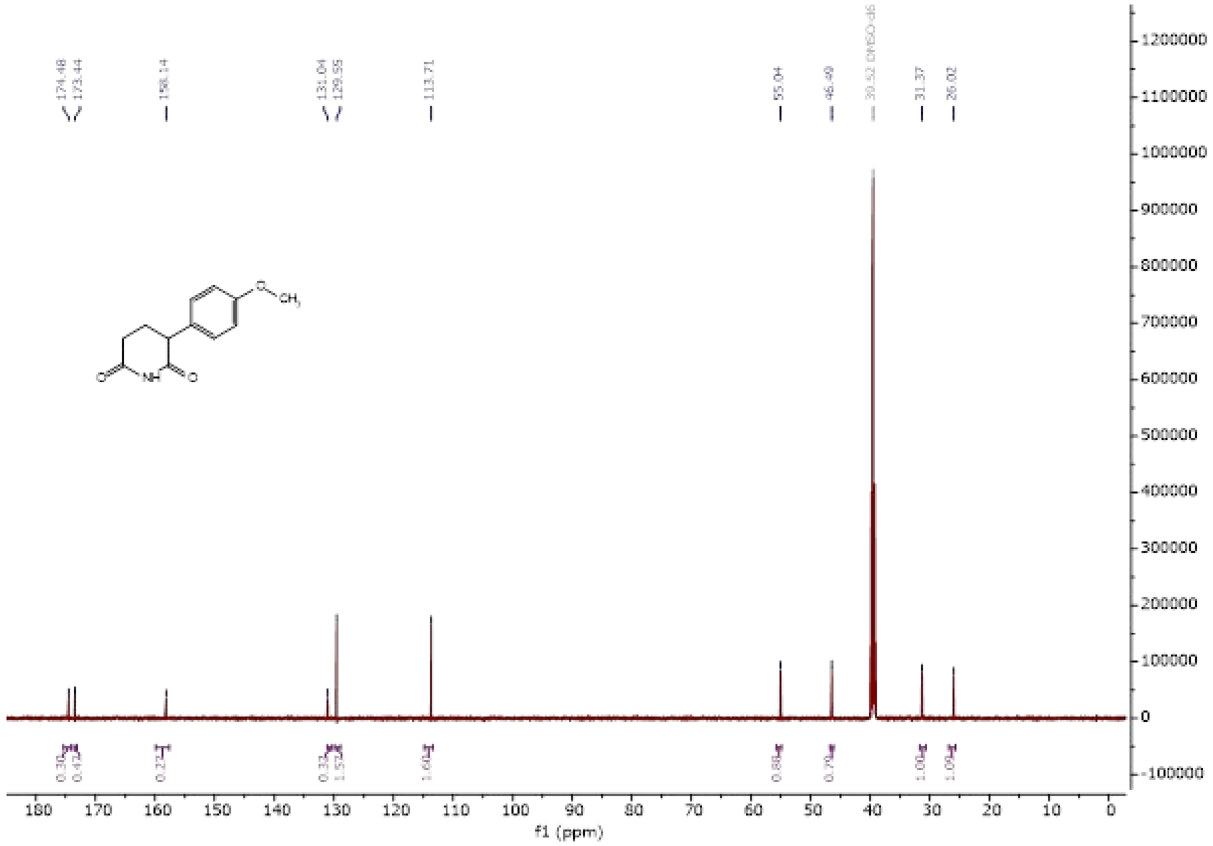

56 – ^{1}H NMR (500 MHz, DMSO-d_6) and ^{13}C NMR (126 MHz, DMSO-d_6)

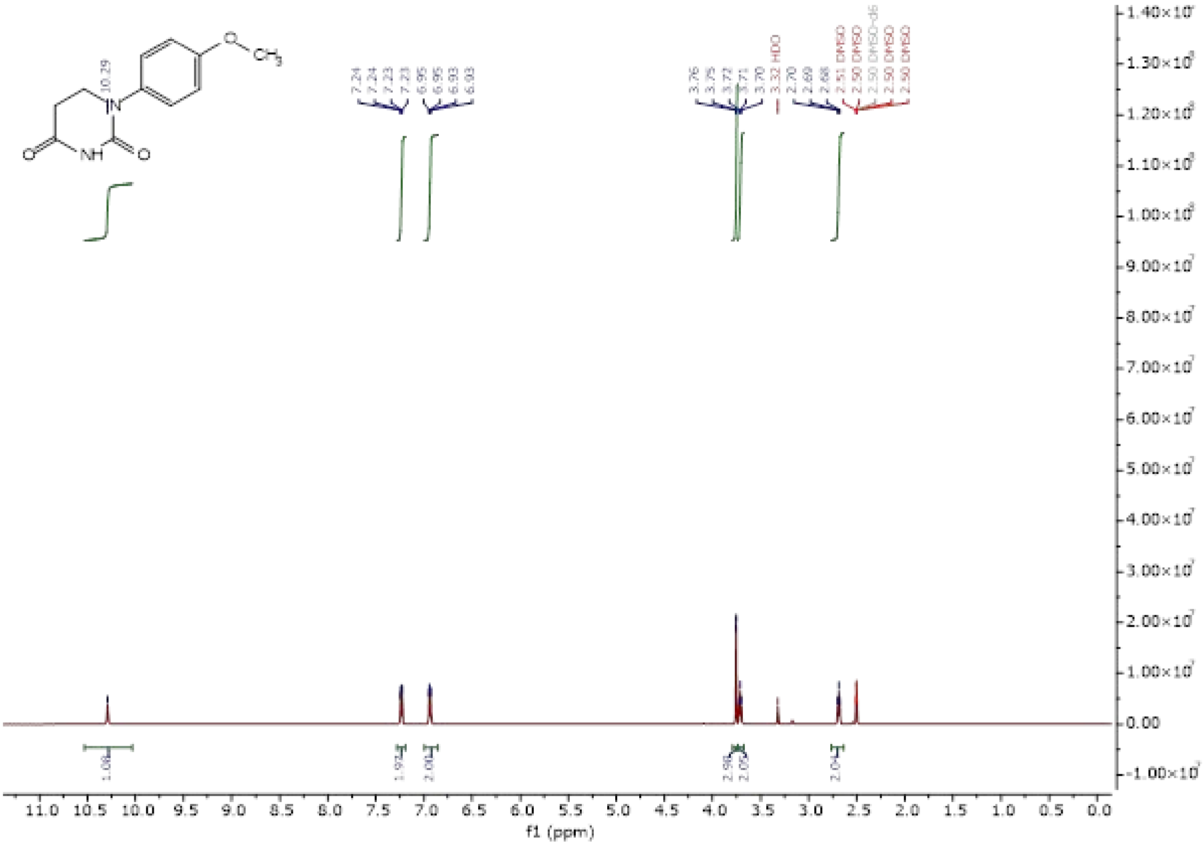

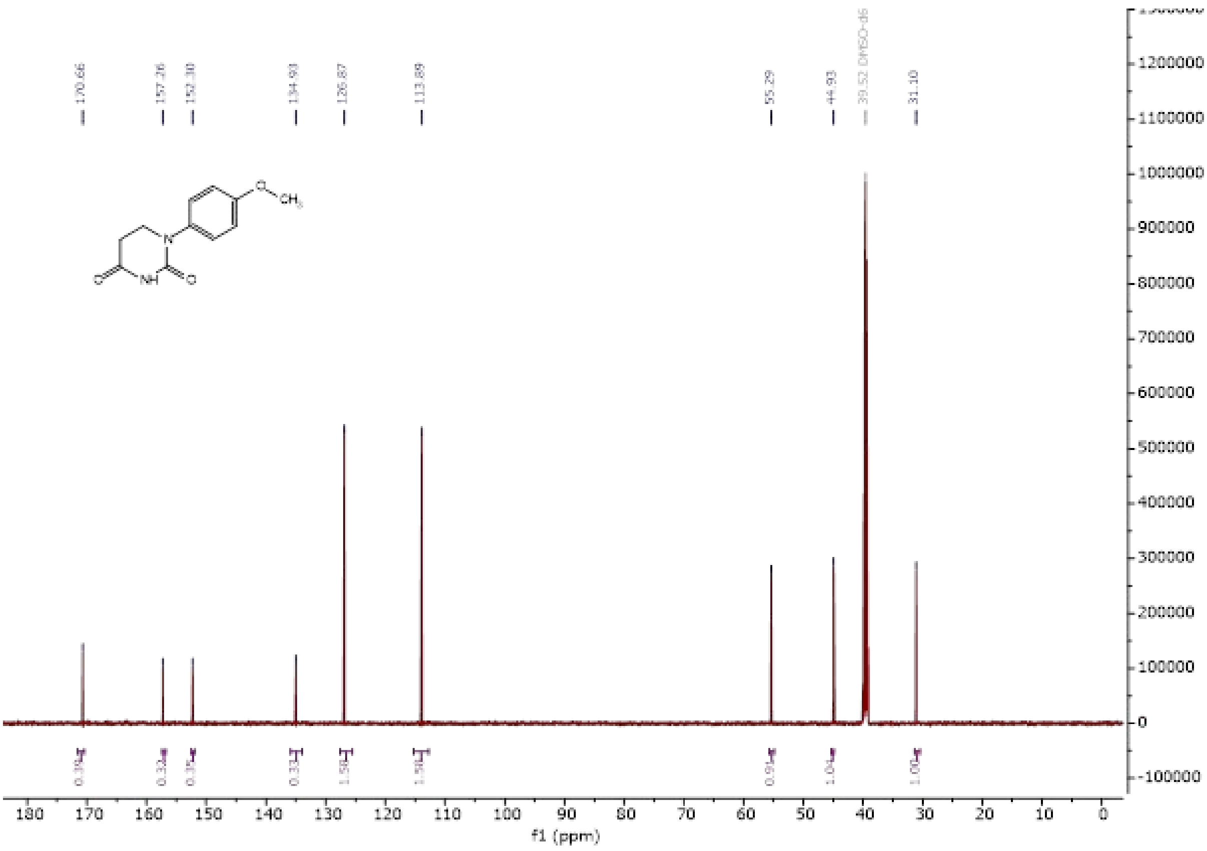

57 - ^{1}H NMR (600 MHz, DMSO-d_6) and ^{13}C NMR (151 MHz, DMSO-d_6)

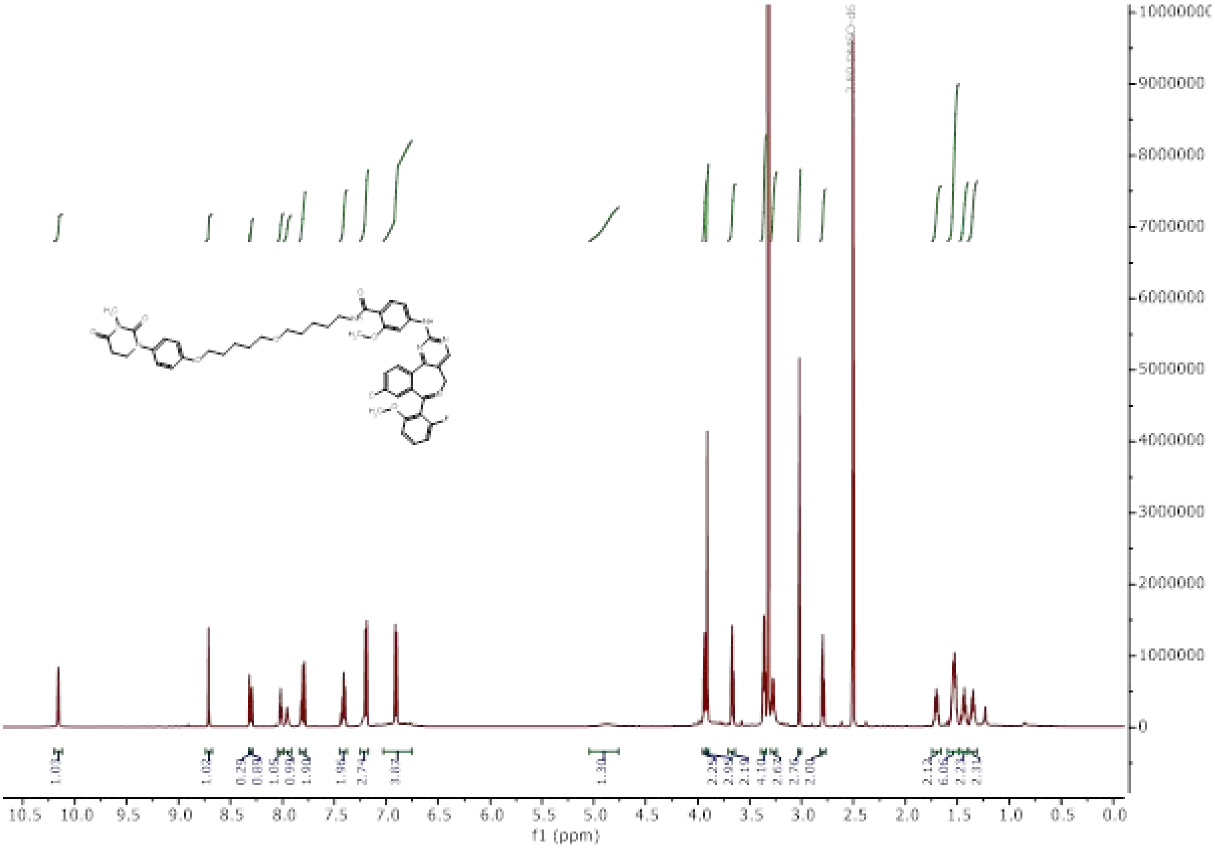

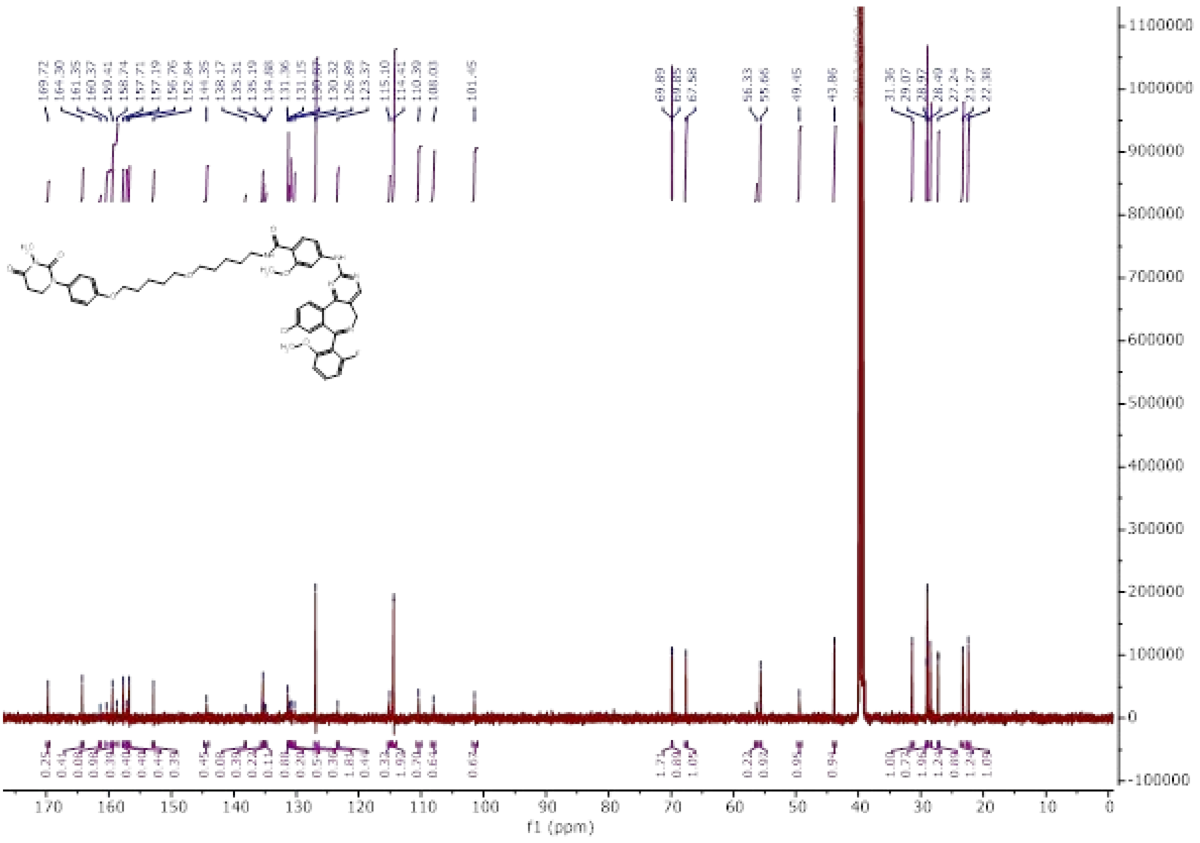

HPLC Traces for Synthesized Compounds

CCT400028

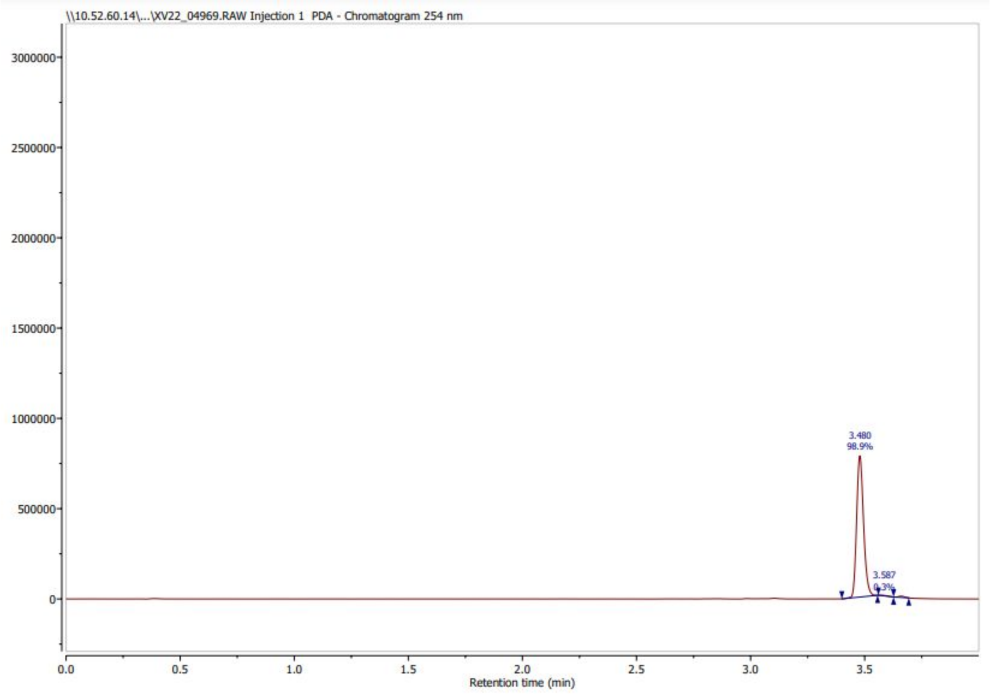

42

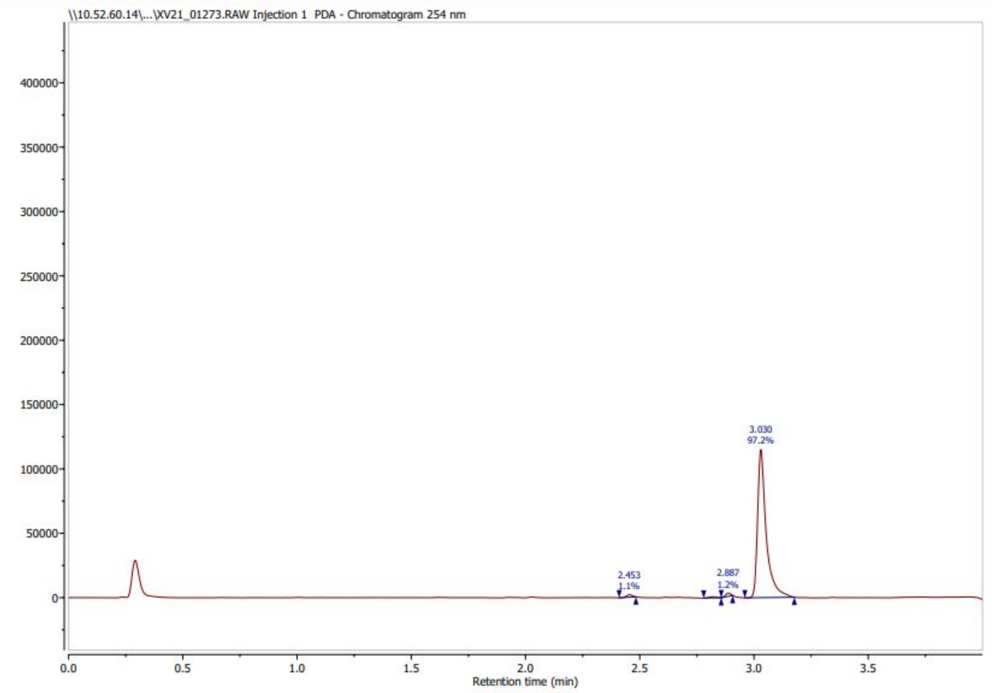

43

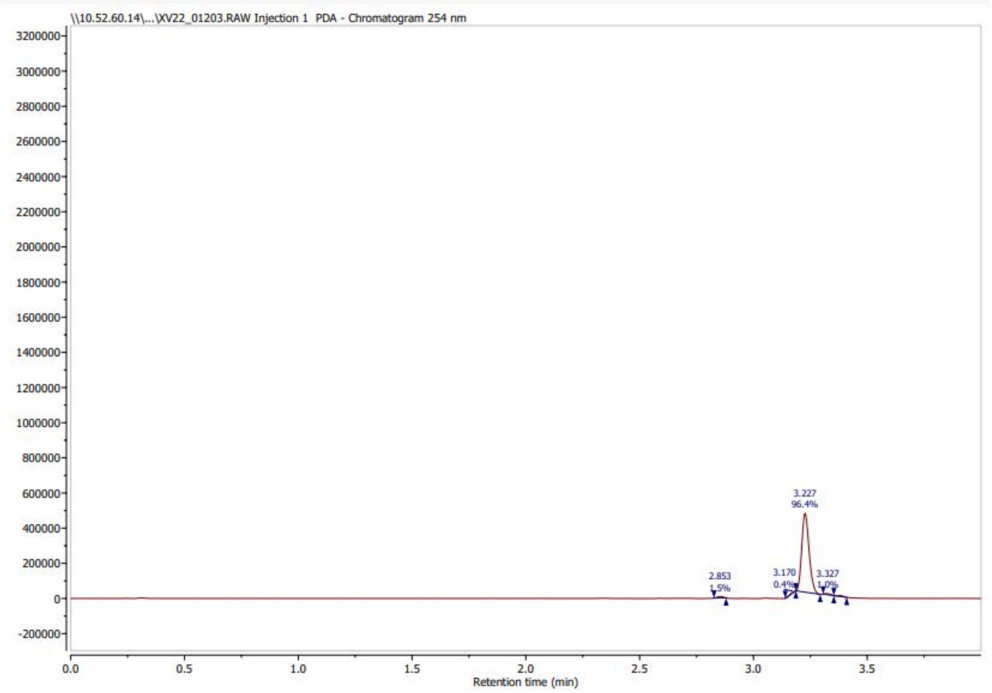
\\10.52.60.14\...\XV22_01203.RAW Injection 1 PDA - Chromatogram 254 nm
3.227
96.4%
2.853
1.5%
3.170
0.4%
3.327
1.0%
Retention time (min)

44

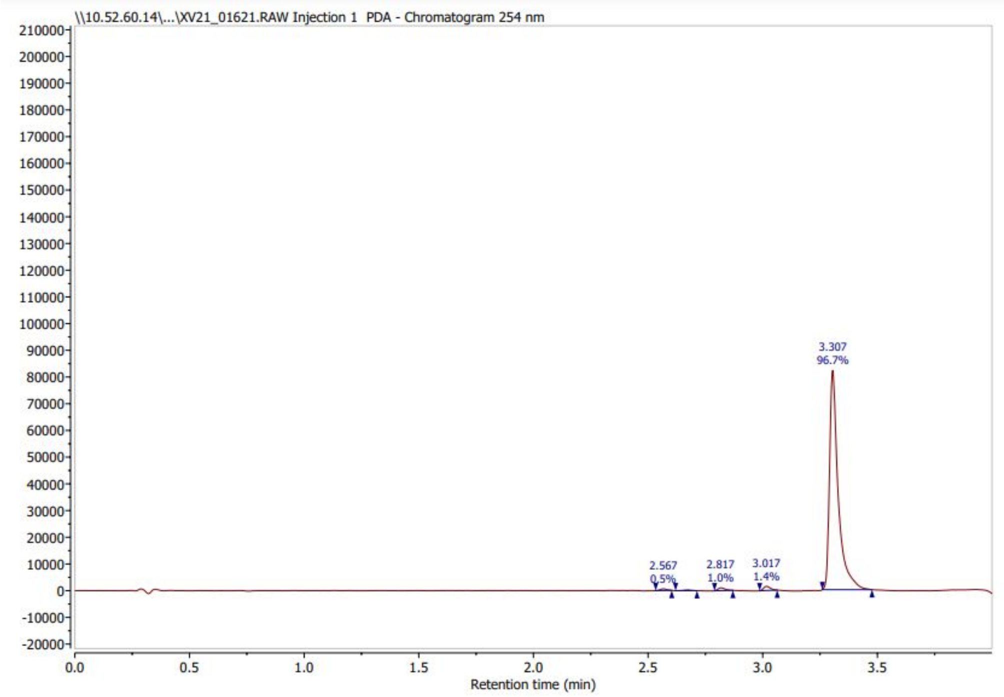
\\10.52.60.14\...\XV21_01621.RAW Injection 1 PDA - Chromatogram 254 nm
3.307
96.7%
2.567
0.5%
2.817
1.0%
3.017
1.4%
Retention time (min)

45

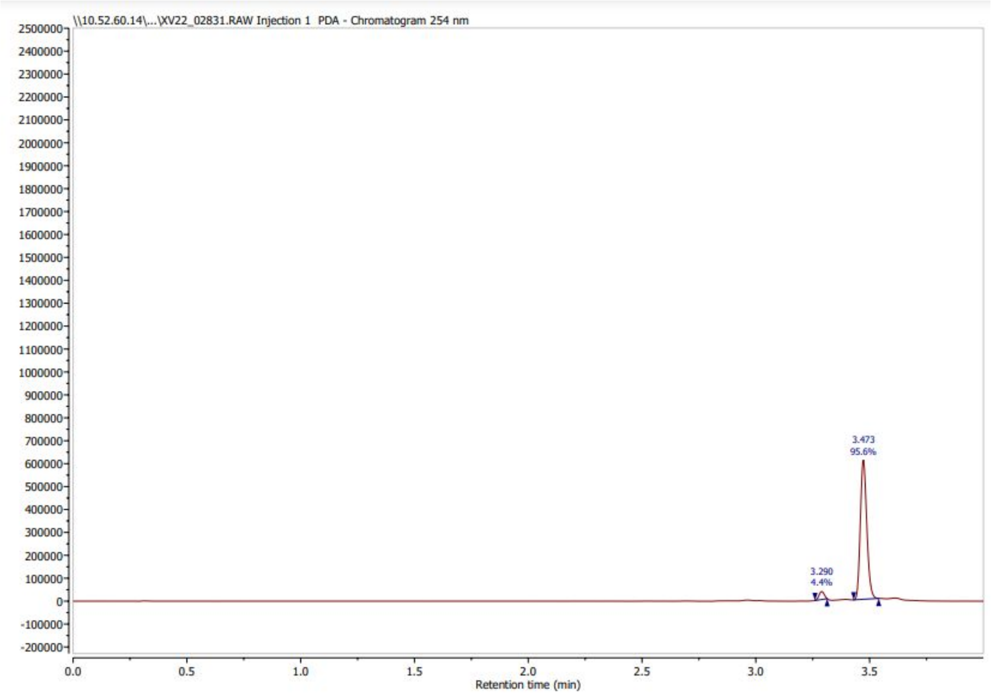
\\10.52.60.14\...\XV22_02831.RAW Injection 1 PDA - Chromatogram 254 nm
3.473
95.6%
3.290
4.4%
Retention time (min)

46

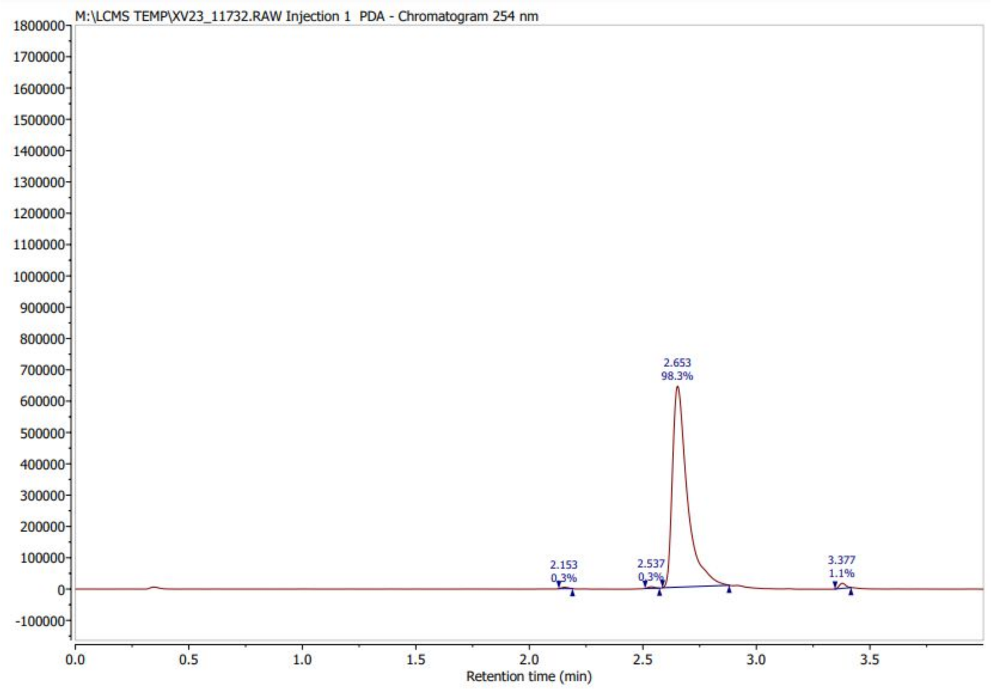
M:\LCMS TEMP\XV23_11732.RAW Injection 1 PDA - Chromatogram 254 nm
2.653
98.3%
2.153
0.3%
2.537
0.3%
3.377
1.1%
Retention time (min)

47

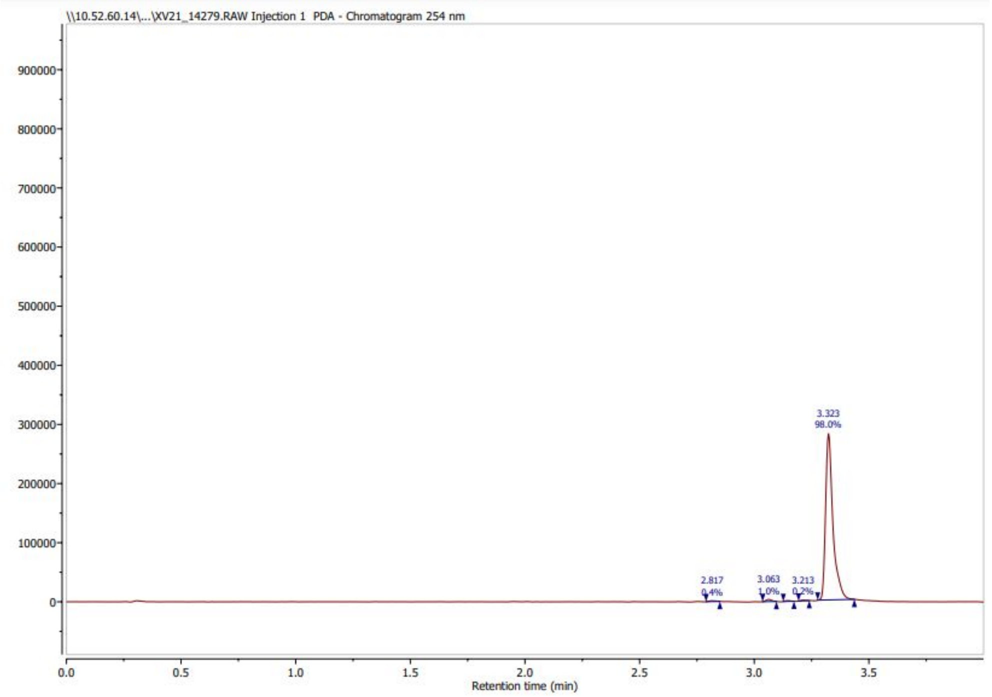
\\10.52.60.14\...\XV21_14279.RAW Injection 1 PDA - Chromatogram 254 nm
3.323
98.0%
2.817
0.4%
3.063
1.0%
3.213
0.2%
Retention time (min)

48

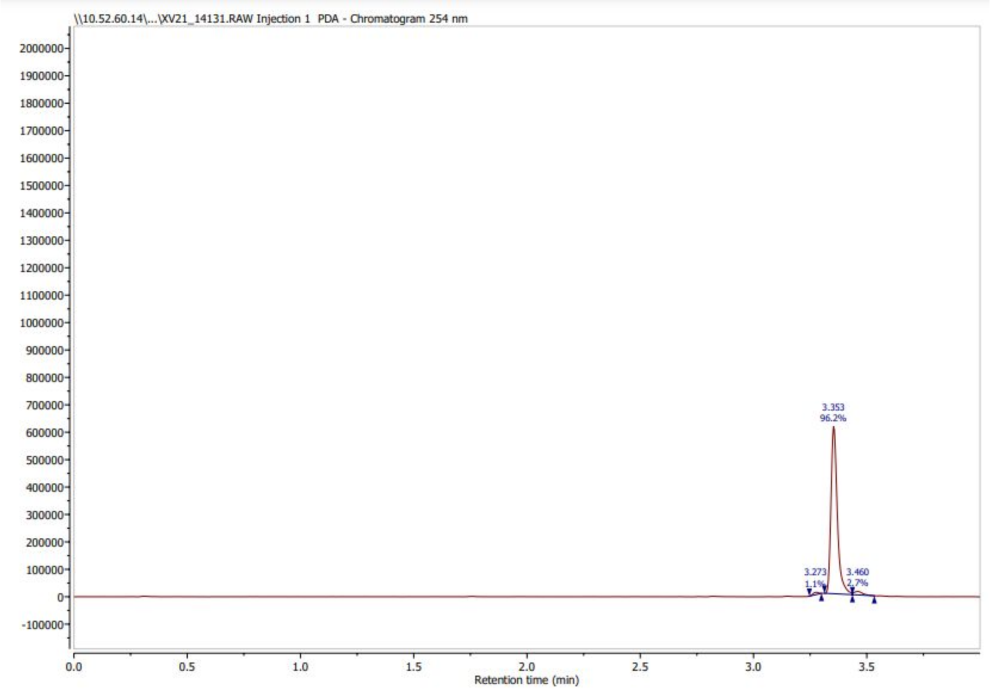
\\10.52.60.14\...\XV21_14131.RAW Injection 1 PDA - Chromatogram 254 nm
3.353
96.2%
3.273
1.1%
3.460
2.7%
Retention time (min)

49

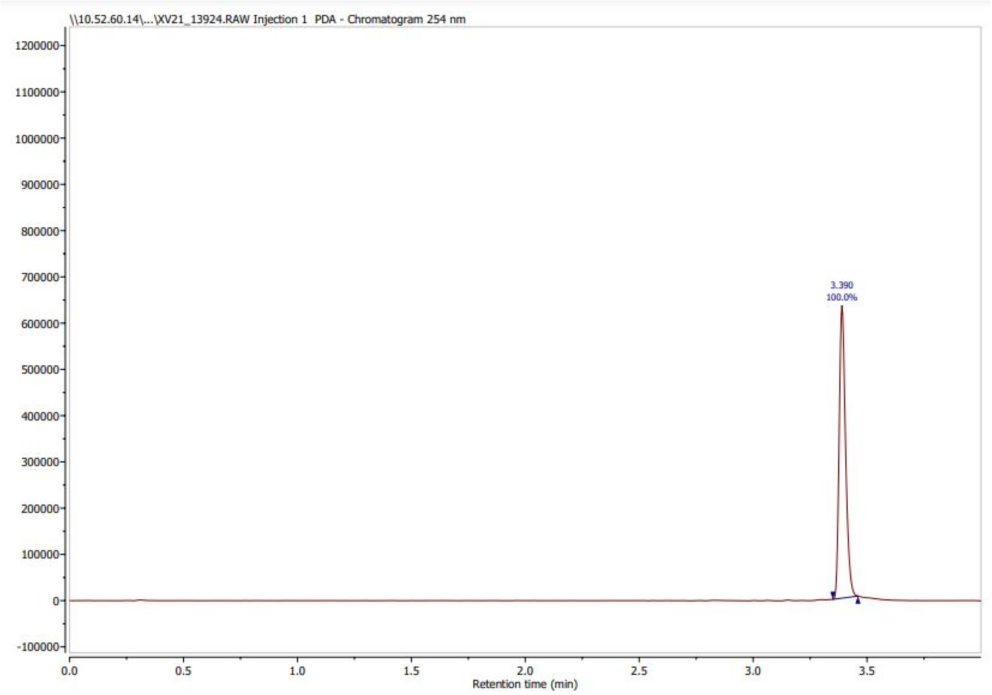
\\10.52.60.14\...\XV21_13924.RAW Injection 1 PDA - Chromatogram 254 nm
1200000
1100000
1000000
900000
800000
700000
600000
500000
400000
300000
200000
100000
0
-100000
3.390
100.0%
0.0
0.5
1.0
1.5
2.0
2.5
3.0
3.5
Retention time (min)

50

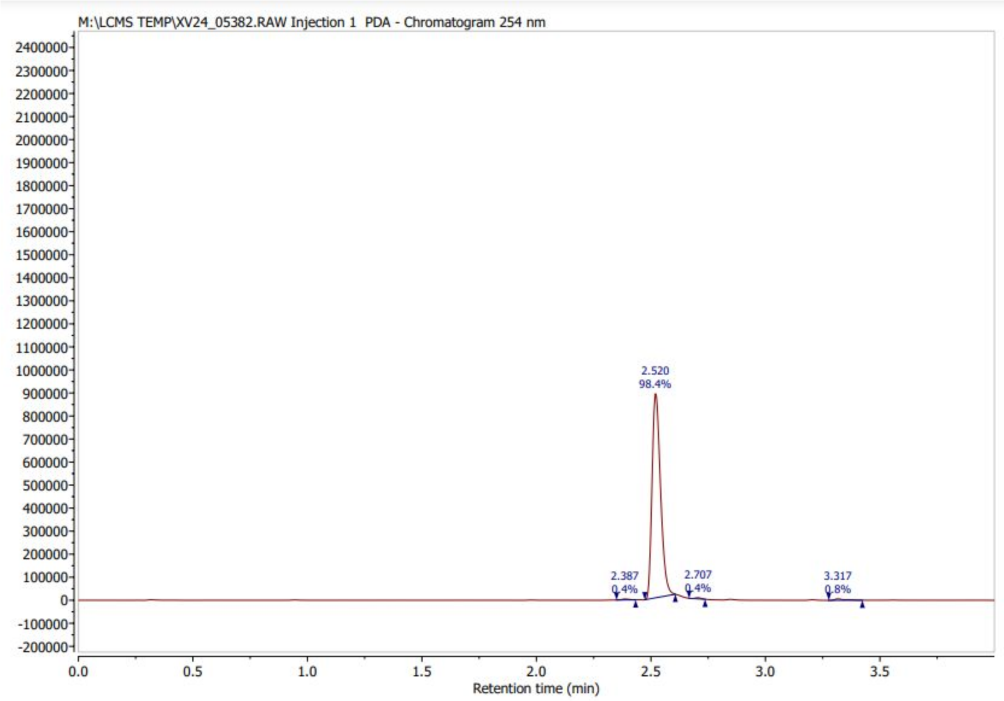
M:\LCMS TEMP\XV24_05382.RAW Injection 1 PDA - Chromatogram 254 nm
2400000
2300000
2200000
2100000
2000000
1900000
1800000
1700000
1600000
1500000
1400000
1300000
1200000
1100000
1000000
900000
800000
700000
600000
500000
400000
300000
200000
100000
0
-100000
-200000
2.520
98.4%
2.387
0.4%
2.707
0.4%
3.317
0.8%
0.0
0.5
1.0
1.5
2.0
2.5
3.0
3.5
Retention time (min)

51

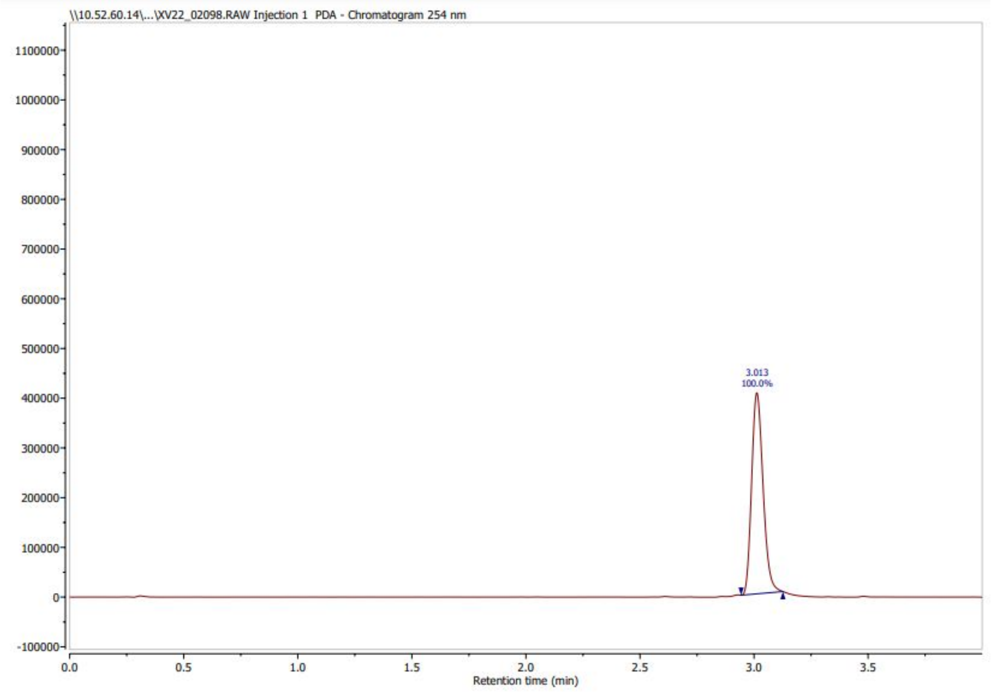

\\10.52.60.14\...\XV22_02098.RAW Injection 1 PDA - Chromatogram 254 nm
1100000
1000000
900000
800000
700000
600000
500000
400000
300000
200000
100000
0
-100000
3.013
100.0%
0.0
0.5
1.0
1.5
2.0
2.5
3.0
3.5
Retention time (min)

55

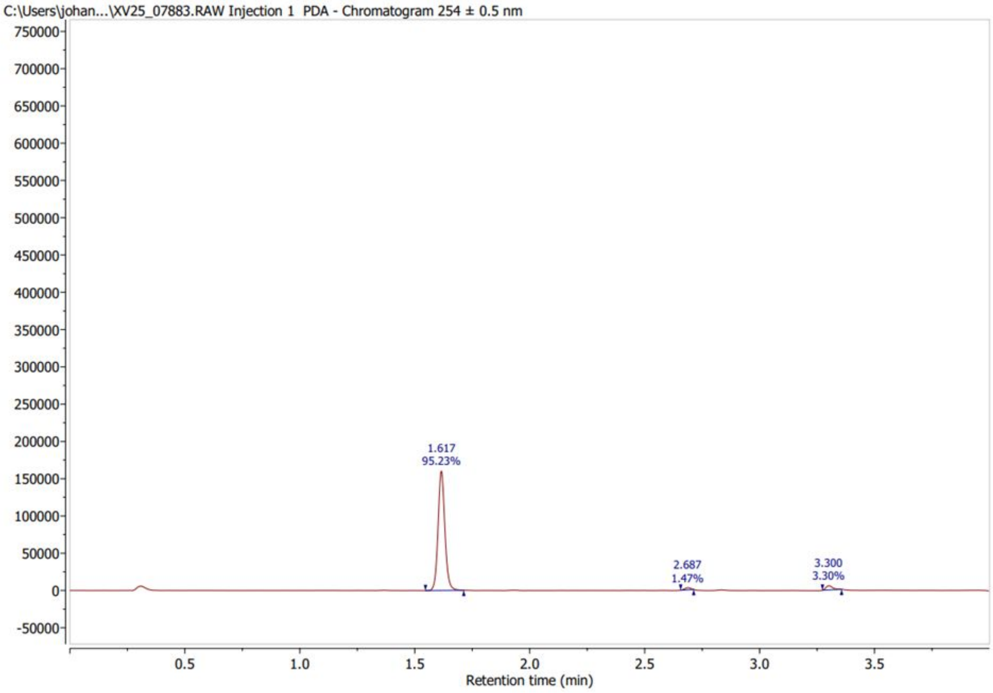

C:\Users\johan...\XV25_07883.RAW Injection 1 PDA - Chromatogram 254 ± 0.5 nm
750000
700000
650000
600000
550000
500000
450000
400000
350000
300000
250000
200000
150000
100000
50000
0
-50000
1.617
95.23%
2.687
1.47%
3.300
3.30%
0.5
1.0
1.5
2.0
2.5
3.0
3.5
Retention time (min)

56

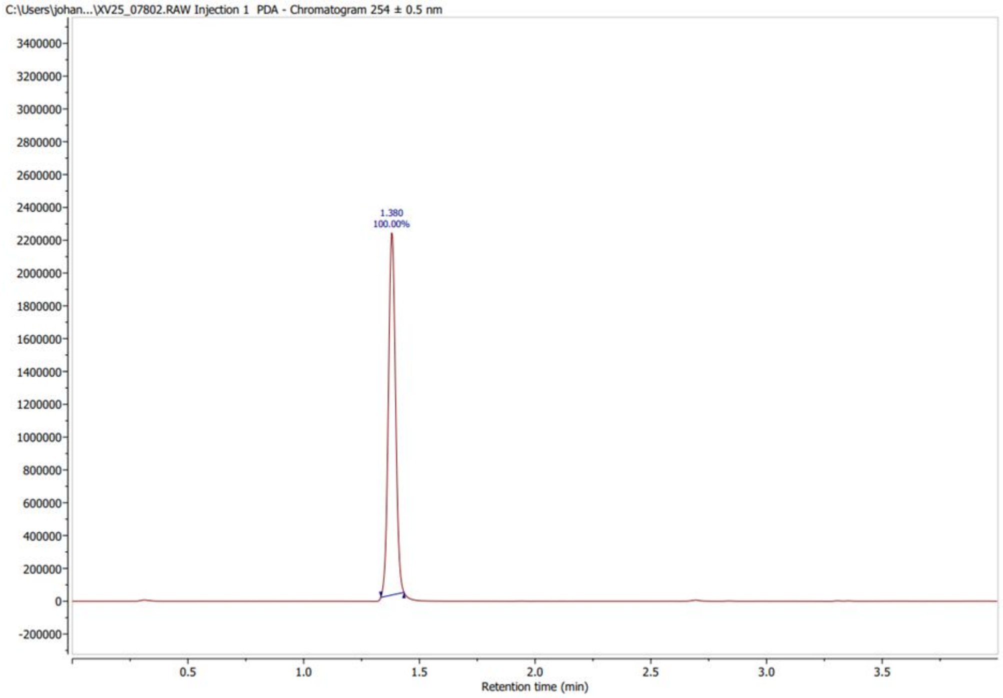
C:\Users\johan...\XV25_07802.RAW Injection 1 PDA - Chromatogram 254 ± 0.5 nm
1.380
100.00%
Retention time (min)

57

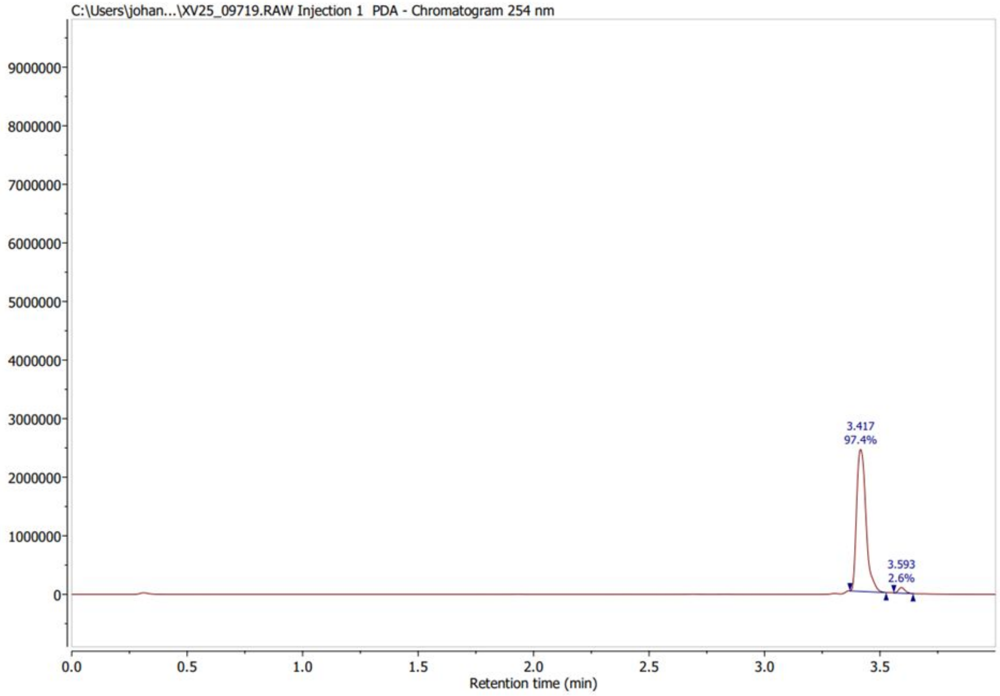
C:\Users\johan...\XV25_09719.RAW Injection 1 PDA - Chromatogram 254 nm
3.417
97.4%
3.593
2.6%
Retention time (min)

References

[1] Bomie, H. A suite of mathematical solutions to describe ternary complex formation and their application to targeted protein degradation by heterobifunctional ligands. *J. Biol. Chem.* **2020**, *295* (45), 15280-15291, DOI:10.1074/jbc.RA120.014715

[2] Douglass, E. F., Miller, C. J., Sparer, G., Shapiro, H., & Spiegel, D. A. A comprehensive mathematical model for three-body binding equilibria. *J. Am. Chem. Soc.* **2013** *135*(16), 6092–6099. DOI:10.1021/JA311795D

[3] Bouguenina, H., Scarpino, A., O'Hanlon, J. A., Warne, J., Wang, H. Z., Wah Hak, L. C., Sadok, A., McAndrew, P. C., Stubbs, M., Pierrat, O. A., Hahner, T., Cabry, M. P., le Bihan, Y. V., Mitsopoulos, C., Sialana, F. J., Roumeliotis, T. I., Burke, R., van Montfort, R. L. M., Choudhari, J., Chopra, R., Caldwell, J., Collins, I.. A Degron Blocking Strategy Towards Improved CRL4CRBN Recruiting PROTAC Selectivity. *ChemBioChem*, **2023,** *24* (23), e202300351. DOI:10.1002/CBIC.202300351

[4] Adhikari, B., Bozilovic, J., Diebold, M., Schwarz, J. D., Hofstetter, J., Schröder, M., Wanior, M., Narain, A., Vogt, M., Dudvarski Stankovic, N., Baluapuri, A., Schönemann, L., Eing, L., Bhandare, P., Kuster, B., Schlosser, A., Heinzlmeir, S., Sotriffer, C., Knapp, S., & Wolf, E. PROTAC-mediated degradation reveals a non-catalytic function of AURORA-A kinase. *Nat. Chem. Biol.* **2020***, 16*, 1179–1188. DOI:10.1038/s41589-020-00652-y

[5] Goedhart, J., & Luijsterburg, M. S. VolcaNoseR is a web app for creating, exploring, labeling and sharing volcano plots. *Scientific Reports*, **2020**, *10* (1), 1–5. DOI:10.1038/S41598-020-76603-3

[6] Perez-Riverol, Y., Bandla, C., Kundu, D. J., Kamatchinathan, S., Bai, J., Hewapathirana, S., John, N. S., Prakash, A., Walzer, M., Wang, S., Vizcaíno, J. A. The PRIDE database at 20 years: 2025 update. Nucleic Acids Res **2025,** *53* (D1), D543-D553. doi: 10.1093/nar/gkae1011.

[7] Wang, L., Pratt, J. K., Soltwedel, T., Sheppard, G. S., Fidanze, S. D., Liu, D., Hasvold, L. A., Mantei, R. A., Holms, J. H., McClellan, W. J., Wendt, M. D., Wada, C., Frey, R., Hansen, T. M., Hubbard, R., Park, C. H., Li, L., Magoc, T. J., Albert, D. H., Lin, X., Warder, S., Kovar, P., Huang, X., Wilcox, D., Wang, R., Rajaraman, G., Petros, A., Hutchins, C., Panchal, S., Sun, C., Elmore, S., Shen, Y., Kati, W., McDaniel, K. F. (2017). Bromodomain targeting degronimers for target protein degradation. WO2017197056A1

[8] Robbins, D. W., Sands, A. T., Mcintosh, J., Mihalic, J., Wu, J., Kato, D., Weiss, D., & Peng, G. (2019). Bifunctional compounds for degrading btk via ubiquitin proteosome pathway. WO2020081450A1.

[9] Stroek, W., Keilwerth, M., Pividori, D. M., Meyer, K., & Albrecht, M. An Iron-Mesoionic Carbene Complex for Catalytic Intramolecular C-H Amination Utilizing Organic Azides. *Journal of the American Chemical Society* 2021 *143* (48), 20157–20165. DOI:10.1021/JACS.1C07378

[10] Ronnebaum, J. M., & Luzzio, F. A. Synthesis of 1,2,3-triazole 'click' analogues of thalidomide. *Tetrahedron* **2016** *72*(40), 6136–6141. DOI:10.1016/J.TET.2016.07.019

[11] Phillips, A. J., Nasveschuk, C. G., Henderson, J. A., Liang, Y., He, M., Duplessis, M., & Chen, C.-L. (2018). *N/o-linked degrons and degronimers for protein degradation*. WO2018237026A1.

[12] Min, J., Mayasundari, A., Keramatnia, F., Jonchere, B., Yang, S. W., Jarusiewicz, J., Actis, M., Das, S., Young, B., Slavish, J., Yang, L., Li, Y., Fu, X., Garrett, S. H., Yun, M. K., Li, Z., Nithianantham, S., Chai, S., Chen, T., Shelat, A., Lee, R., Nishiguchi, G., White, S., Roussel, M., Potts, P., Fischer, M., Rankovic, Z. Phenyl-Glutarimides: Alternative Cereblon Binders for the Design of PROTACs. *Angewandte Chemie International Edition* **2021**, *60*(51), 26663–26670. DOI10.1002/ANIE.202108848

[13] Shinohara, T., Iwata, S., Suzuki, M., Arai, K., Ito, N., & Chiba, T. (2019). *Heterocyclic compounds for the treatment of epilepsy*. WO2020111263A1.

[14] Bonger, K. M., van den Berg, R. J. B. H. N., Heitman, L. H., IJzerman, A. P., Oosterom, J., Timmers, C. M., Overkleeft, H. S., & van der Marel, G. A. Synthesis and evaluation of homo-bivalent GnRHR ligands. *Bioorg. Med. Chem.* **2007** *15* (14), 4841–4856. DOI:10.1016/J.BMC.2007.04.065

[15] Nixon, I. D. G., Bateman, J. M., Michaelides, I. N., Fairley, G., Pemberton, M. J., Braybrooke, E. L., Sutton, K., & Lindsay-Scott, P. J. One-Step Regioselective Synthesis of *N*-1-Substituted Dihydrouracils: A Motif of Growing Popularity in the Targeted Protein Degradation Field. *The Journal of Organic Chemistry* **2024**, *89* (24), 18301–18312. doi.org/10.1021/ACS.JOC.4C02136
